# Proliferation of Murine Midbrain Neural Stem Cells Depends upon an Endogenous Sonic Hedgehog (Shh) Source

**DOI:** 10.1371/journal.pone.0065818

**Published:** 2013-06-11

**Authors:** Constanza Martínez, Víctor Hugo Cornejo, Pablo Lois, Tammy Ellis, Natalia P. Solis, Brandon J. Wainwright, Verónica Palma

**Affiliations:** 1 Laboratory of Stem Cells and Development, FONDAP Center for Genome Regulation, Faculty of Science, University of Chile, Santiago, Chile; 2 The Institute for Molecular Bioscience, The University of Queensland, Brisbane, Queensland, Australia; Wayne State University, United States of America

## Abstract

The Sonic Hedgehog (Shh) pathway is responsible for critical patterning events early in development and for regulating the delicate balance between proliferation and differentiation in the developing and adult vertebrate brain. Currently, our knowledge of the potential role of Shh in regulating neural stem cells (NSC) is largely derived from analyses of the mammalian forebrain, but for dorsal midbrain development it is mostly unknown. For a detailed understanding of the role of Shh pathway for midbrain development *in vivo*, we took advantage of mouse embryos with cell autonomously activated Hedgehog (Hh) signaling in a conditional Patched 1 (Ptc1) mutant mouse model. This animal model shows an extensive embryonic tectal hypertrophy as a result of Hh pathway activation. In order to reveal the cellular and molecular origin of this *in vivo* phenotype, we established a novel culture system to evaluate neurospheres (nsps) viability, proliferation and differentiation. By recreating the three-dimensional (3-D) microenvironment we highlight the pivotal role of endogenous Shh in maintaining the stem cell potential of tectal radial glial cells (RGC) and progenitors by modulating their Ptc1 expression. We demonstrate that during late embryogenesis Shh enhances proliferation of NSC, whereas blockage of endogenous Shh signaling using cyclopamine, a potent Hh pathway inhibitor, produces the opposite effect. We propose that canonical Shh signaling plays a central role in the control of NSC behavior in the developing dorsal midbrain by acting as a niche factor by partially mediating the response of NSC to epidermal growth factor (EGF) and fibroblast growth factor (FGF) signaling. We conclude that endogenous Shh signaling is a critical mechanism regulating the proliferation of stem cell lineages in the embryonic dorsal tissue.

## Introduction

The vertebrate brain is a complex and highly organized structure with numerous neurons and glial cells. During development undifferentiated progenitor cells proliferate from neural stem cells (NSC) and gradually restrict their fates according to environmental cues. Differentiated cells are arranged precisely to accomplish their function and to maintain integrity as a whole brain. Secreted and membrane-bound molecules convey the information between cells and the secreted glycoprotein Sonic Hedgehog (Shh) is one such signaling molecule that has been demonstrated to control many aspects of central nervous system ontogeny. In contrast to its role in early neural patterning and differentiation of the entire ventral axis of the central nervous system, it appears that during late development Shh acts as a mitogen, modulating cell proliferation in the dorsal brain [1]–[3]. By late embryogenesis, Shh expression can be detected in the cerebellum, amygdala, dentate gyrus of the hippocampus, tectal plate, olfactory bulb and neocortex [1], [2], [4]–[8].

Shh, in conjunction with epidermal growth factor (EGF) and fibroblast growth factor (FGF), and endogenous cues regulates the self-renewal ability versus differentiation of embryonic and adult stem/progenitor cells and their progenies in the proliferative neuroepithelium [2], [9], [10]. The sum of all cellular and molecular factors that interact with and regulate the NSC constitutes the three-dimensional (3-D) microenvironment; the so-called stem cell “niche” [11]. Although work has been done to characterize the NSC niche, the precise interactions between signaling molecules involved in their proliferation have not been established. In the case of Shh it has been proposed that by late embryogenesis Shh-producing cells are located in the neocortical and tectal plates since expression of the ligand is not found in the proliferative ventricular zone (VZ) [12].

Canonical Shh signaling is transduced through the transmembrane receptors Patched (Ptc1) and Smoothened (Smo). The inhibition of Smo by Ptc1 is relieved by Shh, thus allowing for transcription of downstream target genes via the Gli zinc-finger transcription factor family. In mouse, the three Gli proteins have distinct biochemical functions and *in vivo* requirements [13]–[15].

Here, we use *in vivo* and *in vitro* approaches to determine whether the tectal neuroepithelium constitutes a mitogenic niche modulated by Shh. To asses the role of Shh signalling in dorsal midbrain (tectum/prospective superior colliculi in mammals) development *in vivo*, we utilized a conditional Ptc1 allele (*Ptc1^Lox/Lox^*) in association with a Nestin Cre Recombinase allele (*Nestin^Cre^*) which, following deletion of Ptc1, results in cell autonomous Hedgehog (Hh) pathway activation in Nestin-positive neural progenitors [16]. As a consequence we observed significant perturbation of tectal proliferation and patterning. Additionally, we seeded NSC from the embryonic dorsal mesencephalon into collagen type-I gels to reveal the endogenous influence of the key growth factor Shh in proliferation of neurospheres (hereafter referred as nsps) grown in the 3-D environment. Our results clearly indicate that the stem cell and progenitor population produces Shh. This Shh signaling is critical for both the modulation of the number of radial glial cells (RGC) and for the proliferation of neuronal precursors in the dorsal mesencephalon.

## Materials and Methods

### Ethics Statement

All animal procedures were in accordance with the Chilean and Australian legislation and were approved by Institutional Animal Care and Use Committees (University of Chile and University of Queensland).

### Mice and Treatment Reagents

C57/BL6 mice (E17.5–E18.5) were used for all *in vitro* assays. We used the dorsal midbrain region (prospective superior colliculi) for cell cultures. Recombinant octyl-modified Shh-N protein was used at 1.5 µg/ml or 3.3 µg/ml (R&D Systems). Other treatments included Hh inhibitor Cyclopamine (Cyc) at 5 µM and 10 µM (Infinity Pharmaceuticals, Inc.), Hh agonist Purmorphamine (Pur) at 10 µM (Infinity Pharmaceuticals, Inc.), EGF 1 and 10 ng/ml (human recombinant, Invitrogen) and/or FGF-2 at 1 and 10 ng/ml (Invitrogen).

Conditional mice carrying a central nervous system-specific deletion of Ptc1 were obtained by breeding animals carrying the conditional *Ptc1* allele (*Ptc1^Lox/Lox^*) [16] with Nestin-Cre transgenic mice (*Nestin^cre^*) and genotyping confirmation was carried out as outlined [17].

### Histology, Immunohistochemistry and *in situ* Hybridization of *Ptc^Lox/Lox^* Mice

Pregnant mice females were injected intraperitoneally with 0.1 ml/g (vol/body weight) of bromodeoxyuridine (BrdU) labelling reagent (Sigma) for 2 hours prior to sacrificing. Embryos were removed, fixed in 4% buffered paraformaldehyde overnight at 4°C, paraffin embedded and cut in 6 µm sections. Sections were stained with Haematoxylin and Eosin (H&E) using standard histological techniques. Non-radioactive *in situ* hybridization was performed using digoxigenin (DIG)-labelled cRNA probes as described [18]. The *Ptc1* probe was designed towards the C-terminal end of the stop codon, detecting both wild-type and mutant *Ptc1* transcript [18]. Sections were incubated in either rabbit anti-Group B1 Sox (1∶200; gift from H. Kondoh); rabbit anti-Hes1 (1∶400; gift from T. Sudo); rat anti-Msi1 (1∶250; gift from H. Okano), mouse anti-Nestin (1∶250, Chemicon); overnight at 4°C as previously described [18]. BrdU-immunohistochemistry was performed on paraffin-embedded sections as described in [16] using mouse anti-BrdU (1∶150; Pharmingen).

### Tectal Explant and nsps Cultures

All mice explants were derived at E17.5–E18.5 from the dorsal midbrain region (prospective superior colliculi), cultured overnight on floating filters in explant serum/free Neurobasal media (Invitrogen) prior to experimental stimulation, and collected 48 hours after treatment. Nsps were isolated from dorsal midbrain by standard procedures and incubated in tectal nsp medium, DMEM/F-12 (Invitrogen), containing B27 (Invitrogen), 2 mM glutamine, Heparin 2 µg/ml (Calbiochem) and antibiotics, with 10 ng/ml of EGF and 10 ng/ml of FGF-2 unless otherwise noted. Cells were used after at least two sequential passages [2]. Paired cell assays on tectal nsps cultures were performed as outlined [19], with minor modifications. Cells were plated at clonal density on coated coverslips and cultured for 24 hours in 10 ng/ml of EGF in the presence of Cyc or 1 ng/ml of EGF plus Shh. Cell pairs resulting from cell division were visually identified and at least 40 cell pairs were counted in each condition per experiment.

### Preparation of Cell–collagen Constructs and Maintenance in 3-D Cultures

Collagen type-I solution was prepared from rat-tail tendons extracted in 0.5 N acetic acid at 4°C for 1–3 days and centrifuged at 17000×g for 2 hours at 4°C. The solution was dialyzed against one-tenth strength DMEM pH 4.0 for 48 hours and the protein quantified by Bradford assay.

To prepare collagen 3-D cultures, the collagen type-I solution was diluted with DMEM (Gibco) 4X to adjust pH and an amount of cell media to achieve a final collagen concentration of 0.05 mg/ml. Small nsps (approximately 20 cells/nsp; from short term suspension cultures) were added at 2000 nsps/gel. The cell–collagen solutions were placed in 96-well tissue culture plates (0.28 cm) at 37°C and 5% CO_2_ for approximately 10 minutes to allow gel formation. Once the gel had set, 0.15 ml of cell media was added to the top of the gels and the matrix was returned to the incubator. Media was exchanged every 3 days during culturing.

### Proliferation and Differentiation Assays on Collagen Cultures

To evaluate the proliferative effect of Shh on nsps in 3-D cultures, small nsps were inoculated in the presence of EGF and FGF-2 (10 ng/ml). After 2 days media was exchanged to culture media plus Shh alone, Shh+(EGF+FGF-2, 1 ng/ml), using culture media without any growth factors as control. Treatments were for 8 hours; a BrdU pulse (1 µg/ml) was done for the last 2 hours of culture. Pharmacological inhibition with Cyc (5 or 10 µM) was performed in EGF+FGF-2, 10 ng/ml. In a complementary experimental set up gels were treated with EGF+FGF-2 containing media plus 5 µl/ml of 5E1, a Shh blocking antibody. To evaluate a possible role for the Shh pathway in differentiation, nsps in collagen cultures were seeded in the presence of both EFG and FGF-2 (10 ng/ml). After 2 days small nsps were deprived of EFG and FGF-2 and cultured in the presence or absence of Shh alone (3.3 µg/ml) to permit cell differentiation for 2, 7 or 14 days, exchanging media every 3 days. Pharmacological inhibition with Cyc (5 or 10 µM) was performed similarly.

### Immunofluorescence

3-D cultures were fixed with 4% buffered paraformaldehyde followed by immunostaining. Tectal NSC were identified with polyclonal antibodies against Group B1 Sox (1∶400) or guinea pig GLAST (1∶1000, Chemicon); polyclonal rabbit anti-brain lipid-binding protein (Blbp) was used to label RGCs (1∶200, Abcam). For pair assay mouse PKCλ (1∶100, Transduction Labs) and polyclonal EGF-Receptor (EGF-R) (1∶50, Abcam) antibodies were used. Differentiating cells in the 3-D cultures in gain or loss of function conditions, were fixed after 2, 7 or 14 days of treatment. For detection of Shh anti-Shh 5E1 (1∶10, Hybridoma Bank) was used. Ptc1 was detected with polyclonal goat anti-Ptc1 antibody (1∶50, Santa Cruz). Cell death was assayed with polyclonal rabbit anti-cleaved caspase-3 antibody (1∶400, Cell Signaling). DNA damage was identified with rabbit anti-phospho-histone H2A.X antibodies (1∶400, Cell Signaling). All primary antibodies were coupled to secondary antibodies conjugated with Alexa fluor (488 or 546, Invitrogen). Finally, F-actin structure was identified with phalloidin staining (1∶50, Molecular Probes).

### Scanning Electron Microscopy (SEM)

Nsps inmobilized in collagen type-I gels were fixed in 2.5% glutaraldehyde followed by dehydration in a graded series of ethanol (50–100%). After sputter coating with gold–palladium, specimens were examined with a SEM FEI-Quanta 200 [20]. Images were recorded at different magnifications.

### Western Blot

For immunoblot, extracts of 3-D cultures were prepared in lysis buffer (10 mM Tris-HCl, 5 mM EDTA, 150 mM NaCl buffer and 1% Triton X-100) containing proteases and phosphatase inhibitors. Protein extracts were separated on 6% and 10% SDS-PAGE gels [21]. Mouse tissue homogenates of both E18.5 tectum and cerebellum (40 µg) were separated by polyacrylamide gel (12%) electrophoresis. Following transfer, PVDF membranes were incubated with antibodies as correspond. We used rabbit polyclonal antibodies against Sox-2 (1∶500, Abcam), Nestin (1∶1000, Abcam), Blbp (1∶1000, Abcam), CyclinD1 (1∶1000, Santa Cruz biotechnology), Shh (1∶1000, Hybridoma Bank), and α-tubulin (1∶5000, Sigma) or β-actin (1∶5000, Sigma) as loading control. Proteins were visualized using peroxidase coupled secondary antibodies (Jackson Immuno Research) for enhanced chemiluminescence (ECL, Pierce) and quantified by densitometry using the public domain NIH Image J program.

### Alkaline Phosphatase Assay (ALPA)

C3H/10T1/2 (ATCC, CCL-226) cells were grown for 24 hours in DMEM containing 10% FBS. After this, cells were washed and cultured for 24 hours in the presence of conditioned media derived from 2 days cultured tectal nsps. Cyc was used to verify the presence of biologically active Shh in the conditioned media. Pur was used as a positive control. Cells were fixed and washed twice in phosphate buffered saline. ALPA was visualized by incubating cells in BCIP/NBT (Roche Mannheim, Germany) as previously reported [22].

### Statistics

Cells were quantified by counting the number of marker-positive cells as a percentage of DAPI positive cells per field at 40X magnification under epifluorescence (Zeiss microscope model Axiovert 200M), or as a percentage of TO-PRO-3 (Invitrogene) positive cells in confocal Z-stacks (Zeiss Laser Meta confocal microscope), on 6–12 random areas per experiment, from at least 3 independent samples and at least 2 independent counters. Images were captured with a Leica MZ12 dissecting microscope fitted with a Leica DFC300 FX camera, and processed on a Macintosh computer using the public domain NIH Image J program or Adobe Photoshop CS5. All probability values were obtained using the Student’s t-test or ANOVA. P<0.05 was considered to be statistically significant.

## Results

### Shh Signaling is Required for Tectal Progenitor Proliferation in the Developing Dorsal Midbrain in Mice

An increasing amount of evidence has revealed that the Shh/Gli signaling pathway is essential for the growth of both dorsal and ventral regions of the embryonic neural tube. A detailed analysis of the different *Gli* mice mutants implicated Gli2 as the main downstream effector since *Gli2* null mice display a clear reduction in the size of several late developing dorsal brain structures, such as the neocortex, tectum and cerebellum [2]. Furthermore, it has been proposed that a balance between Gli2A- and Gli3R- mediated Shh signaling is instrumental in controlling the size and intricate morphology of all mesencephalic derived structures [14]. However, this study implicated Shh/Gli mainly in dorso-ventral patterning and prevention of cell death and did not address a possible contribution in progenitor proliferation [14].

**Figure 1:**
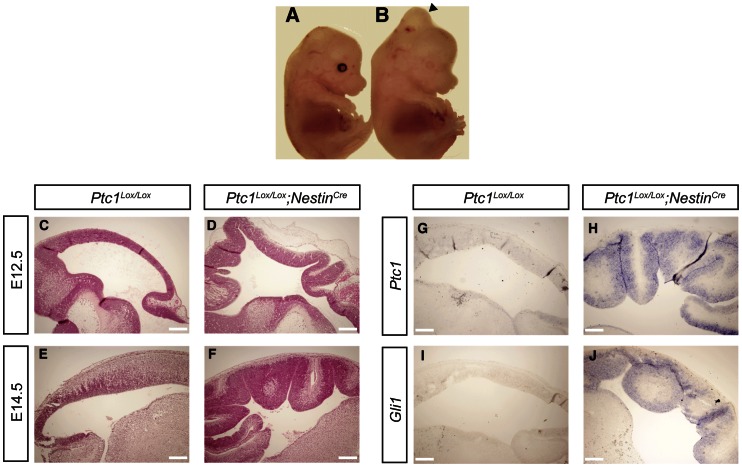
Ptc1 inactivation and consequent Hh pathway activation results in tectal defects. In comparison to *Ptc1^Lox/Lox^* littermates (**A**), E14.5 *Ptc1^Lox/Lox^*;*Nestin^Cre^* embryos are distinguished by protrusion emanating from the tectum (arrowhead) (**B**). H&E-stained sagittal sections at E12.5 (**C, D**) and E14.5 (**E, F**) demonstrating that *Ptc1^Lox/Lox^*;*Nestin^Cre^* embryos (**D, F**) displayed expansion of the tectum neural epithelium resulting in the appearance of *gyri* and *sulci* compared to *Ptc1^Lox/Lox^* littermates (**C, E**). *In situ* hybridization on E14.5 sagittal sections demonstrating that both *Ptc1* (**G, H**) and *Gli1* (**I, J**) transcripts are upregulated in VZ of tectum of *Ptc1^Lox/Lox^*;*Nestin^Cre^* embryos (**H, J**) compared to *Ptc1^Lox/Lox^* littermates (**G, I**). Bar, 100 µm (C–J).

To investigate the role of Hh signaling in the murine tectum *in vivo* we generated a neural progenitor cell specific inactivation of Ptc1 through breeding the floxed Ptc1 mouse (*Ptc1^Lox/Lox^*) [16] to the Nestin-promoter driven Cre-recombinase mouse (*Nestin^Cre^*). Nestin is a ubiquitous marker of neural progenitor cells activated during early neurogenesis. The *Nestin^Cre^* mouse effects recombination in neural progenitors by E12.5 [10], resulting in Ptc1 inactivation and subsequent Hh pathway activation. Mice homozygous for neural progenitor cell specific inactivation of the Ptc1 gene (*Ptc1^Lox/Lox^*;*Nestin^Cre^*) displayed embryonic lethality at E14.5–E15.5. They show a brain hypertrophy phenotype and were easily distinguished by a protrusion emanating from the midbrain (Fig. 1 A and B) whereas both *Ptc1^Lox/Lox^* mice and Ptc1 heterozygous mutant mice were of wild-type phenotype [17].

Histological analysis revealed that *Ptc1^Lox/Lox^*;*Nestin^Cre^* embryos display a dorsal midbrain defect beginning at E12.5. The inferior and superior colliculi comprise the tectum or roof of the midbrain. In contrast to wild-type *Ptc1^Lox/Lox^* mice, where the developing tectum is a smooth structure that forms the roof of the midbrain (Fig. 1 C and E), the inferior and superior colliculi of mutant *Ptc1^Lox/Lox^*;*Nestin^Cre^* mice exhibit extensive folding of the neural epithelium that invades the fourth ventricle resembling *gyri* and *sulci* apparent at both E12.5 (Fig. 1 D) and E14.5 (Fig. 1 F). This infolding disrupts hindbrain structures such as the cerebellum and the ependymal cell-lined central canal (data not shown). Furthermore, the distinction between the inferior and superior colliculi cannot be identified (Fig. 1 F).

To demonstrate activation of Hh signalling in *Ptc1^Lox/Lox^*;*Nestin^Cre^* embryos we analyzed the expression of the universal targets of pathway activation *Ptc1* and *Gli1*. The *Ptc1* in situ probe recognizes both wild-type and floxed alleles. We observed that *Ptc1* and *Gli1* transcripts were expressed at low levels and unable to be detected by *in situ* hybridization in the tectum of E14.5 *Ptc1^Lox/Lox^* mice (Fig. 1 G and I). However, upon Ptc1 inactivation in *Ptc1^Lox/Lox^*;*Nestin^Cre^* mice and consequent Hh pathway activation, we observed upregulation of both *Ptc1* and *Gli1* transcripts in the E14.5 VZ of the developing superior and inferior colliculi (Fig. 1 H and J).

**Figure 2:**
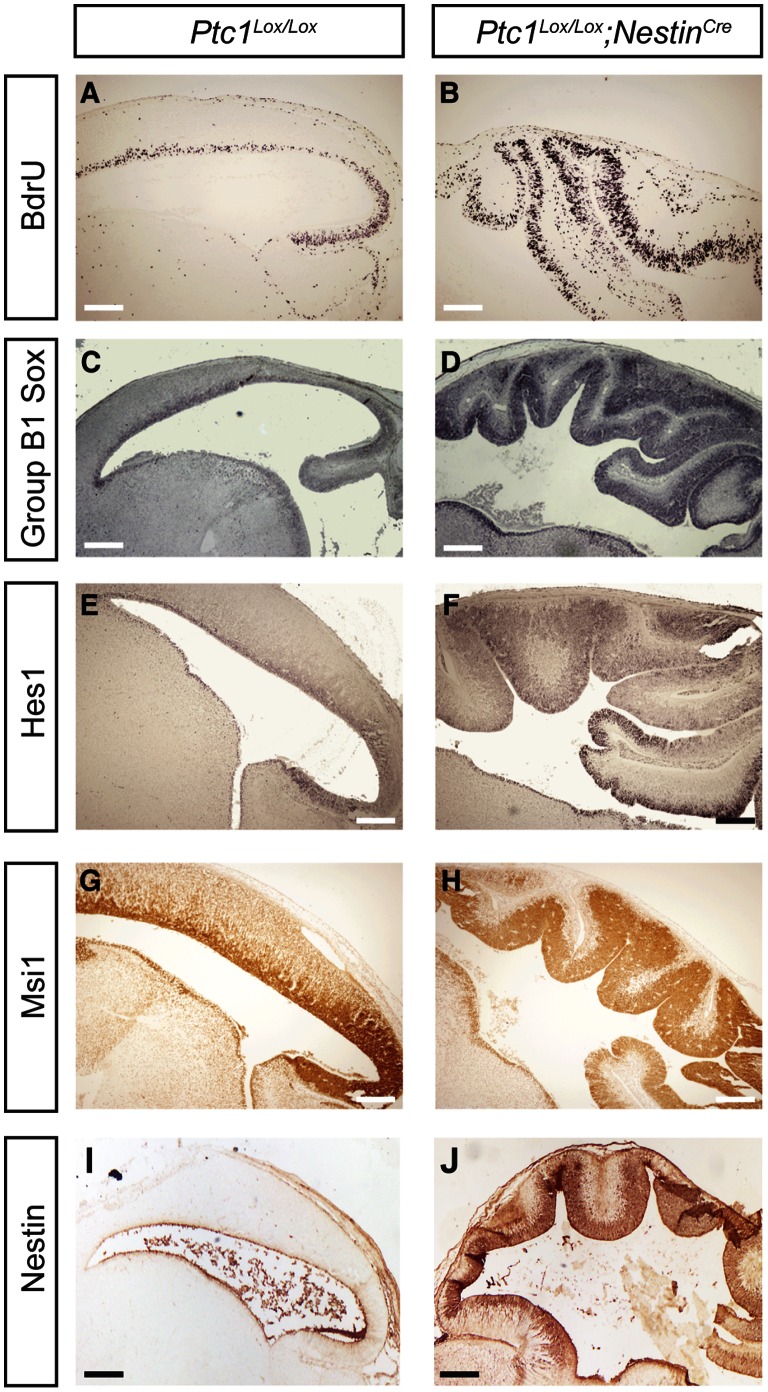
Increased proliferating neural progenitors due to *in vivo* Ptc1 inactivation. In comparison to *Ptc1^Lox/Lox^* littermates (**A**), immunohistochemical analysis demonstrates expansion of proliferating BrdU positive cells in the VZ of the *Ptc1^Lox/Lox^*;*Nestin^Cre^* tectum at E14.5 (**B**). Group B1 Sox (**C, D**), Hes1 (**E, F**), Msi1 (**G, H**) and Nestin (**I, J**) expression is expanded in the VZ of the *Ptc1^Lox/Lox^*;*Nestin^Cre^* tectum (**D, F, H, J**) as compared to the *Ptc1^Lox/Lox^* tectum (**C, E, G, I**). Bar, 100 µm.

To investigate the control of proliferation of progenitor cells by Hh pathway activation we first examined the E14.5 dividing neural precursor by BrdU incorporation. We observed a dramatic increase in proliferating progenitor cells in E14.5 *Ptc1^Lox/Lox^*;*Nestin^Cre^* embryos (Fig. 2 B) as compared to *Ptc1^Lox/Lox^* embryos (Fig. 2 A). In order to identify the cells that are responsible for the neural epithelial expansion observed in the *Ptc1^Lox/Lox^*;*Nestin^Cre^* brains, we examined the expression of markers of neuronal precursors and differentiated neurons in E14.5 embryos. Group B1 Sox proteins, the basic helix-loop-helix (bHLH) transcription factor Hes1 and the RNA-binding protein Musashi1 (Msi1) are highly expressed in neuronal precursors (Fig. 2 C, E and G). We observed increased and expanded expression of Group B1 Sox proteins, Hes1 and Msi1 in the VZ tectum of *Ptc1^Lox/Lox^*;*Nestin^Cre^* brains (Fig. 2 D, F and H) suggesting that the neuronal precursor pool is expanded upon *in vivo* Hh pathway activation. Having observed an increase in progenitor cells, we then analyzed expression of Nestin, verifying that the number of neuronal progenitors in the mutant brains is increased at the same stage of development (Fig. 2 J) in comparison to wild-type mice (Fig. 2 I).

### Shh Signaling Increases Proliferation of Tectal NSC Cultures

To corroborate and investigate the molecular and cellular causes of proliferation of tectal NSC upon activation of Hh pathway in the animal model we expanded tectal NSC as free floating aggregates of cells known as nsps in the presence of EGF and FGF-2. Consequently, were able to produce a consistent, renewable source of E17.5–E18.5 tectal precursors (a portion of which are stem cells). Addition of the specific inhibitor of Hh signaling Cyc reduced proliferation of established plated nsps cultures growing in EGF/FGF-2, suggesting the presence of an endogenous Shh source but addition of recombinant Shh did not increase proliferation (Fig. S1 A and B). Nevertheless, following a 48 hours treatment of tectal explants with exogenous Shh resulted in a two-fold expansion of the BrdU positive population when compared to controls, confirming that both stem cells and progenitors respond to Shh signaling (Fig. S1 C).

### Collagen Type-I Gels have High Biocompatibility with Tectal NSC

The above results suggested strongly that the natural niche, preserved in the explant *ex-vivo* approach, allows the modeling of a dynamic changing environment such as varying growth factors. In order to explore the potential role of Shh as a niche factor, we next aimed to establish a more *in vivo* like environment by developing a 3-D assay based on embedding tectal NSC into collagen type-I gels. To assess the feasibility of the collagen hydrogel system as a delivery vehicle for NSC, we evaluated cell morphology, viability, proliferation and differentiation capacity of nsps cultured within the hydrogels. We obtained a successful 3-D culture of nsps by seeding small nsps (≥20 cells/nsp approximately) into collagen gels in the presence of both EGF and FGF-2. The nsp cultures proliferate actively, maintain its morphology, express NSC markers such as Group B1 Sox transcription factors (1, 2, 3) and GLAST (Fig. S2 A), even in long-term experiments after two weeks of maintaining cultures. Evaluation by SEM indicates that the collagen scaffold appears as a highly porous structure with good interconnections between the pores, a feature necessary to facilitate exchange of nutrients and biomolecules such as Shh (Fig. S2 B). In accordance with many previous reports we verified that Cyc does not produce any toxic effects to the cells since rates of DNA damage, evidenced by low expression of histone H2A.X as well as apoptosis, revealed by low percentage of cleaved caspase-3 positive cells, were similar to those assayed in absence of the drug. Similarly, recombinant Shh treatment did not affect significantly cell viability (Fig. S2 C and D).

### Shh Pathway Maintains RGC and Neural Progenitor Proliferation of Tectal NSC

**Figure 3:**
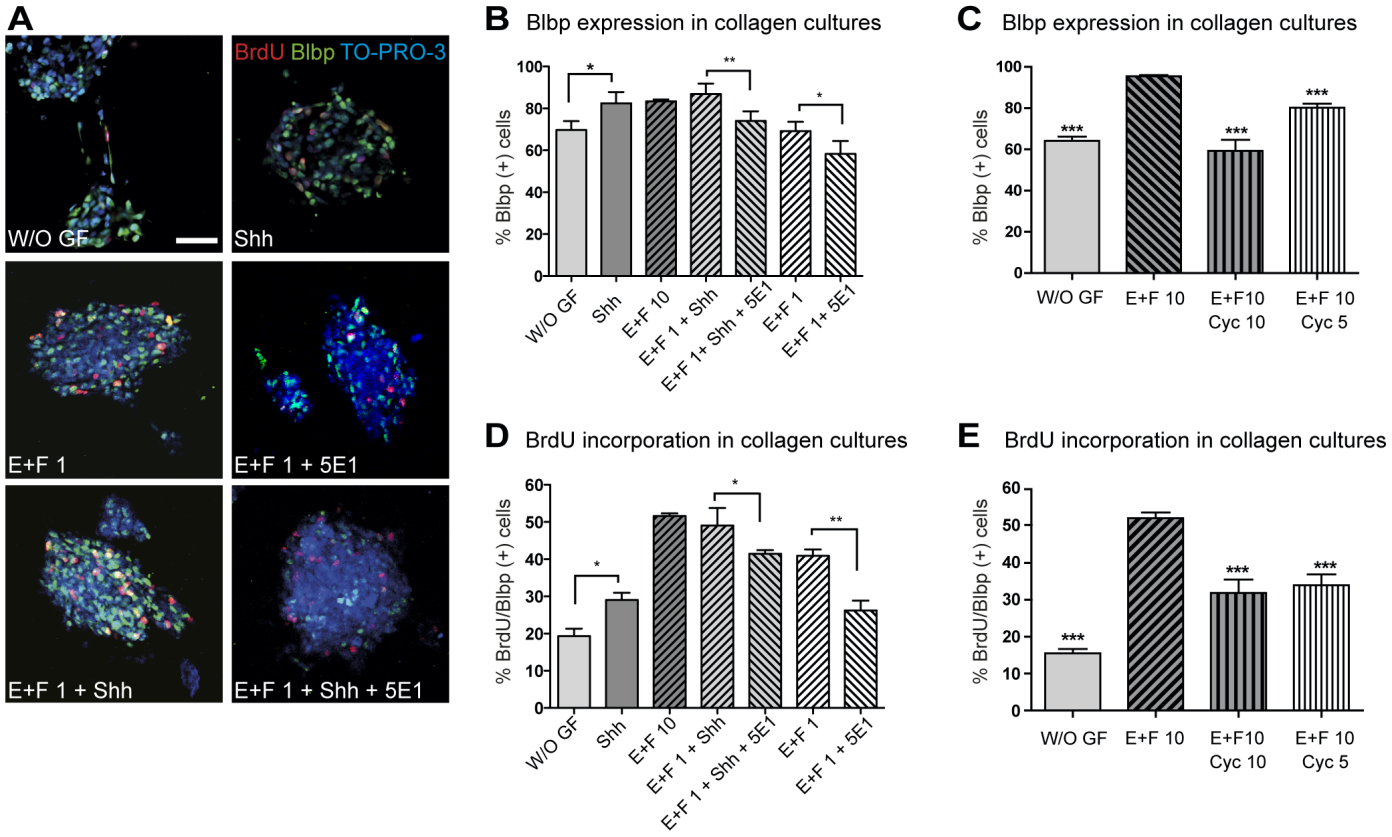
Shh induces tectal NSC proliferation. Nsp suspension cultures were immobilized in collagen type-I gels. Proliferation experiments were performed for 8 hours and included a BrdU (1 µg/ml) pulse for the last 2 hours in the presence of EGF and FGF-2 (used at 1 ng/ml or 10 ng/ml); combined with either Shh (3,3 µg/ml) or Cyc (5 or 10 µM). (**A**) Representative images of double immunostaining for BrdU and the RGC marker Blbp after indicated treatments. Bar, 20 µm. (**B**) Quantification of the percentage of positive cells for Blbp over total numbers of cells, in Z-stacks of confocal microscope. Anti-Shh antibody 5E1 affects both endogenous Shh source as well as recombinant Shh effect resulting in less number of Blbp positive cells. (**C**) Quantification of the effect of endogenous Shh on the number of Blbp positive cells shows significant inhibition after Cyc treatment. (**D**) Histogram showing proliferation of nsps after exposures to treatments as indicated. The anti-Shh antibody 5E1 reduces significantly number of BrdU positive cells in the Blbp positive cell population. (**E**) Quantification of positive cells for BrdU over Blbp positive cells in Z-stacks of confocal microscope treated with Cyc. *, p<0.05; **, p<0.01; ***, p<0.0001. W/O GF: without growth factors, E: EGF, F: FGF-2.

Based on the observation that approximately 50% of the tectal nsps cultured in the presence of traditional mitogens such as EGF and FGF-2 incorporate BrdU even with short pulses, we performed an 8-hour proliferation assay with Shh and evaluated first the putative Shh responsive target cell population. According to our *in vivo* data, we found an increase in the number of Blbp positive cells after Shh treatment. Hence, tectal NSC, and in particular RGC, which are labeled by Blbp, respond to Shh treatment (Fig. 3 A, B). Also, expression of Blbp is similarly reduced in the anti-Shh antibody 5E1 treated cultures (Fig. 3 A, B). Moreover, when evaluating the number of Blbp positive cells after a short term Cyc treatment, a dose dependent decrease was found, with 10 µM being more effective (Fig. 3 A, C).

Of note, in our 3-D cultures addition of Shh alone was sufficient to promote efficient proliferation of RGC when compared to cultures grown without any growth factor. Interestingly, Shh appears to collaborate with the EGF and/or FGF-2 pathways to promote RGC proliferation. At low concentration of both mitogens (1 ng/ml) addition of Shh promotes significant nsp growth (Fig. 3 A and D). In order to confirm that the mature Shh protein resulting from intracellular processing and secreted from nsps into the extracellular compartment is biologically active we blocked Shh by treating with 5E1 antibody and evaluated proliferation. Proliferation was significantly reduced in nsps cultures exposed to 5E1 even in presence of EFG and FGF-2 suggesting that endogenous Shh might mediate its mitogenic effects on responsive tectal NSC in an autocrine and/or paracrine manner (Fig. 3 A and D). Next, we assessed the effect of Cyc by treating at 5 or 10 µM for 8 hours. As shown in Fig. 3 E, BrdU incorporation decreased significantly. Even in the presence of EGF and FGF-2 (both at 10 ng/ml) Cyc reduced RGC proliferation, confirming the likelihood of an endogenous source of Shh in our 3-D cultures.

**Figure 4:**
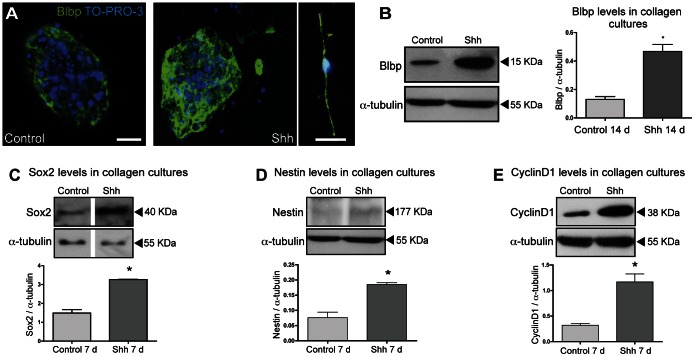
Shh promotes proliferation and maintenance of RGCs. Differentiation experiments were performed during 14 days without addition of EGF or FGF-2. (**A**) Representative images of immunostaining for Blbp after control or Shh treatment. Close-up view of a RGC stained for Blbp treated with Shh. Bar, 20 µm. (**B**) Western blot and densitometry analysis for Blbp expression show higher levels in Shh treated cultures. Western Blot analysis of Sox2 (**C**), and Nestin (**D**) levels after 7 days of treatment with Shh indicate an increase of neural progenitors. (**E**) Western blot of Cyclin D1, a read-out response to Shh pathway activation, indicates an increased proliferation even in absence of other additional growth factors. *, p<0.05.

These data suggest that Shh acts by driving RGC proliferation within tectal nsps cultures. Presumably Shh signaling is influenced considerably by a variety of fine tuning mechanisms and regulating factors, allowing Shh to display a diverse range of functions. Even within the same tissue the response to Shh can be survival, proliferation and/or differentiation, as has been shown by a series of studies using the developing chick neural tube as a model system [3]. In order to confirm if indeed RGC are maintained and/or expanded in presence of Shh we next tested the long-term effect of Shh incubating the 3-D collagen cultures for 14 days in the absence of exogenous EGF or FGF-2. Both by immunolabeling as well as by western blot we verified that indeed Shh is acting on RGCs, the main subpopulation of NSCs at late embryogenesis, since Blbp expression was significantly increased when compared to a control condition without any mitogenic factor (Fig. 4 A and B). Of note, we were able to distinguish more cells with the characteristic RCG morphology after Shh treatment in comparison to control growth factor deprived cultures (Fig. 4 A).

Immunoblots of both Sox2 and Nestin which mark multipotent NSCs during different stages of mouse ontogeny, revealed increased expression in differentiated 3-D collagen cultures after 1-week exposure to Shh (Fig. 4 C and D). We confirmed that neuronal progenitor cells are actively proliferating in response to Shh since expression levels of CyclinD1, described as a direct target of Hh signaling [23] were significantly increased in comparison to the control (Fig. 4 E).

These observations support the interpretation that an endogenous source of Shh acts on RGCs and neuronal precursors during late embryogenesis.

### Shh Promotes Symmetric Divisions of Tectal NSC

When a progenitor cell divides it may either divide symmetrically to give two identical cell types or asymmetrically produce a progenitor and a more differentiated cell. Clearly, this is a key step in developmental biology and tissue repair yet the environmental factors that control the occurrence of these two modes of cell division are largely unknown [24]. Shh can indeed be a candidate, as it seems to exert an important role on the modulation of this process, as we demonstrated previously in neocortical development [17]. In order to determine whether Shh plays a key role in symmetric/assymetric division of tectal stem cells we next performed paired cell assay of tectal nsps at clonal density. EGF-R distribution in pairs of daughter cells, during or immediately after mitosis, was assessed as an established protocol to verify symmetric versus asymmetric division modes [19] (Fig. S3 A and B). Approximately 50% of the pairs showed similar levels of EGF-R staining (symmetric-EGF-R) in both daughter cells. In the other 50%, one of the daughter cells expressed high levels of EGF-R while the other cell exhibited none or very low EGF-R levels (asymmetric-EGF-R). Co-labeling experiments revealed that EGF-R distribution in cell pairs always correlated with that of the apical domain polarity marker, atypical protein kinase C-PKCλ (Fig. S3 B) Strikingly, treatment with Shh increased the number of symmetric divisions by 20% with the opposite result in the presence of Cyc (Fig. S3 A). These results reveal for the first time that Shh can contribute to EGF responsiveness by promoting symmetric distribution of EGF-R between sibling NSC thereby increasing the NSC pool.

### Shh is a Crucial Niche Factor for Tectal NSC Growth

**Figure 5:**
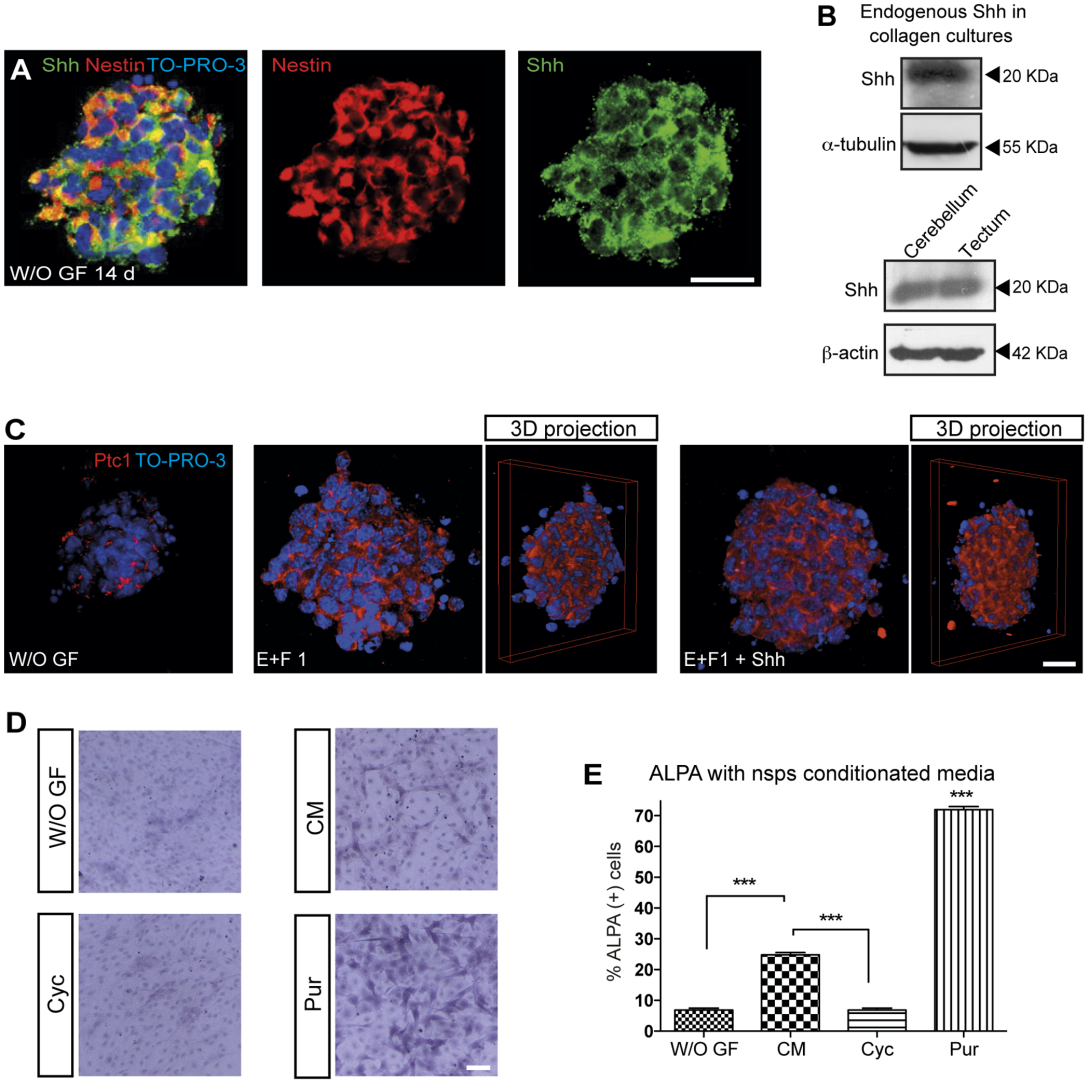
Endogenous Shh acts as a niche factor. (**A**) Nsps expresses high levels of Shh ligand that co-localizes with Nestin, a NSC marker. (**B**) Western blots analysis shows that Shh is expressed in nsps even in control conditions. The Shh ligand is endogenously expressed in E18.5 tectum; cerebellum is shown as a positive control. (**C**) Ptc1 immunostaining images of nsps revealed increased expression of the receptor in the presence of Shh. Image is shown in 3-D projection resulting of a clockwise 135° rotation in the Z-axis. (**D**) Representative images of ALPA of C3H-10T1/2 cells, treated with fresh conditioned serum-free media obtained from 2 day cultured nsp populations, conditioned media plus Cyc, or Pur (used as positive control) in comparison to nsp media without growth factors used as control media. (**E**) Percentage of ALPA positive cells in response to indicated treatments. Bar, 20 µm. *** p<0.0001. W/O GF: without growth factors, E: EGF, F: FGF-2. CM: conditioned media.

A niche integrates cell growth, cell adhesion, and cell-cell signals that mediate the balanced response of NSC cells to their needs. We were able to detect Shh expression in Nestin positive cells within the nsps, revealing an autocrine production of this diffusible signal both by immunolabeling as well as by western blot (Fig. 5 A and B). Importantly, cerebellar and tectal E18.5 explants acutely processed and tested for Shh expression revealed endogenous expression in both tissues favoring thereby the niche hypothesis (Fig. 5 B). That Shh is secreted as a lipid-modified protein suggests the existence of a mechanism to regulate its movement. Additionally, in agreement with these data, Ptc1 immunolabeling of collagen-trapped nsps treated with Shh revealed higher expression of the receptor in comparison to control condition either without growth factors or only EGF plus FGF-2, confirming that indeed cells within the nsps are actively responding to their ligand Shh (Fig. 5 C).

Finally, the possible functional activity of Shh secreted by nsps was evaluated in the Shh reporter cell line (C3H-10T1/2). This experimental approach showed a significant increase in the alkaline phosphatase activity (ALPA) of C3H-10T1/2 cells upon treatment with 48 hours tectal nsp-conditioned media which was further increased by the Hh agonist purmorphamine (Pur) and more importantly, abolished by co-treatment with Cyc (Fig. 5 D and E).

## Discussion

There is now good evidence that Shh signaling is involved in regulation of stem cell proliferation [25]. Our *in vivo* results confirm that Shh at late stages of central nervous system development has an important role acting as a mitogen on the midbrain. Shh is also expressed locally in both the cortex and the cerebellum, two other main regions of the brain that undergo a late burst of proliferation and neurogenesis [4], [6].

*Ptc1^Lox/Lox^*;*Nestin^Cre^* mice die at mid embryogenesis making it impossible to asses the role of Hh signaling at later stages. Stem cells in the multicellular organism are regulated by their environment, which imparts to them properties that may be altered by removal and isolation of the stem cell from this niche [26]. Our approach involved the use of a 3-D scaffold to more closely mimic *in vivo* growth conditions, combined with the addition of growth factors [27], [28]. Within this setting, our work aimed to assess the functional role of Shh in experiments in which differential NSC potencies could be evaluated. An important novel outcome of the present work is the identification of a Shh responsive Ptc1 positive population of E17.5–E18.5 tectal NSC and precursors. We show that RGC proliferation increases in response to exogenous Shh whereas it decreases after addition of Cyc. The rates of apoptosis were not affected in our 3-D cultures indicating that Shh can regulate tectal NSC growth by controlling cell cycle progression and symmetric divisions. Our results show that endogenous Shh is necessary and sufficient to promote tectal nsp proliferation *in vitro* even in absence of other mitogens such as FGF-2 or EGF. Importantly, we show that Shh is produced and concentrated within nsps.

Even if the nervous system proper (i.e. excluding meninges and protective layers of the nerves) does not contain collagen fibers, increasing evidence indicates that collagens are critical elements of its integrity. Collagen type-IV together with collagen type-I is found in fractone, an extracellular matrix structure that is present in the lateral wall of the ventricles. This location is one of the main neural stem cell niches in the adult brain (reviewed in [1], [29]). We demonstrate that our collagen type-I 3-D scaffold favors the concentration of cues and signals such as extracellular matrix factors, cell adhesion receptors (e.g. integrin-β1) and growth factors (e.g. Shh) driving the efficient maturation and differentiation of NSC [30], [31]. In this sense we propose that our 3-D cultures could emulate the niche function, supporting NSCs both physically and nutritionally, by providing growth and extracellular matrix factors.

Niches have evolved to protect and perpetuate the self-renewing, undifferentiated state of the cells within and to regulate the rate of production of committed, tissue-specific progenitors. It is well established that nsps consist predominantly of committed progenitors mixed with differentiated astrocytes and neurons. This mixed cellular environment likely provides a niche that sustains the relatively few stem cells. Therefore, we propose that tectal NSCs, through Shh secretion, may modulate self-renewal of its own cell population. A transient increase in stem/precursor cells by Shh pathway activation could explain a later increase in production of neurons derived from such expanded progenitor pools in the tectum. At late embryonic stages the tectum is indeed in active neurogenesis and as such tectal NSC differentiation might recapitulate events occurring during neurodevelopment. Shh signaling may thus be a mechanism for the regulation of the number of neural stem cells and precursors and most probably the number of neurons derived from these primary progenitors.

Using a collagen type-I 3-D scaffold as a base material, we demonstrate that the growth of NSC can be monitored under various conditions in this microenvironment. Our 3-D culture system has the advantage of maintaining the nsp architecture and therefore allows evaluation of relationships between cells within the same nsp, and characterization of the stem cell niche, something almost impossible to explore in a 2-D plated culture or explant. Our 3-D assay also has advantages because we can isolate NSC without contamination of other growth factors and/or extracellular matrix components, which allows exact evaluation of the function of biomolecules such as Shh.

In summary, to our knowledge, this is the first report giving conclusive evidence of a mechanism by which canonical Shh maintains the self-renewal capacity of the NSC population in the tectum, and it is evolutionary conserved as we demonstrated previously in chicken and zebrafish models [32], [33]. More importantly, since we identify an endogenous source of Shh in tectal NSC we suggest that Shh is acting as a niche factor, favoring the microenvironment by potentiating the response of other growth factors such as EGF and FGF-2. As previously reported in the neocortex, a specific combination of FGF-2, EGF and Shh seems to be a potent synergistic stimulus for tectal NSC proliferation [2].

We propose that the use of biomaterial-based scaffold combined with Shh therefore may be a promising strategy to enhance NSC proliferation and actively contribute to fate determination. Our findings demonstrate that culture of NSCs within a hydrogel system supplemented with appropriate growth factors can maintain their stemness properties and could thus functions as a synthetic equivalent of the stem cell niche [34], [35]. Future studies using this system will allow analysis not only of the effects of the Shh signaling pathway on NSC, but also the specific source of this signal within the niche and how this and other signals collaborate.

## Supporting Information

Figure S1 **Differential proliferative response to Shh in tectal plated nsps versus explants.** Representative images **(A)** and quantification **(B)** of BrdU incorporation on plated nsps after treatments as indicated for 8 hours. **(C)** BrdU quantitative analysis of explants treated with Shh for 8 hours and pulsed with BrdU for the last 2 hours of treatment. Bar, 20 µm. *, p<0.05; **, p<0.01. W/O GF: without growth factors, E: EGF, F: FGF-2.
(TIF)

Figure S2 **Tectal nsps in collagen cultures are viable and respond to Shh stimulation.** NSC suspension cultures were immobilized in collagen type-I gels in presence of growth factors (EGF/FGF-2; 10 ng/ml). **(A)** Immunofluorescence analysis of Group B1 Sox, GLAST and Phalloidin revealed a high percentage of active proliferating NSC. Bar, 50 µm. **(B)** Collagen culture examination with SEM reveals nsps immersed into the collagen matrix. Detail of a nsp growing out of the gel, interacting nsps are indicated by double arrow. A clear adhesive interaction of nsps and the gel is shown; arrows denote porous texture of the collagen scaffold. Bar, 20 µm. (**C**) Viability was assayed by cleaved caspase-3 labeling. Quantification of the percentage of cells undergoing apoptosis was not significantly different when Cyc (10 µM) or Shh (3.3 µg/ml) were incubated for 48 hours in presence/absence of growth factors. Accompanied are representative images of chosen nsps for cell counts. Bar, 10 µm. **(D)** H2A.X marker show low DNA damage even after Cyc treatment. Bar, 20 µm. W/O GF: without growth factors, E: EGF, F: FGF-2.
(AI) Click here for additional data file.

Figure S3 **Shh regulates EGF-R induced symmetric cell divisions in NSCs. (A)** Effect of Cyc and Shh after 24 hours treatments on plated nsps without any other growth factors. Histogram shows significant increase in the relative percentage of EGF-R asymmetric divisions at the expense of EGF-R symmetric divisions in Cyc (10 µM), and the opposite is seen upon Shh (3.3 µg/ml) treatment. Total number of pairs per coverslip was scored. **(B)** Representative immunofluorescence of EGF-R in two sister pairs. The two modes of divisions, either symmetric or asymmetric EGF-R segregation, are illustrated. Co-labeling experiments revealed that EGF-R distribution in sibling cells always correlates with that of PKCλ, used as a control. Bar, 10 µm. *p<0.05.
(TIF)
