# Correction: Proliferation of Murine Midbrain Neural Stem Cells Depends upon an Endogenous Sonic Hedgehog (Shh) Source

**DOI:** 10.1371/journal.pone.0239995

**Published:** 2020-09-24

**Authors:** Constanza Martínez, Víctor Hugo Cornejo, Pablo Lois, Tammy Ellis, Natalia P. Solis, Brandon J. Wainwright, Verónica Palma

After publication of this article [1], the authors notified the journal of errors in Figs 4 and S2, which depict the results of western blot and microscopy experiments, respectively.

**Fig 4:**
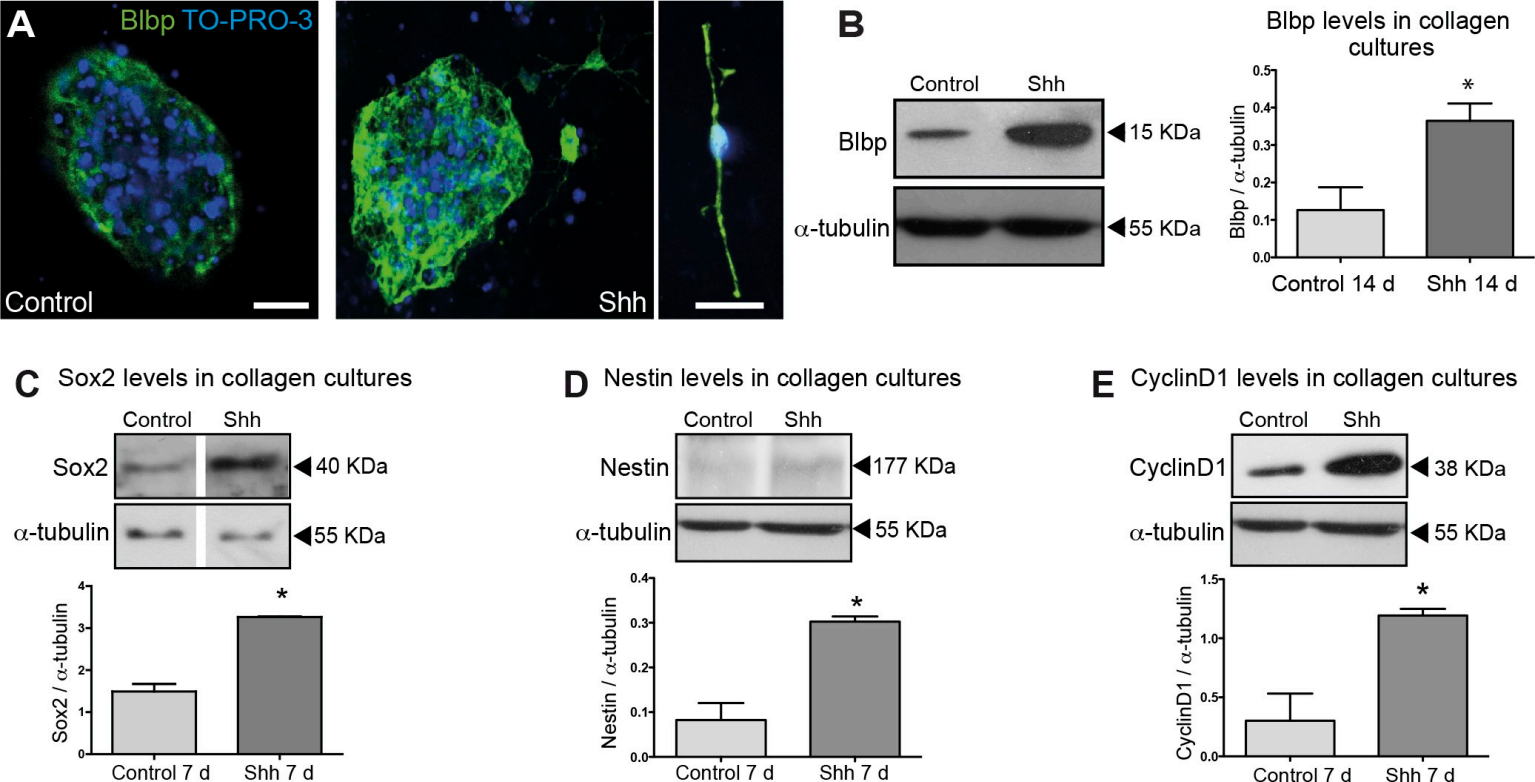
Shh promotes proliferation and maintenance of RGCs. Differentiation experiments were performed during 14 days without addition of EGF or FGF-2. (A) Representative images of immunostaining for Blbp after control or Shh treatment. Close-up view of a RGC stained for Blbp treated with Shh. Bar, 20 μm. (B) Western blot and densitometry analysis for Blbp expression show higher levels in Shh treated cultures. Western Blot analysis of Sox2 (C), and Nestin (D) levels after 7 days of treatment with Shh indicate an increase of neural progenitors. (E) Western blot of Cyclin D1, a read-out response to Shh pathway activation, indicates an increased proliferation even in absence of other additional growth factors after 7 days of Shh incubation. *, p<0.05. In Fig 4C, lane 1 and 2 of each panel present non-adjacent lanes of the same original blot; additional lanes were removed from the image in preparing the figure. The original image data supporting Fig 4C are in S2 File, and data supporting Fig 4B, D, E are in S1 and S3 Files.

Specifically:

- The same data were reported in Fig 4B and 4D α-tubulin panels, and lane 2 in α-tubulin panels of Fig 4B and 4D reports the same data as lane 1 in the α-tubulin panel of Fig 4E, although with different aspect ratio. The authors noted that they inadvertently included incorrect α-tubulin loading controls when preparing Fig 4B and 4E, and that these errors also impacted the quantification of band intensity in the accompanying graphs.
- When preparing each panel of Fig 4C, the authors spliced out irrelevant lanes from the blot images. Consequently, the panels show non-adjacent lanes of the indicated blots and there are vertical discontinuities between lanes 1 and 2. For each figure panel, the reported data were obtained from the same blot imaged at the same exposure. The authors provide with this notice an updated figure legend that explains these aspects of the figure’s preparation.
- In S2C Fig, the W/O GF image was mistakenly shown in the E+F10 + Cyc panel, and the W/O GF and E+F1+Shh panels reported overlapping fields of the same (E+F1+Shh) data. The authors noted that these issues resulted from errors in figure preparation.

To address these errors, the authors provide here:

1. A revised version of Fig 4 with the correct loading controls and with re-quantified band intensities in panels B, D, and E. Raw blot images underlying the revised figure are provided in S1 and S2 Files, and quantification data are in S3 and S4 Files. Note that the same tubulin data apply to Fig 4D and 4E as the same samples and blot were used for these experiments. To generate the updated quantitative data, the original blot results were re-scanned; the images used for quantitative analyses are in S5 File.
2. A revised version of S2 Fig in which the E+F1+Shh and E+F10 + Cyc panels have been replaced with the correct representative microscopy images from the original experiments. The full set of unprocessed microscopy images obtained in this experiment and the quantification data used to generate the graph in Fig S2C are in S6 and S7 Files, respectively.

The authors also provide the following clarifications regarding replication and the study design for experiments reported in Fig 4:

Primary tectal neurosphere cultures were prepared from 8–12 embryos (littermates) and after the first passage we seeded embryonic dorsal murine mesencephalon NSC into collagen type-I gels to establish 3D cultures (typically 20+). For Fig 4 data presented in the paper, twelve healthy 3D cultures in total were chosen for the experiment (six control—no growth factors vs. six experimental–no growth factors + Shh). For all western blot experiments three tectal neurosphere 3D cultures per lane were used, both in the control or experimental condition. Two lanes were loaded per condition, thereby comparing the pooled average of N = 3 cultures total for each condition (control vs. experimental), in duplicate (12 cultures total). Samples for each experiment were run as a single gel and re-stained sequentially for multiple antibodies. Membranes were cut to facilitate the multiple antibody detection; different types of film were used in detection of α-tubulin and Cyclin D1 (Agfa films) and Nestin, BLBP, Sox2, Neurofilament (Amersham Hyperfilm ECL film). The authors chose the most representative band lane for the image, and two bands per blot were included in the densitometric analyses. Densitometric results were analyzed using student unpaired t tests, with the GraphPad Prism software.

A *PLOS ONE* Editorial Board member reviewed the updated figures and underlying data. They confirmed that the updated materials addressed the errors in the published figures and overall provide support for the results and conclusions reported in the original article. For the Nestin experiment reported in Fig 4D, the raw data suggest elevated levels of a ~170 kD protein in Shh treated cultures at 7 days of treatment that corresponds with the expected size of Nestin protein. However, comparison of control versus Shh data at the 14 day timepoint do not indicate upregulation of this band, and the Nestin blot also includes bands for a number of additional proteins that migrated above and below the 170kD marker. Whether these additional proteins are Nestin variants (e.g. with post-translational modifications) or other background proteins detected by the antibody was not addressed.

The authors apologize for the errors in the published article. Underlying data for other results reported in this article and its Supporting Information files are available upon request from the corresponding author, except for the data underlying Figs 1, 2 and 5.

The *PLOS ONE* Editors apologize for our delay in correcting the article after the authors notified us in 2015 of the errors and requested an erratum.

## Supporting information

S2 Fig Tectal nsps in collagen cultures are viable and respond to Shh stimulation. NSC suspension cultures were immobilized in collagen type-I gels in presence of growth factors (EGF/FGF-2; 10 ng/ml). (A) Immunofluorescence analysis of Group B1 Sox, GLAST and Phalloidin revealed a high percentage of active proliferating NSC. Bar, 50 μm. (B) Collagen culture examination with SEM reveals nsps immersed into the collagen matrix. Detail of a nsp growing out of the gel, interacting nsps are indicated by double arrow. A clear adhesive interaction of nsps and the gel is shown; arrows denote porous texture of the collagen scaffold. Bar, 20 μm. (C) Viability was assayed by cleaved caspase-3 labeling. Quantification of the percentage of cells undergoing apoptosis was not significantly different when Cyc (10 μM) or Shh (3.3 μg/ml) were incubated for 48 hours in presence/absence of growth factors. Accompanied are representative images of chosen nsps for cell counts. Bar, 10 μm. (D) H2A.X marker show low DNA damage even after Cyc treatment. Bar, 20 μm. W/O GF: without growth factors, E: EGF, F: FGF-2
(TIF)

S1 File Raw western blot data underlying Fig 4B, 1D and 1E. The membrane was cut at 100 kDa and 25 kDa to facilitate detection of different proteins. Lanes 6, 7 were included in panel B; lanes 2, 3 of the indicated blots were included in panels D and E. The tubulin, Cyclin D1, BLBP, and Nestin data were generated by cutting the same blot into three sections and re-probing with sequential antibodies. I.e., these data represent results for the same neurosphere collagen culture extracts run on the same gel/blot. Abcam ab11306-25 was used for the anti-Nestin western blot.
(PDF)

S2 File Raw western blot data underlying Fig 4C. The membrane was cut, the 40–70 kDa fragment was used to detect a-tubulin and the 35–40 kDa fragment was used to detect Sox-2. Lanes 3 and 8 are shown in the figure.
(PDF)

S3 File Quantification data to support graphs in the updated version of Fig 4B, 4D and 4E. (XLSX)

S4 File Quantitative data supporting Fig 4C. (PZF)

S5 File New scans of the original data that were used to re-quantify western blot data for the updated Fig 4. (ZIP)

S6 File Raw microscopy images for caspase-3 detection experiment. Representative images in the revised S2C Fig were generated from raw files woGF_4_ch1/2 (W/O GF); EF10cyc_4_ch1/2 (E+F10+Cyc); EF1shh_7_ch1/2 (E+F1+Shh).
(ZIP)

S7 File Quantification data for caspase-3 detection experiment used to generate graph in S2 Fig. (PZF)
