# Supplementary figures and images for: Proliferation of Murine Midbrain Neural Stem Cells Depends upon an Endogenous Sonic Hedgehog (Shh) Source
Source: PLoS One. 2013 Jun 11;8(6):e65818. doi: 10.1371/journal.pone.0065818 (PMC3679138; doi:10.1371/journal.pone.0065818)

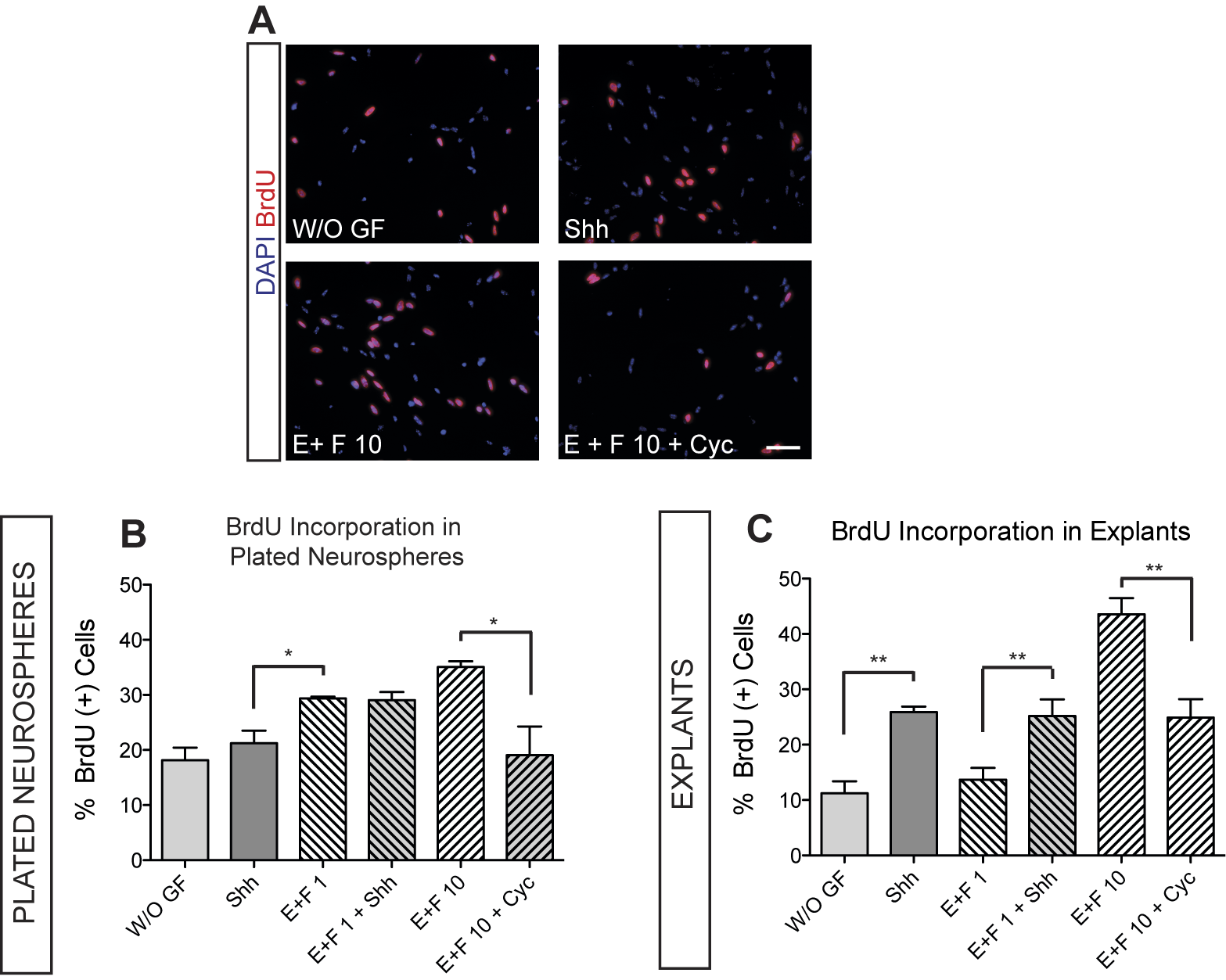

Supplement: Figure S1 — Differential proliferative response to Shh in tectal plated nsps versus explants. Representative images (A) and quantification (B) of BrdU incorporation on plated nsps after treatments as indicated for 8 hours. (C) BrdU quantitative analysis of explants treated with Shh for 8 hours and pulsed with BrdU for the last 2 hours of treatment. Bar, 20 µm. *, p<0.05; **, p<0.01. W/O GF: without growth factors, E: EGF, F: FGF-2. (TIF) [file pone.0065818.s001.tif]

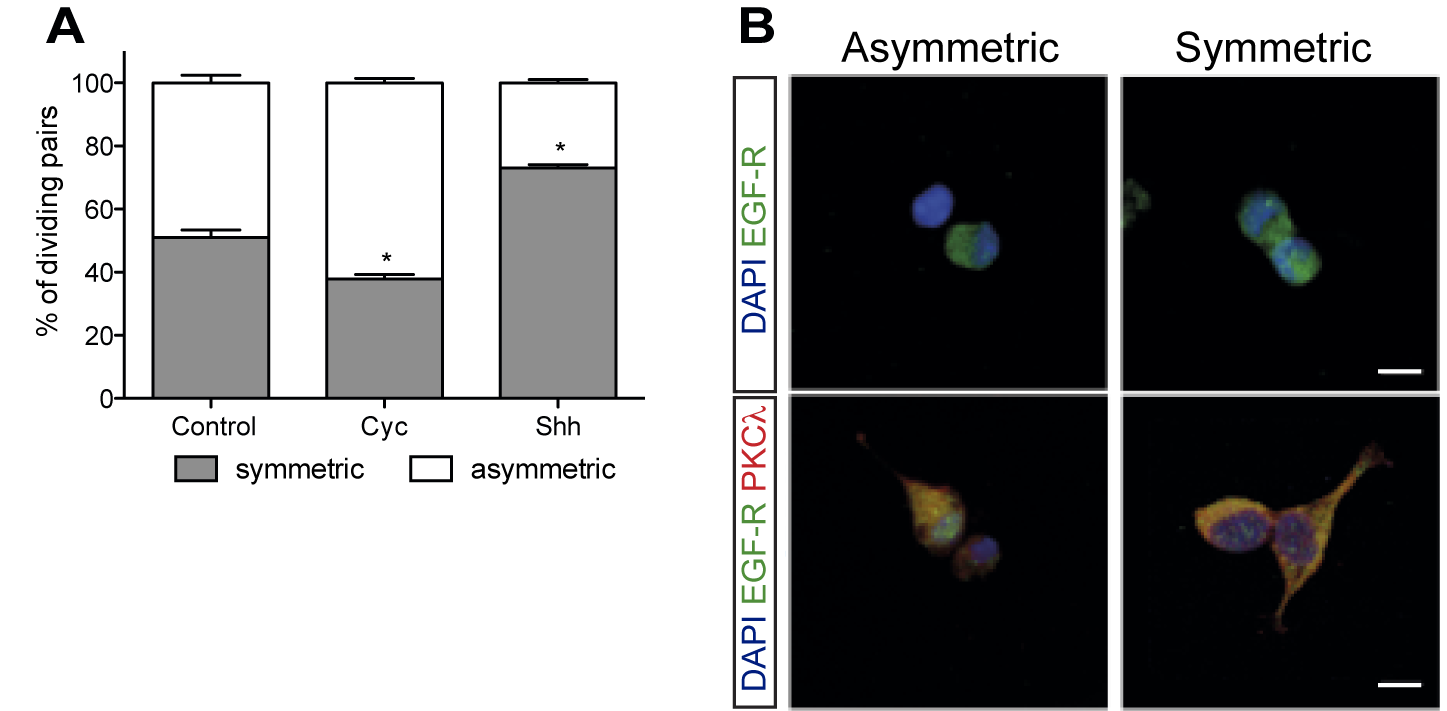

Supplement: Figure S3 — Shh regulates EGF-R induced symmetric cell divisions in NSCs. (A) Effect of Cyc and Shh after 24 hours treatments on plated nsps without any other growth factors. Histogram shows significant increase in the relative percentage of EGF-R asymmetric divisions at the expense of EGF-R symmetric divisions in Cyc (10 µM), and the opposite is seen upon Shh (3.3 µg/ml) treatment. Total number of pairs per coverslip was scored. (B) Representative immunofluorescence of EGF-R in two sister pairs. The two modes of divisions, either symmetric or asymmetric EGF-R segregation, are illustrated. Co-labeling experiments revealed that EGF-R distribution in sibling cells always correlates with that of PKCλ, used as a control. Bar, 10 µm. *p<0.05. (TIF) [file pone.0065818.s003.tif]
