# Supplementary figures and images for: Correction: Proliferation of Murine Midbrain Neural Stem Cells Depends upon an Endogenous Sonic Hedgehog (Shh) Source (part 1 of 2)
Source: PLoS One. 2020 Sep 24;15(9):e0239995. doi: 10.1371/journal.pone.0239995 (PMC7514037; doi:10.1371/journal.pone.0239995)

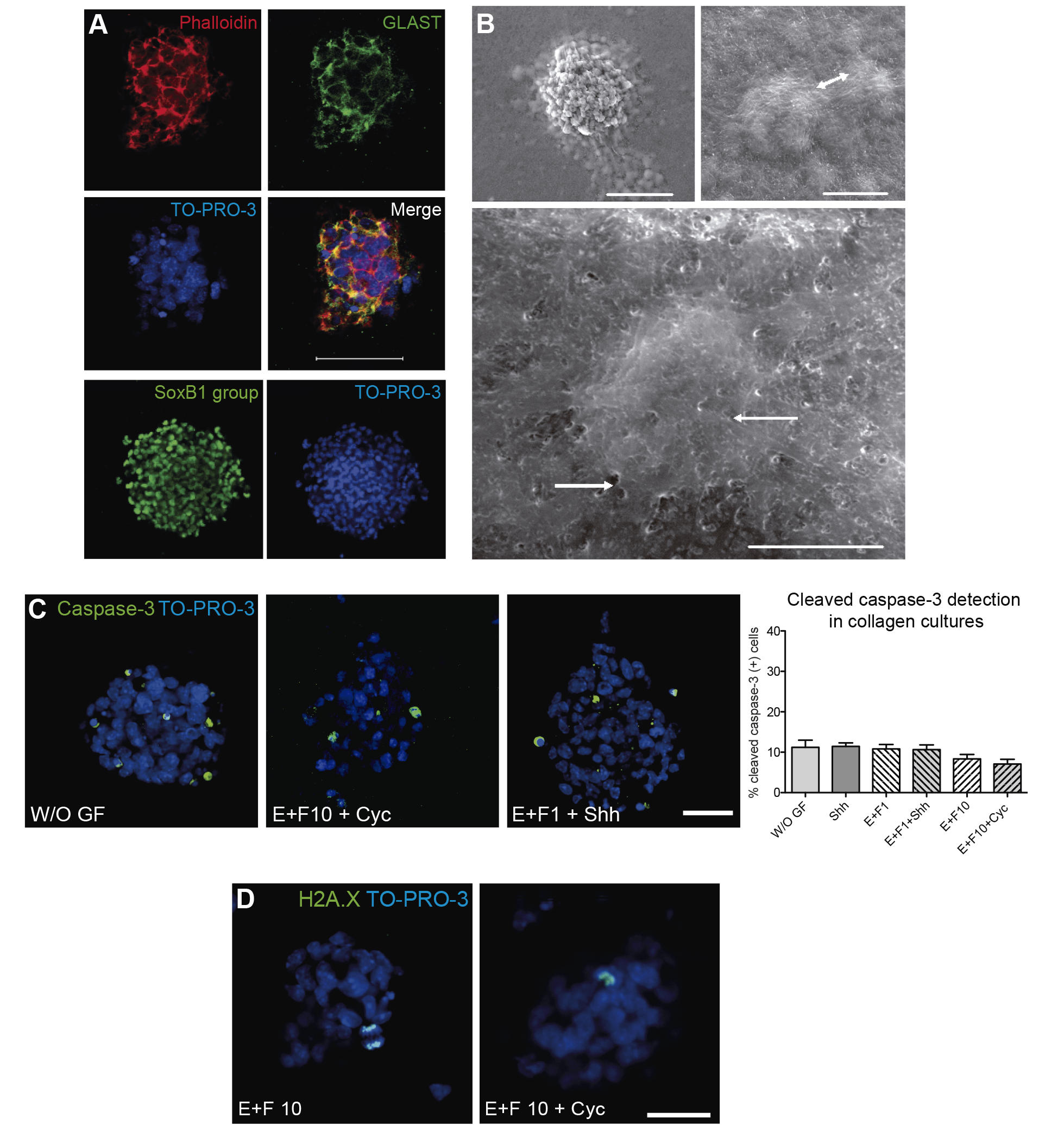

Supplement: S2 Fig — NSC suspension cultures were immobilized in collagen type-I gels in presence of growth factors (EGF/FGF-2; 10 ng/ml). (A) Immunofluorescence analysis of Group B1 Sox, GLAST and Phalloidin revealed a high percentage of active proliferating NSC. Bar, 50 μm. (B) Collagen culture examination with SEM reveals nsps immersed into the collagen matrix. Detail of a nsp growing out of the gel, interacting nsps are indicated by double arrow. A clear adhesive interaction of nsps and the gel is shown; arrows denote porous texture of the collagen scaffold. Bar, 20 μm. (C) Viability was assayed by cleaved caspase-3 labeling. Quantification of the percentage of cells undergoing apoptosis was not significantly different when Cyc (10 μM) or Shh (3.3 μg/ml) were incubated for 48 hours in presence/absence of growth factors. Accompanied are representative images of chosen nsps for cell counts. Bar, 10 μm. (D) H2A.X marker show low DNA damage even after Cyc treatment. Bar, 20 μm. W/O GF: without growth factors, E: EGF, F: FGF-2 (TIF) [file pone.0239995.s001.tif]

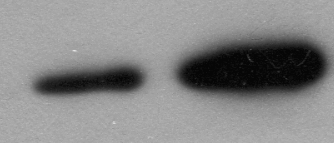

Supplement: S5 File — (ZIP) [file pone.0239995.s006.zip › ciclina D1 para figura dic 2012.tif]

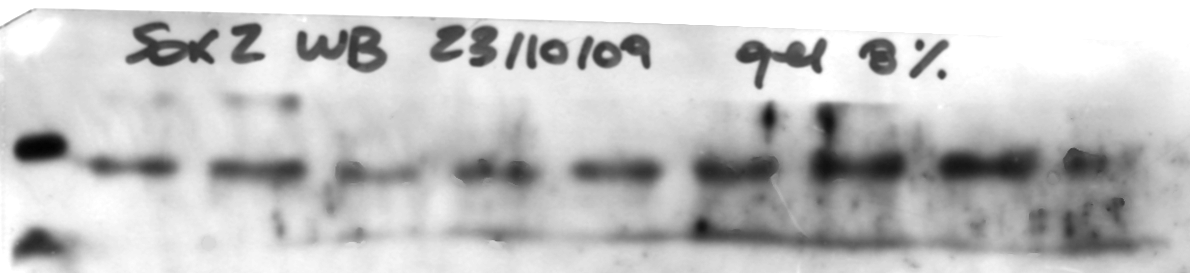

Supplement: S5 File — (ZIP) [file pone.0239995.s006.zip › SOX 2 densitometria.tif]

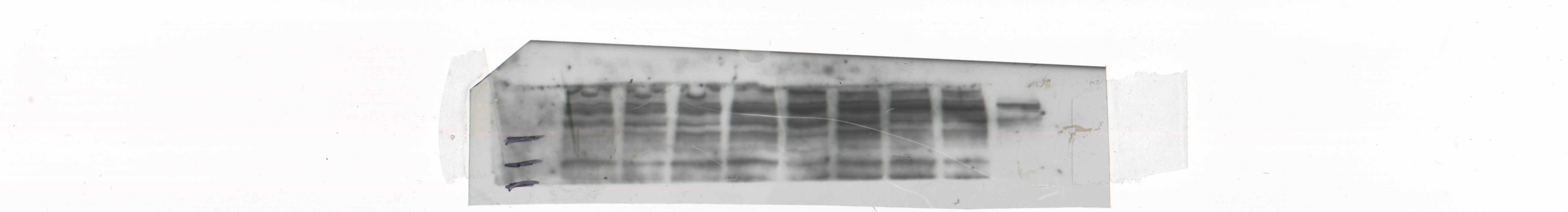

Supplement: S5 File — (ZIP) [file pone.0239995.s006.zip › third gel Nestin 600 dpi005.tif]

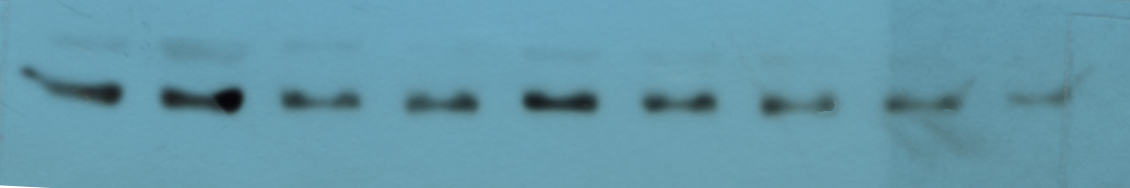

Supplement: S5 File — (ZIP) [file pone.0239995.s006.zip › TUBULINA DE SOX2.tif]

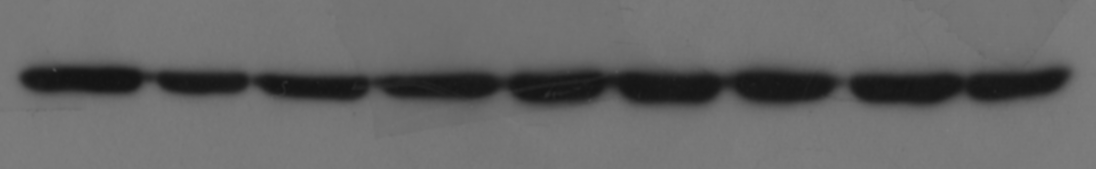

Supplement: S5 File — (ZIP) [file pone.0239995.s006.zip › tubulina para densitometria DE BLBP JUNIO 4 VERO.tif]

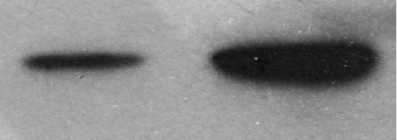

Supplement: S5 File — (ZIP) [file pone.0239995.s006.zip › wb blbp.tif]

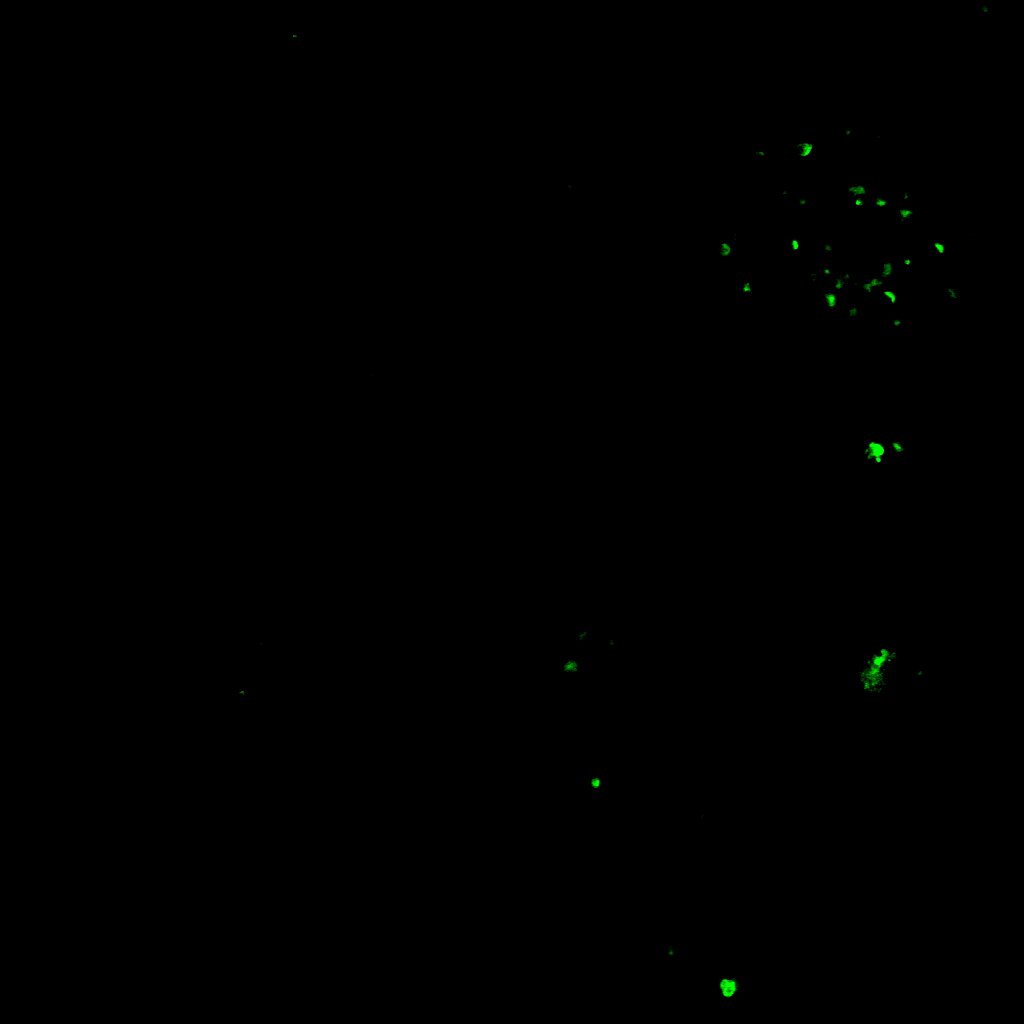

Supplement: S6 File — Representative images in the revised S2C Fig were generated from raw files woGF_4_ch1/2 (W/O GF); EF10cyc_4_ch1/2 (E+F10+Cyc); EF1shh_7_ch1/2 (E+F1+Shh). (ZIP) [file pone.0239995.s007.zip › S6_File/EF1_1_ch1.jpg]

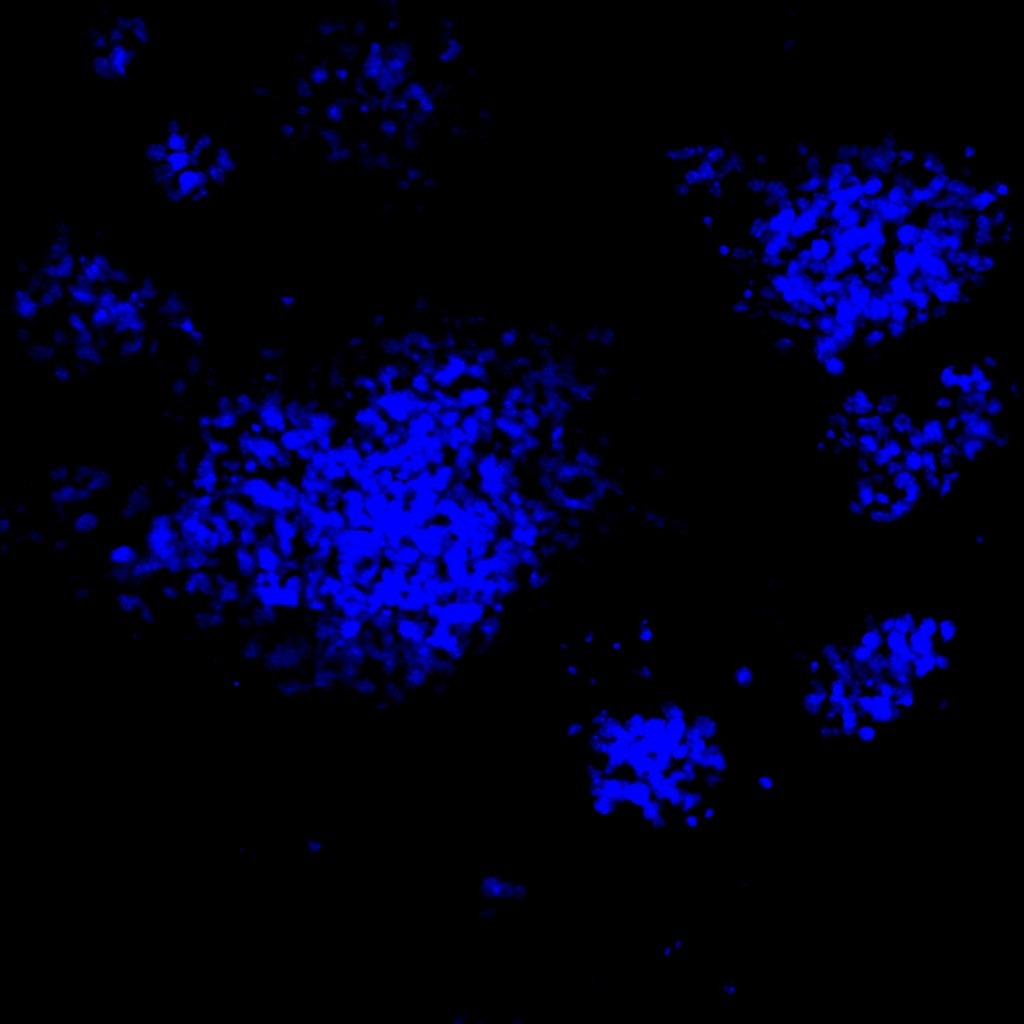

Supplement: S6 File — Representative images in the revised S2C Fig were generated from raw files woGF_4_ch1/2 (W/O GF); EF10cyc_4_ch1/2 (E+F10+Cyc); EF1shh_7_ch1/2 (E+F1+Shh). (ZIP) [file pone.0239995.s007.zip › S6_File/EF1_1_ch2.jpg]

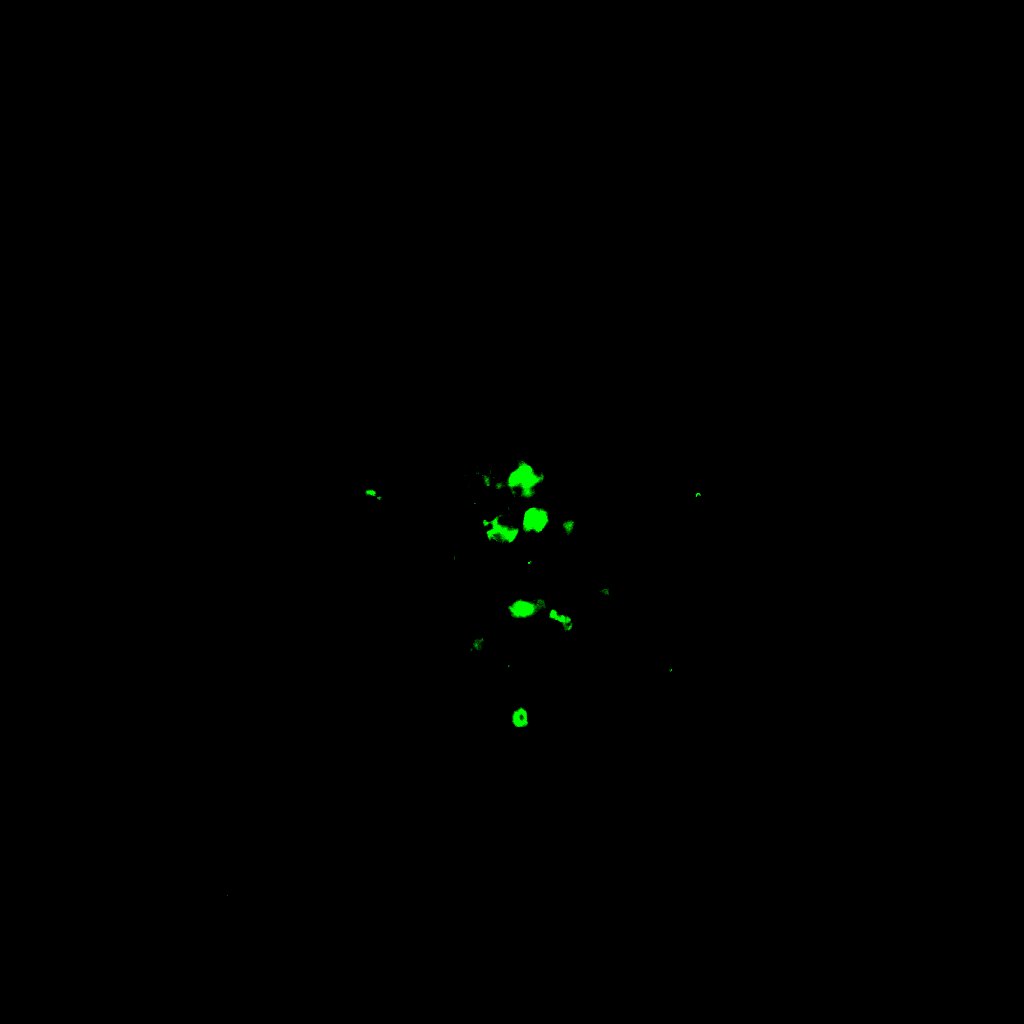

Supplement: S6 File — Representative images in the revised S2C Fig were generated from raw files woGF_4_ch1/2 (W/O GF); EF10cyc_4_ch1/2 (E+F10+Cyc); EF1shh_7_ch1/2 (E+F1+Shh). (ZIP) [file pone.0239995.s007.zip › S6_File/EF1_10_ch1.jpg]

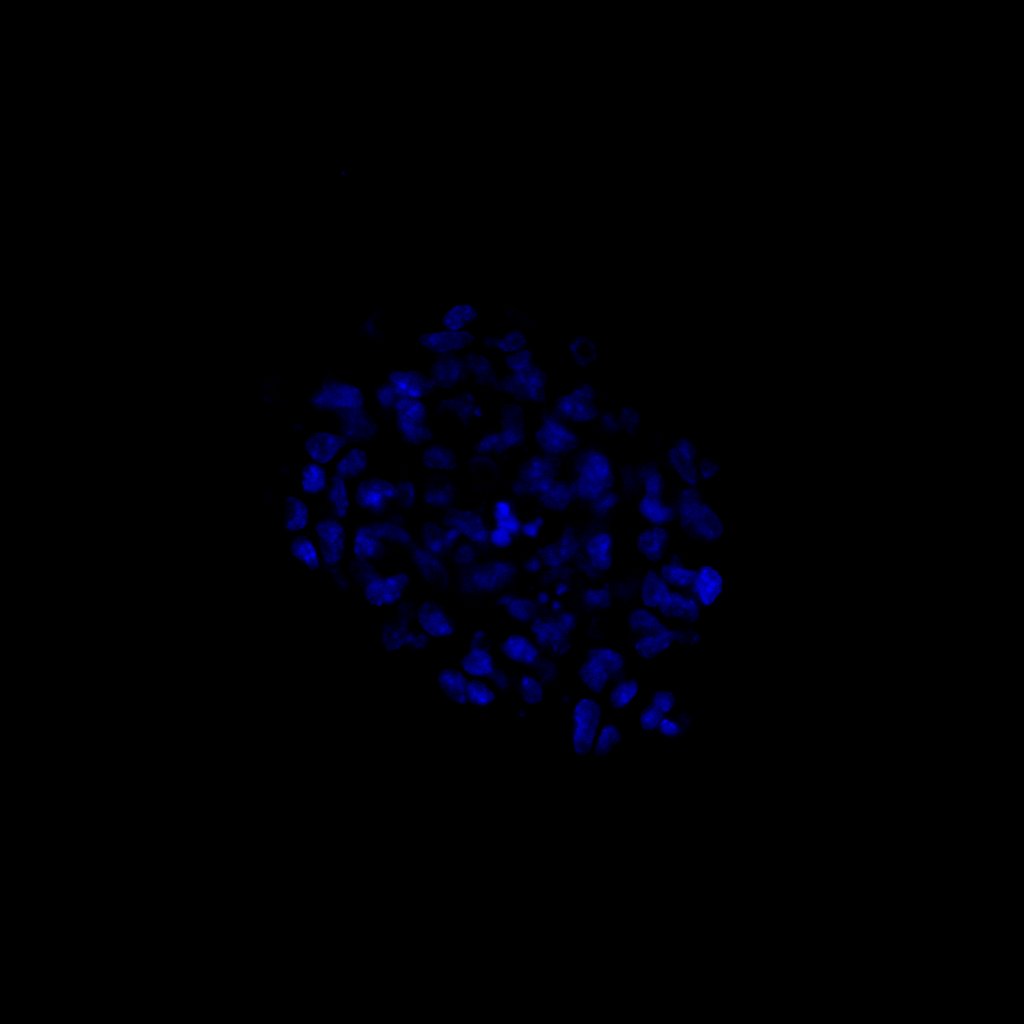

Supplement: S6 File — Representative images in the revised S2C Fig were generated from raw files woGF_4_ch1/2 (W/O GF); EF10cyc_4_ch1/2 (E+F10+Cyc); EF1shh_7_ch1/2 (E+F1+Shh). (ZIP) [file pone.0239995.s007.zip › S6_File/EF1_10_ch2.jpg]

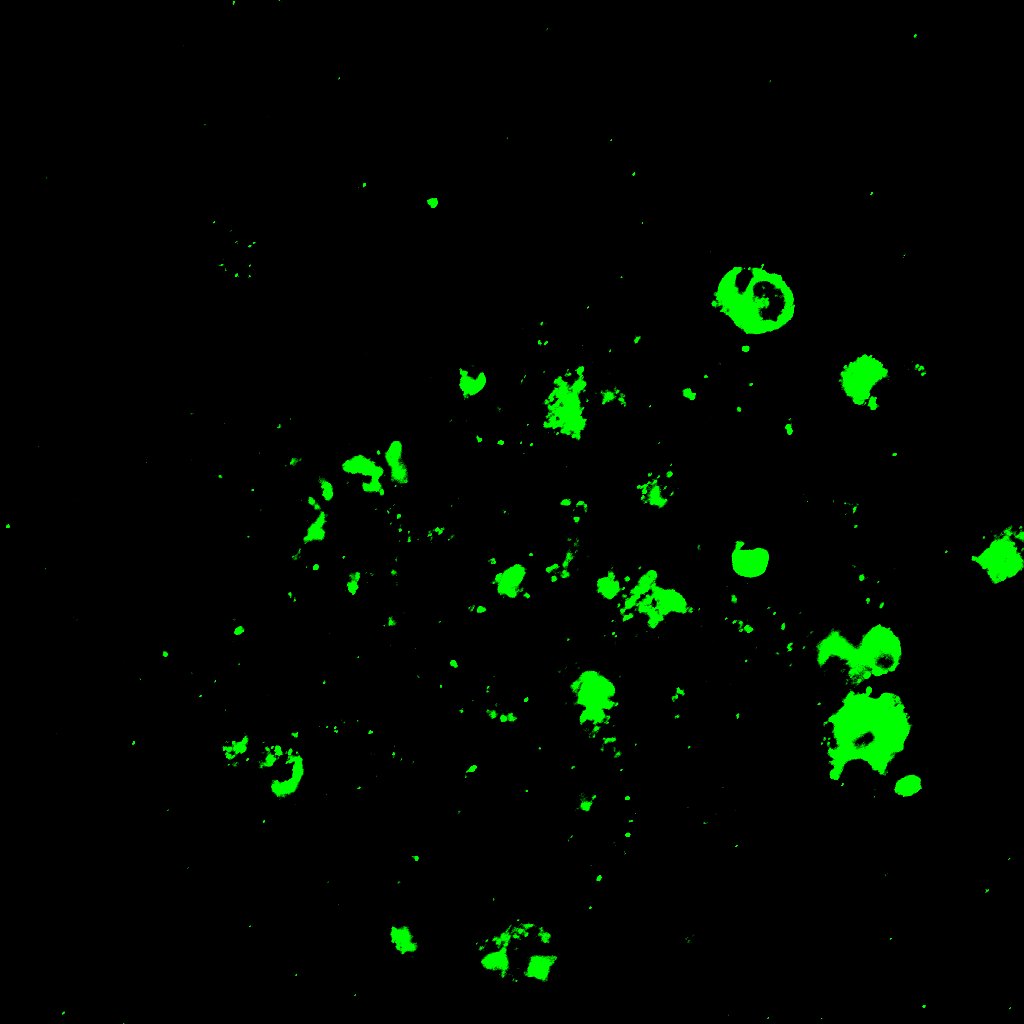

Supplement: S6 File — Representative images in the revised S2C Fig were generated from raw files woGF_4_ch1/2 (W/O GF); EF10cyc_4_ch1/2 (E+F10+Cyc); EF1shh_7_ch1/2 (E+F1+Shh). (ZIP) [file pone.0239995.s007.zip › S6_File/EF1_11_ch1.jpg]

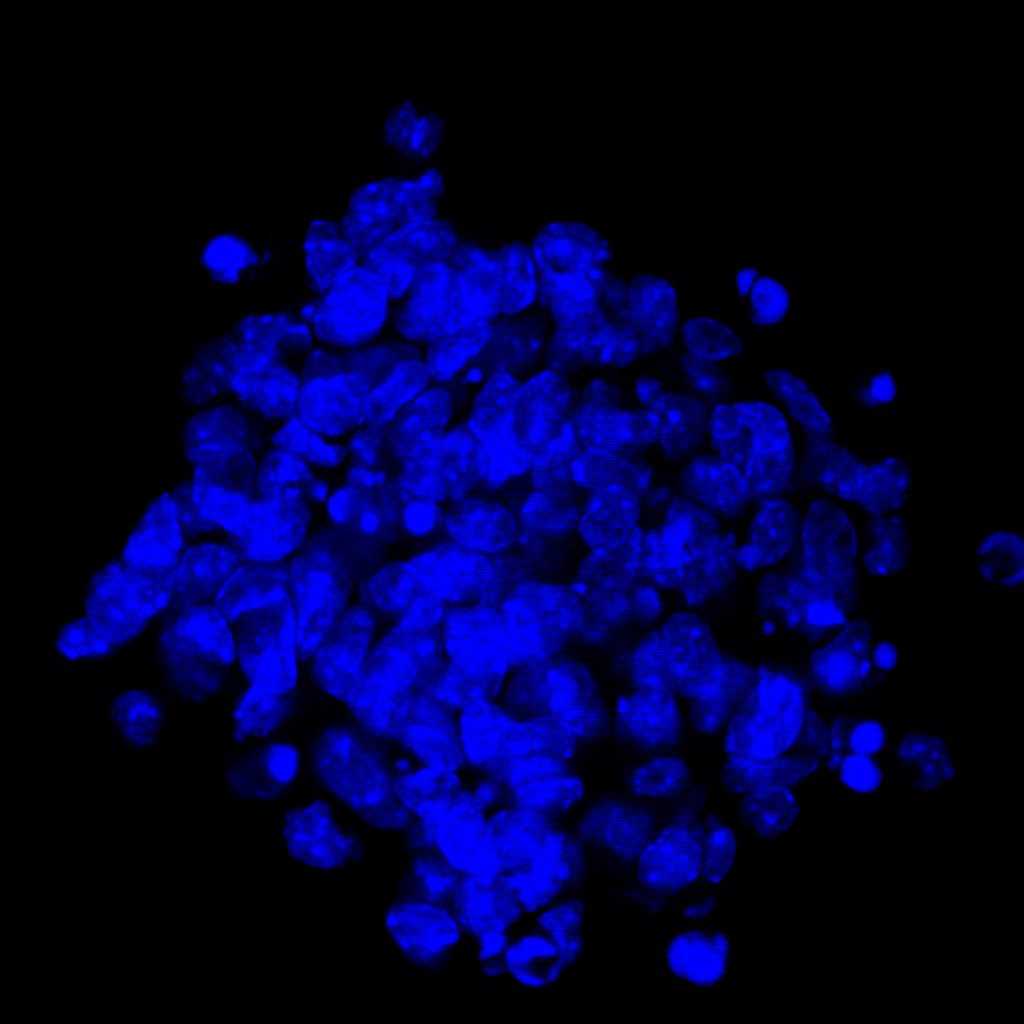

Supplement: S6 File — Representative images in the revised S2C Fig were generated from raw files woGF_4_ch1/2 (W/O GF); EF10cyc_4_ch1/2 (E+F10+Cyc); EF1shh_7_ch1/2 (E+F1+Shh). (ZIP) [file pone.0239995.s007.zip › S6_File/EF1_11_ch2.jpg]

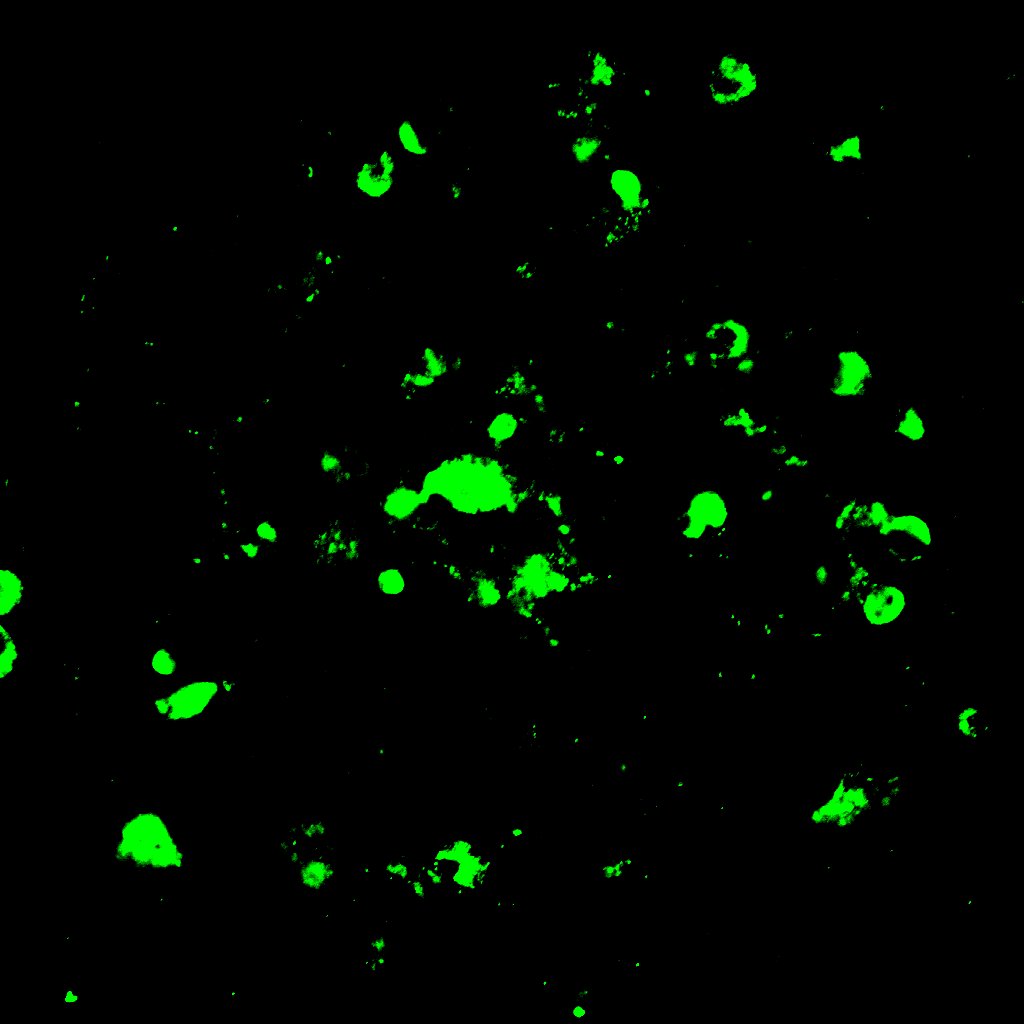

Supplement: S6 File — Representative images in the revised S2C Fig were generated from raw files woGF_4_ch1/2 (W/O GF); EF10cyc_4_ch1/2 (E+F10+Cyc); EF1shh_7_ch1/2 (E+F1+Shh). (ZIP) [file pone.0239995.s007.zip › S6_File/EF1_12_ch1.jpg]

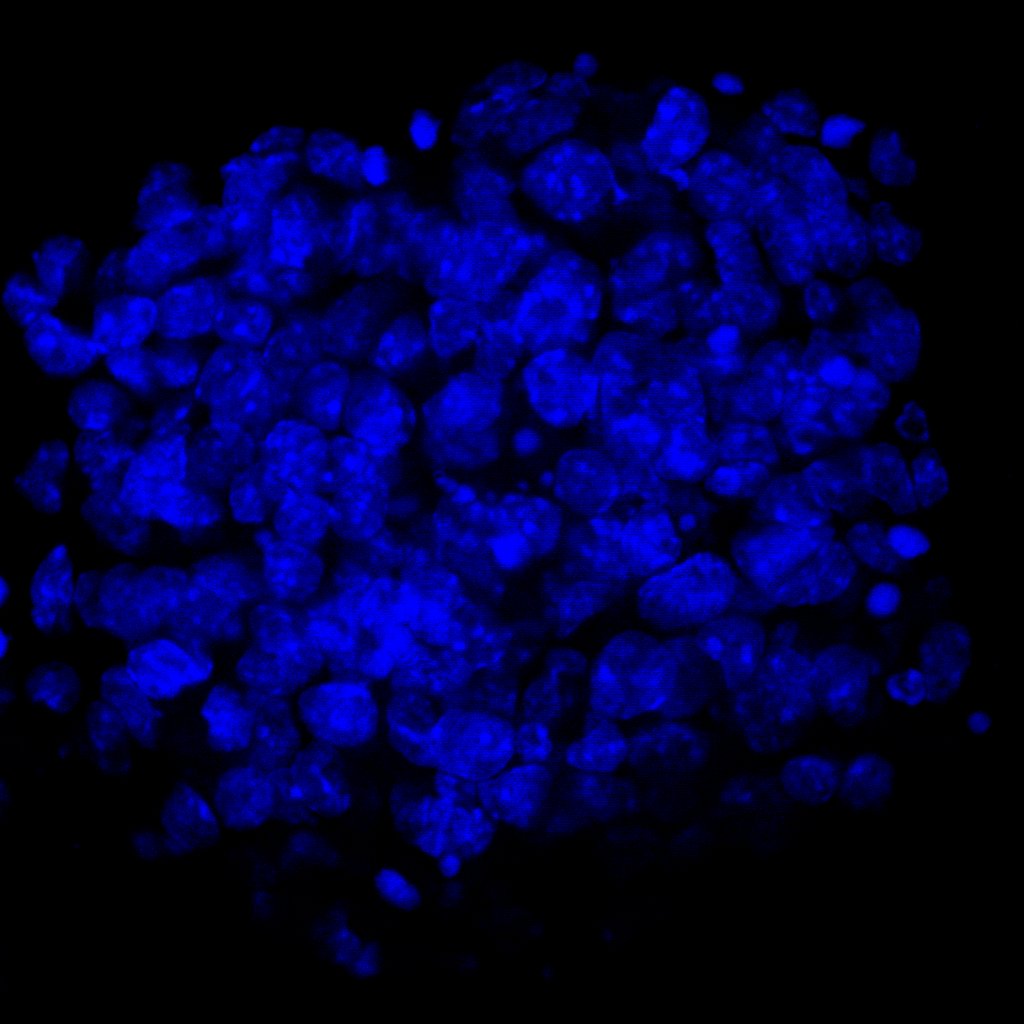

Supplement: S6 File — Representative images in the revised S2C Fig were generated from raw files woGF_4_ch1/2 (W/O GF); EF10cyc_4_ch1/2 (E+F10+Cyc); EF1shh_7_ch1/2 (E+F1+Shh). (ZIP) [file pone.0239995.s007.zip › S6_File/EF1_12_ch2.jpg]

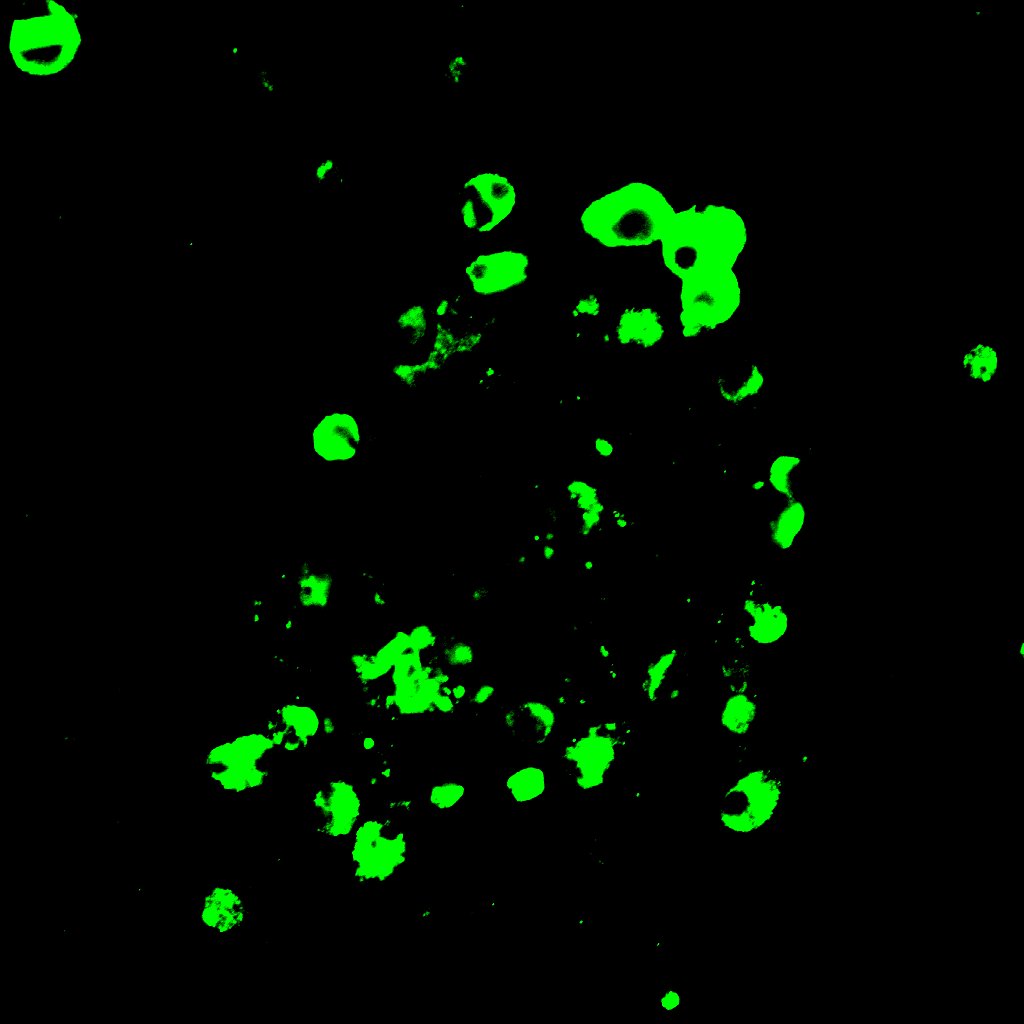

Supplement: S6 File — Representative images in the revised S2C Fig were generated from raw files woGF_4_ch1/2 (W/O GF); EF10cyc_4_ch1/2 (E+F10+Cyc); EF1shh_7_ch1/2 (E+F1+Shh). (ZIP) [file pone.0239995.s007.zip › S6_File/EF1_13_ch1.jpg]

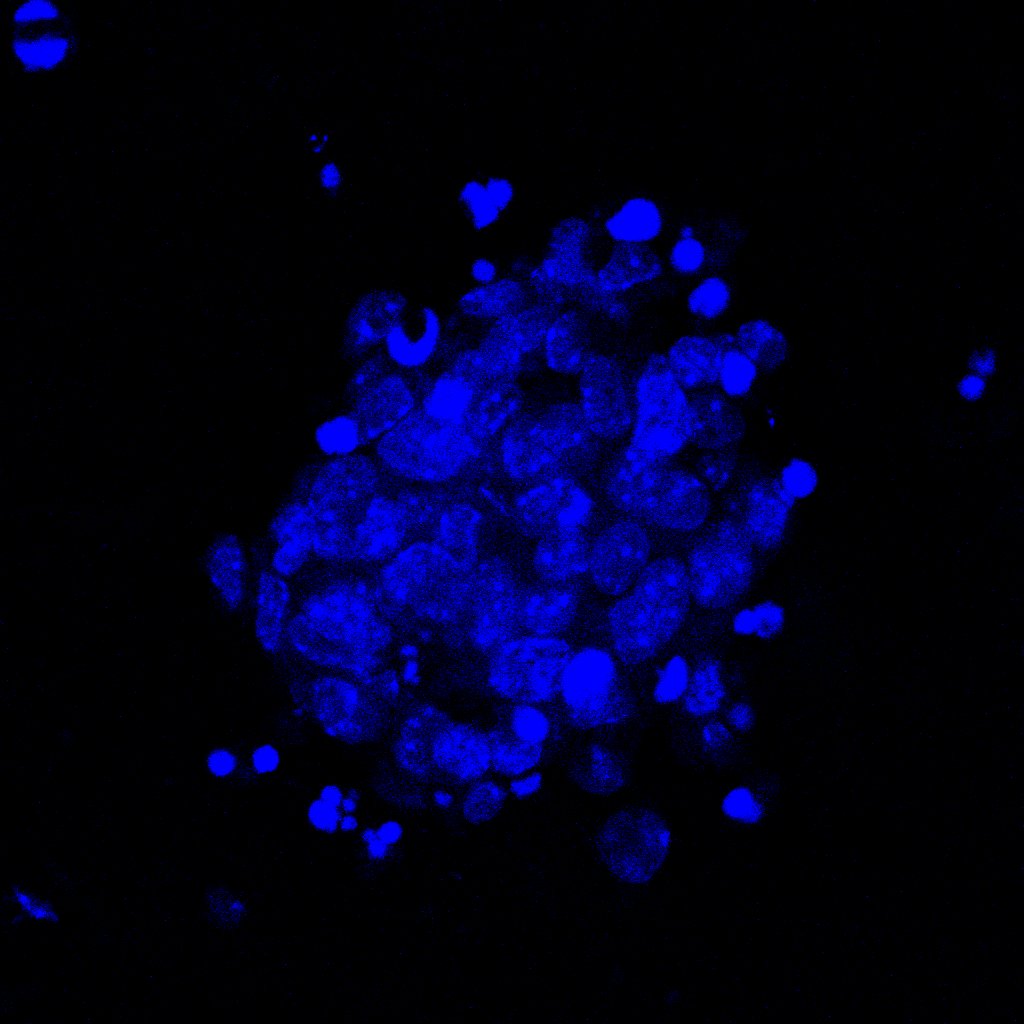

Supplement: S6 File — Representative images in the revised S2C Fig were generated from raw files woGF_4_ch1/2 (W/O GF); EF10cyc_4_ch1/2 (E+F10+Cyc); EF1shh_7_ch1/2 (E+F1+Shh). (ZIP) [file pone.0239995.s007.zip › S6_File/EF1_13_ch2.jpg]

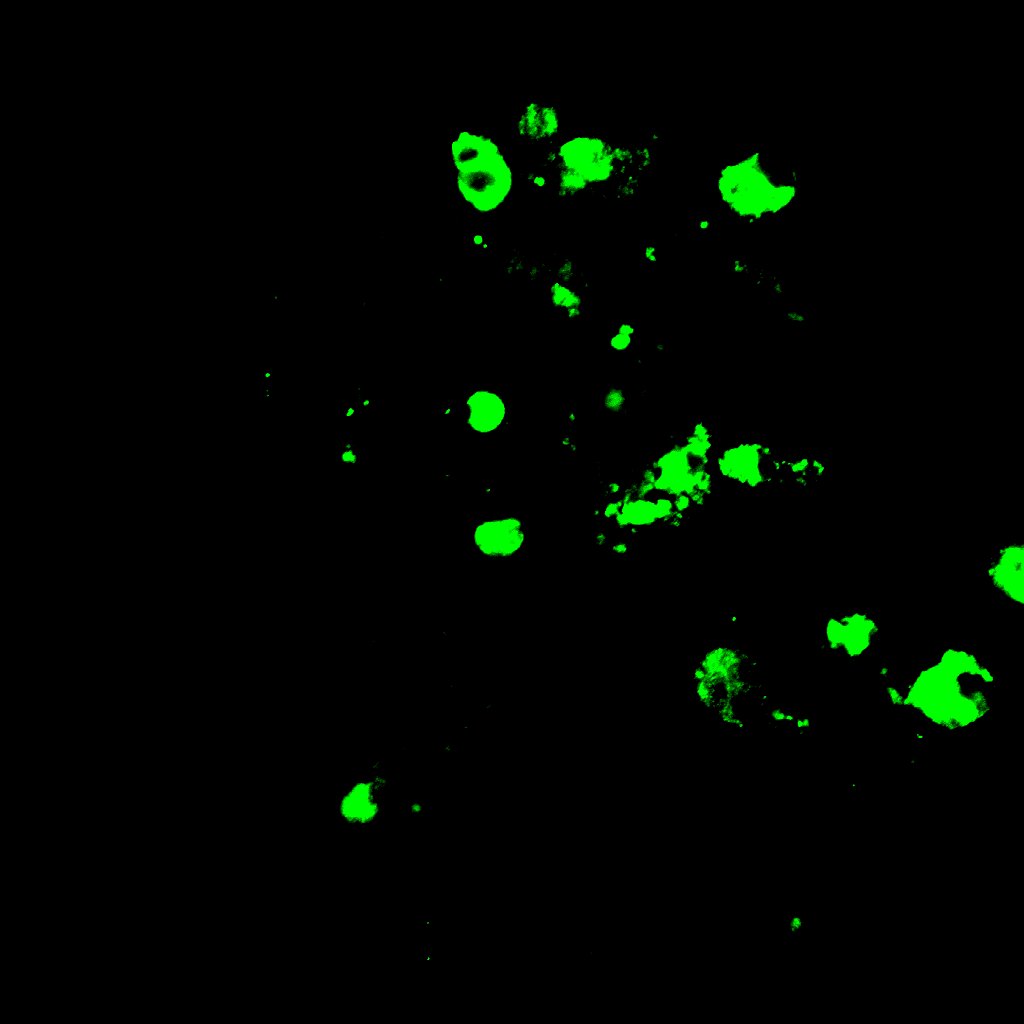

Supplement: S6 File — Representative images in the revised S2C Fig were generated from raw files woGF_4_ch1/2 (W/O GF); EF10cyc_4_ch1/2 (E+F10+Cyc); EF1shh_7_ch1/2 (E+F1+Shh). (ZIP) [file pone.0239995.s007.zip › S6_File/EF1_14_ch1.jpg]

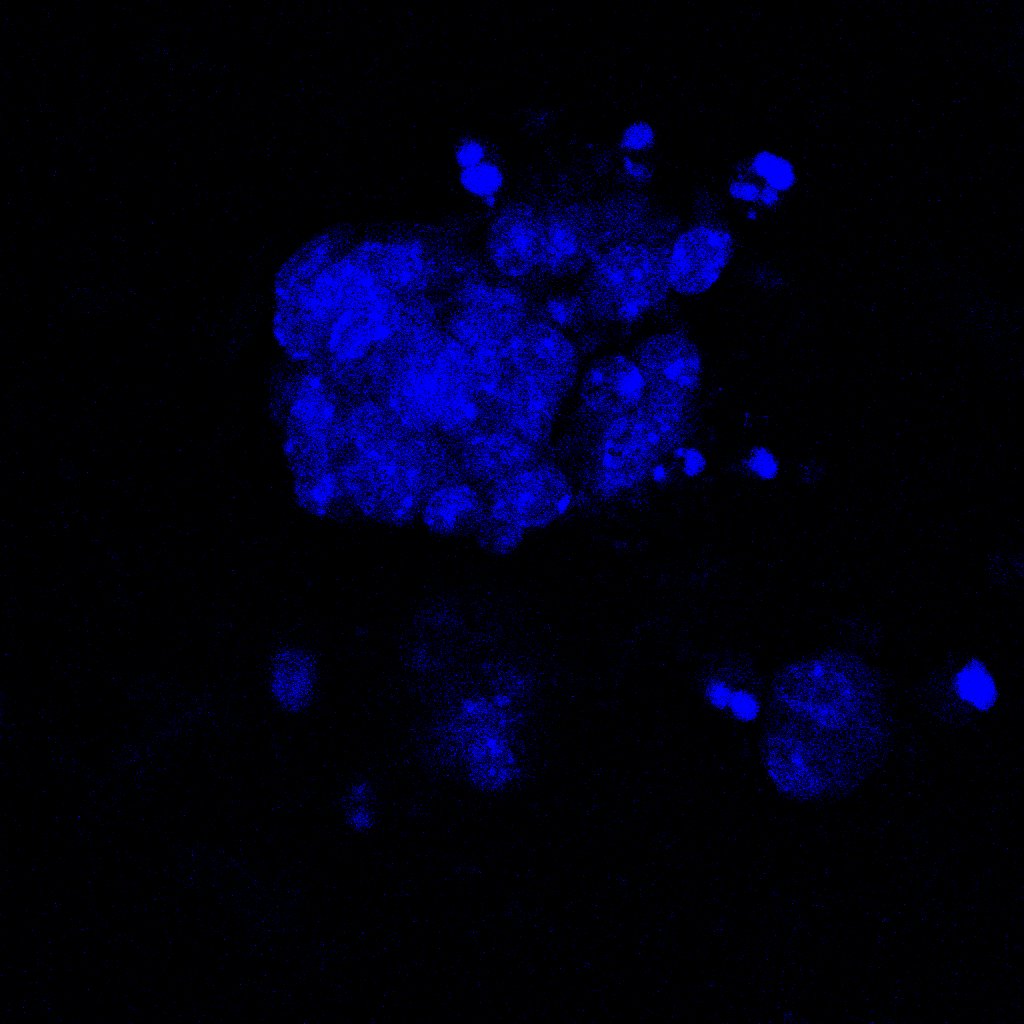

Supplement: S6 File — Representative images in the revised S2C Fig were generated from raw files woGF_4_ch1/2 (W/O GF); EF10cyc_4_ch1/2 (E+F10+Cyc); EF1shh_7_ch1/2 (E+F1+Shh). (ZIP) [file pone.0239995.s007.zip › S6_File/EF1_14_ch2.jpg]

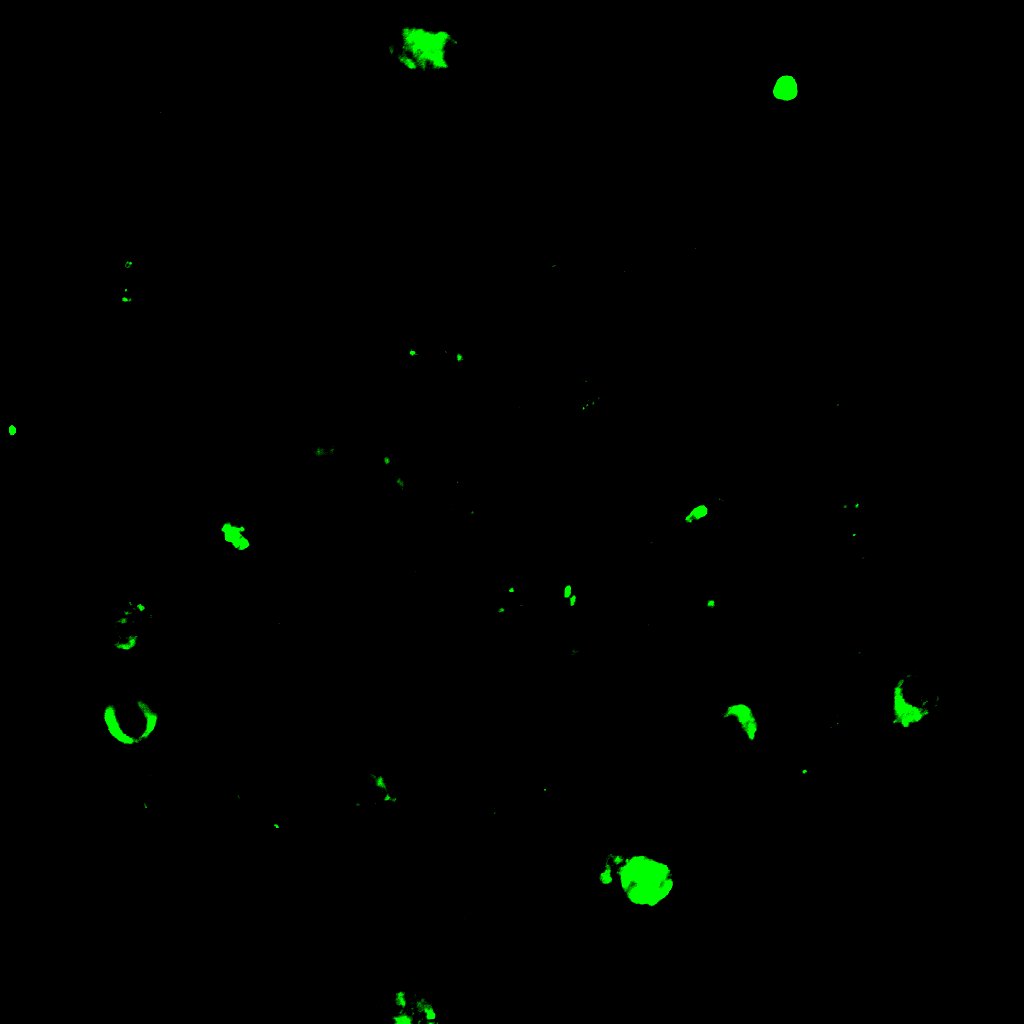

Supplement: S6 File — Representative images in the revised S2C Fig were generated from raw files woGF_4_ch1/2 (W/O GF); EF10cyc_4_ch1/2 (E+F10+Cyc); EF1shh_7_ch1/2 (E+F1+Shh). (ZIP) [file pone.0239995.s007.zip › S6_File/EF1_15_ch1.jpg]

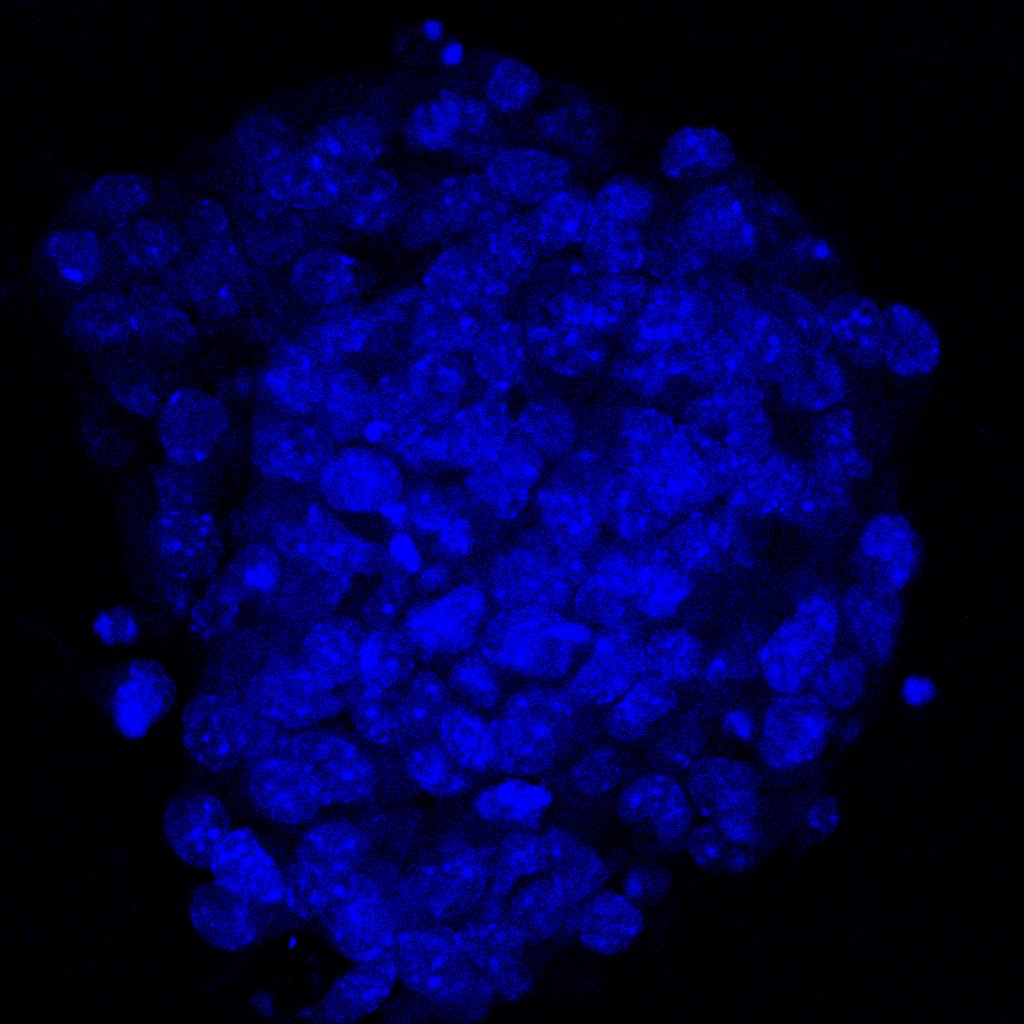

Supplement: S6 File — Representative images in the revised S2C Fig were generated from raw files woGF_4_ch1/2 (W/O GF); EF10cyc_4_ch1/2 (E+F10+Cyc); EF1shh_7_ch1/2 (E+F1+Shh). (ZIP) [file pone.0239995.s007.zip › S6_File/EF1_15_ch2.jpg]

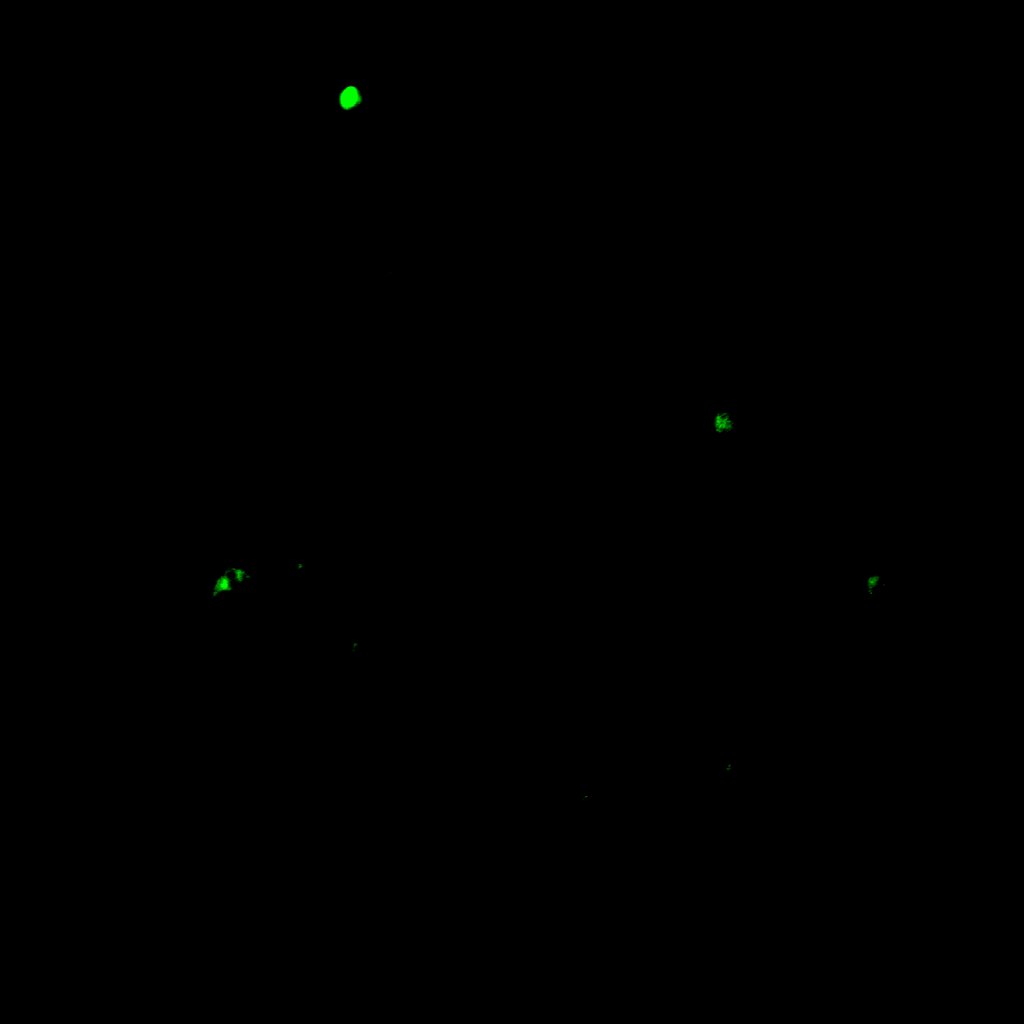

Supplement: S6 File — Representative images in the revised S2C Fig were generated from raw files woGF_4_ch1/2 (W/O GF); EF10cyc_4_ch1/2 (E+F10+Cyc); EF1shh_7_ch1/2 (E+F1+Shh). (ZIP) [file pone.0239995.s007.zip › S6_File/EF1_16_ch1.jpg]

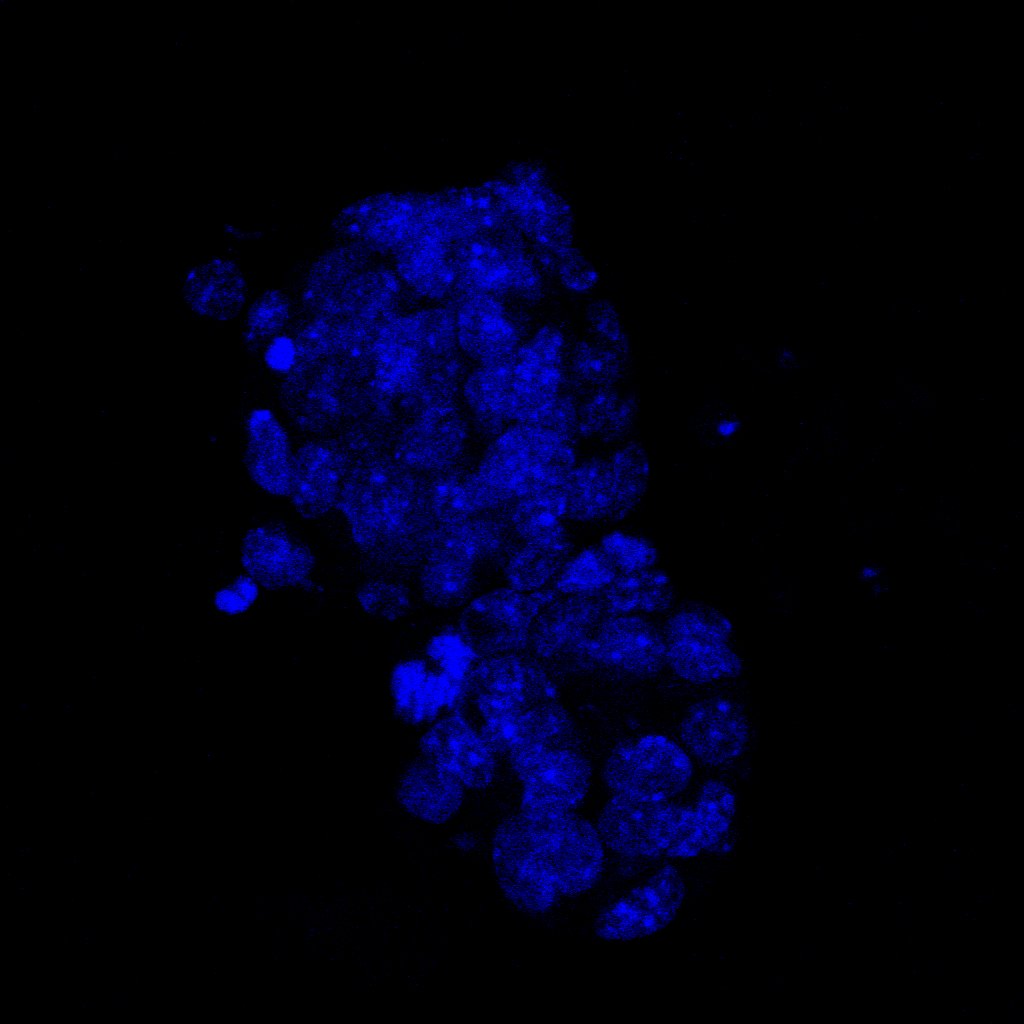

Supplement: S6 File — Representative images in the revised S2C Fig were generated from raw files woGF_4_ch1/2 (W/O GF); EF10cyc_4_ch1/2 (E+F10+Cyc); EF1shh_7_ch1/2 (E+F1+Shh). (ZIP) [file pone.0239995.s007.zip › S6_File/EF1_16_ch2.jpg]

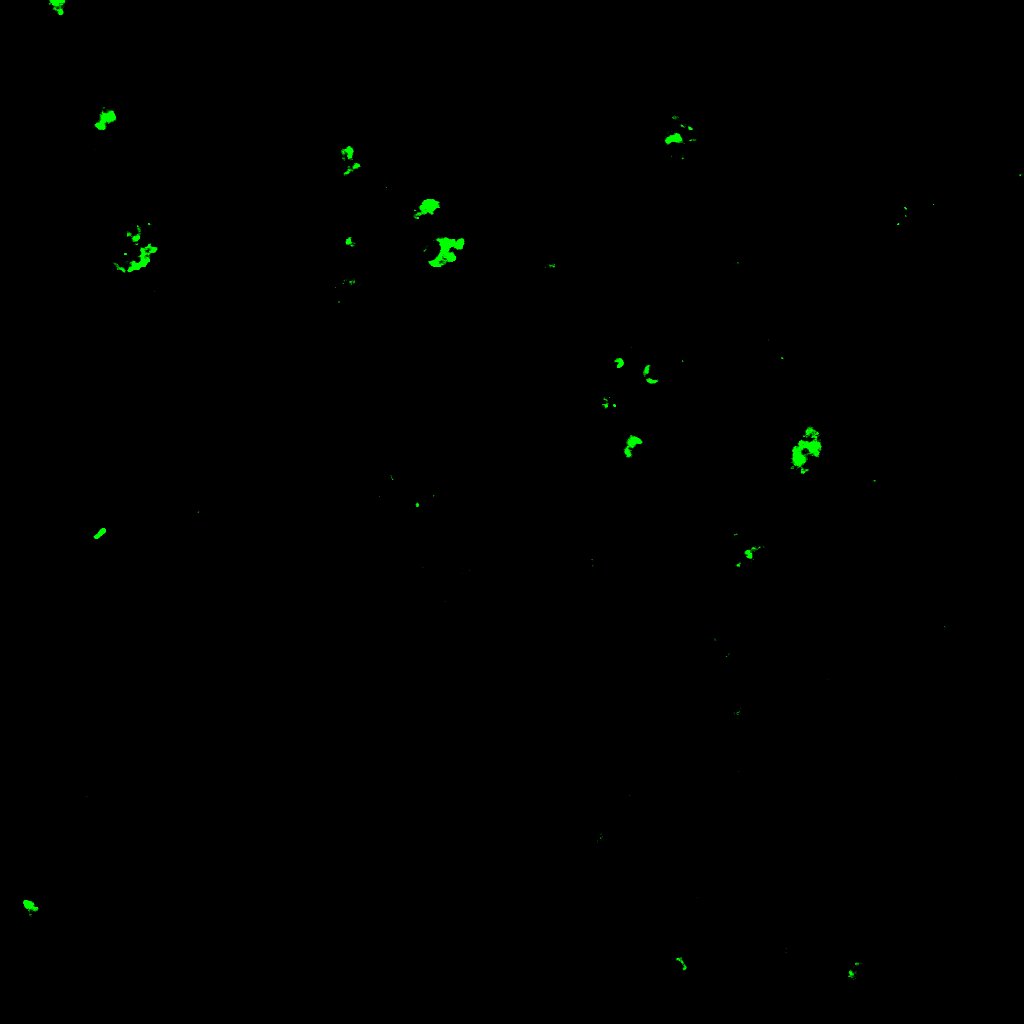

Supplement: S6 File — Representative images in the revised S2C Fig were generated from raw files woGF_4_ch1/2 (W/O GF); EF10cyc_4_ch1/2 (E+F10+Cyc); EF1shh_7_ch1/2 (E+F1+Shh). (ZIP) [file pone.0239995.s007.zip › S6_File/EF1_17_ch1.jpg]

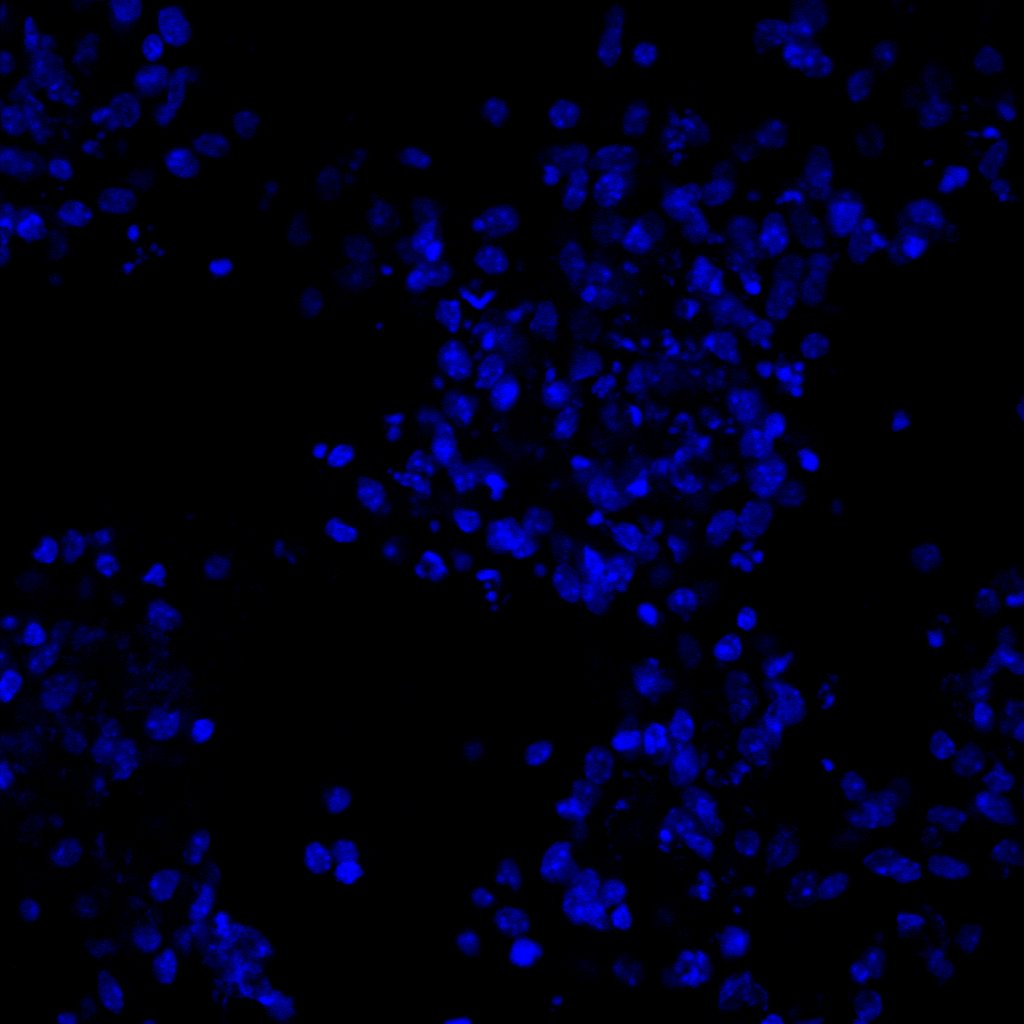

Supplement: S6 File — Representative images in the revised S2C Fig were generated from raw files woGF_4_ch1/2 (W/O GF); EF10cyc_4_ch1/2 (E+F10+Cyc); EF1shh_7_ch1/2 (E+F1+Shh). (ZIP) [file pone.0239995.s007.zip › S6_File/EF1_17_ch2.jpg]

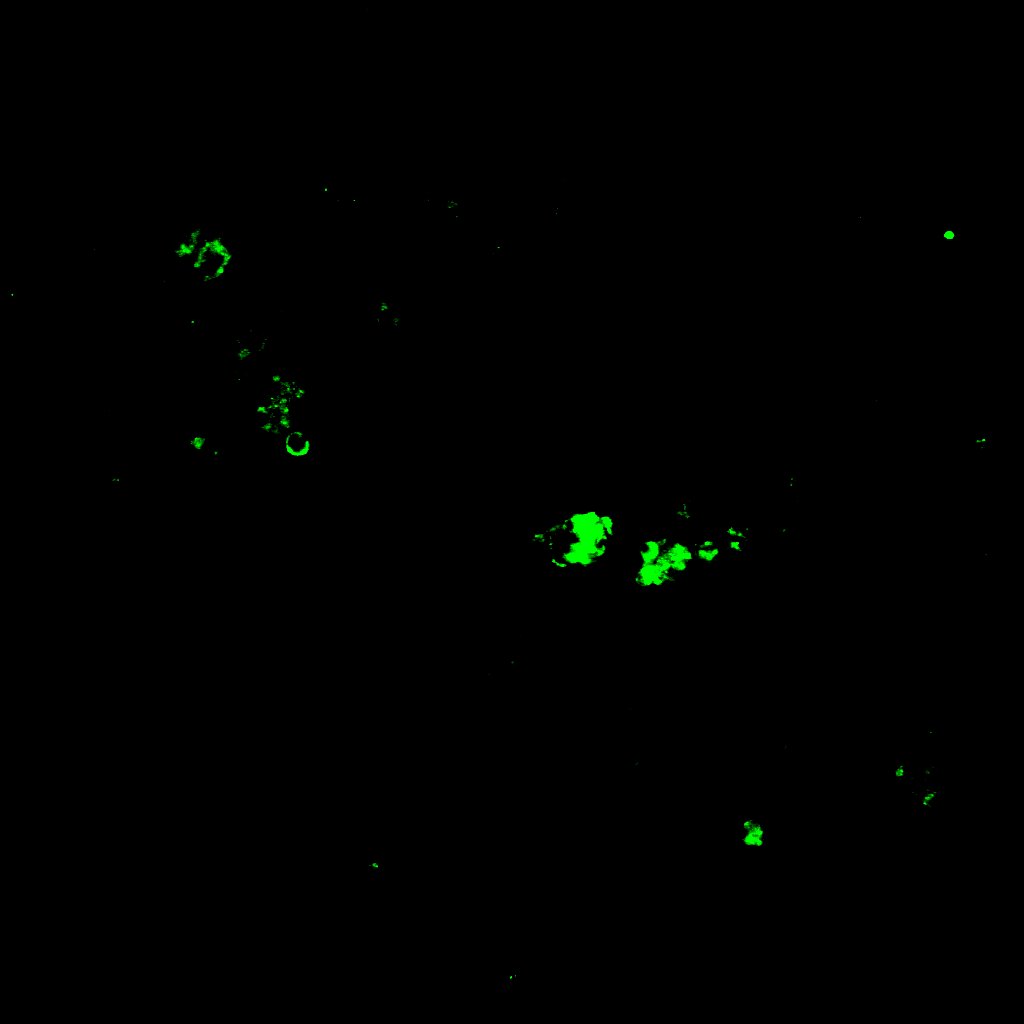

Supplement: S6 File — Representative images in the revised S2C Fig were generated from raw files woGF_4_ch1/2 (W/O GF); EF10cyc_4_ch1/2 (E+F10+Cyc); EF1shh_7_ch1/2 (E+F1+Shh). (ZIP) [file pone.0239995.s007.zip › S6_File/EF1_18_ch1.jpg]

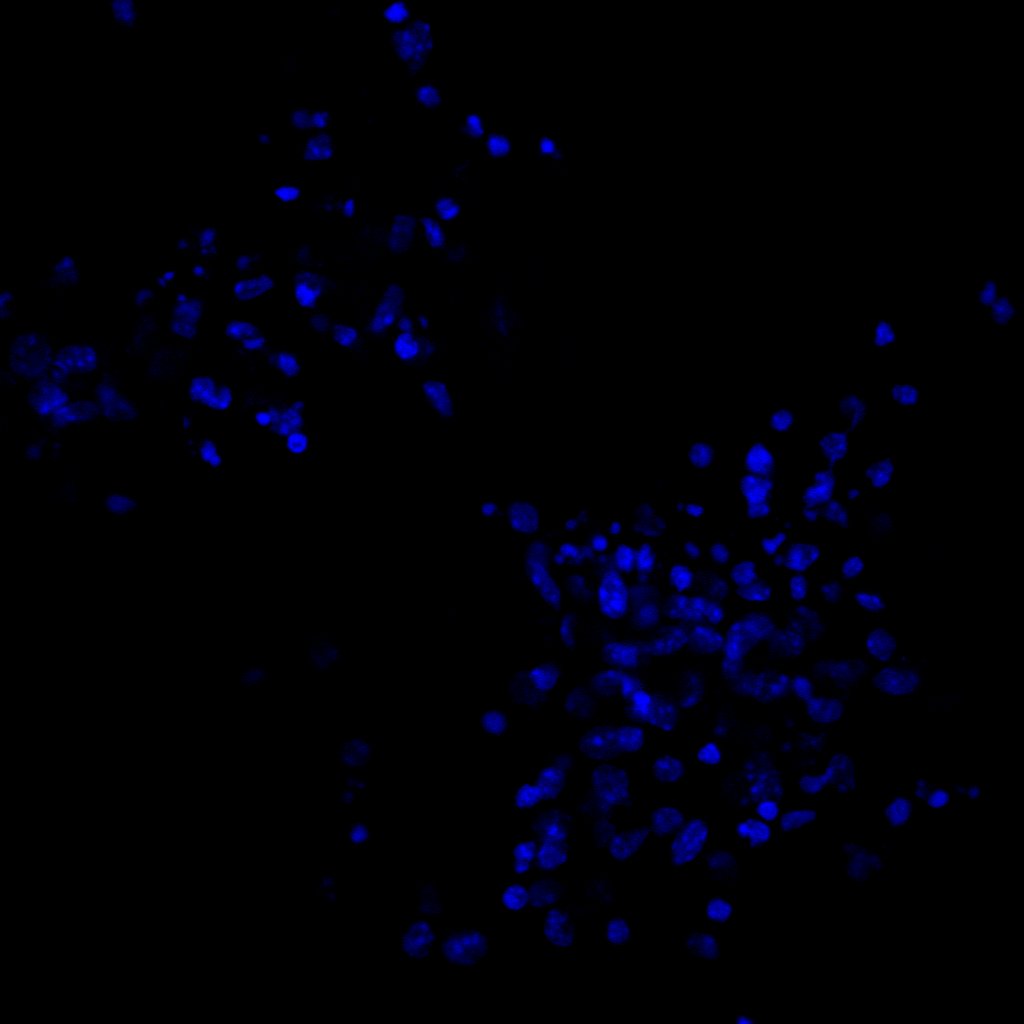

Supplement: S6 File — Representative images in the revised S2C Fig were generated from raw files woGF_4_ch1/2 (W/O GF); EF10cyc_4_ch1/2 (E+F10+Cyc); EF1shh_7_ch1/2 (E+F1+Shh). (ZIP) [file pone.0239995.s007.zip › S6_File/EF1_18_ch2.jpg]

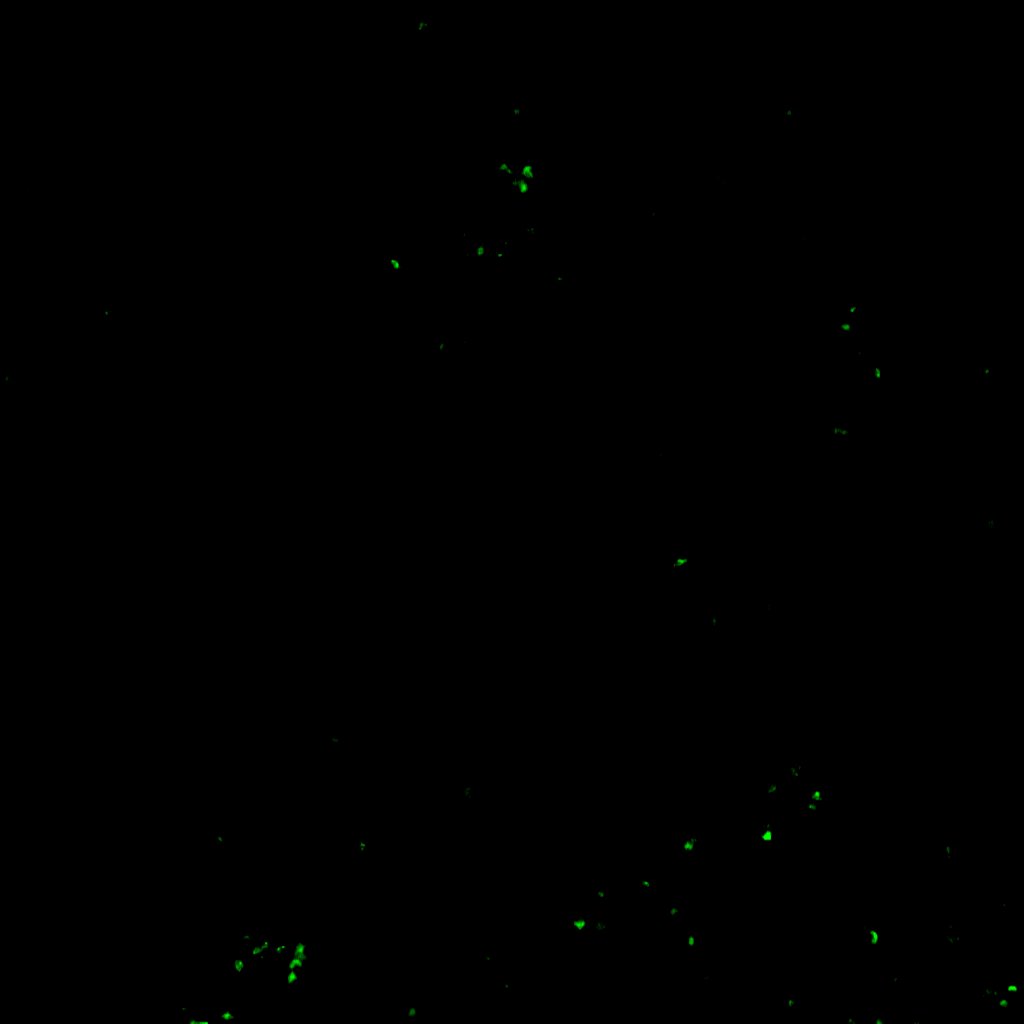

Supplement: S6 File — Representative images in the revised S2C Fig were generated from raw files woGF_4_ch1/2 (W/O GF); EF10cyc_4_ch1/2 (E+F10+Cyc); EF1shh_7_ch1/2 (E+F1+Shh). (ZIP) [file pone.0239995.s007.zip › S6_File/EF1_2_ch1.jpg]

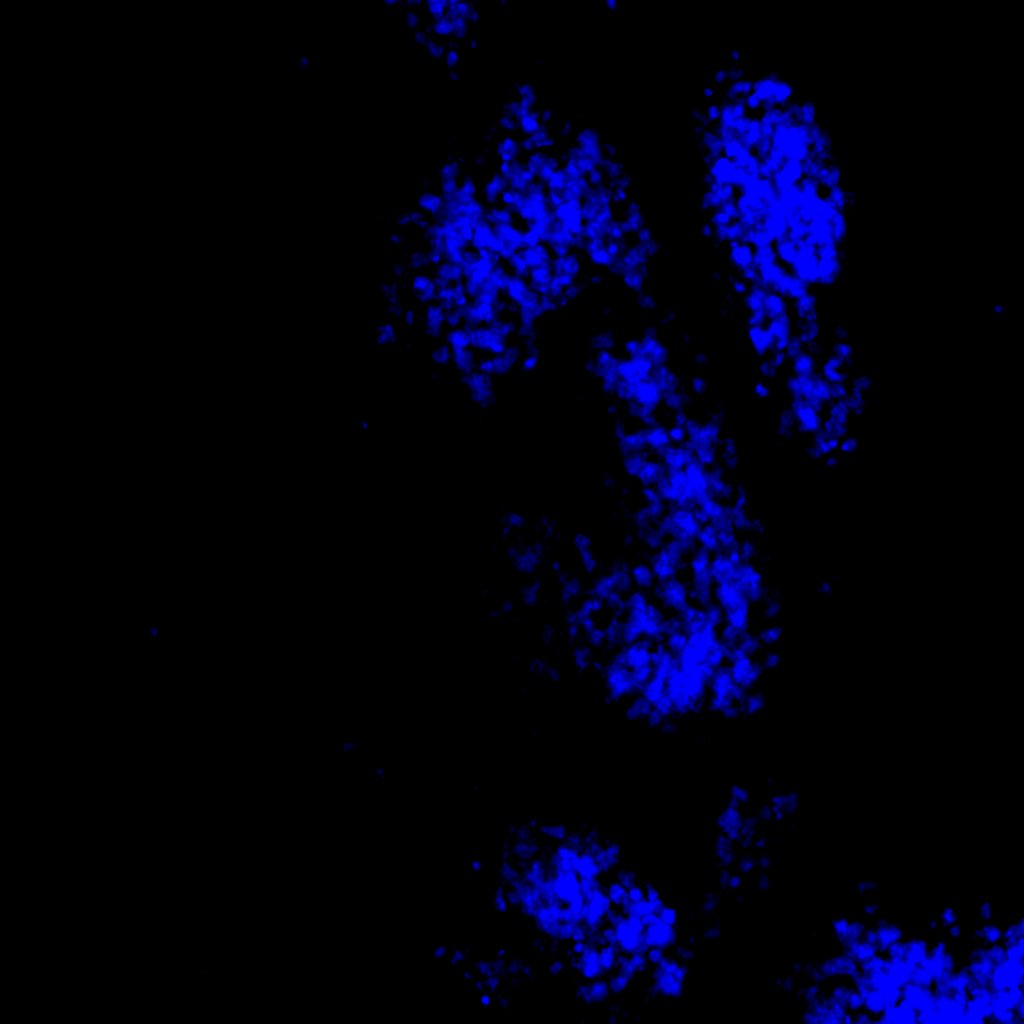

Supplement: S6 File — Representative images in the revised S2C Fig were generated from raw files woGF_4_ch1/2 (W/O GF); EF10cyc_4_ch1/2 (E+F10+Cyc); EF1shh_7_ch1/2 (E+F1+Shh). (ZIP) [file pone.0239995.s007.zip › S6_File/EF1_2_ch2.jpg]

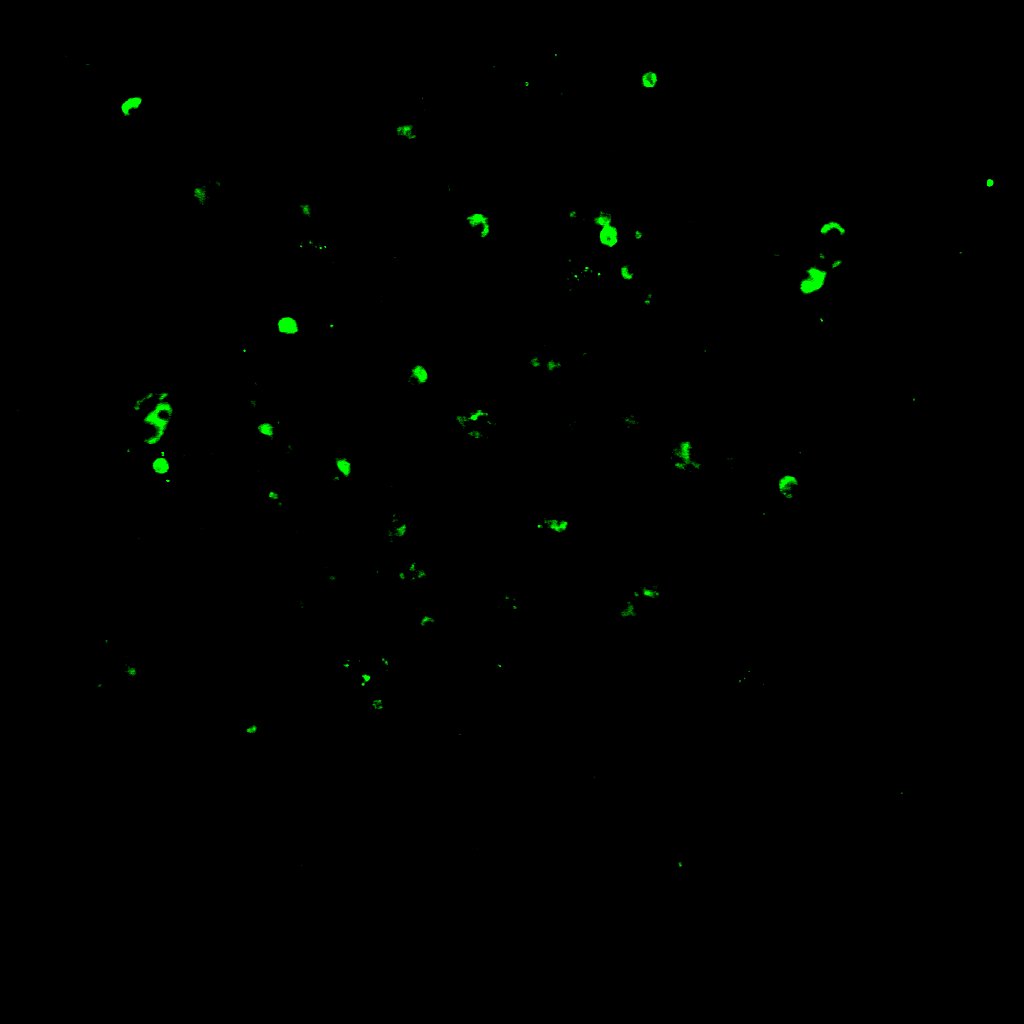

Supplement: S6 File — Representative images in the revised S2C Fig were generated from raw files woGF_4_ch1/2 (W/O GF); EF10cyc_4_ch1/2 (E+F10+Cyc); EF1shh_7_ch1/2 (E+F1+Shh). (ZIP) [file pone.0239995.s007.zip › S6_File/EF1_3_ch1.jpg]

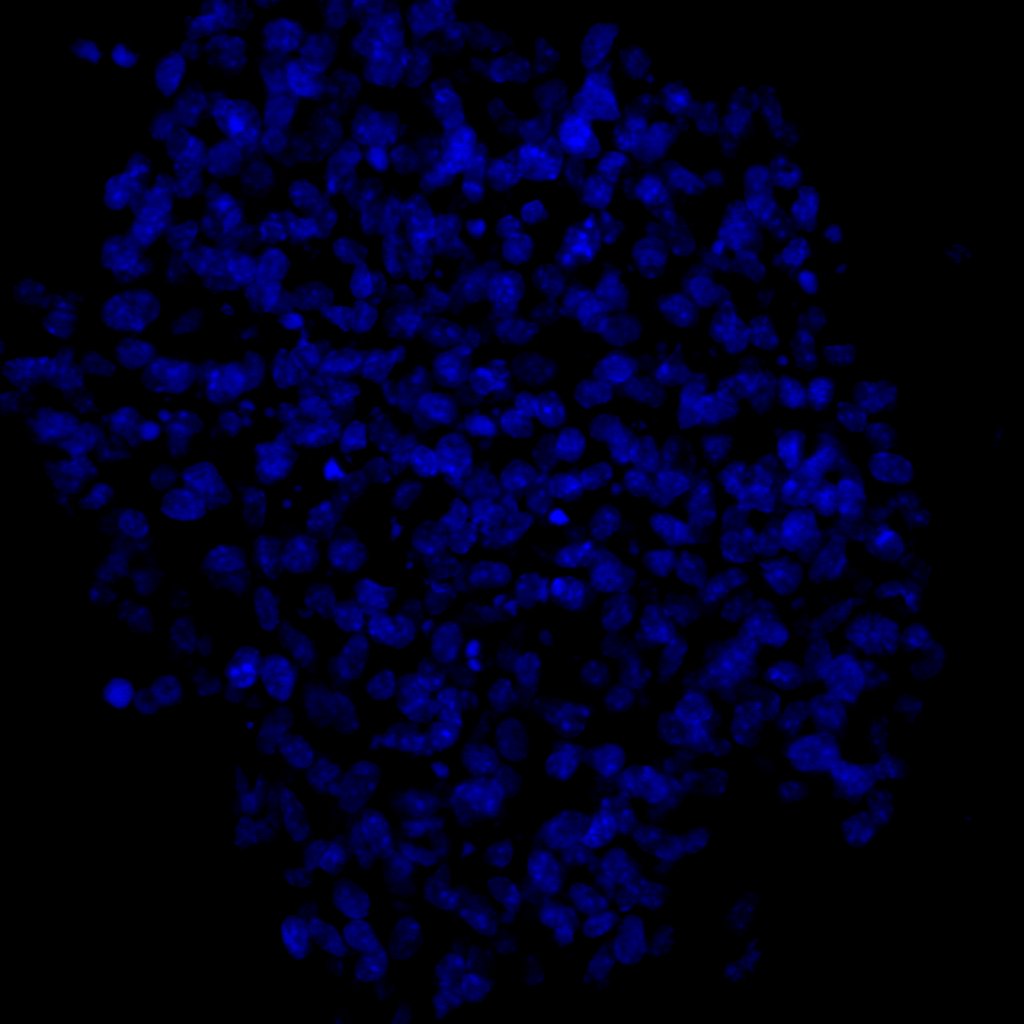

Supplement: S6 File — Representative images in the revised S2C Fig were generated from raw files woGF_4_ch1/2 (W/O GF); EF10cyc_4_ch1/2 (E+F10+Cyc); EF1shh_7_ch1/2 (E+F1+Shh). (ZIP) [file pone.0239995.s007.zip › S6_File/EF1_3_ch2.jpg]

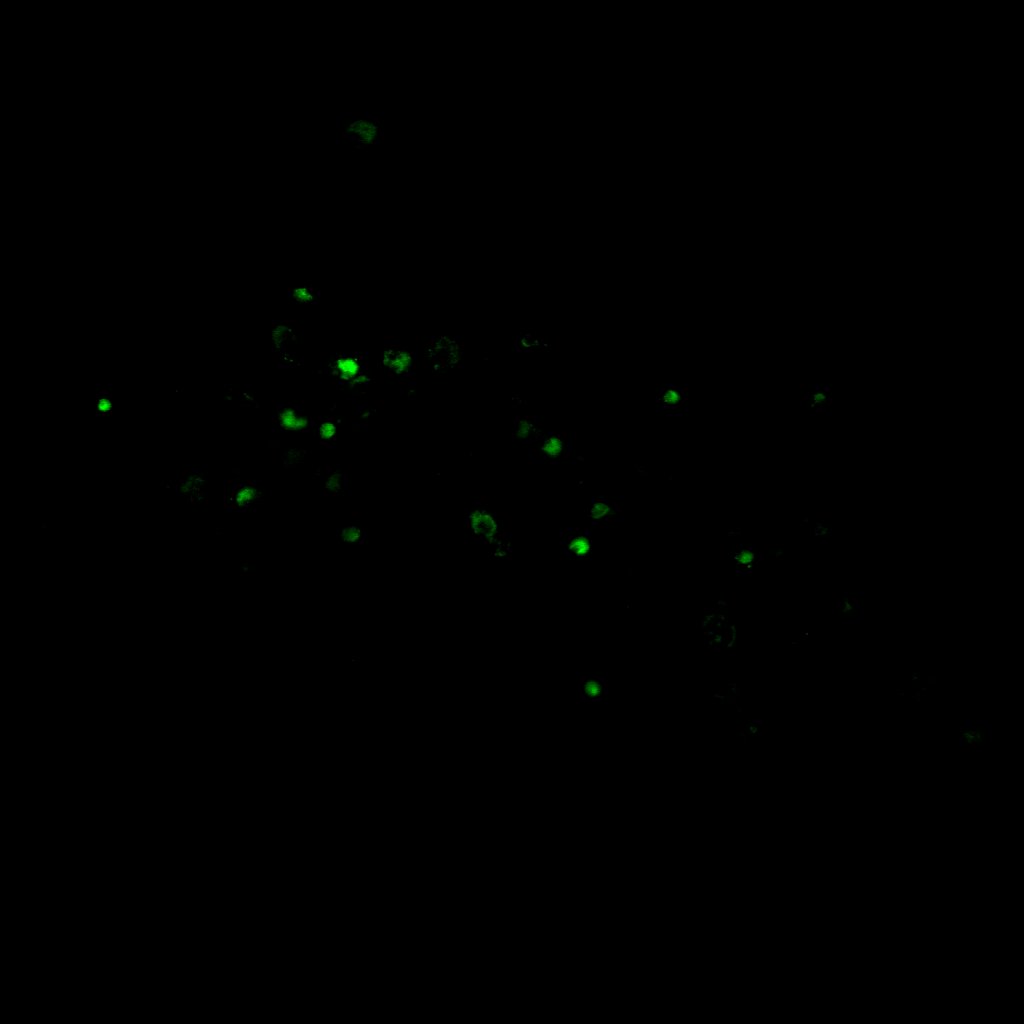

Supplement: S6 File — Representative images in the revised S2C Fig were generated from raw files woGF_4_ch1/2 (W/O GF); EF10cyc_4_ch1/2 (E+F10+Cyc); EF1shh_7_ch1/2 (E+F1+Shh). (ZIP) [file pone.0239995.s007.zip › S6_File/EF1_4_ch1.jpg]

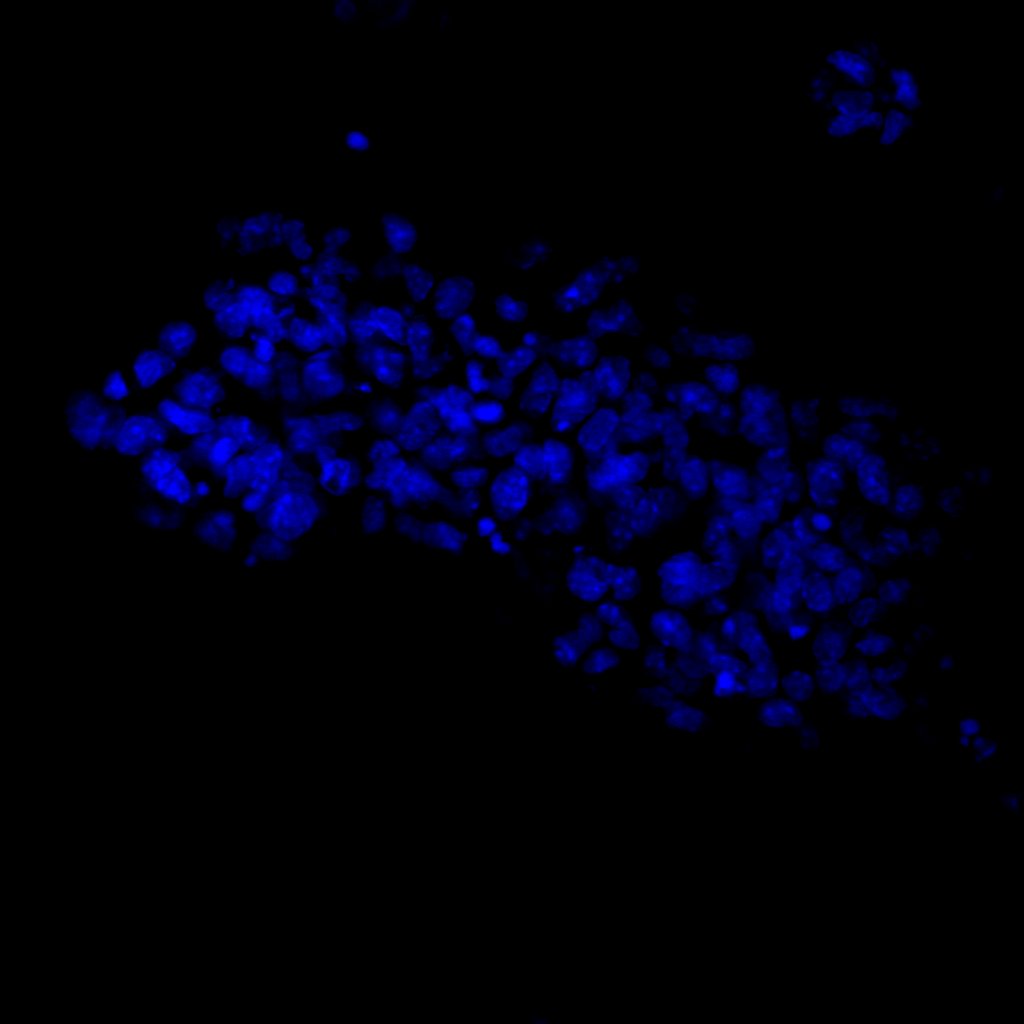

Supplement: S6 File — Representative images in the revised S2C Fig were generated from raw files woGF_4_ch1/2 (W/O GF); EF10cyc_4_ch1/2 (E+F10+Cyc); EF1shh_7_ch1/2 (E+F1+Shh). (ZIP) [file pone.0239995.s007.zip › S6_File/EF1_4_ch2.jpg]

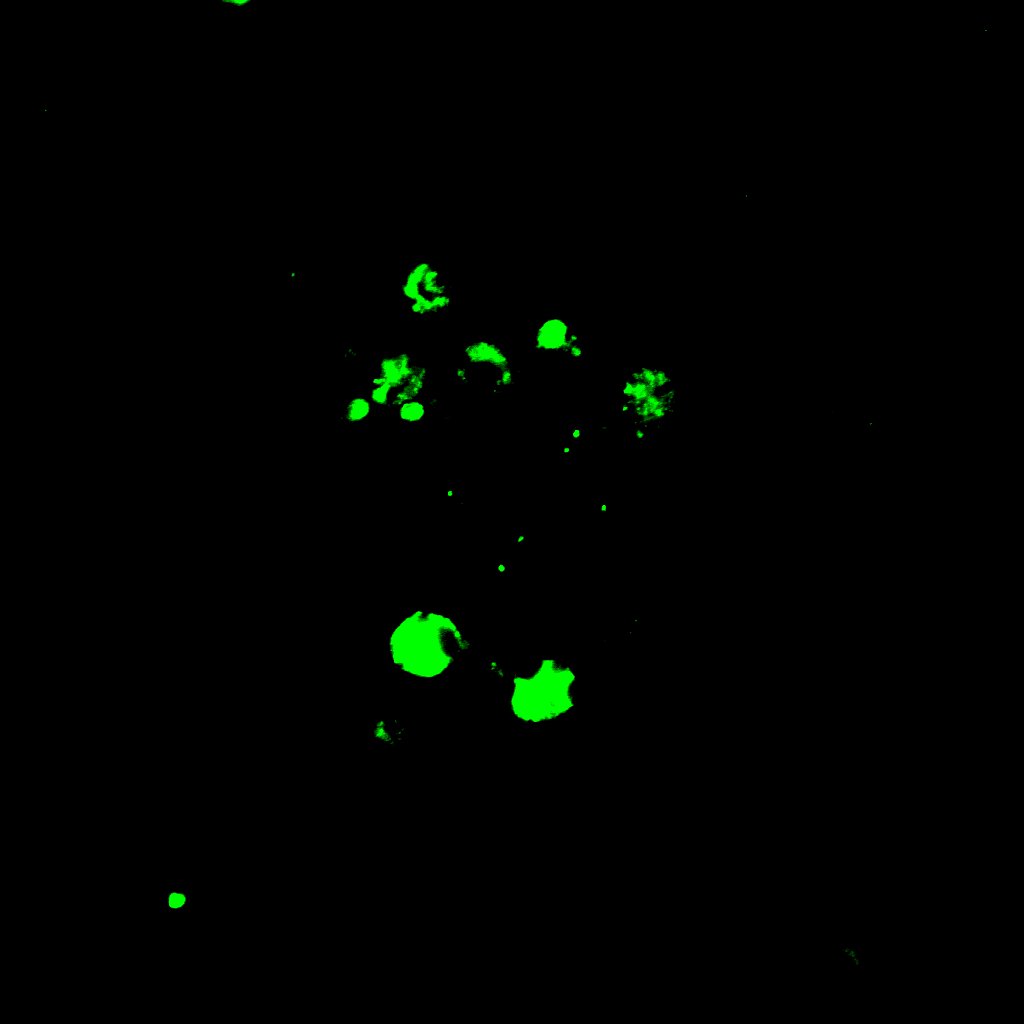

Supplement: S6 File — Representative images in the revised S2C Fig were generated from raw files woGF_4_ch1/2 (W/O GF); EF10cyc_4_ch1/2 (E+F10+Cyc); EF1shh_7_ch1/2 (E+F1+Shh). (ZIP) [file pone.0239995.s007.zip › S6_File/EF1_5_ch1.jpg]

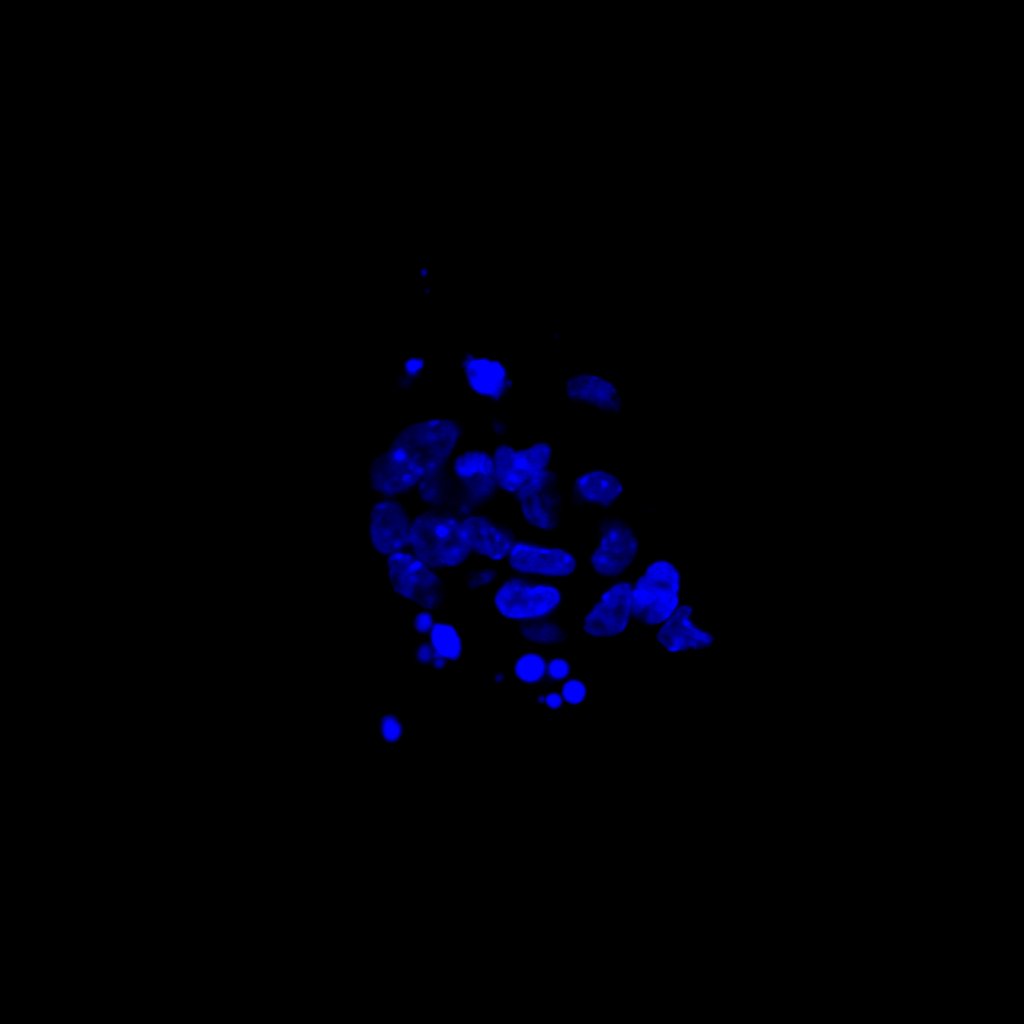

Supplement: S6 File — Representative images in the revised S2C Fig were generated from raw files woGF_4_ch1/2 (W/O GF); EF10cyc_4_ch1/2 (E+F10+Cyc); EF1shh_7_ch1/2 (E+F1+Shh). (ZIP) [file pone.0239995.s007.zip › S6_File/EF1_5_ch2.jpg]

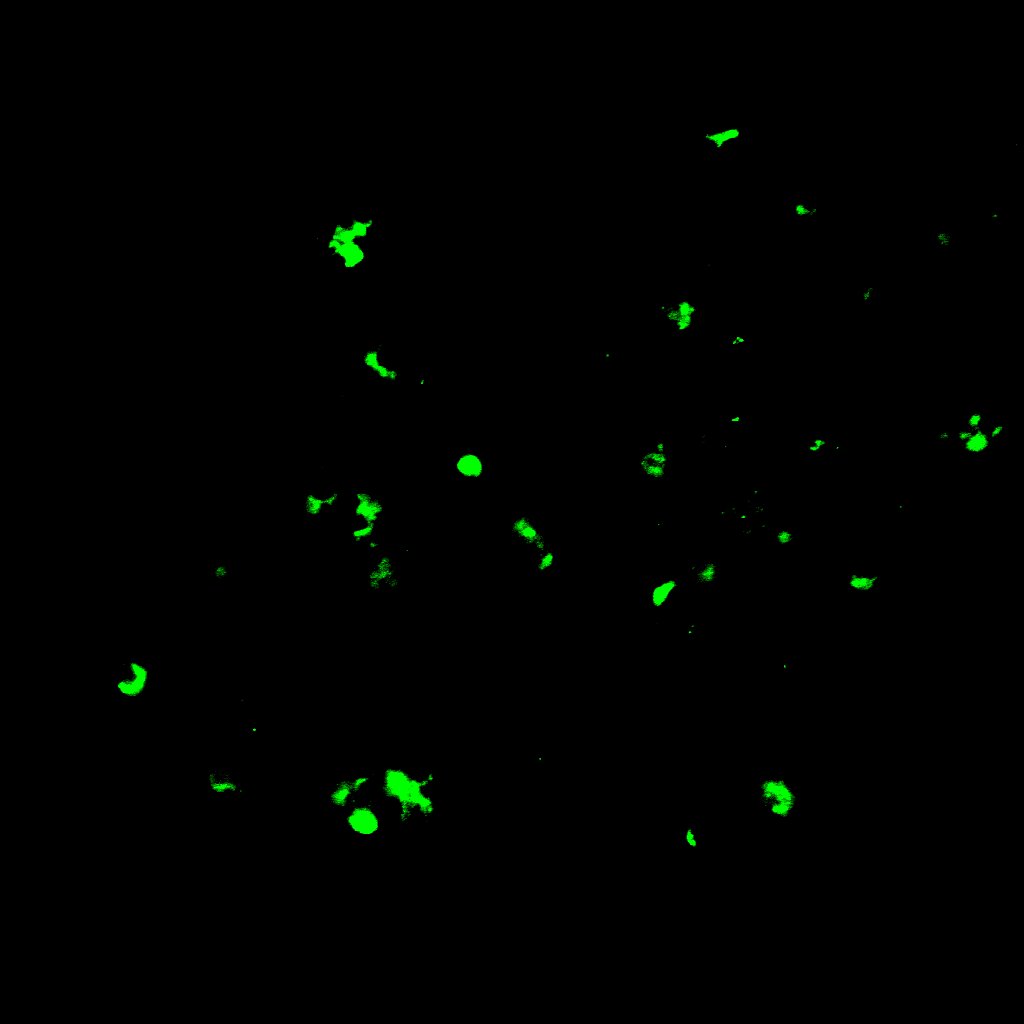

Supplement: S6 File — Representative images in the revised S2C Fig were generated from raw files woGF_4_ch1/2 (W/O GF); EF10cyc_4_ch1/2 (E+F10+Cyc); EF1shh_7_ch1/2 (E+F1+Shh). (ZIP) [file pone.0239995.s007.zip › S6_File/EF1_6_ch1.jpg]

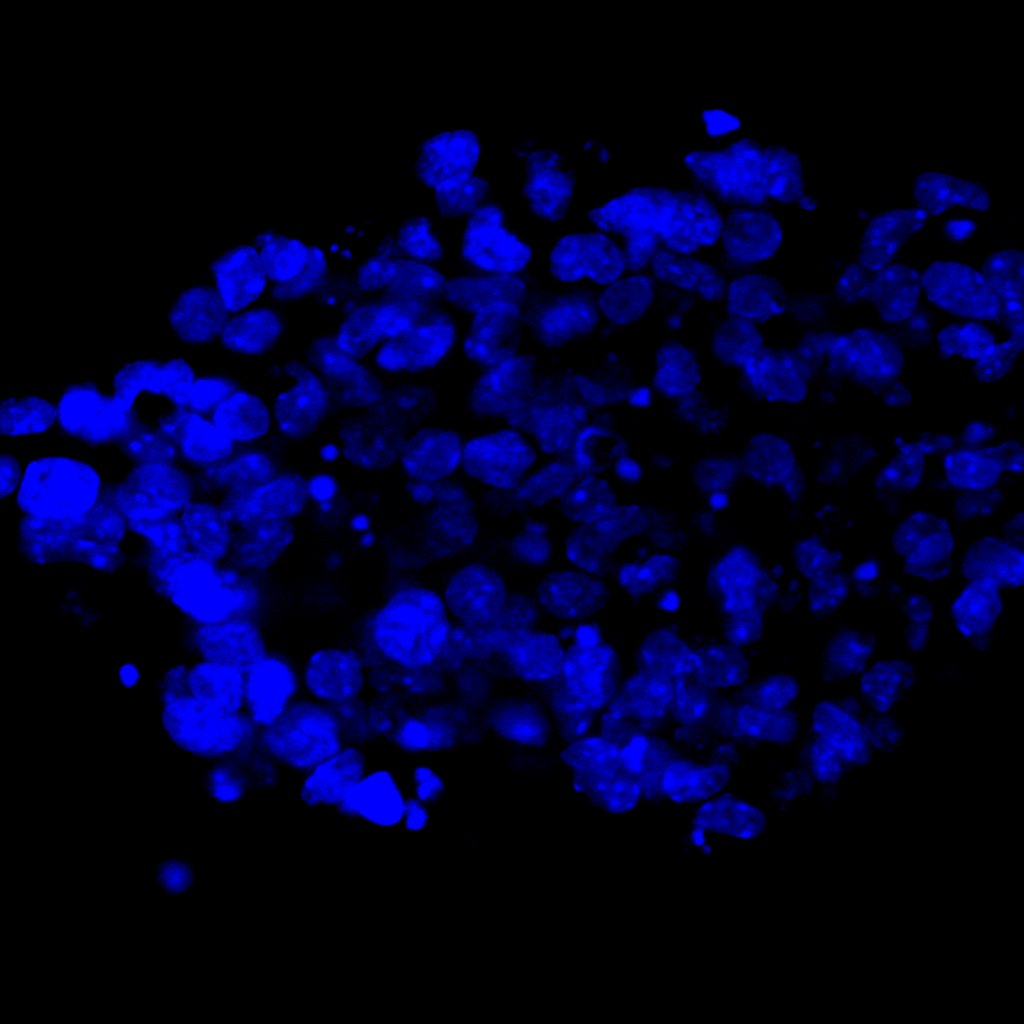

Supplement: S6 File — Representative images in the revised S2C Fig were generated from raw files woGF_4_ch1/2 (W/O GF); EF10cyc_4_ch1/2 (E+F10+Cyc); EF1shh_7_ch1/2 (E+F1+Shh). (ZIP) [file pone.0239995.s007.zip › S6_File/EF1_6_ch2.jpg]

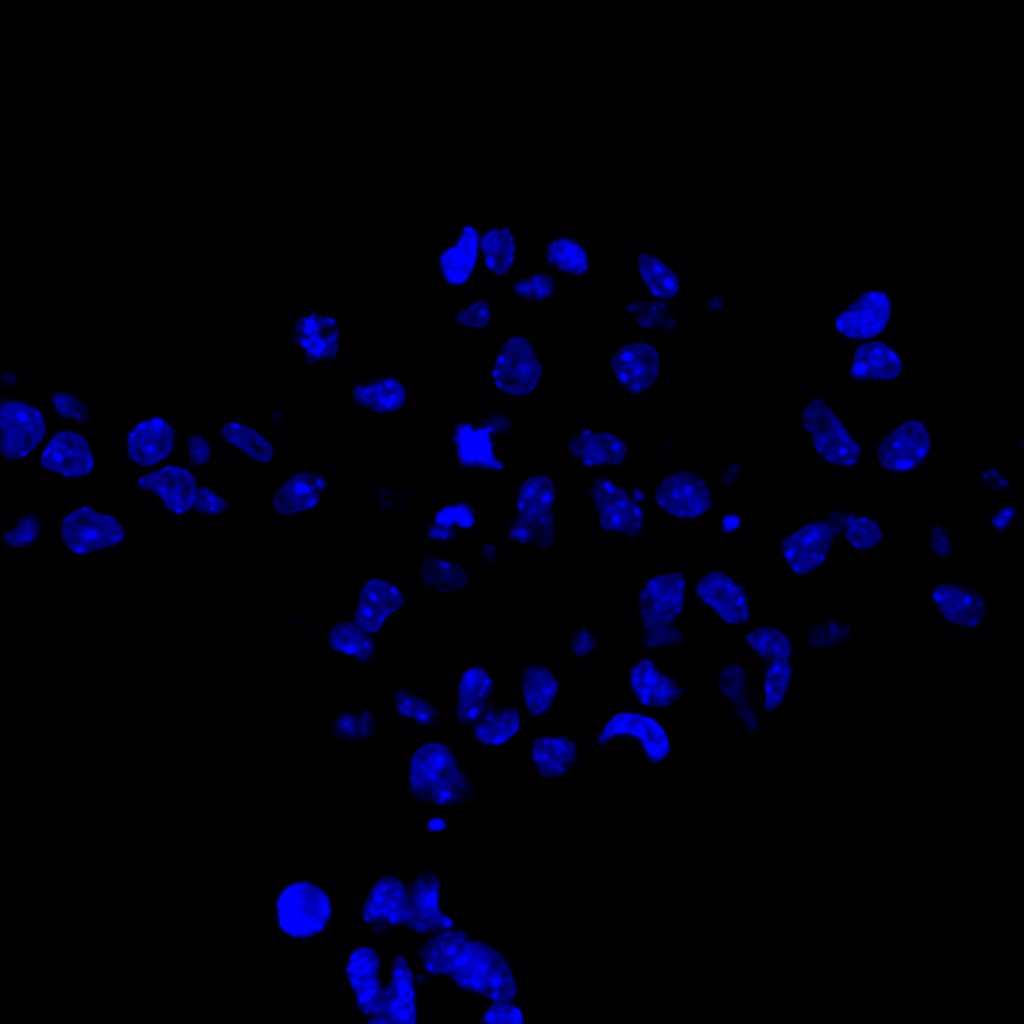

Supplement: S6 File — Representative images in the revised S2C Fig were generated from raw files woGF_4_ch1/2 (W/O GF); EF10cyc_4_ch1/2 (E+F10+Cyc); EF1shh_7_ch1/2 (E+F1+Shh). (ZIP) [file pone.0239995.s007.zip › S6_File/EF1_7_cch2.jpg]

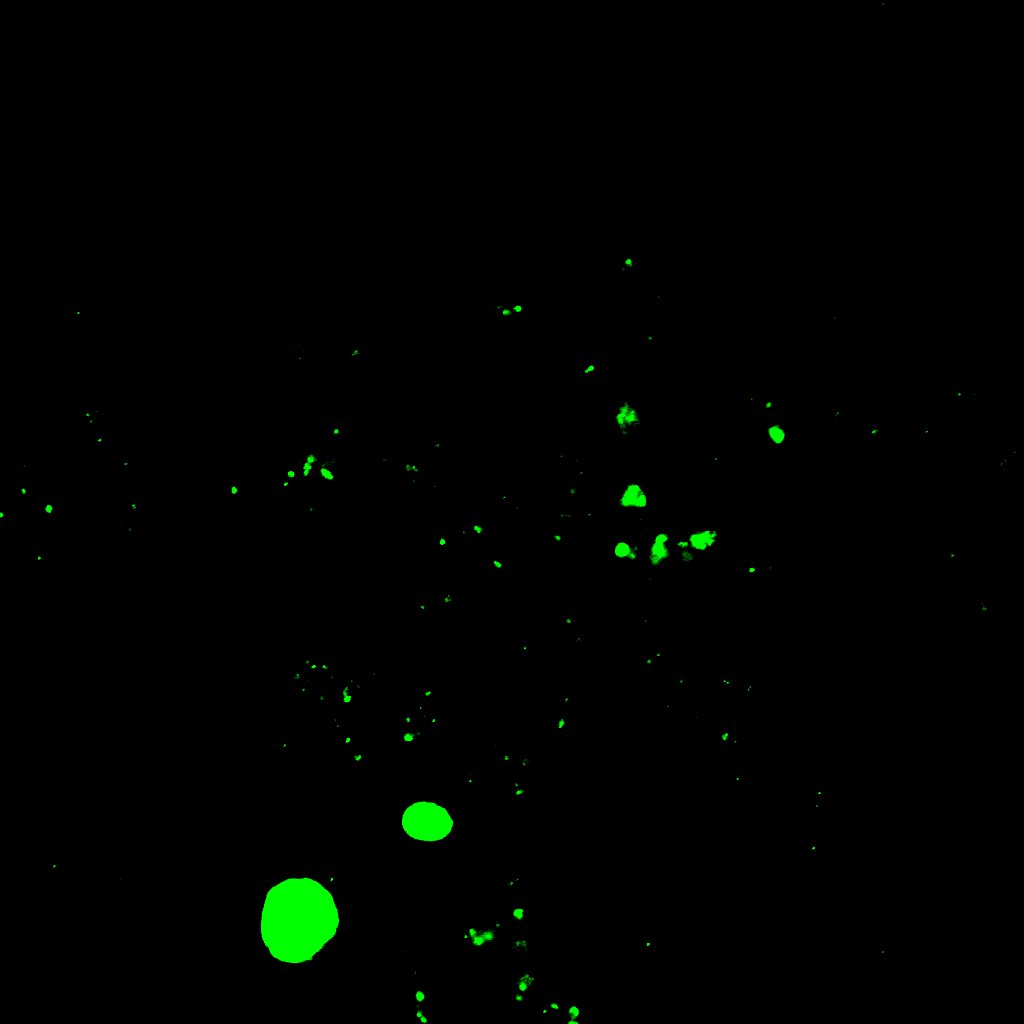

Supplement: S6 File — Representative images in the revised S2C Fig were generated from raw files woGF_4_ch1/2 (W/O GF); EF10cyc_4_ch1/2 (E+F10+Cyc); EF1shh_7_ch1/2 (E+F1+Shh). (ZIP) [file pone.0239995.s007.zip › S6_File/EF1_7_ch1.jpg]

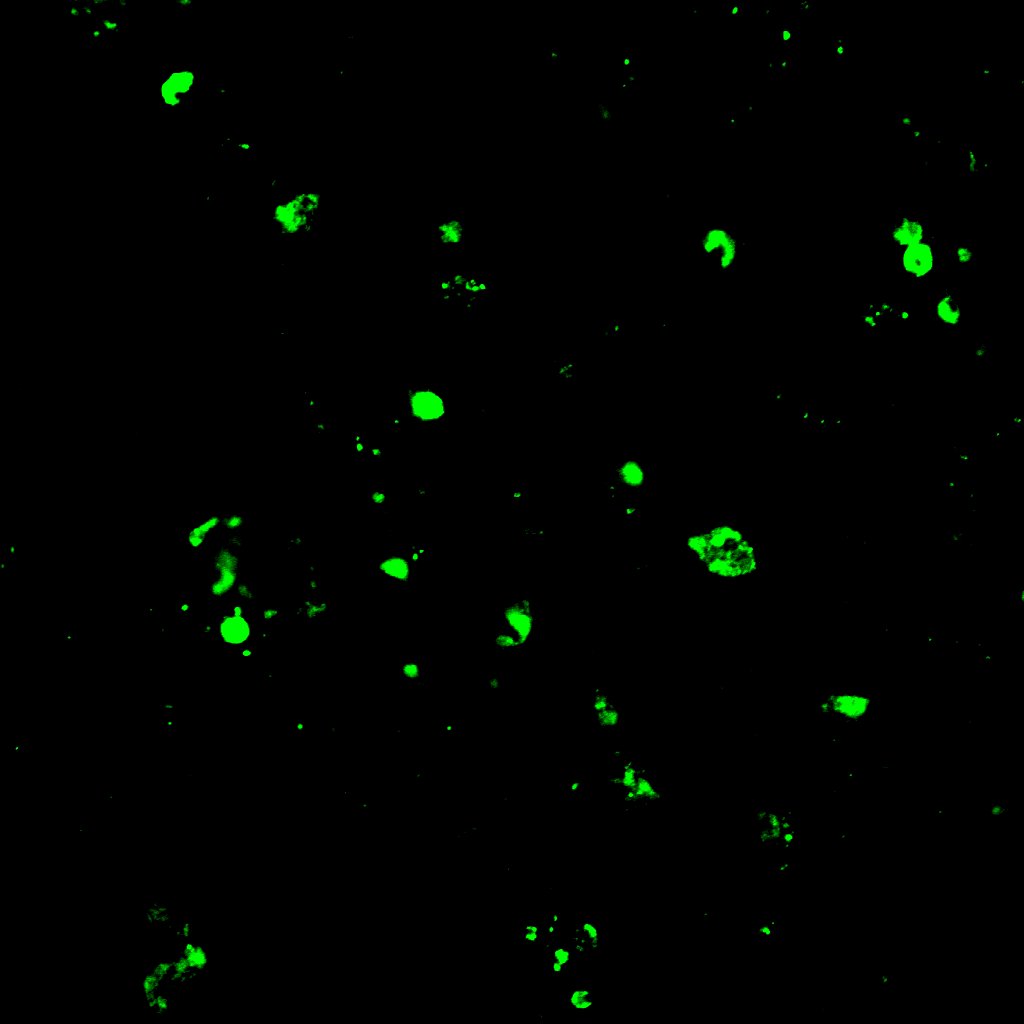

Supplement: S6 File — Representative images in the revised S2C Fig were generated from raw files woGF_4_ch1/2 (W/O GF); EF10cyc_4_ch1/2 (E+F10+Cyc); EF1shh_7_ch1/2 (E+F1+Shh). (ZIP) [file pone.0239995.s007.zip › S6_File/EF1_8_ch1.jpg]

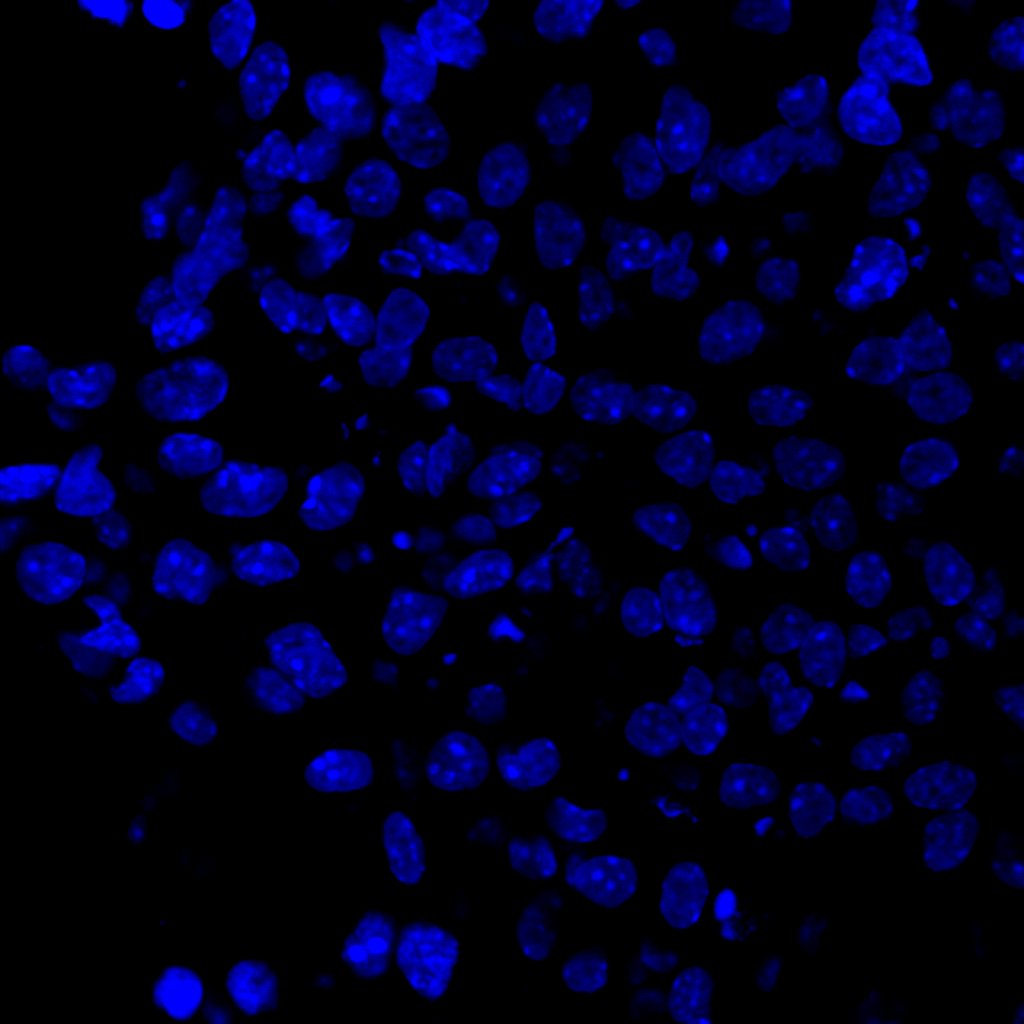

Supplement: S6 File — Representative images in the revised S2C Fig were generated from raw files woGF_4_ch1/2 (W/O GF); EF10cyc_4_ch1/2 (E+F10+Cyc); EF1shh_7_ch1/2 (E+F1+Shh). (ZIP) [file pone.0239995.s007.zip › S6_File/EF1_8_ch2.jpg]

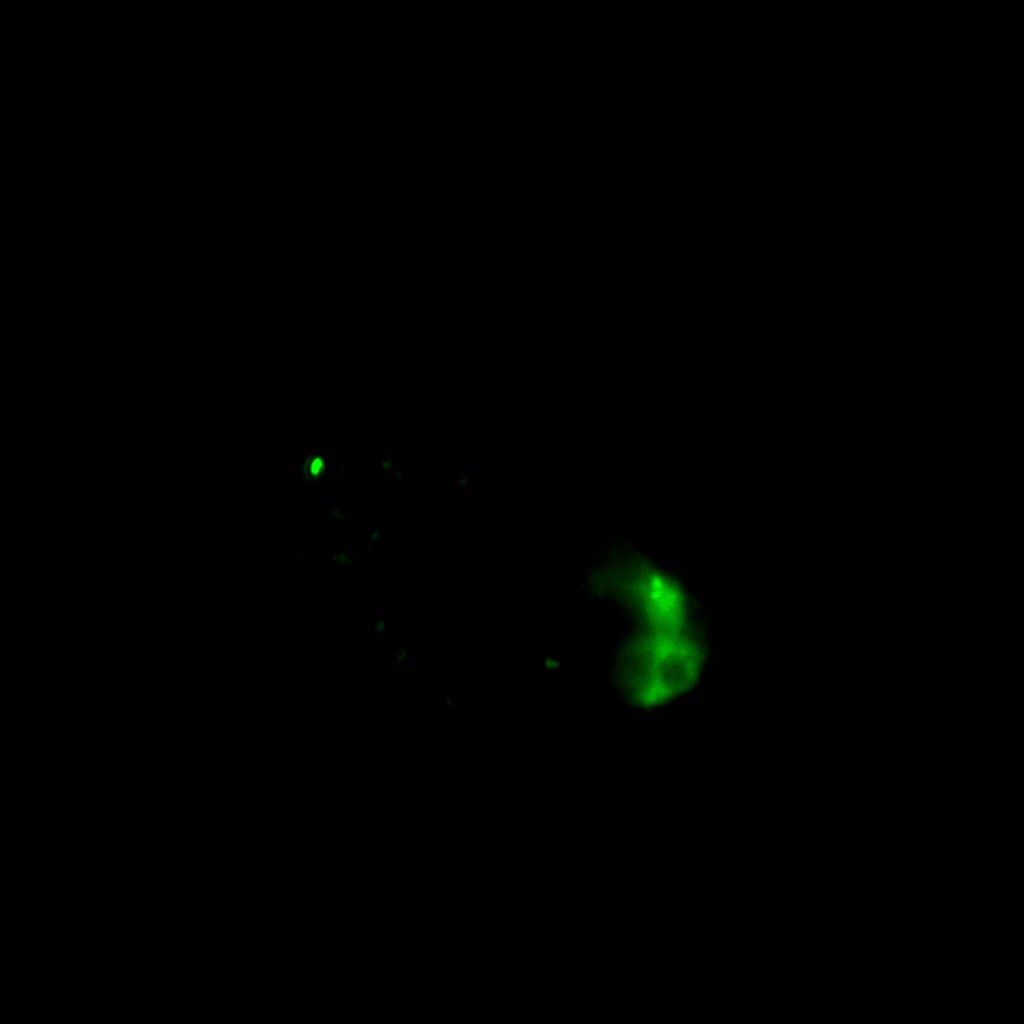

Supplement: S6 File — Representative images in the revised S2C Fig were generated from raw files woGF_4_ch1/2 (W/O GF); EF10cyc_4_ch1/2 (E+F10+Cyc); EF1shh_7_ch1/2 (E+F1+Shh). (ZIP) [file pone.0239995.s007.zip › S6_File/EF1_9_ch1.jpg]

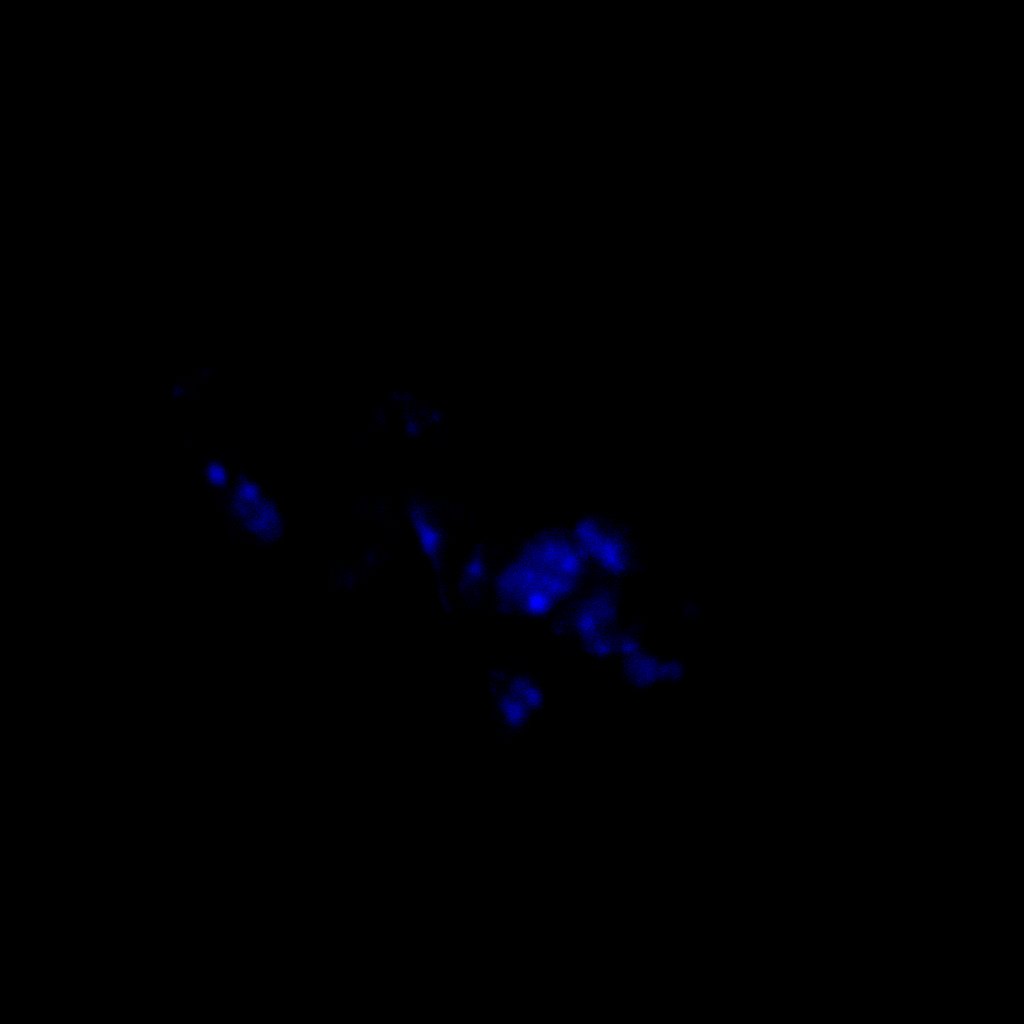

Supplement: S6 File — Representative images in the revised S2C Fig were generated from raw files woGF_4_ch1/2 (W/O GF); EF10cyc_4_ch1/2 (E+F10+Cyc); EF1shh_7_ch1/2 (E+F1+Shh). (ZIP) [file pone.0239995.s007.zip › S6_File/EF1_9_ch2.jpg]

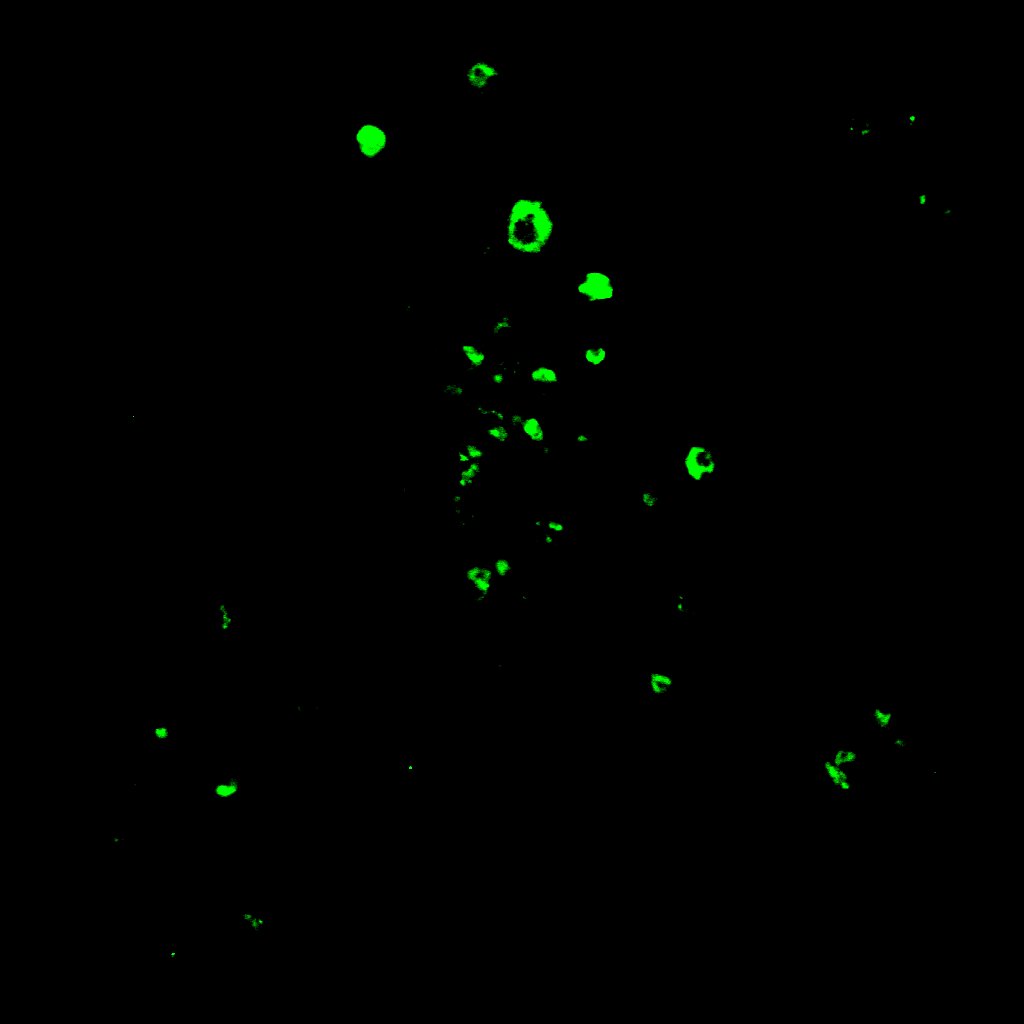

Supplement: S6 File — Representative images in the revised S2C Fig were generated from raw files woGF_4_ch1/2 (W/O GF); EF10cyc_4_ch1/2 (E+F10+Cyc); EF1shh_7_ch1/2 (E+F1+Shh). (ZIP) [file pone.0239995.s007.zip › S6_File/EF10_1_ch1.jpg]

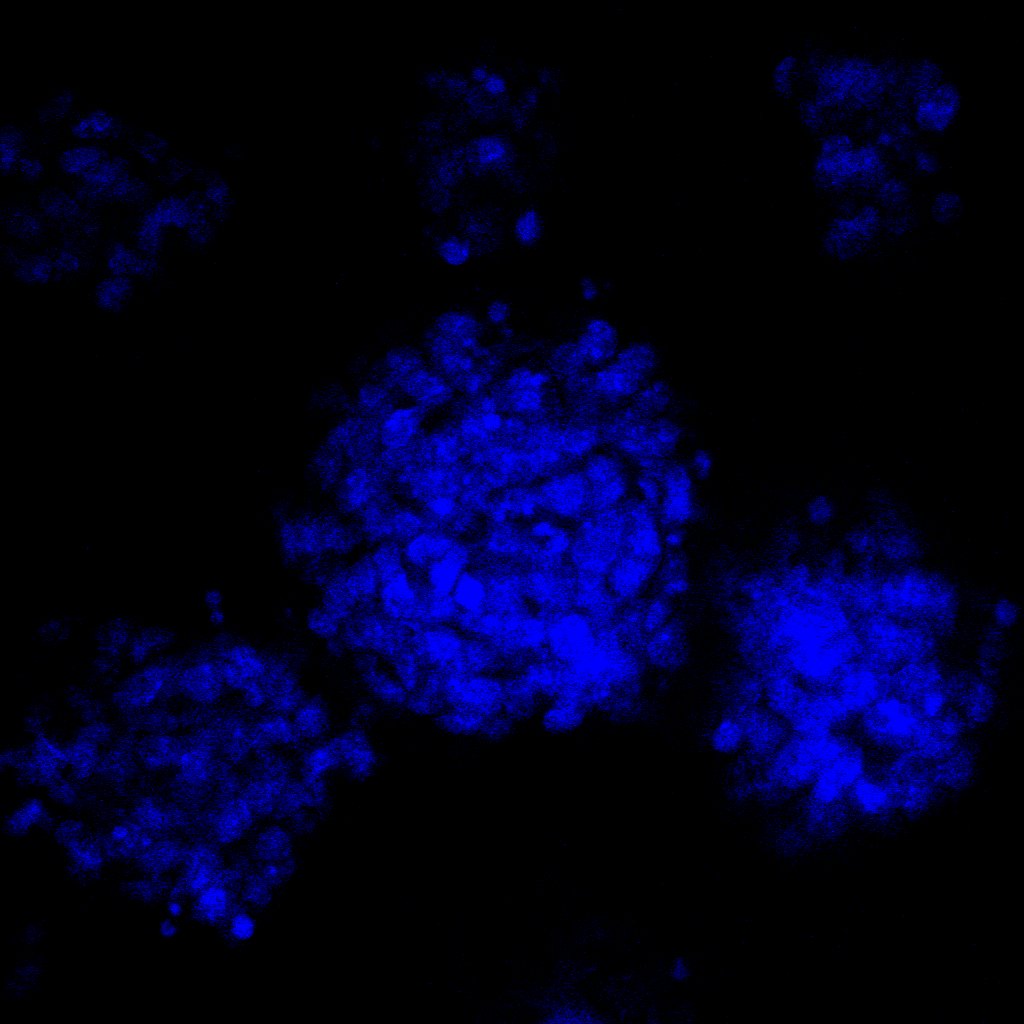

Supplement: S6 File — Representative images in the revised S2C Fig were generated from raw files woGF_4_ch1/2 (W/O GF); EF10cyc_4_ch1/2 (E+F10+Cyc); EF1shh_7_ch1/2 (E+F1+Shh). (ZIP) [file pone.0239995.s007.zip › S6_File/EF10_1_ch2.jpg]

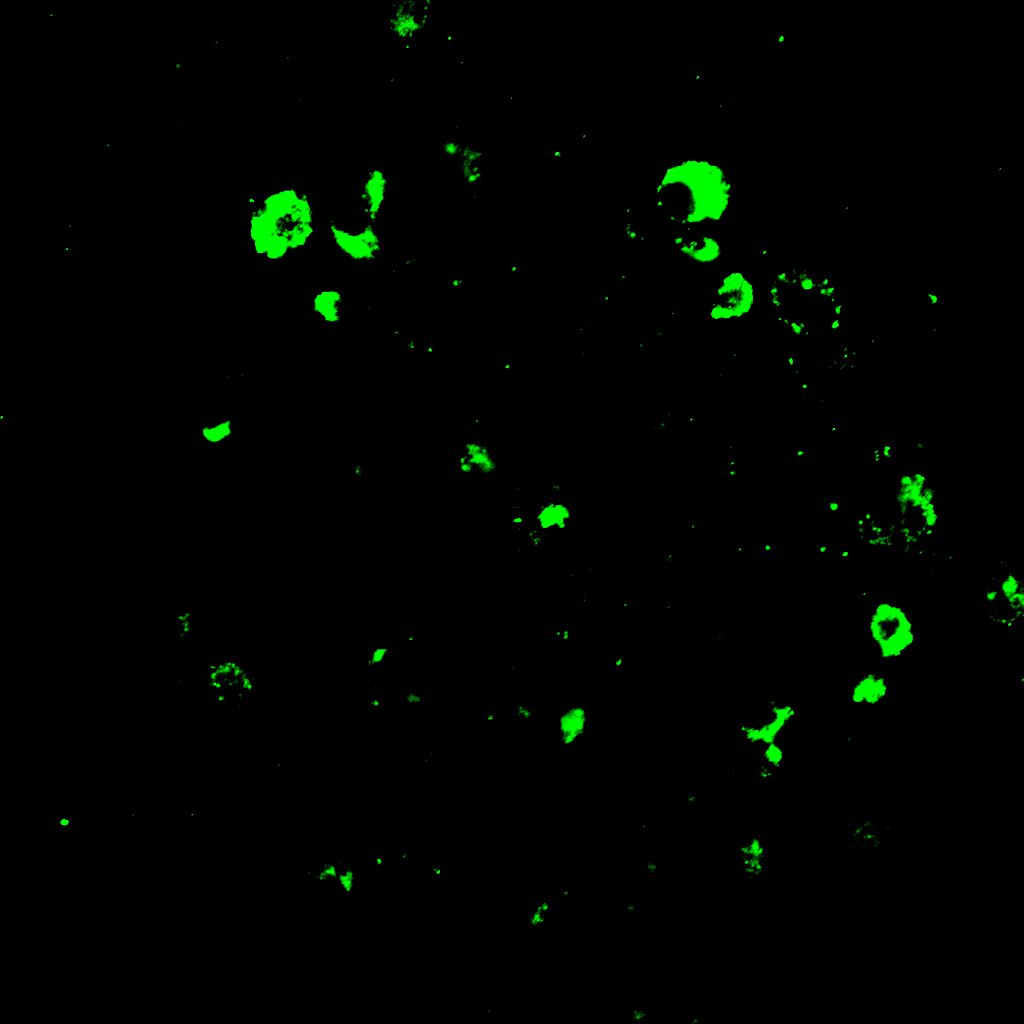

Supplement: S6 File — Representative images in the revised S2C Fig were generated from raw files woGF_4_ch1/2 (W/O GF); EF10cyc_4_ch1/2 (E+F10+Cyc); EF1shh_7_ch1/2 (E+F1+Shh). (ZIP) [file pone.0239995.s007.zip › S6_File/EF10_10_ch1.jpg]

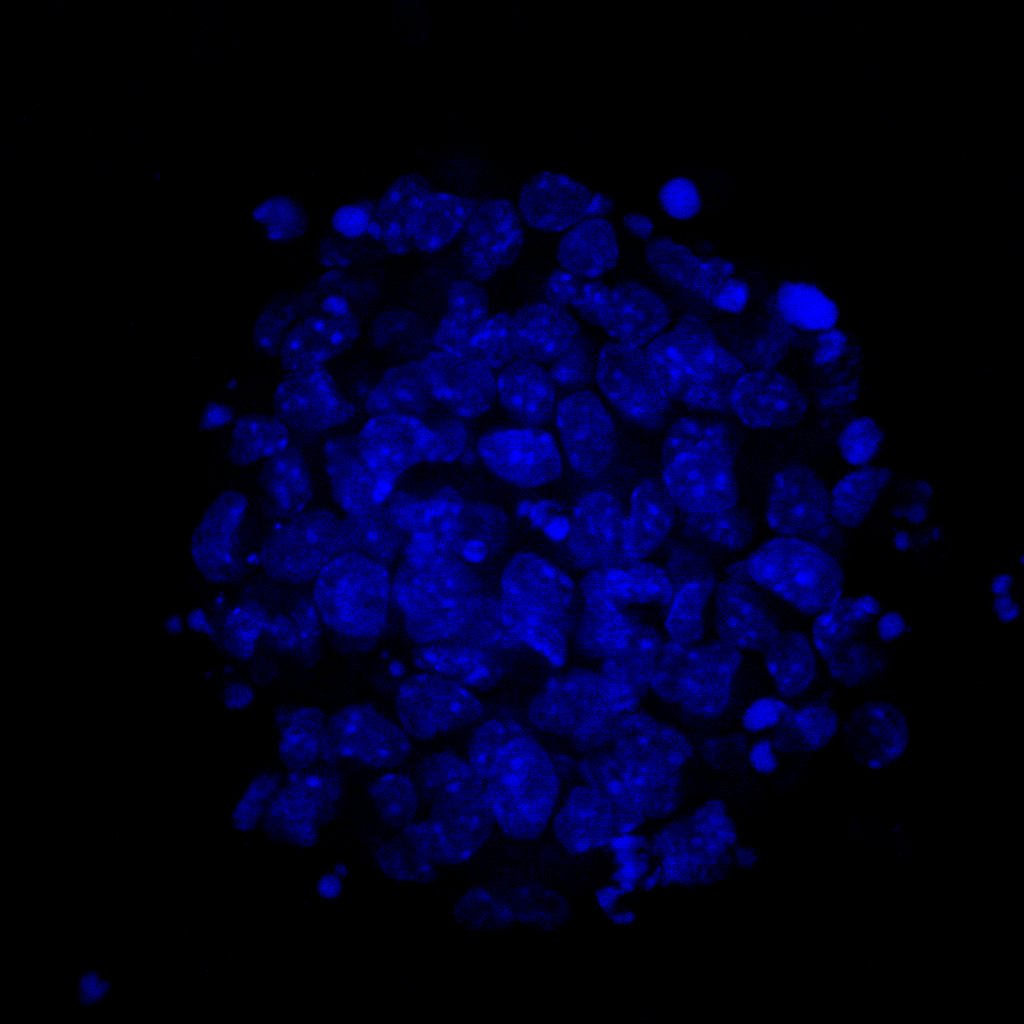

Supplement: S6 File — Representative images in the revised S2C Fig were generated from raw files woGF_4_ch1/2 (W/O GF); EF10cyc_4_ch1/2 (E+F10+Cyc); EF1shh_7_ch1/2 (E+F1+Shh). (ZIP) [file pone.0239995.s007.zip › S6_File/EF10_10_ch2.jpg]

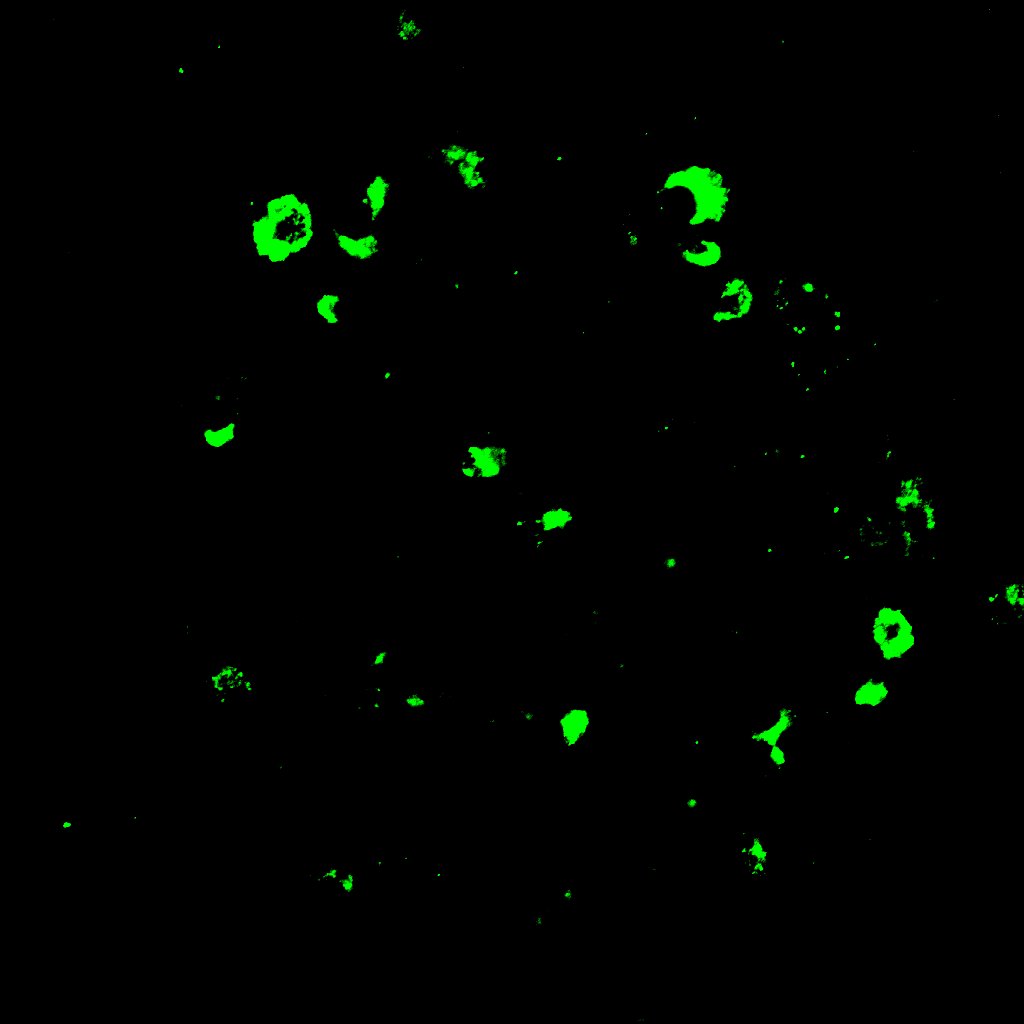

Supplement: S6 File — Representative images in the revised S2C Fig were generated from raw files woGF_4_ch1/2 (W/O GF); EF10cyc_4_ch1/2 (E+F10+Cyc); EF1shh_7_ch1/2 (E+F1+Shh). (ZIP) [file pone.0239995.s007.zip › S6_File/EF10_11_ch1.jpg]

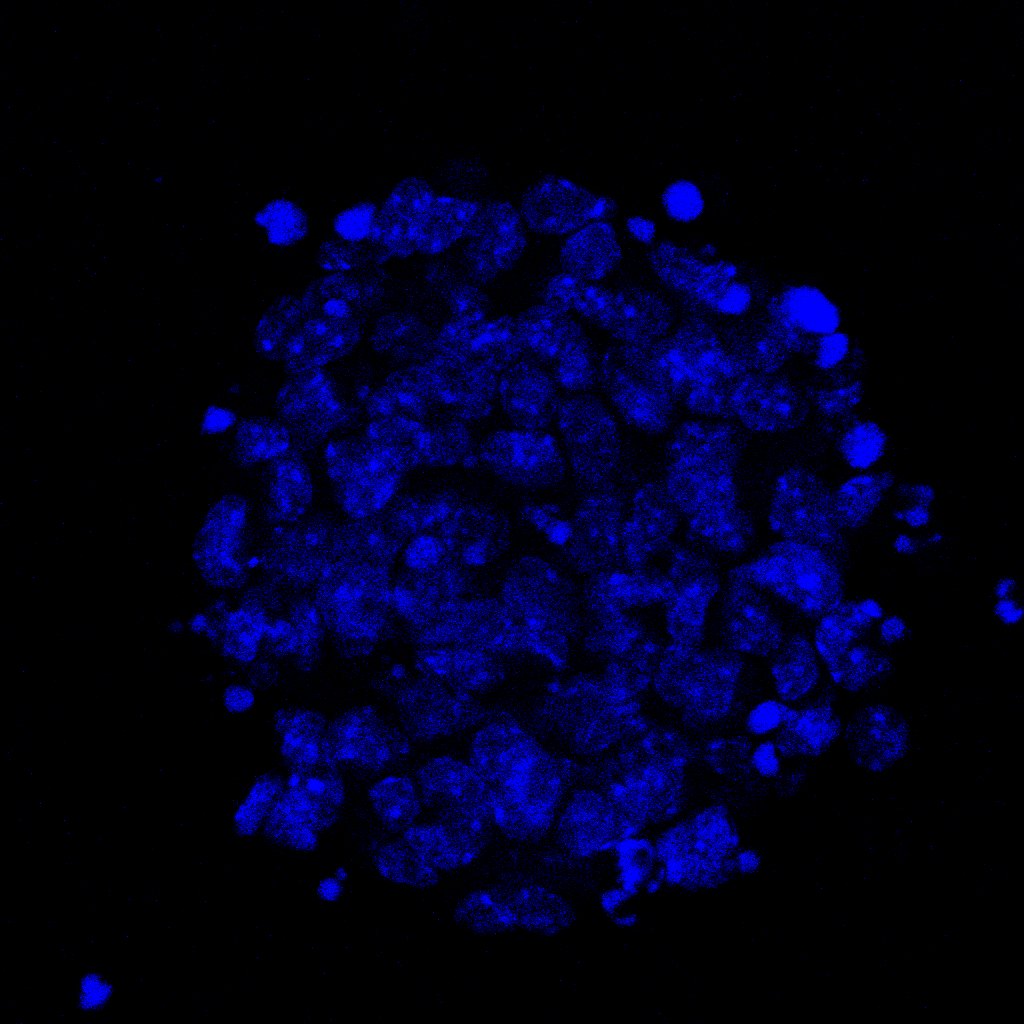

Supplement: S6 File — Representative images in the revised S2C Fig were generated from raw files woGF_4_ch1/2 (W/O GF); EF10cyc_4_ch1/2 (E+F10+Cyc); EF1shh_7_ch1/2 (E+F1+Shh). (ZIP) [file pone.0239995.s007.zip › S6_File/EF10_11_ch2.jpg]

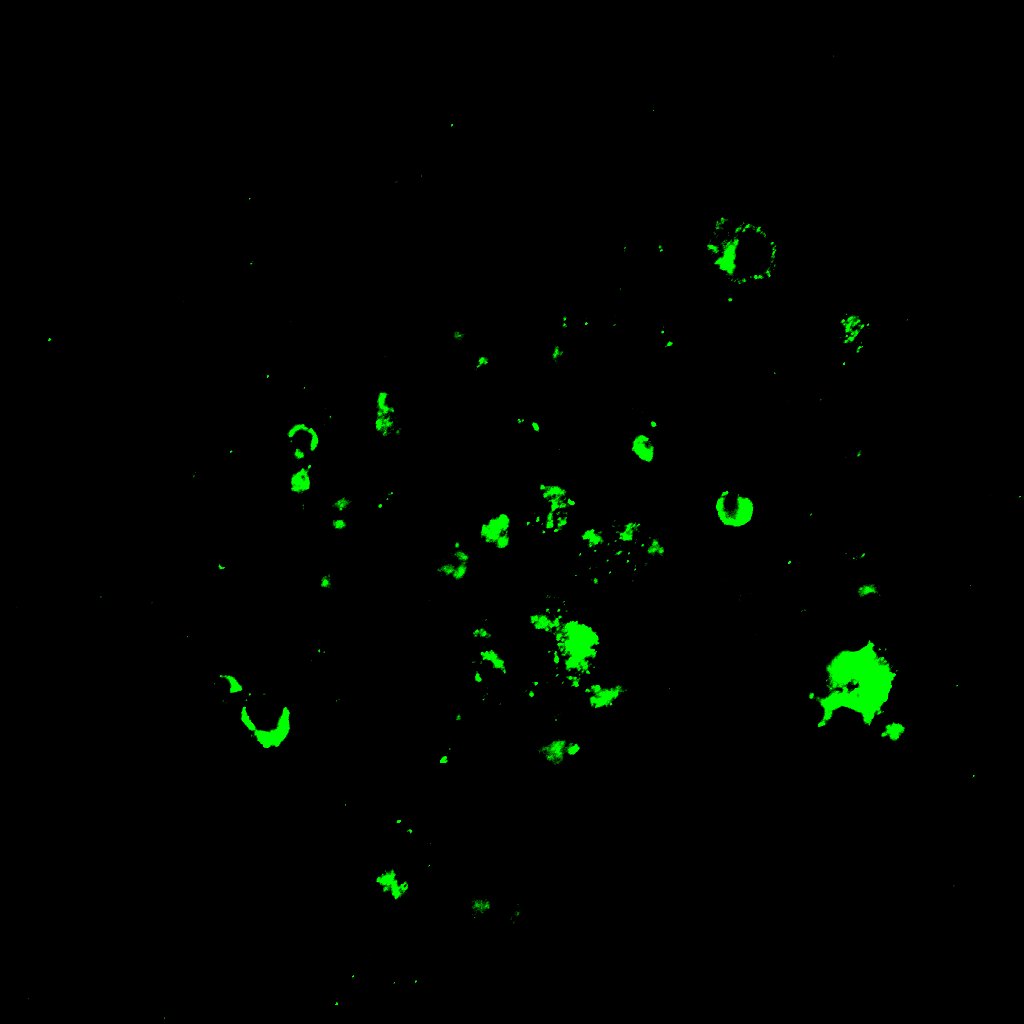

Supplement: S6 File — Representative images in the revised S2C Fig were generated from raw files woGF_4_ch1/2 (W/O GF); EF10cyc_4_ch1/2 (E+F10+Cyc); EF1shh_7_ch1/2 (E+F1+Shh). (ZIP) [file pone.0239995.s007.zip › S6_File/EF10_12_ch1.jpg]

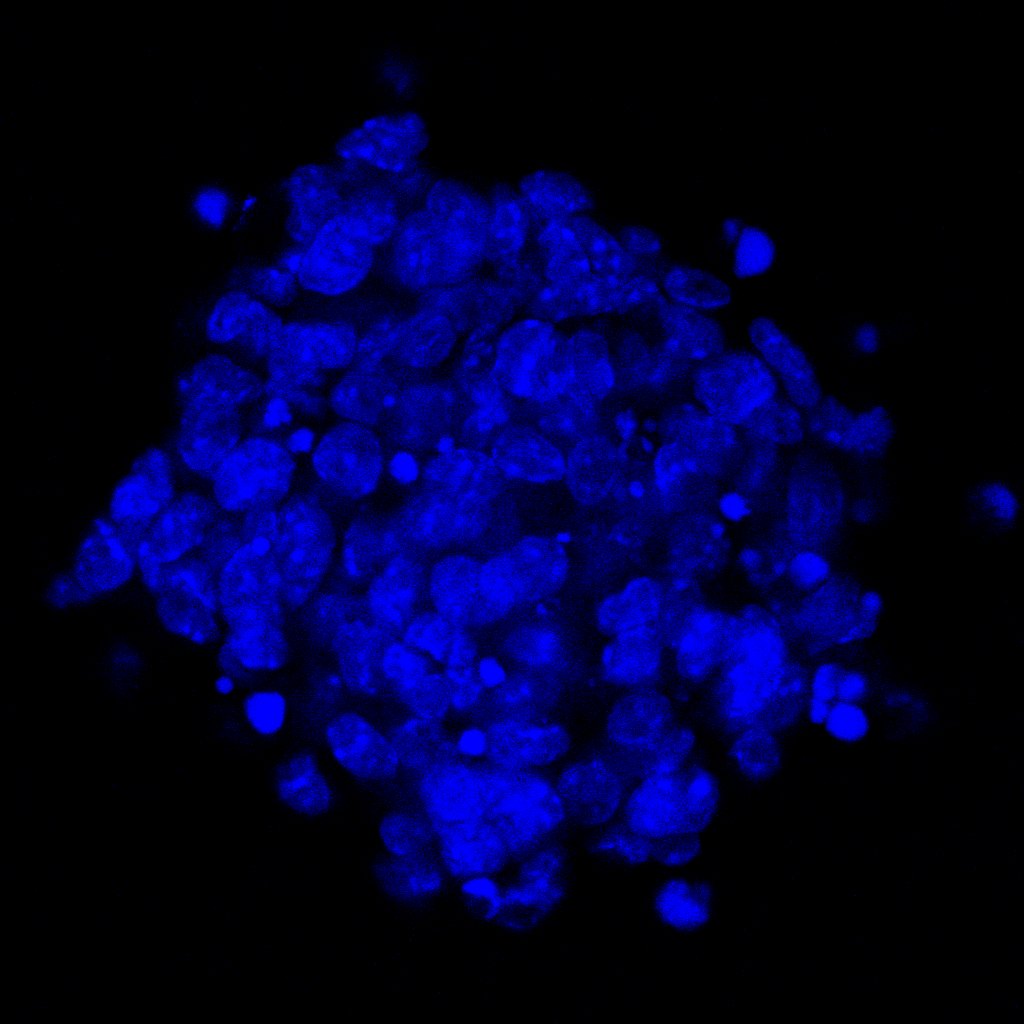

Supplement: S6 File — Representative images in the revised S2C Fig were generated from raw files woGF_4_ch1/2 (W/O GF); EF10cyc_4_ch1/2 (E+F10+Cyc); EF1shh_7_ch1/2 (E+F1+Shh). (ZIP) [file pone.0239995.s007.zip › S6_File/EF10_12_ch2.jpg]

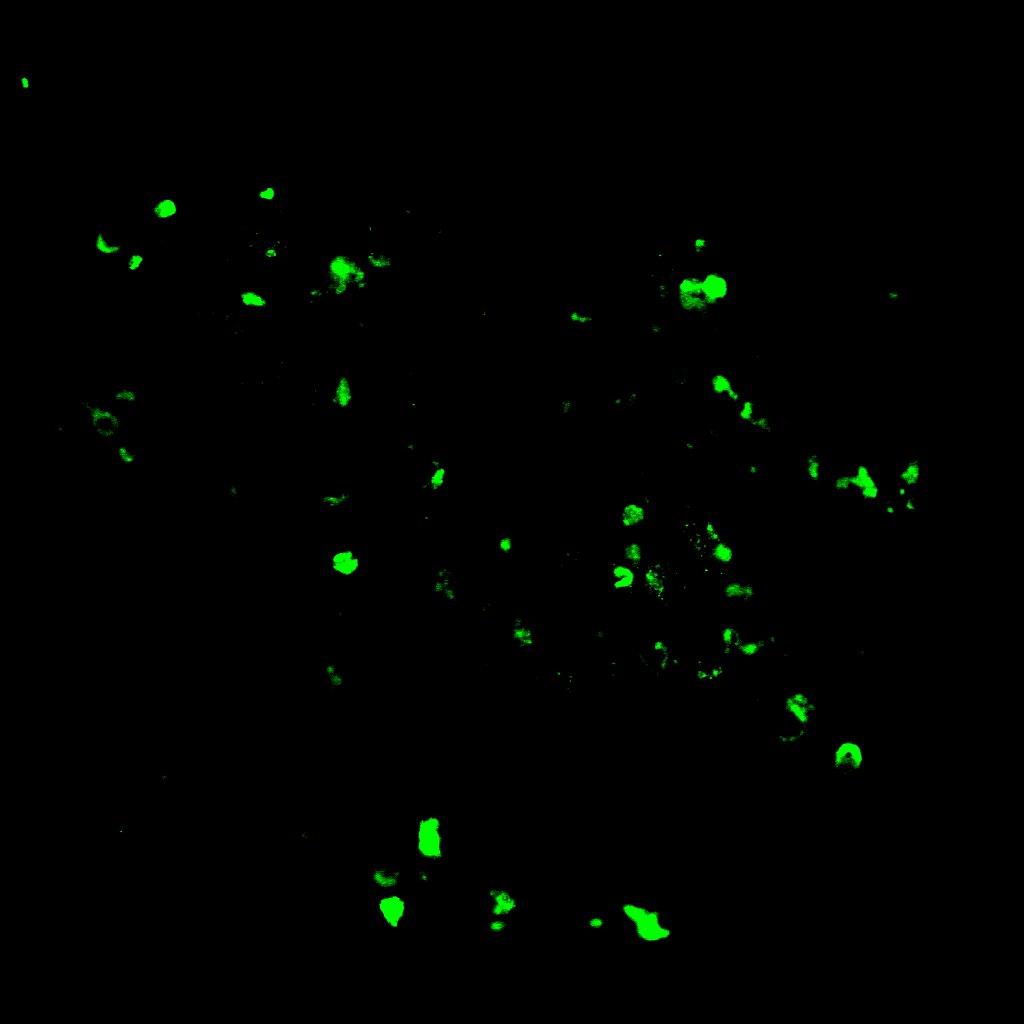

Supplement: S6 File — Representative images in the revised S2C Fig were generated from raw files woGF_4_ch1/2 (W/O GF); EF10cyc_4_ch1/2 (E+F10+Cyc); EF1shh_7_ch1/2 (E+F1+Shh). (ZIP) [file pone.0239995.s007.zip › S6_File/EF10_2_ch1.jpg]

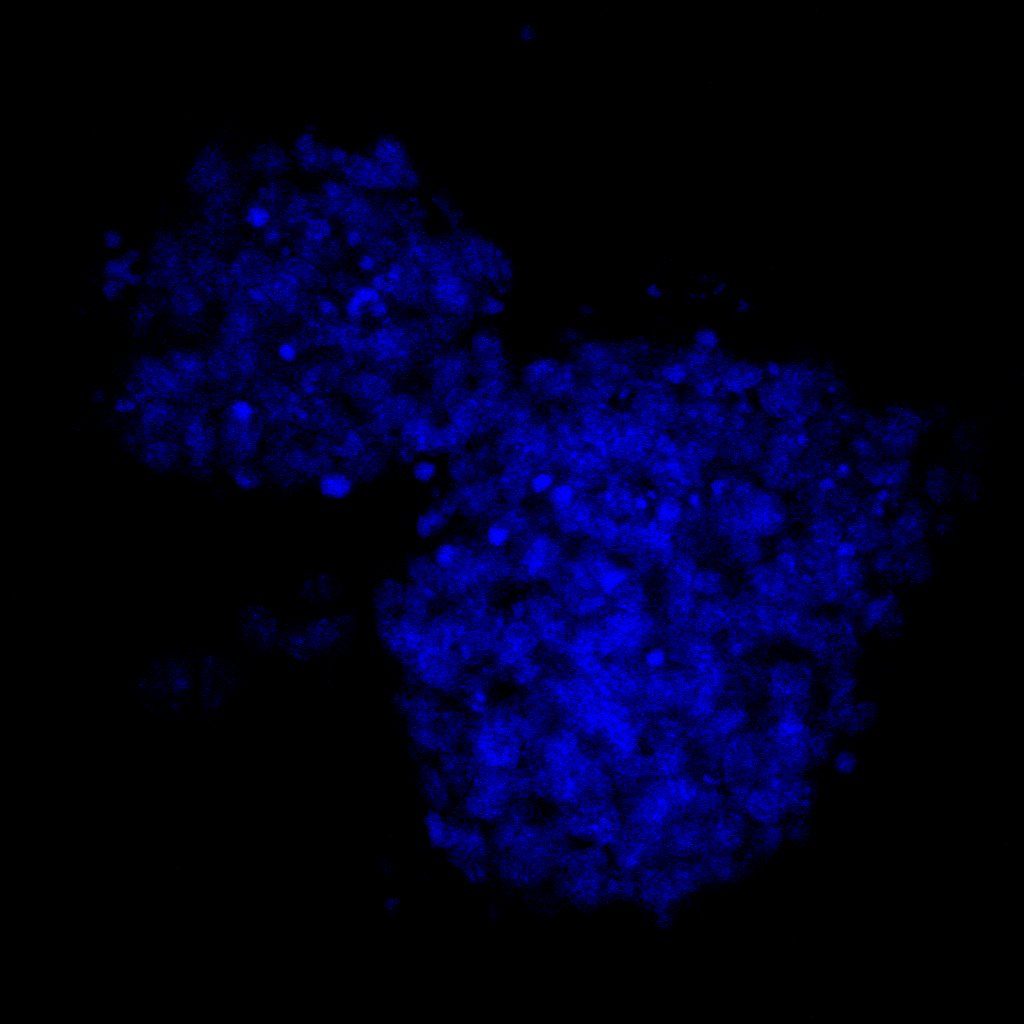

Supplement: S6 File — Representative images in the revised S2C Fig were generated from raw files woGF_4_ch1/2 (W/O GF); EF10cyc_4_ch1/2 (E+F10+Cyc); EF1shh_7_ch1/2 (E+F1+Shh). (ZIP) [file pone.0239995.s007.zip › S6_File/EF10_2_ch2.jpg]

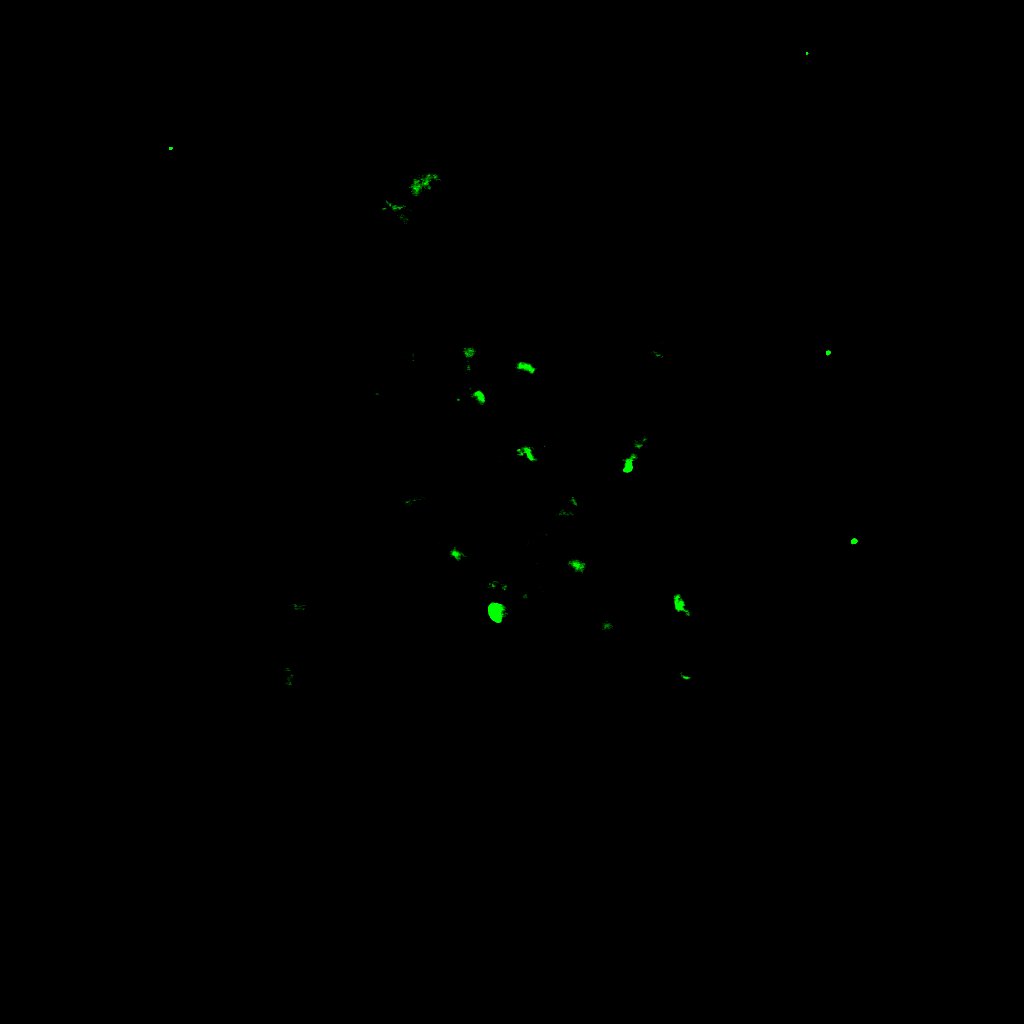

Supplement: S6 File — Representative images in the revised S2C Fig were generated from raw files woGF_4_ch1/2 (W/O GF); EF10cyc_4_ch1/2 (E+F10+Cyc); EF1shh_7_ch1/2 (E+F1+Shh). (ZIP) [file pone.0239995.s007.zip › S6_File/EF10_3_ch1.jpg]

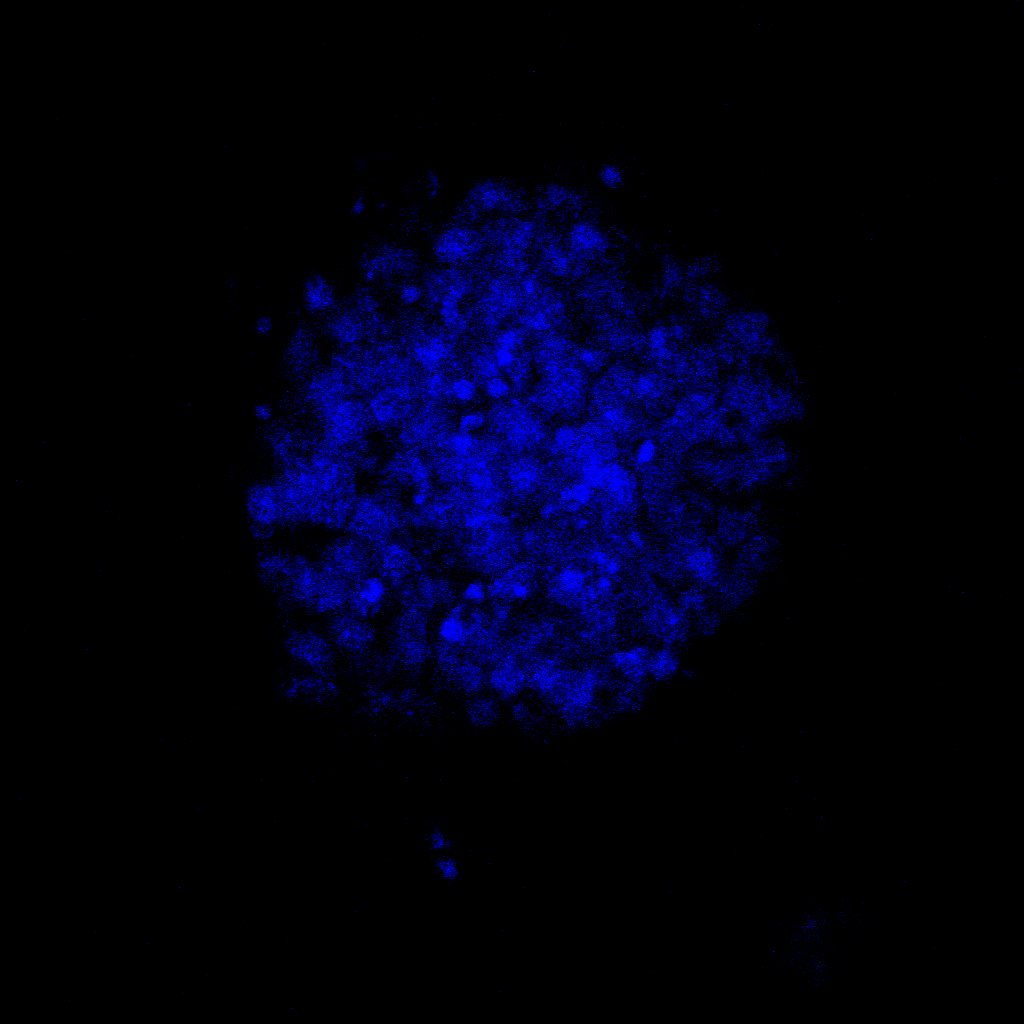

Supplement: S6 File — Representative images in the revised S2C Fig were generated from raw files woGF_4_ch1/2 (W/O GF); EF10cyc_4_ch1/2 (E+F10+Cyc); EF1shh_7_ch1/2 (E+F1+Shh). (ZIP) [file pone.0239995.s007.zip › S6_File/EF10_3_ch2.jpg]

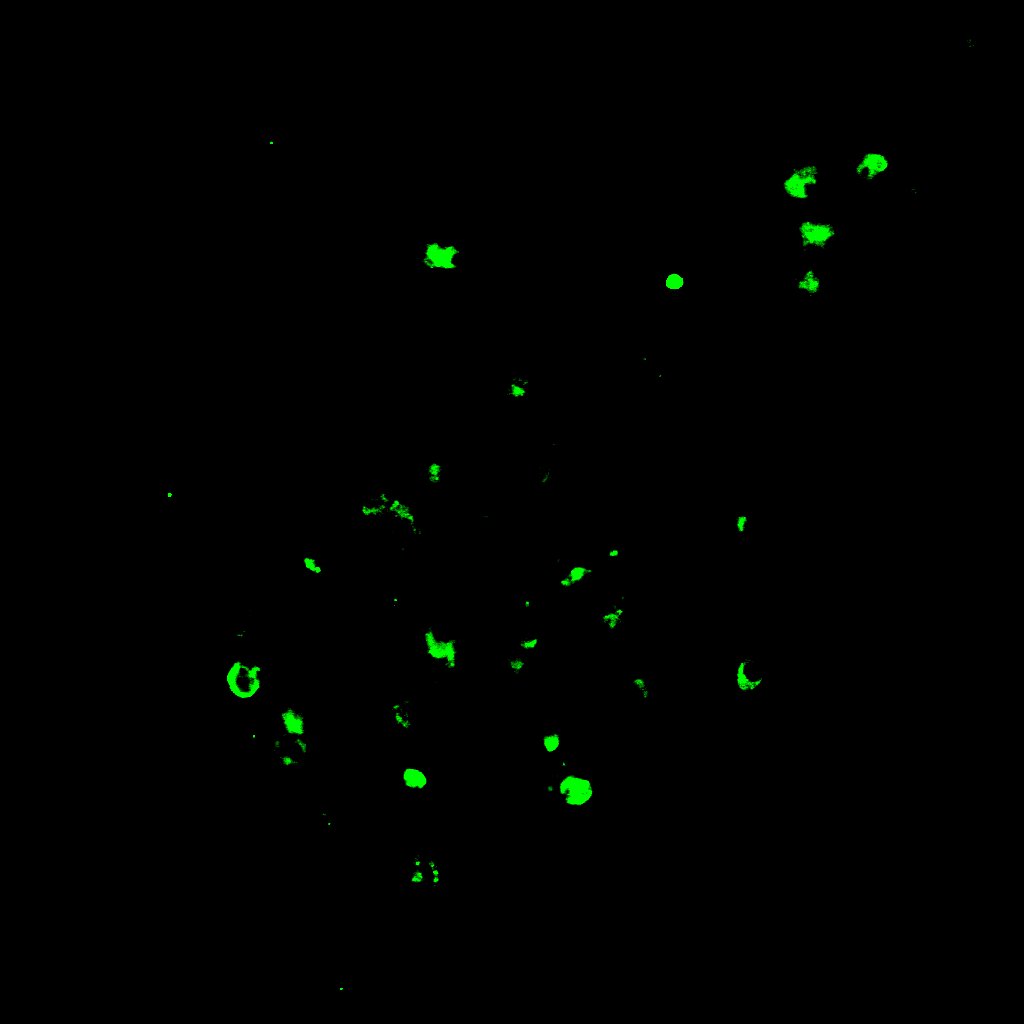

Supplement: S6 File — Representative images in the revised S2C Fig were generated from raw files woGF_4_ch1/2 (W/O GF); EF10cyc_4_ch1/2 (E+F10+Cyc); EF1shh_7_ch1/2 (E+F1+Shh). (ZIP) [file pone.0239995.s007.zip › S6_File/EF10_4_ch1.jpg]

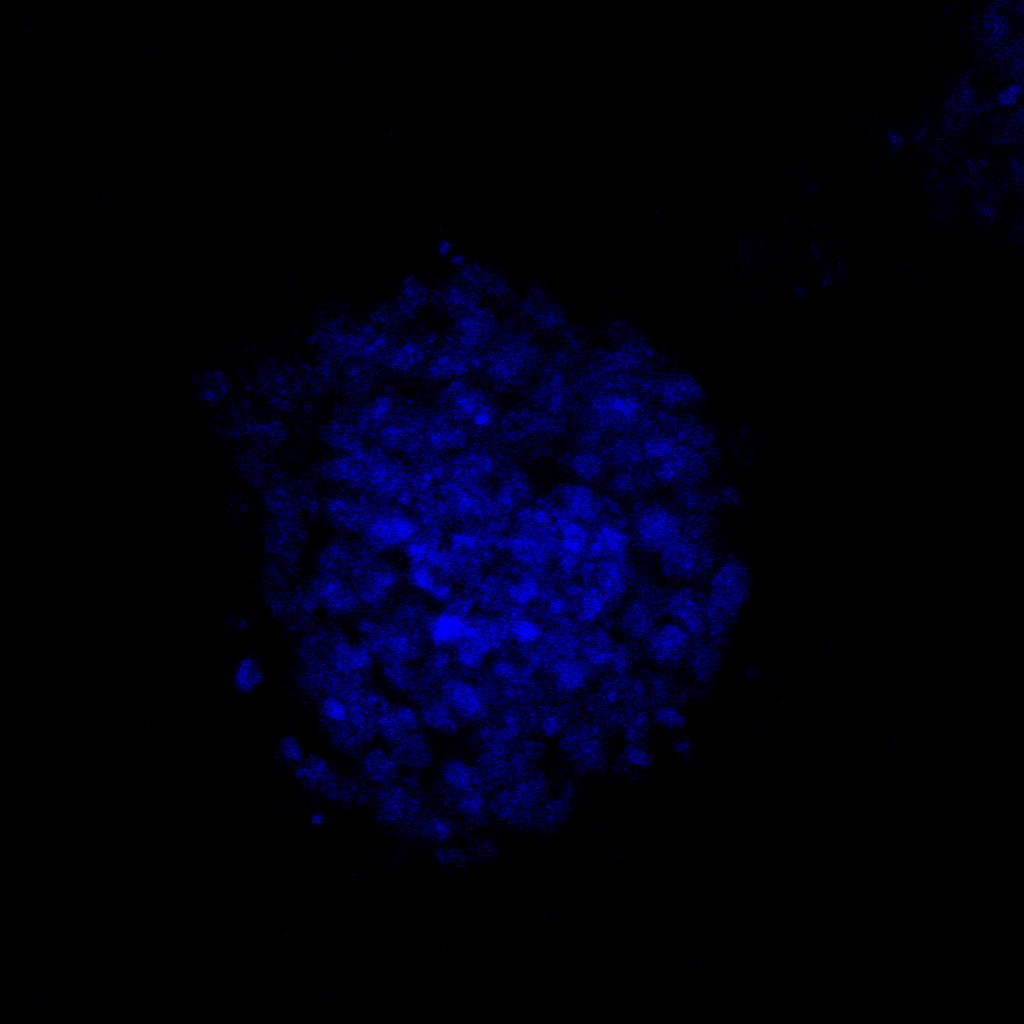

Supplement: S6 File — Representative images in the revised S2C Fig were generated from raw files woGF_4_ch1/2 (W/O GF); EF10cyc_4_ch1/2 (E+F10+Cyc); EF1shh_7_ch1/2 (E+F1+Shh). (ZIP) [file pone.0239995.s007.zip › S6_File/EF10_4_ch2.jpg]

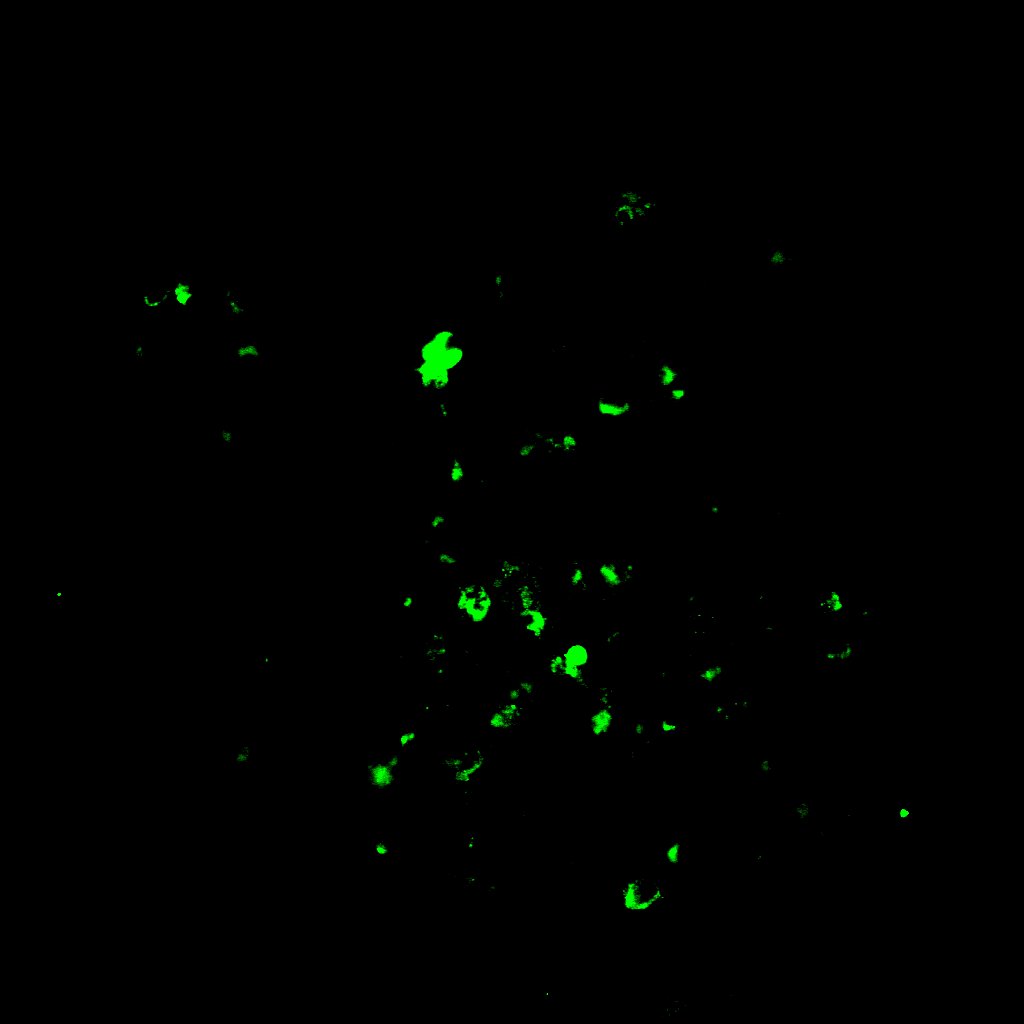

Supplement: S6 File — Representative images in the revised S2C Fig were generated from raw files woGF_4_ch1/2 (W/O GF); EF10cyc_4_ch1/2 (E+F10+Cyc); EF1shh_7_ch1/2 (E+F1+Shh). (ZIP) [file pone.0239995.s007.zip › S6_File/EF10_5_ch1.jpg]

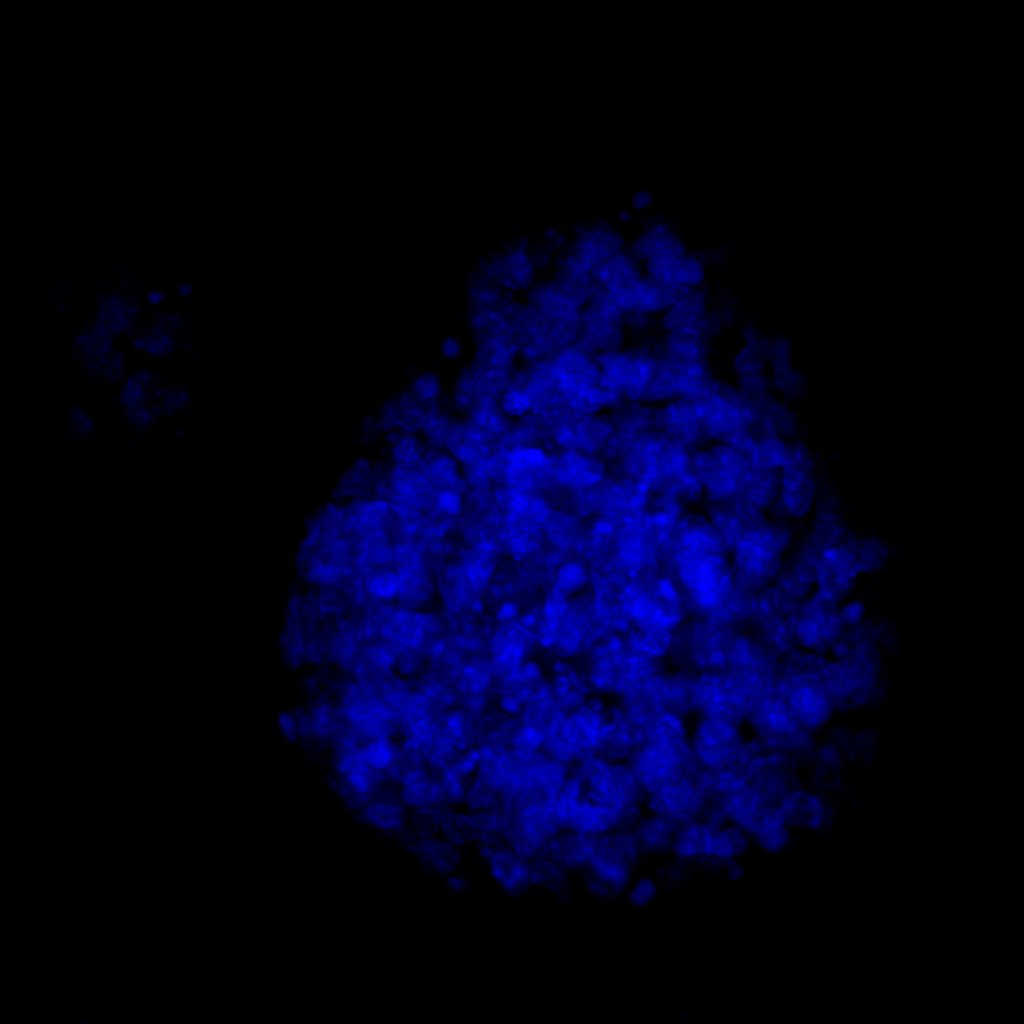

Supplement: S6 File — Representative images in the revised S2C Fig were generated from raw files woGF_4_ch1/2 (W/O GF); EF10cyc_4_ch1/2 (E+F10+Cyc); EF1shh_7_ch1/2 (E+F1+Shh). (ZIP) [file pone.0239995.s007.zip › S6_File/EF10_5_ch2.jpg]

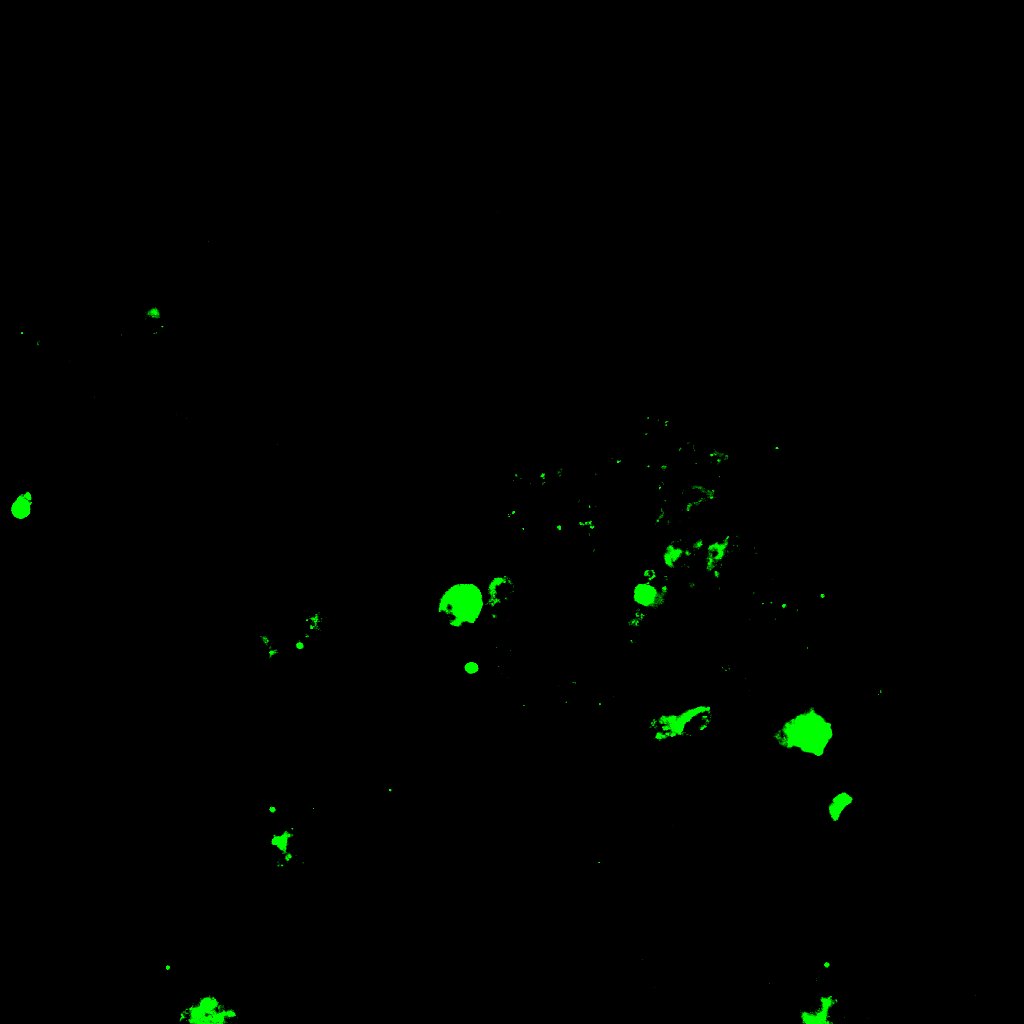

Supplement: S6 File — Representative images in the revised S2C Fig were generated from raw files woGF_4_ch1/2 (W/O GF); EF10cyc_4_ch1/2 (E+F10+Cyc); EF1shh_7_ch1/2 (E+F1+Shh). (ZIP) [file pone.0239995.s007.zip › S6_File/EF10_6_ch1.jpg]

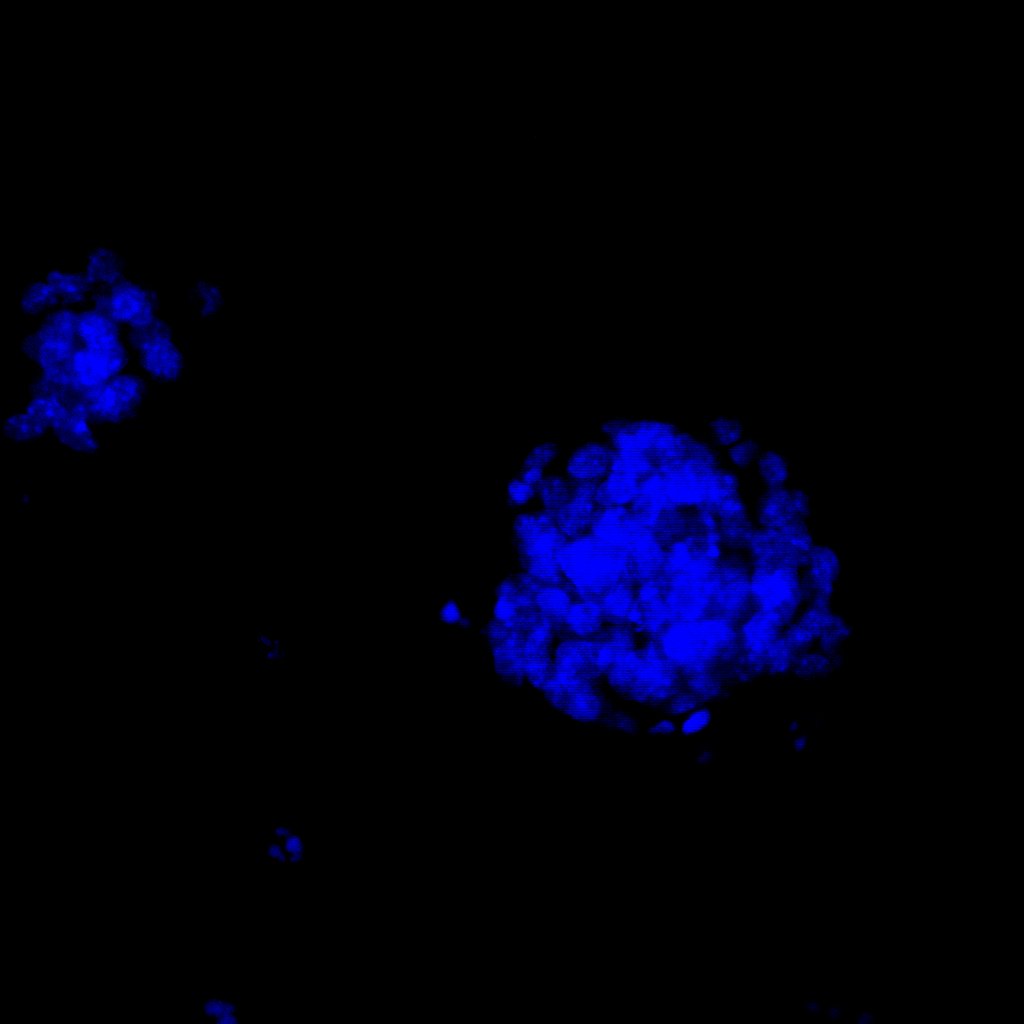

Supplement: S6 File — Representative images in the revised S2C Fig were generated from raw files woGF_4_ch1/2 (W/O GF); EF10cyc_4_ch1/2 (E+F10+Cyc); EF1shh_7_ch1/2 (E+F1+Shh). (ZIP) [file pone.0239995.s007.zip › S6_File/EF10_6_ch2.jpg]

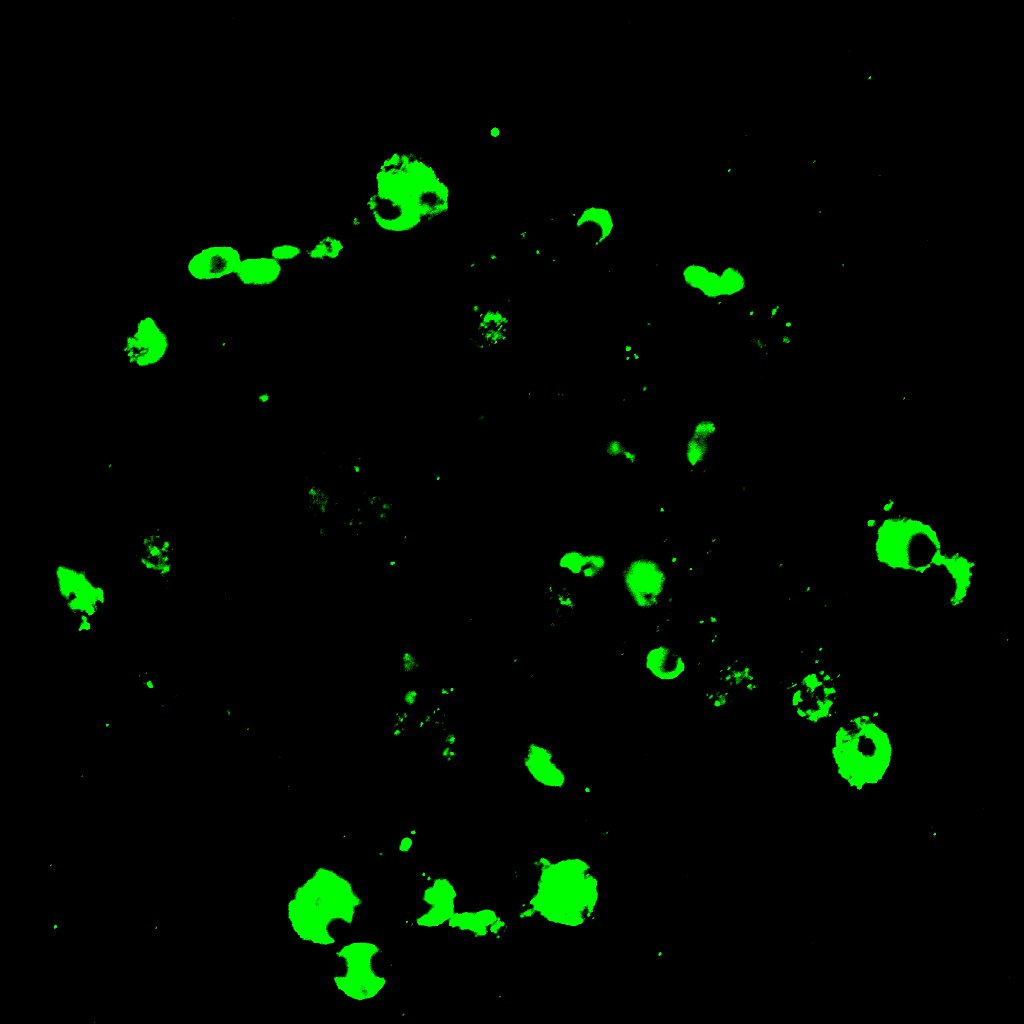

Supplement: S6 File — Representative images in the revised S2C Fig were generated from raw files woGF_4_ch1/2 (W/O GF); EF10cyc_4_ch1/2 (E+F10+Cyc); EF1shh_7_ch1/2 (E+F1+Shh). (ZIP) [file pone.0239995.s007.zip › S6_File/EF10_7_ch1.jpg]

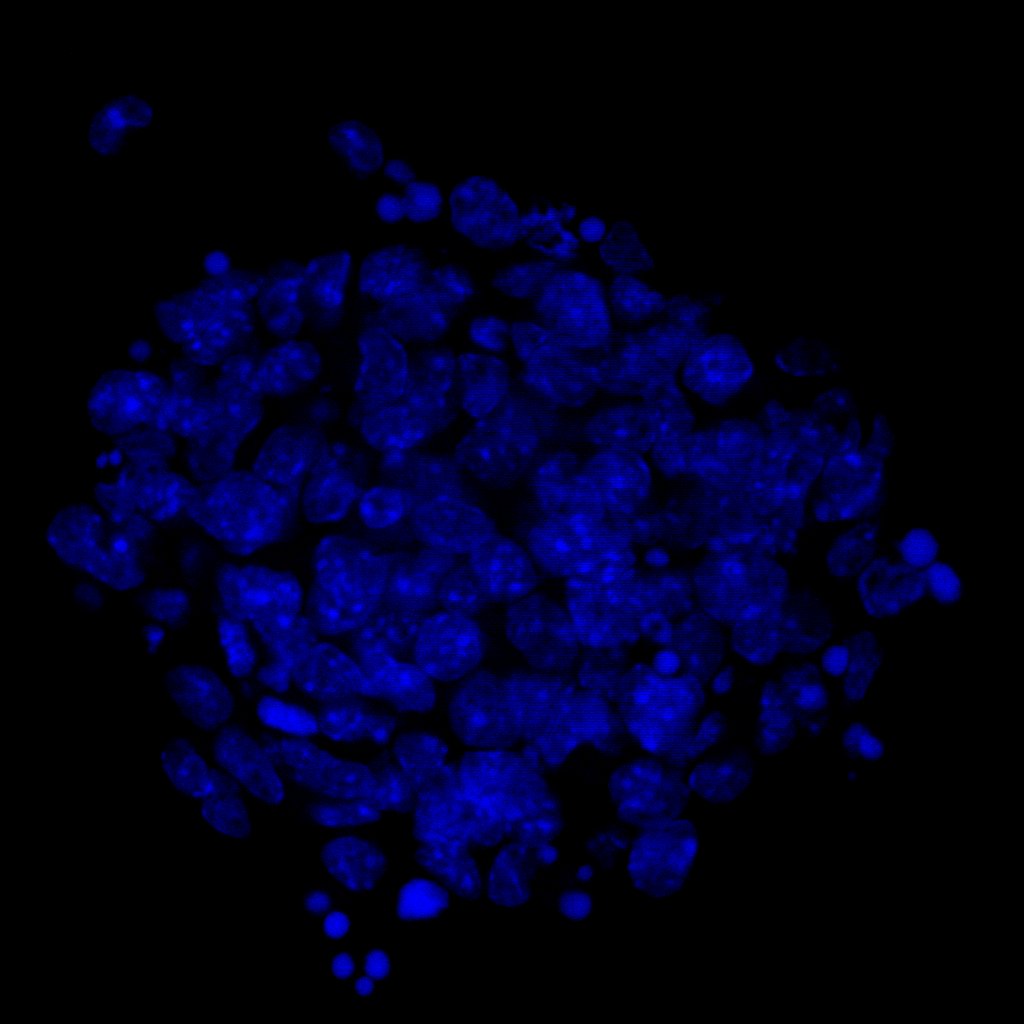

Supplement: S6 File — Representative images in the revised S2C Fig were generated from raw files woGF_4_ch1/2 (W/O GF); EF10cyc_4_ch1/2 (E+F10+Cyc); EF1shh_7_ch1/2 (E+F1+Shh). (ZIP) [file pone.0239995.s007.zip › S6_File/EF10_7_ch2.jpg]

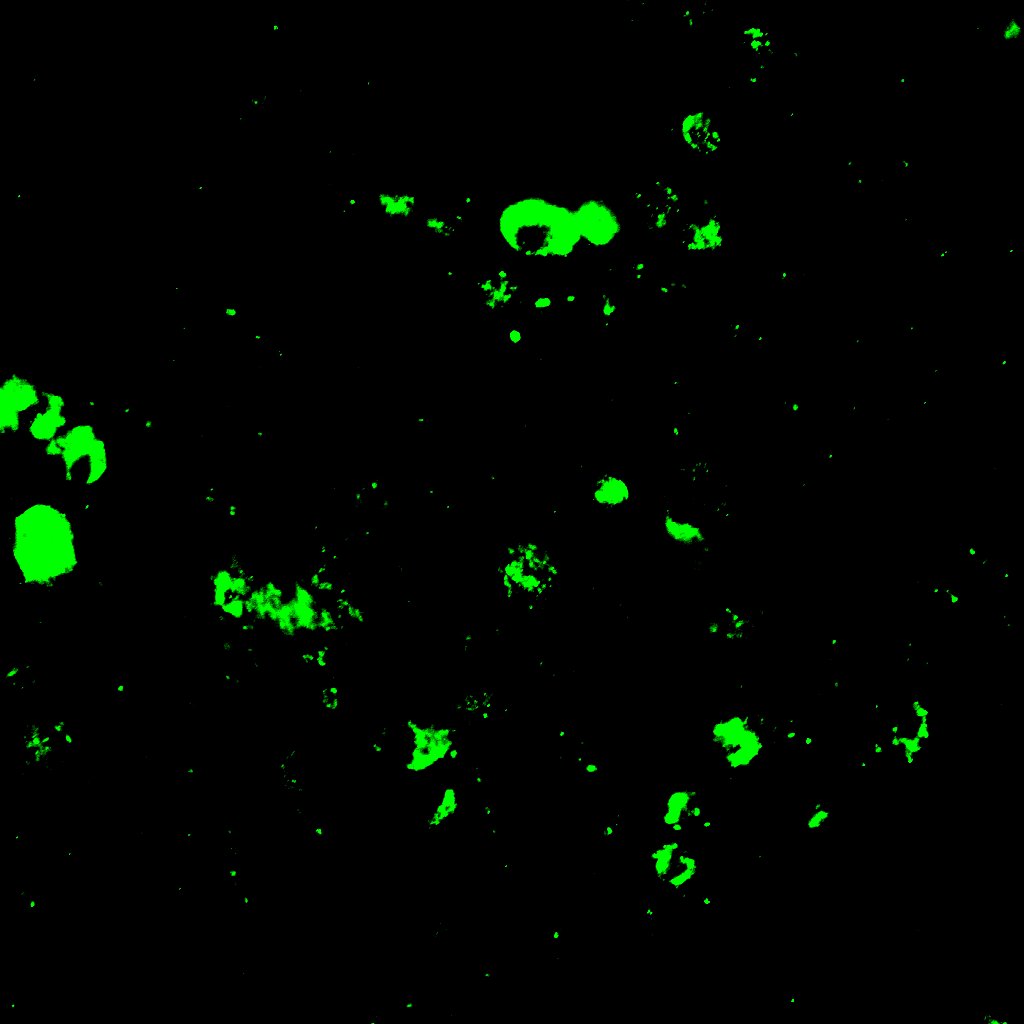

Supplement: S6 File — Representative images in the revised S2C Fig were generated from raw files woGF_4_ch1/2 (W/O GF); EF10cyc_4_ch1/2 (E+F10+Cyc); EF1shh_7_ch1/2 (E+F1+Shh). (ZIP) [file pone.0239995.s007.zip › S6_File/EF10_8_ch1.jpg]

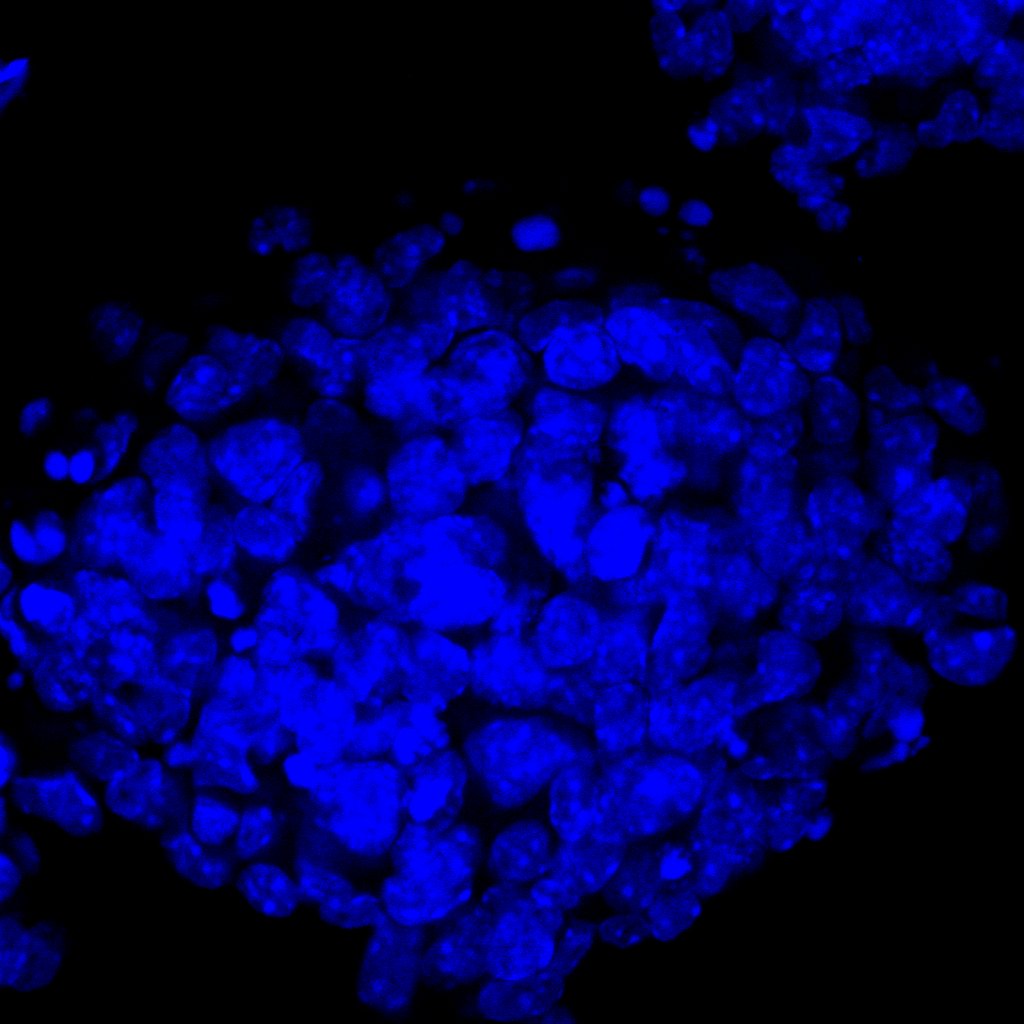

Supplement: S6 File — Representative images in the revised S2C Fig were generated from raw files woGF_4_ch1/2 (W/O GF); EF10cyc_4_ch1/2 (E+F10+Cyc); EF1shh_7_ch1/2 (E+F1+Shh). (ZIP) [file pone.0239995.s007.zip › S6_File/EF10_8_ch2.jpg]

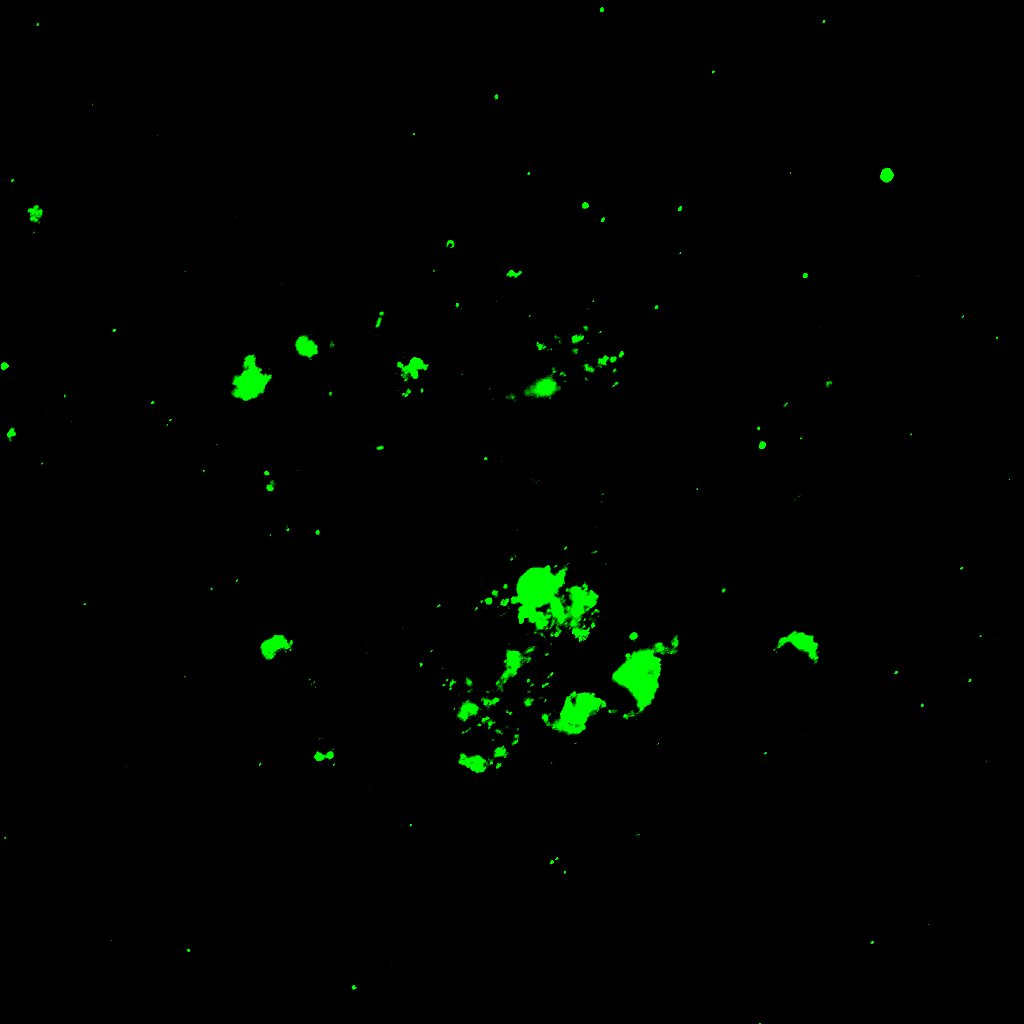

Supplement: S6 File — Representative images in the revised S2C Fig were generated from raw files woGF_4_ch1/2 (W/O GF); EF10cyc_4_ch1/2 (E+F10+Cyc); EF1shh_7_ch1/2 (E+F1+Shh). (ZIP) [file pone.0239995.s007.zip › S6_File/EF10_9_ch1.jpg]

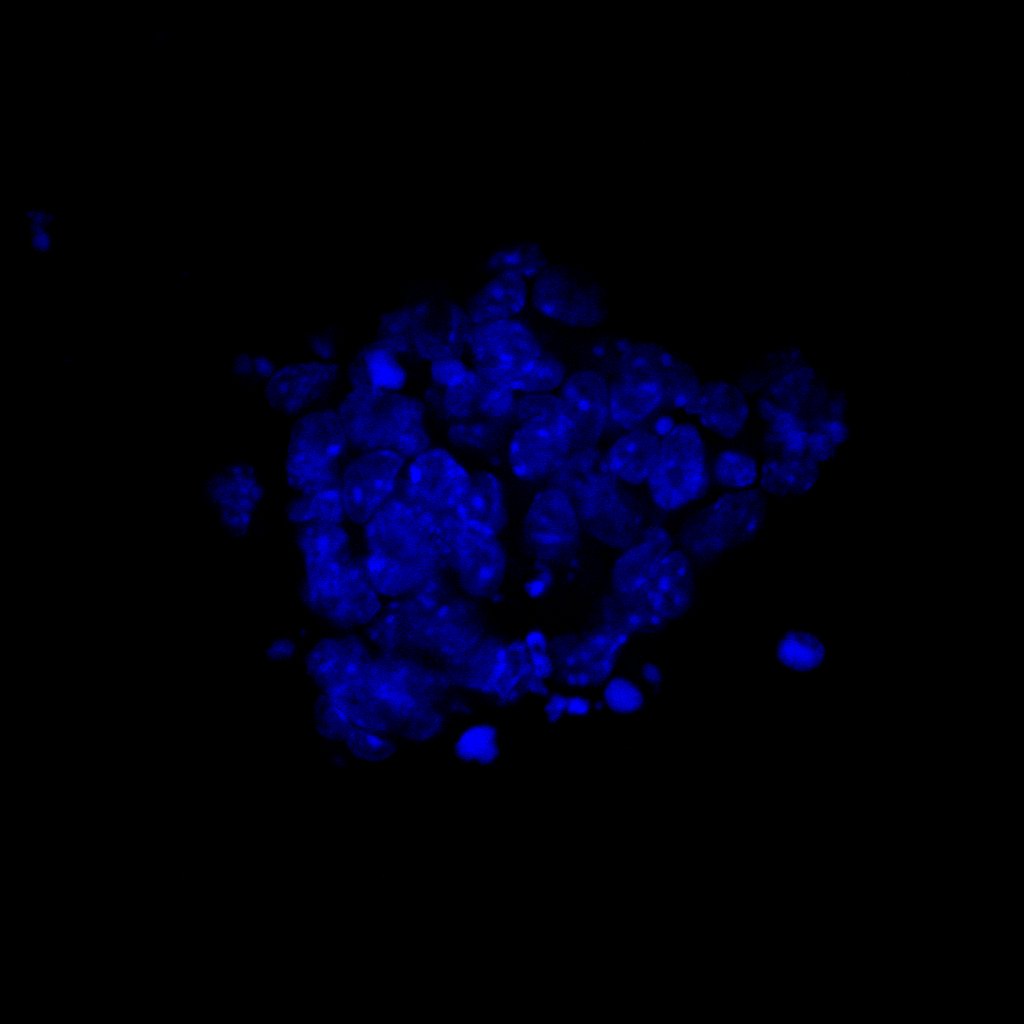

Supplement: S6 File — Representative images in the revised S2C Fig were generated from raw files woGF_4_ch1/2 (W/O GF); EF10cyc_4_ch1/2 (E+F10+Cyc); EF1shh_7_ch1/2 (E+F1+Shh). (ZIP) [file pone.0239995.s007.zip › S6_File/EF10_9_ch2.jpg]

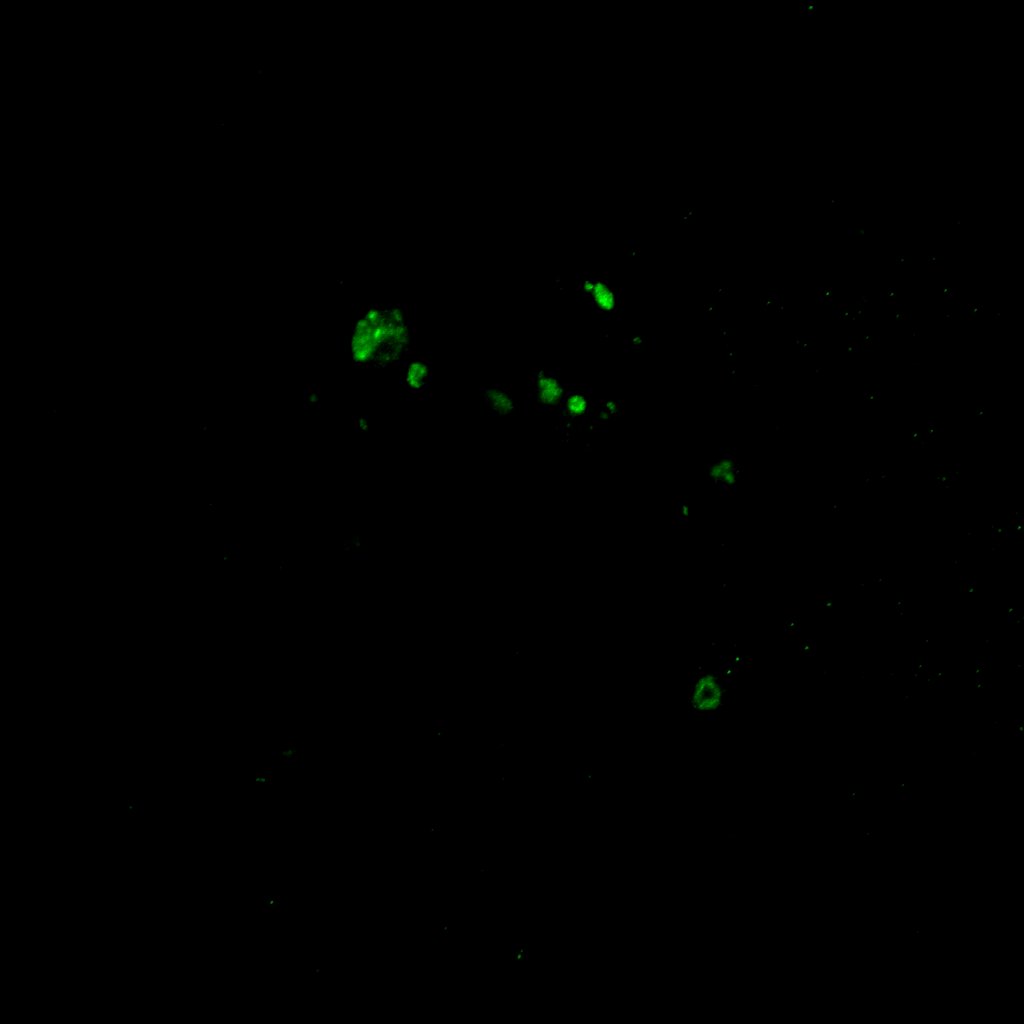

Supplement: S6 File — Representative images in the revised S2C Fig were generated from raw files woGF_4_ch1/2 (W/O GF); EF10cyc_4_ch1/2 (E+F10+Cyc); EF1shh_7_ch1/2 (E+F1+Shh). (ZIP) [file pone.0239995.s007.zip › S6_File/EF10cyc_1_ch1.jpg]

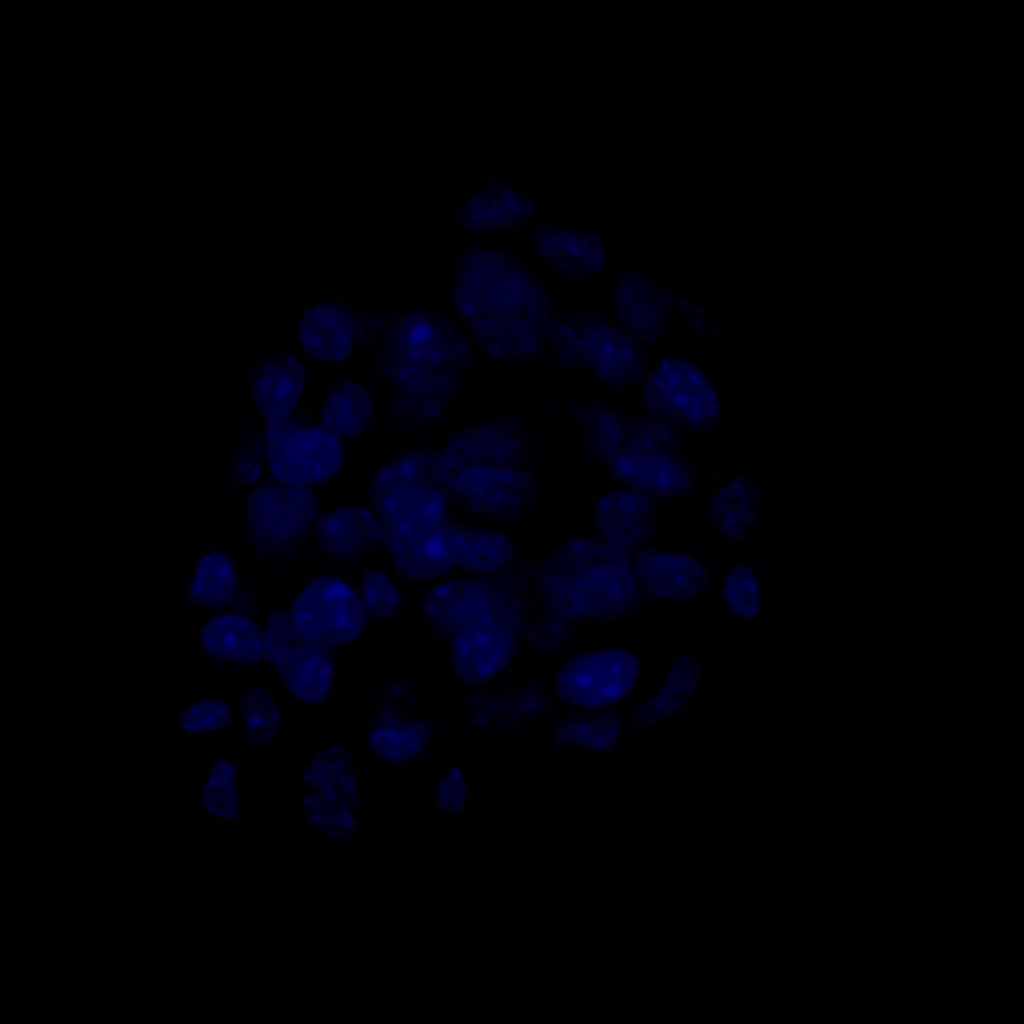

Supplement: S6 File — Representative images in the revised S2C Fig were generated from raw files woGF_4_ch1/2 (W/O GF); EF10cyc_4_ch1/2 (E+F10+Cyc); EF1shh_7_ch1/2 (E+F1+Shh). (ZIP) [file pone.0239995.s007.zip › S6_File/EF10cyc_1_ch2.jpg]

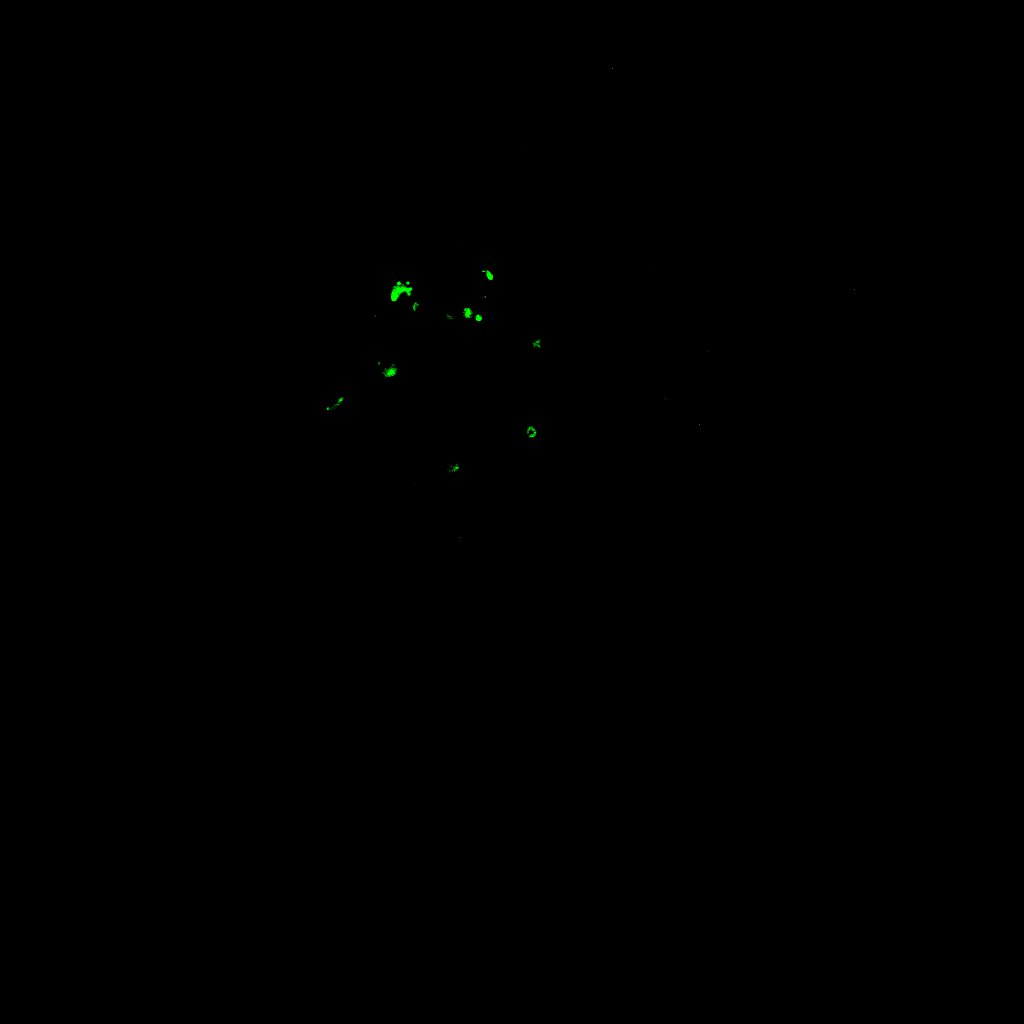

Supplement: S6 File — Representative images in the revised S2C Fig were generated from raw files woGF_4_ch1/2 (W/O GF); EF10cyc_4_ch1/2 (E+F10+Cyc); EF1shh_7_ch1/2 (E+F1+Shh). (ZIP) [file pone.0239995.s007.zip › S6_File/EF10cyc_2_ch1.jpg]

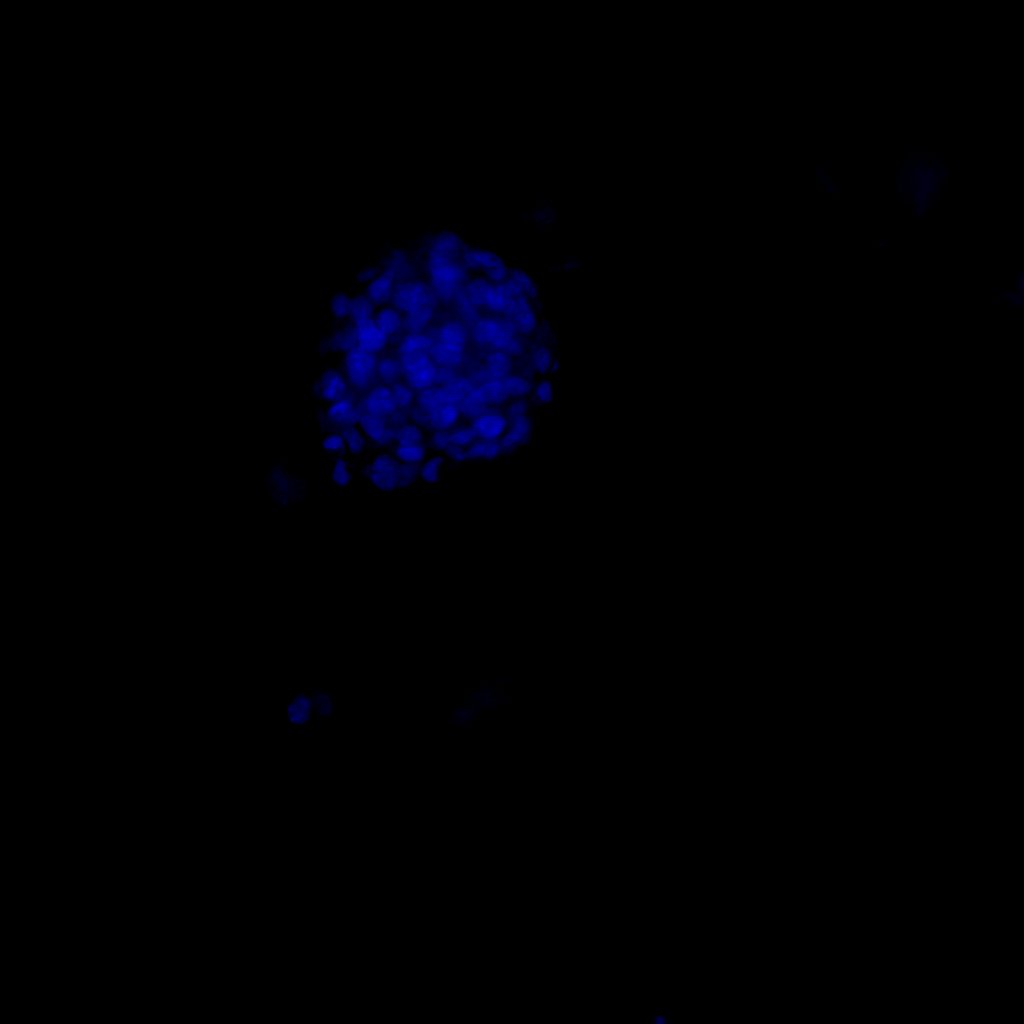

Supplement: S6 File — Representative images in the revised S2C Fig were generated from raw files woGF_4_ch1/2 (W/O GF); EF10cyc_4_ch1/2 (E+F10+Cyc); EF1shh_7_ch1/2 (E+F1+Shh). (ZIP) [file pone.0239995.s007.zip › S6_File/EF10cyc_2_ch2.jpg]

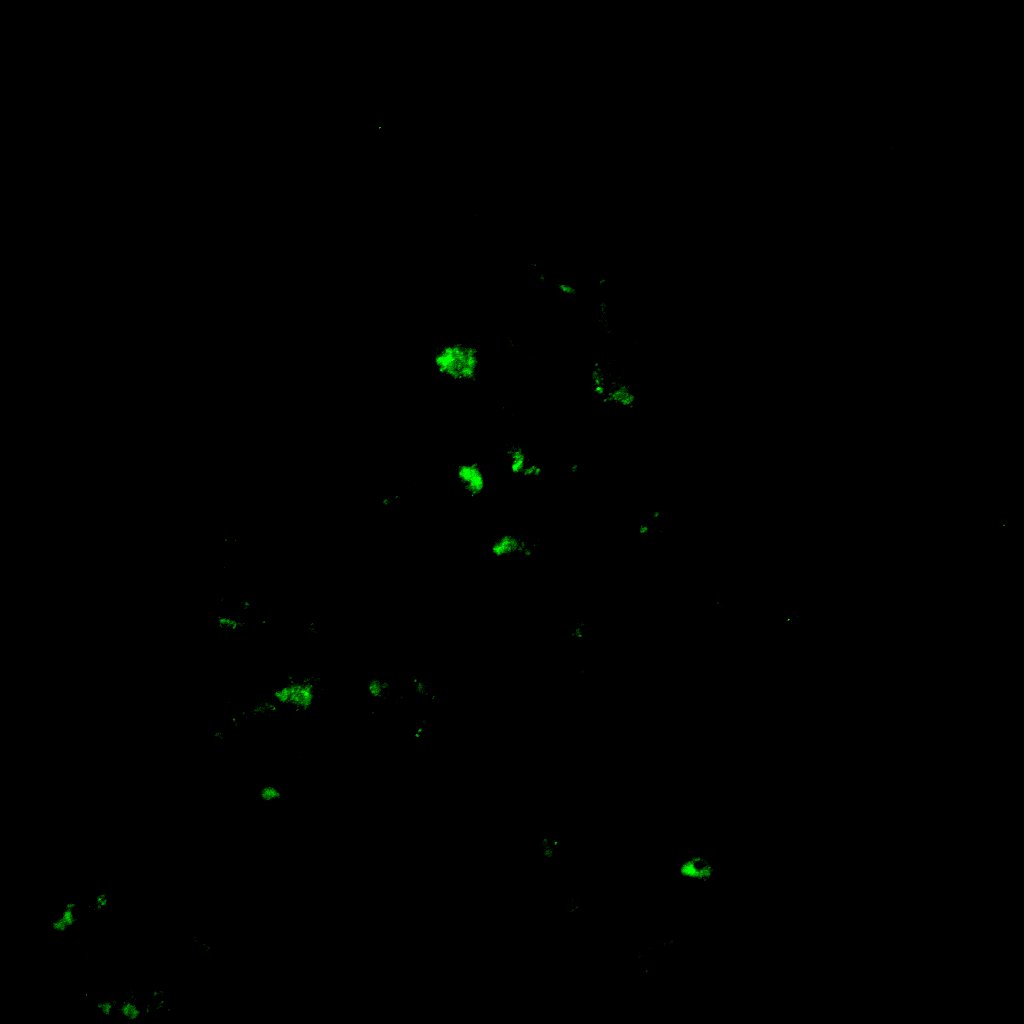

Supplement: S6 File — Representative images in the revised S2C Fig were generated from raw files woGF_4_ch1/2 (W/O GF); EF10cyc_4_ch1/2 (E+F10+Cyc); EF1shh_7_ch1/2 (E+F1+Shh). (ZIP) [file pone.0239995.s007.zip › S6_File/EF10cyc_3_ch1.jpg]

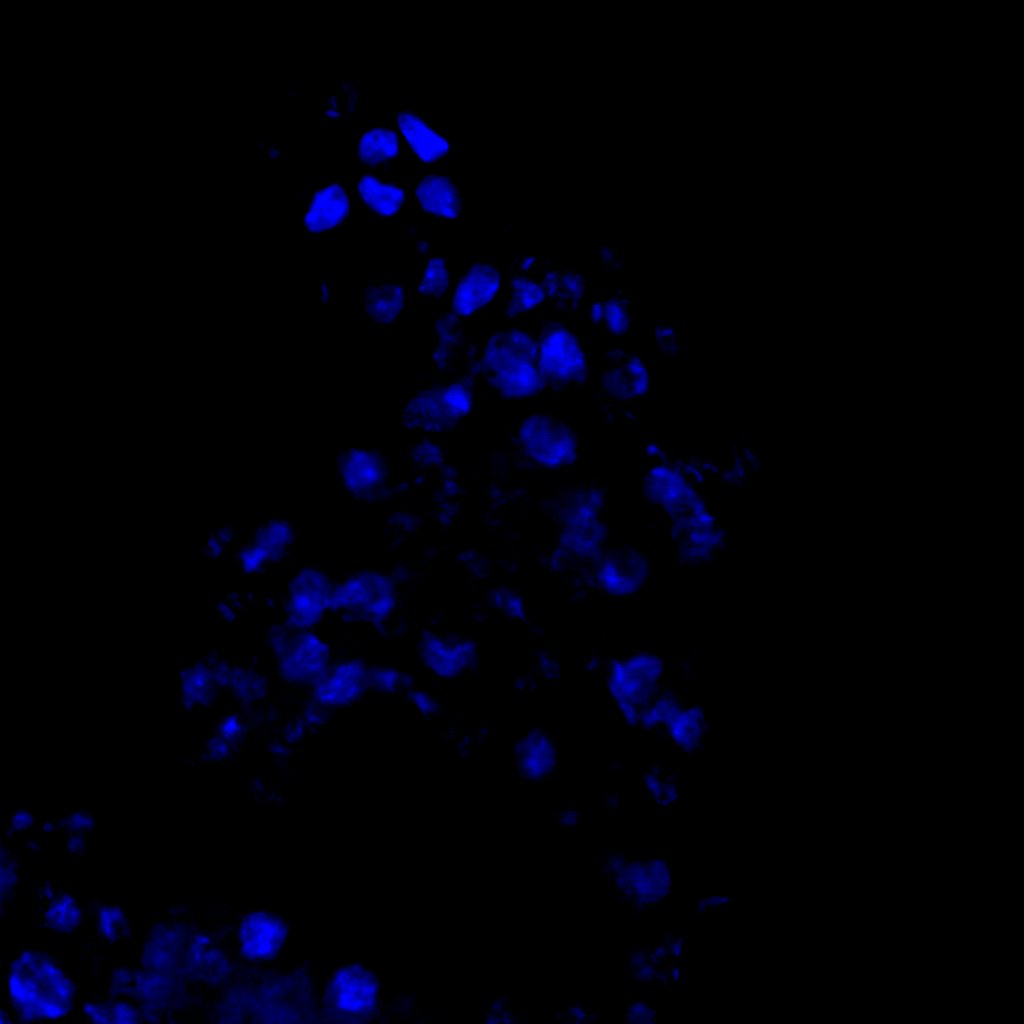

Supplement: S6 File — Representative images in the revised S2C Fig were generated from raw files woGF_4_ch1/2 (W/O GF); EF10cyc_4_ch1/2 (E+F10+Cyc); EF1shh_7_ch1/2 (E+F1+Shh). (ZIP) [file pone.0239995.s007.zip › S6_File/EF10cyc_3_ch2.jpg]

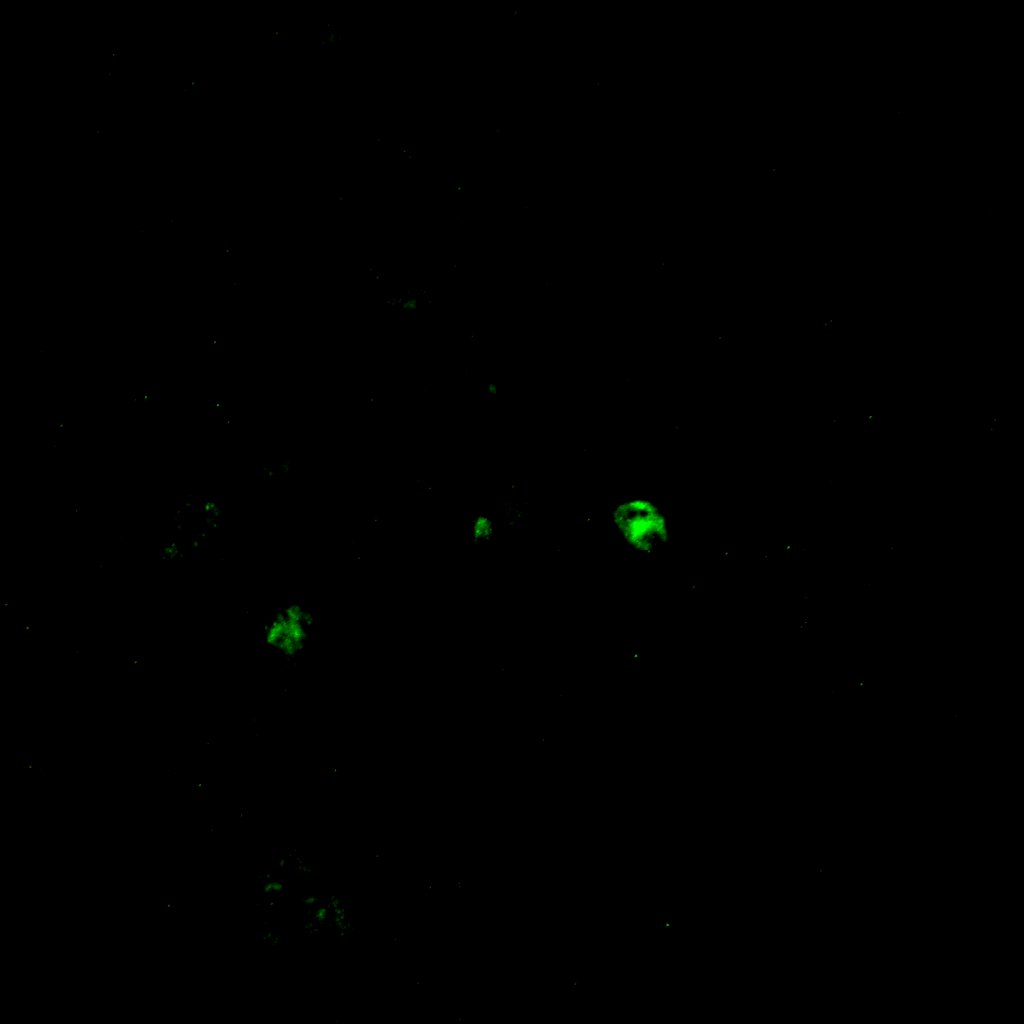

Supplement: S6 File — Representative images in the revised S2C Fig were generated from raw files woGF_4_ch1/2 (W/O GF); EF10cyc_4_ch1/2 (E+F10+Cyc); EF1shh_7_ch1/2 (E+F1+Shh). (ZIP) [file pone.0239995.s007.zip › S6_File/EF10cyc_4_ch1.jpg]

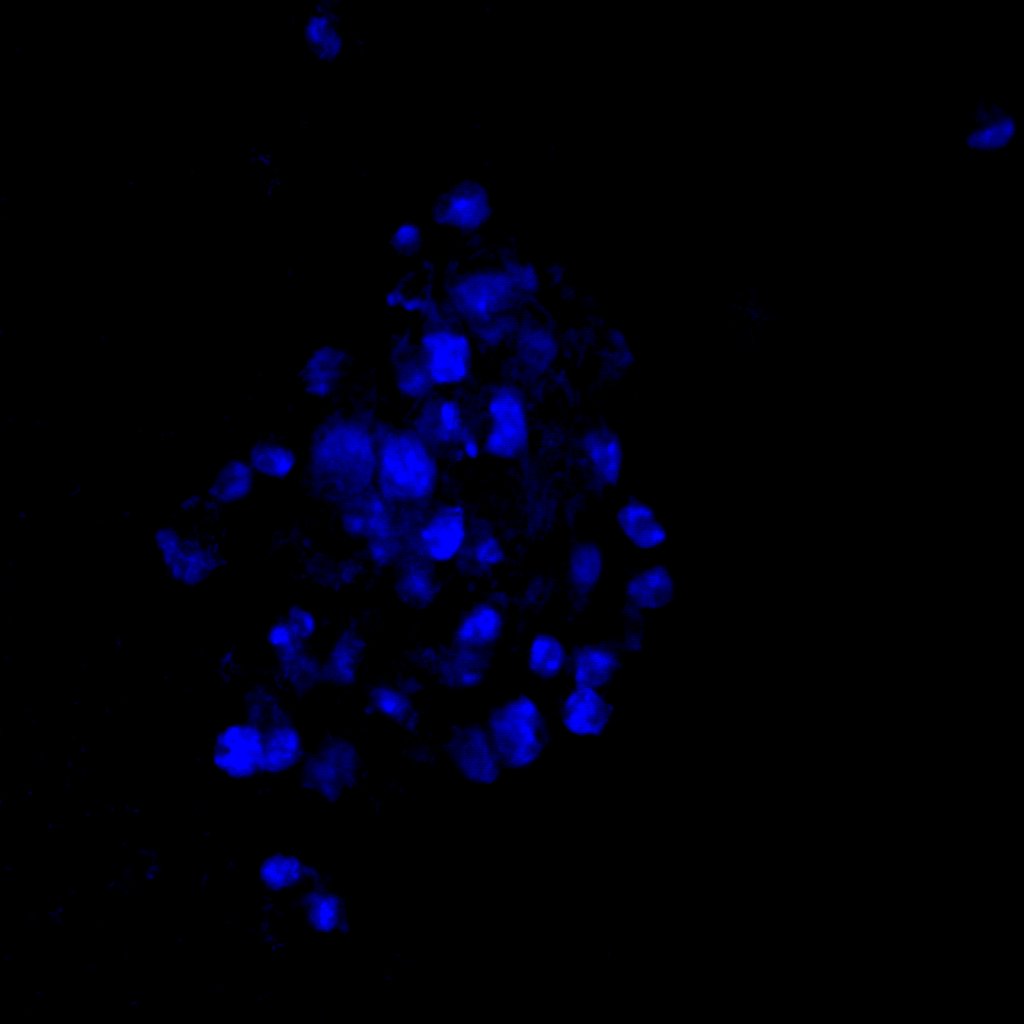

Supplement: S6 File — Representative images in the revised S2C Fig were generated from raw files woGF_4_ch1/2 (W/O GF); EF10cyc_4_ch1/2 (E+F10+Cyc); EF1shh_7_ch1/2 (E+F1+Shh). (ZIP) [file pone.0239995.s007.zip › S6_File/EF10cyc_4_ch2.jpg]

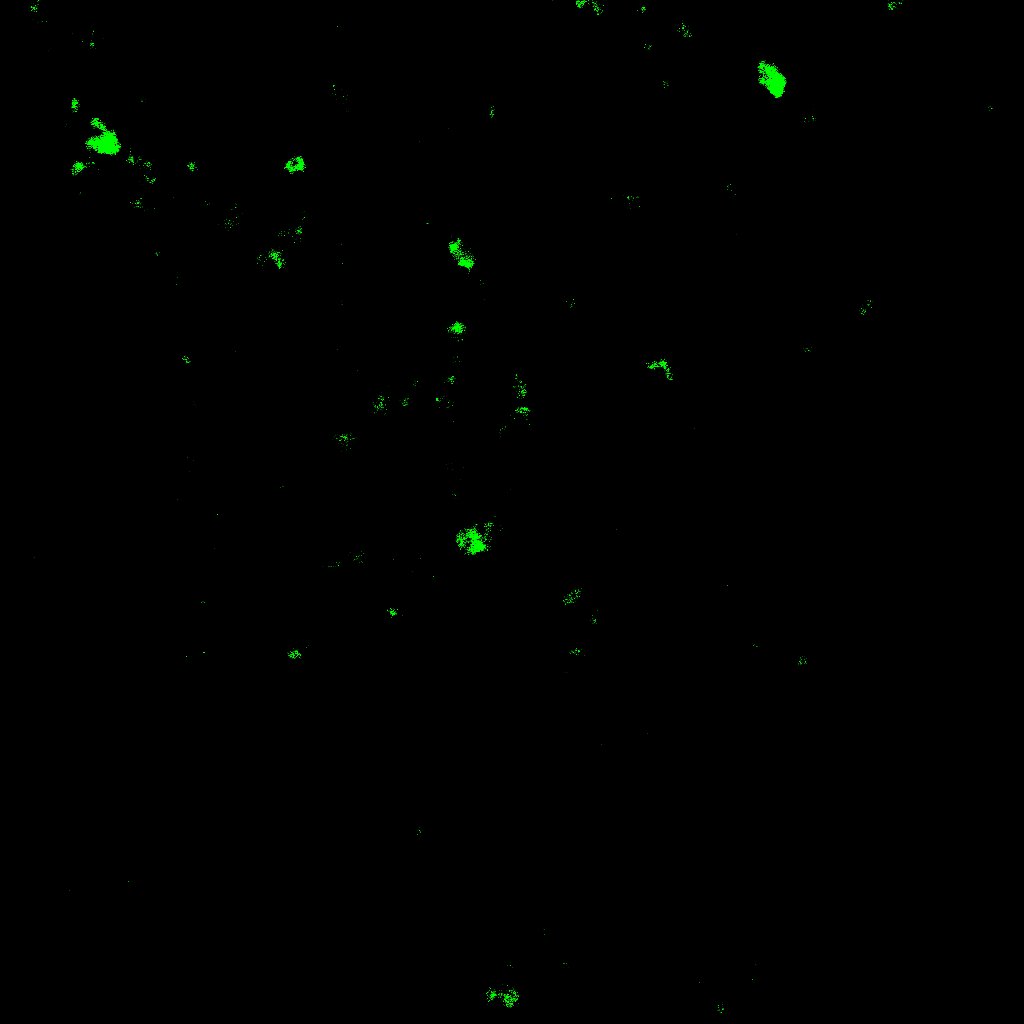

Supplement: S6 File — Representative images in the revised S2C Fig were generated from raw files woGF_4_ch1/2 (W/O GF); EF10cyc_4_ch1/2 (E+F10+Cyc); EF1shh_7_ch1/2 (E+F1+Shh). (ZIP) [file pone.0239995.s007.zip › S6_File/EF10cyc_5_ch1.jpg]

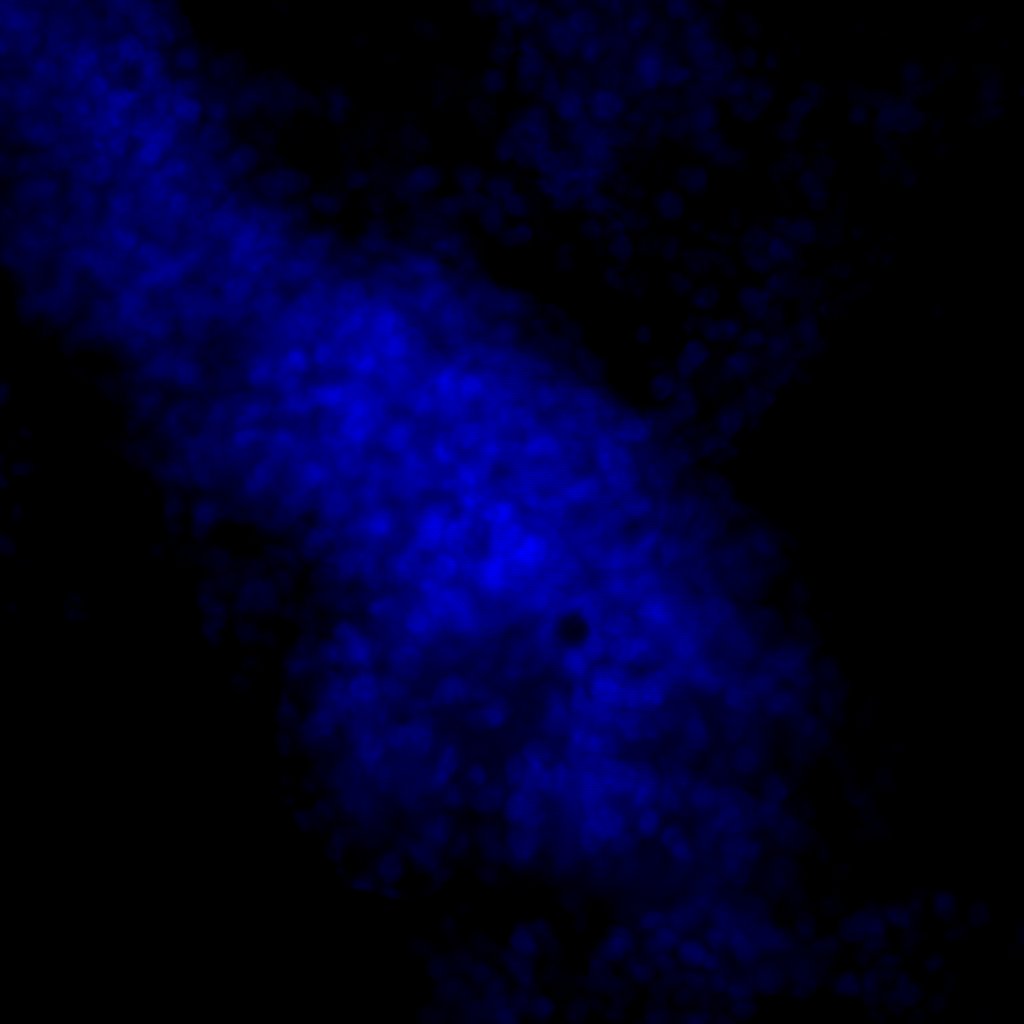

Supplement: S6 File — Representative images in the revised S2C Fig were generated from raw files woGF_4_ch1/2 (W/O GF); EF10cyc_4_ch1/2 (E+F10+Cyc); EF1shh_7_ch1/2 (E+F1+Shh). (ZIP) [file pone.0239995.s007.zip › S6_File/EF10cyc_5_ch2.jpg]

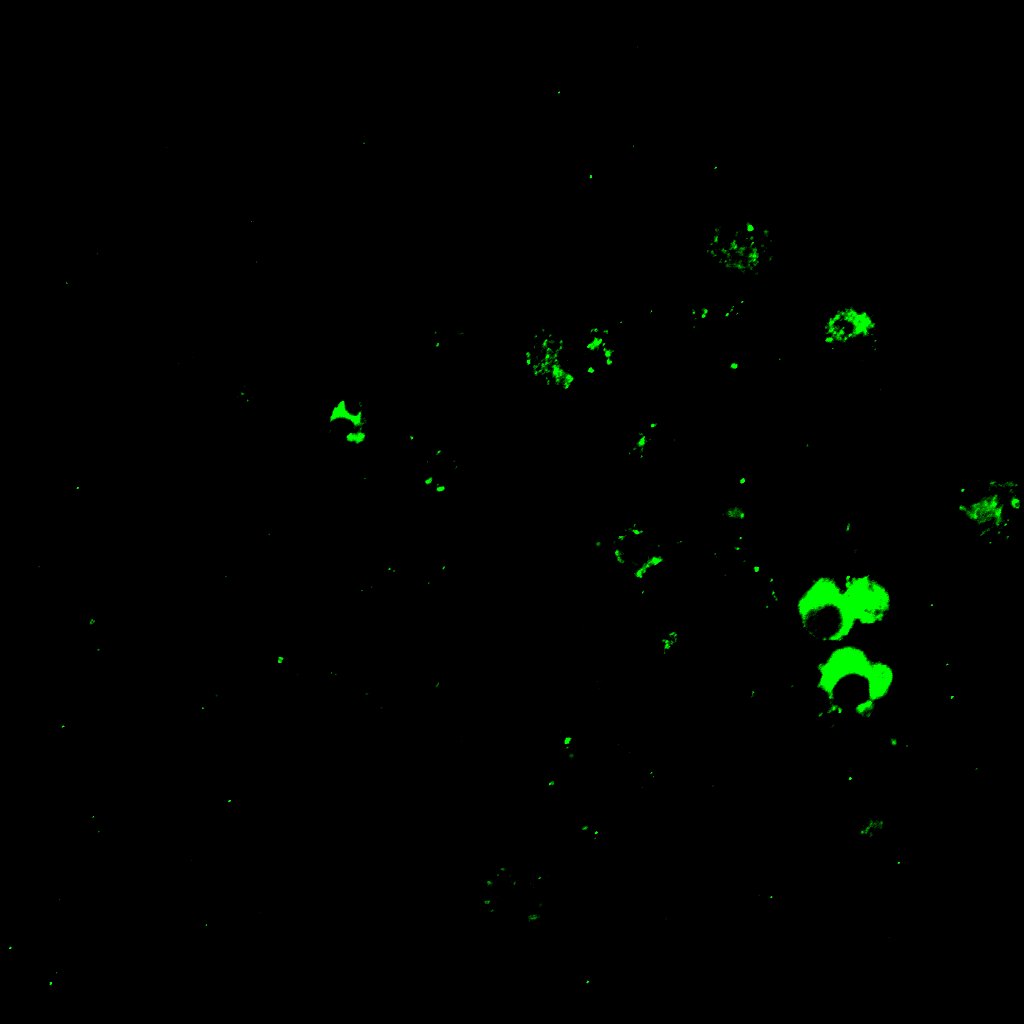

Supplement: S6 File — Representative images in the revised S2C Fig were generated from raw files woGF_4_ch1/2 (W/O GF); EF10cyc_4_ch1/2 (E+F10+Cyc); EF1shh_7_ch1/2 (E+F1+Shh). (ZIP) [file pone.0239995.s007.zip › S6_File/EF10cyc_6_ch1.jpg]

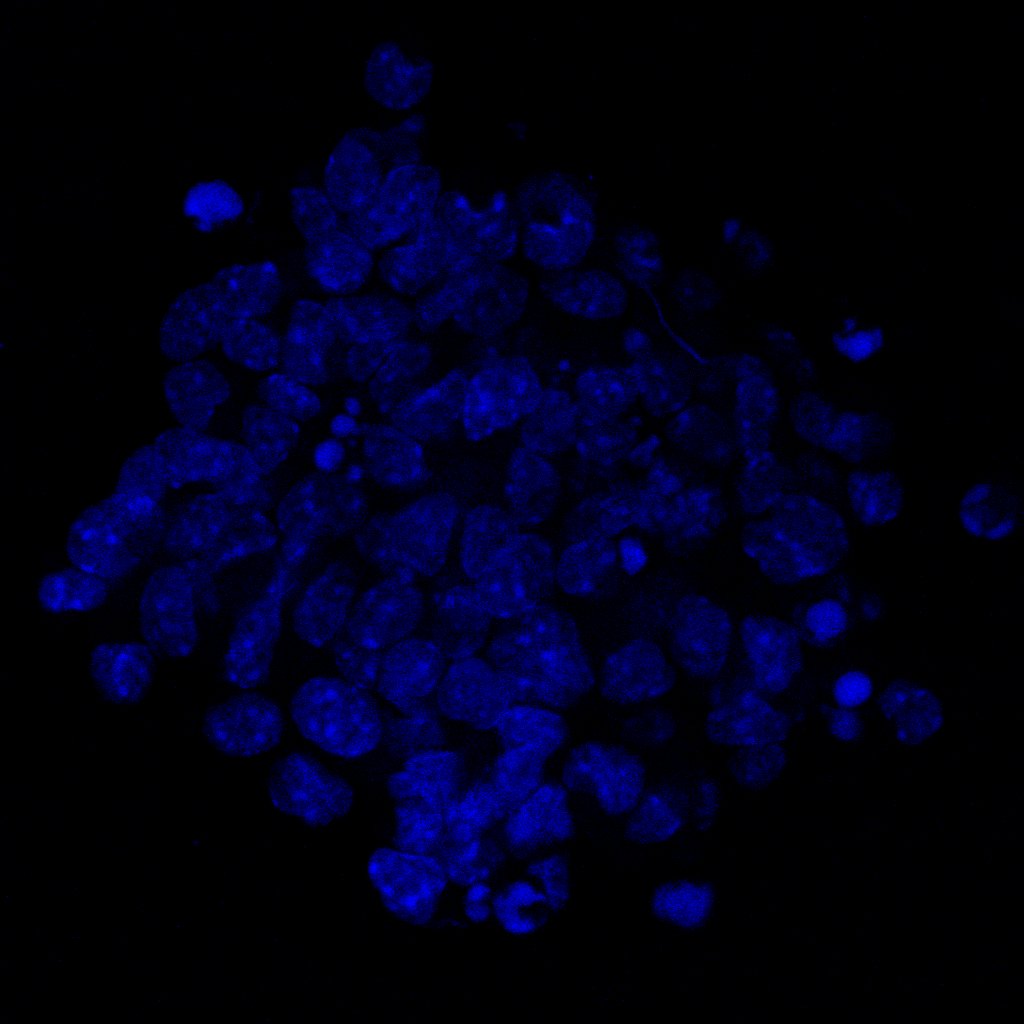

Supplement: S6 File — Representative images in the revised S2C Fig were generated from raw files woGF_4_ch1/2 (W/O GF); EF10cyc_4_ch1/2 (E+F10+Cyc); EF1shh_7_ch1/2 (E+F1+Shh). (ZIP) [file pone.0239995.s007.zip › S6_File/EF10cyc_6_ch2.jpg]

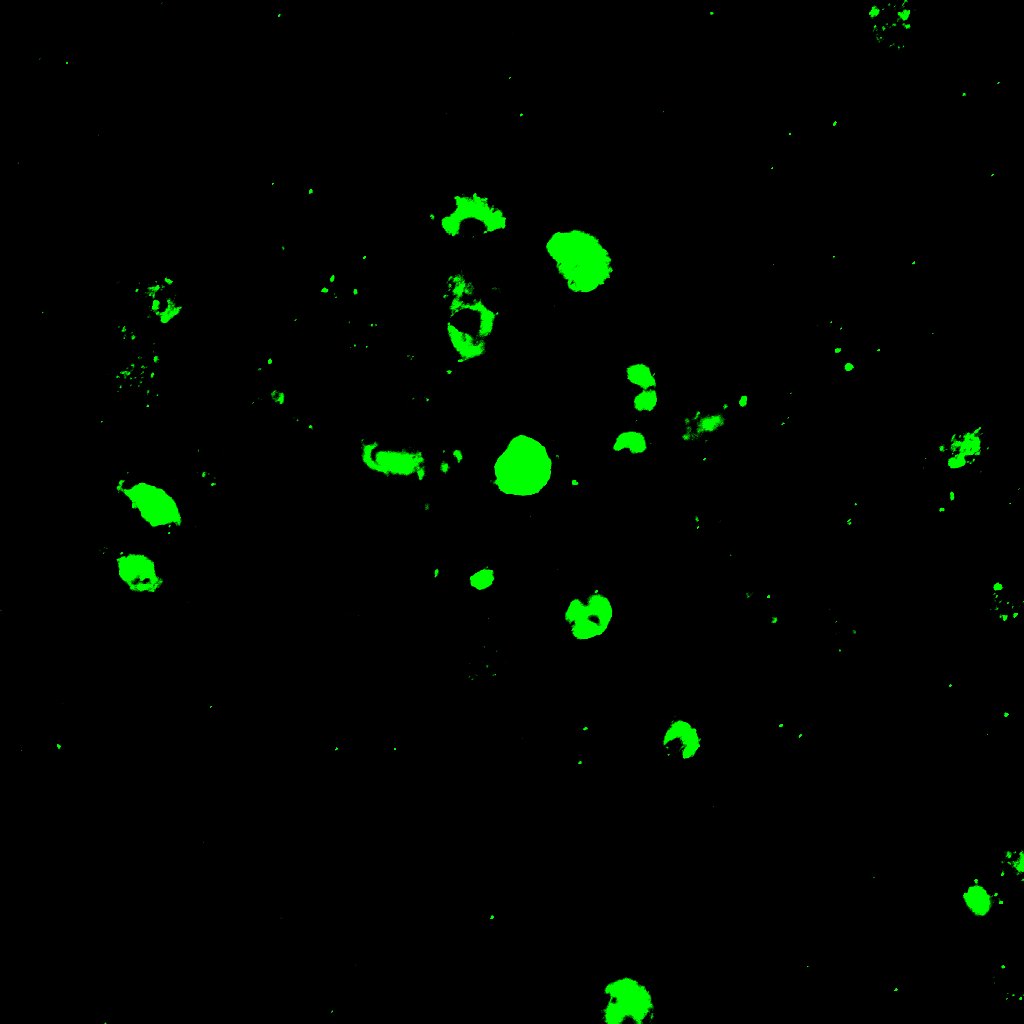

Supplement: S6 File — Representative images in the revised S2C Fig were generated from raw files woGF_4_ch1/2 (W/O GF); EF10cyc_4_ch1/2 (E+F10+Cyc); EF1shh_7_ch1/2 (E+F1+Shh). (ZIP) [file pone.0239995.s007.zip › S6_File/EF10cyc_7_ch1.jpg]

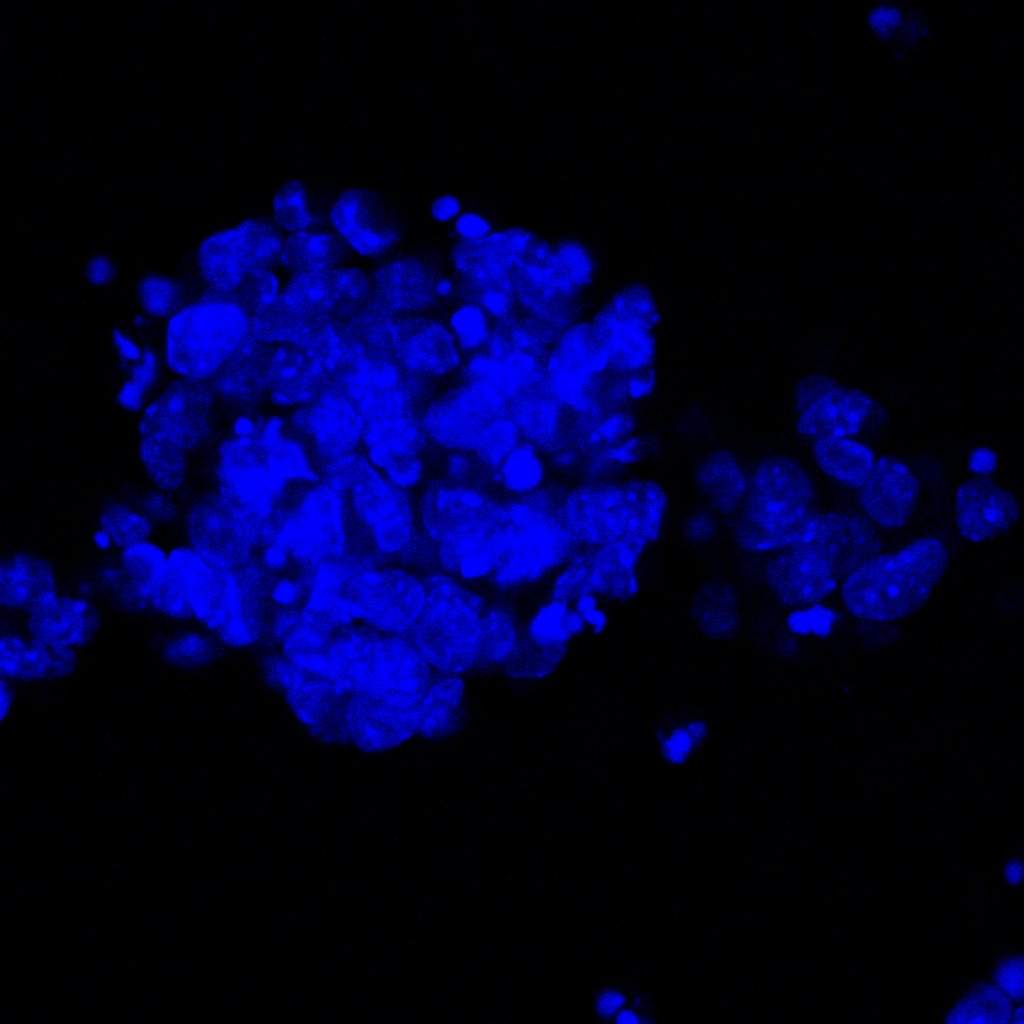

Supplement: S6 File — Representative images in the revised S2C Fig were generated from raw files woGF_4_ch1/2 (W/O GF); EF10cyc_4_ch1/2 (E+F10+Cyc); EF1shh_7_ch1/2 (E+F1+Shh). (ZIP) [file pone.0239995.s007.zip › S6_File/EF10cyc_7_ch2.jpg]

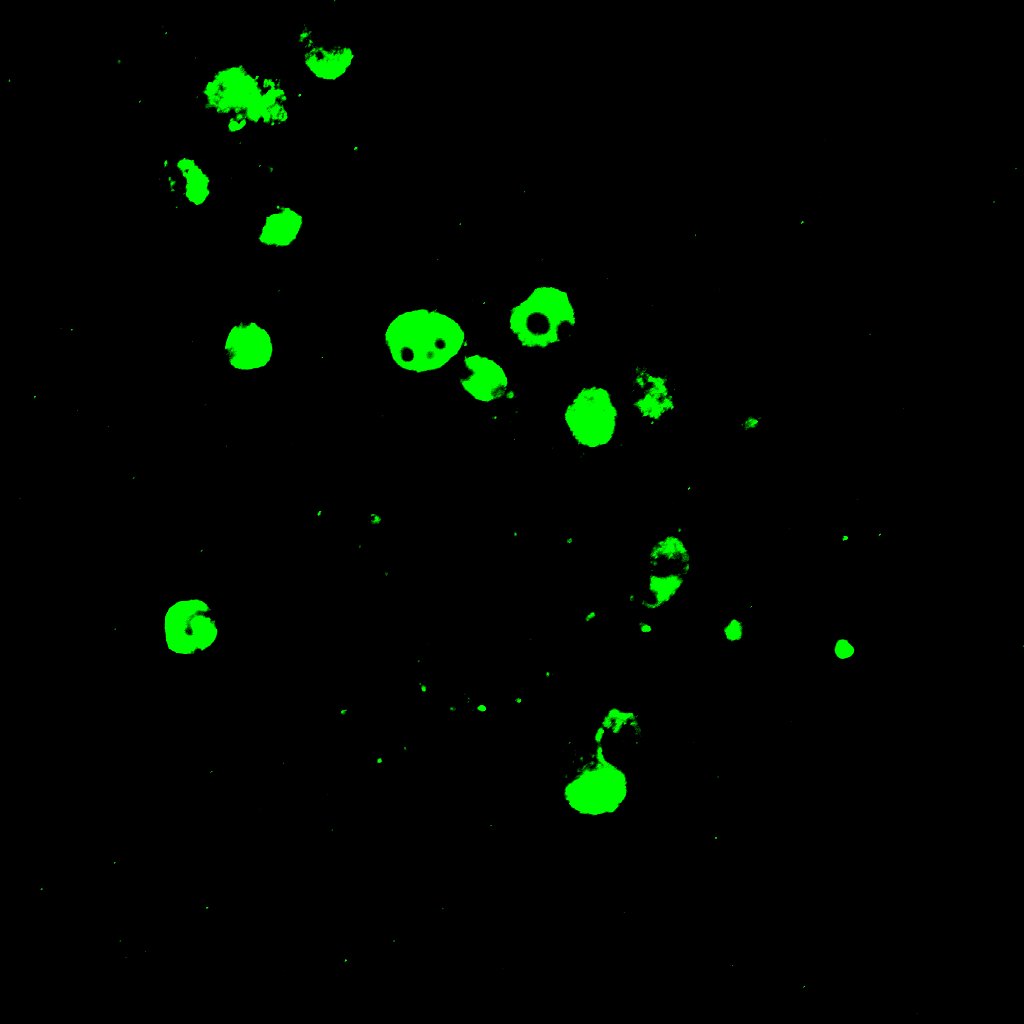

Supplement: S6 File — Representative images in the revised S2C Fig were generated from raw files woGF_4_ch1/2 (W/O GF); EF10cyc_4_ch1/2 (E+F10+Cyc); EF1shh_7_ch1/2 (E+F1+Shh). (ZIP) [file pone.0239995.s007.zip › S6_File/EF10cyc_8_ch1.jpg]

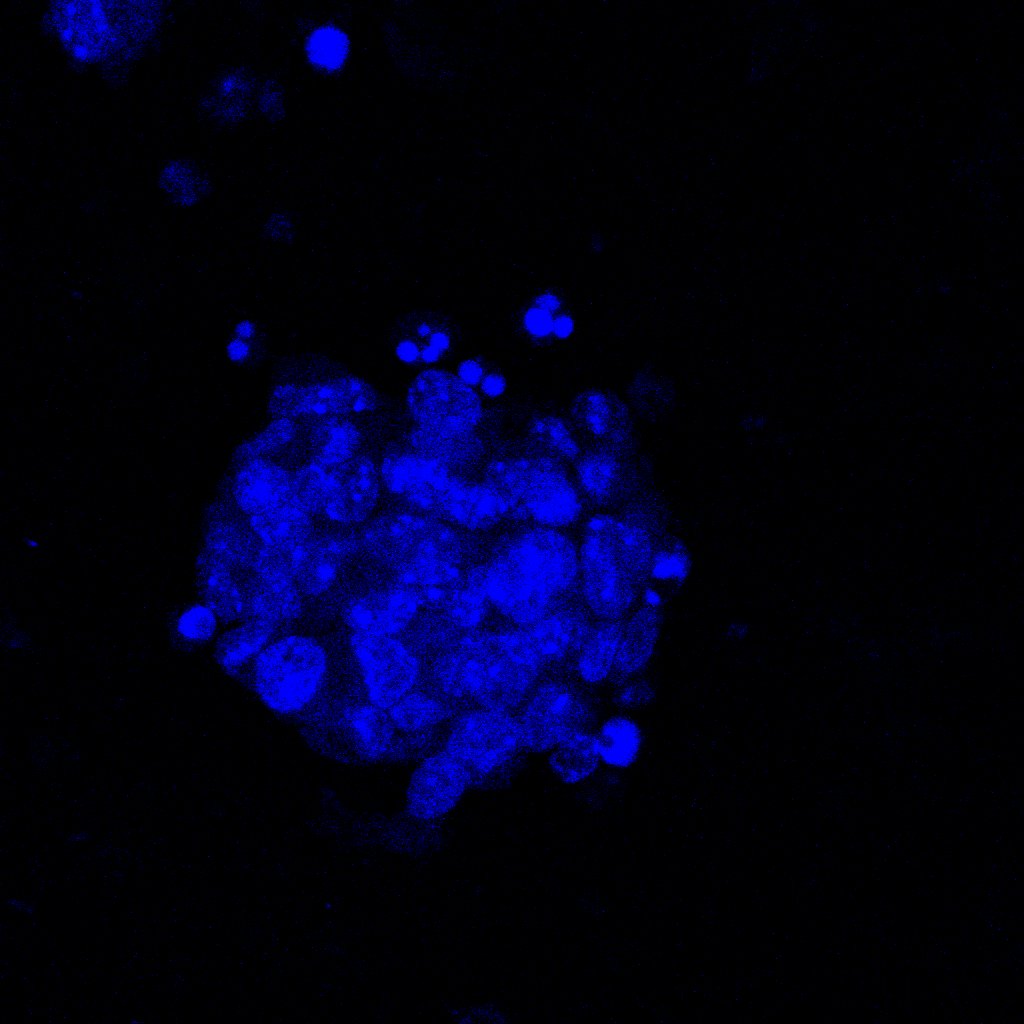

Supplement: S6 File — Representative images in the revised S2C Fig were generated from raw files woGF_4_ch1/2 (W/O GF); EF10cyc_4_ch1/2 (E+F10+Cyc); EF1shh_7_ch1/2 (E+F1+Shh). (ZIP) [file pone.0239995.s007.zip › S6_File/EF10cyc_8_ch2.jpg]

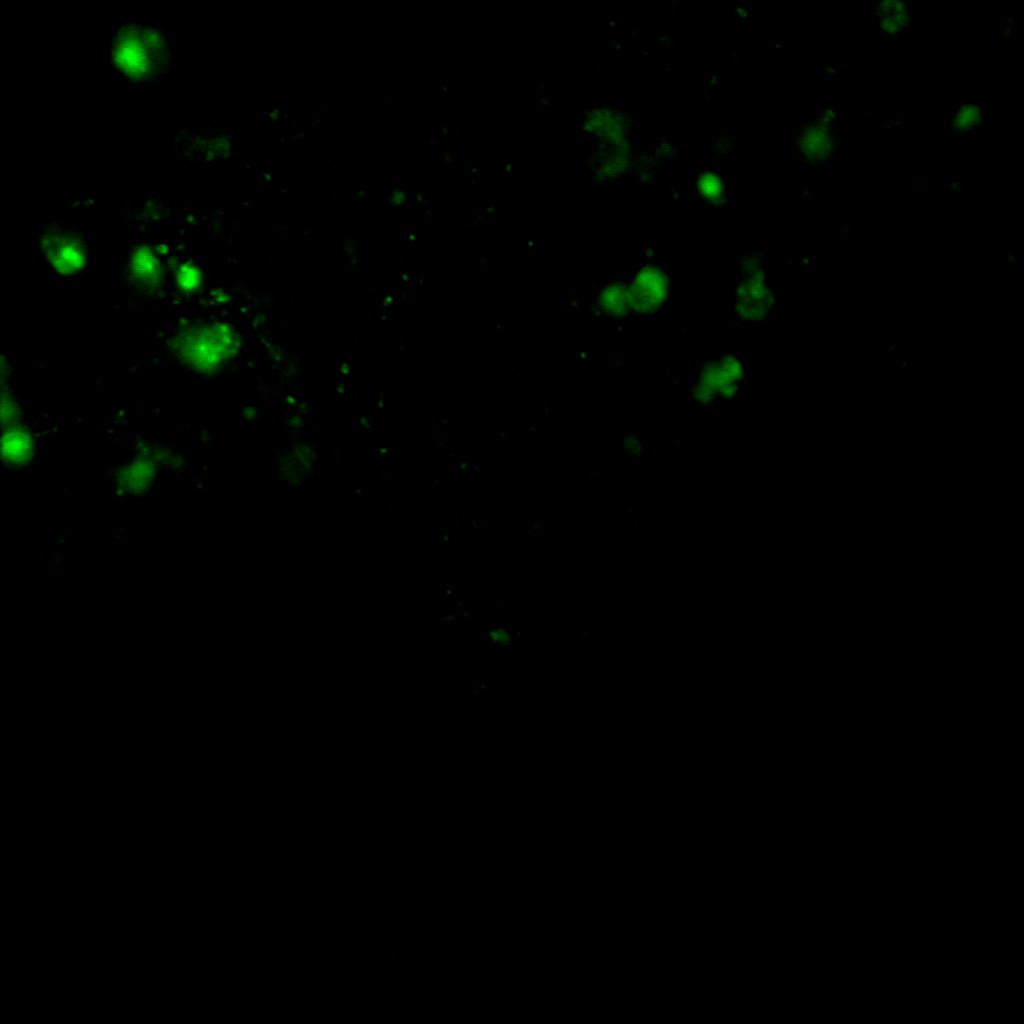

Supplement: S6 File — Representative images in the revised S2C Fig were generated from raw files woGF_4_ch1/2 (W/O GF); EF10cyc_4_ch1/2 (E+F10+Cyc); EF1shh_7_ch1/2 (E+F1+Shh). (ZIP) [file pone.0239995.s007.zip › S6_File/EF1shh_1_ch1.jpg]

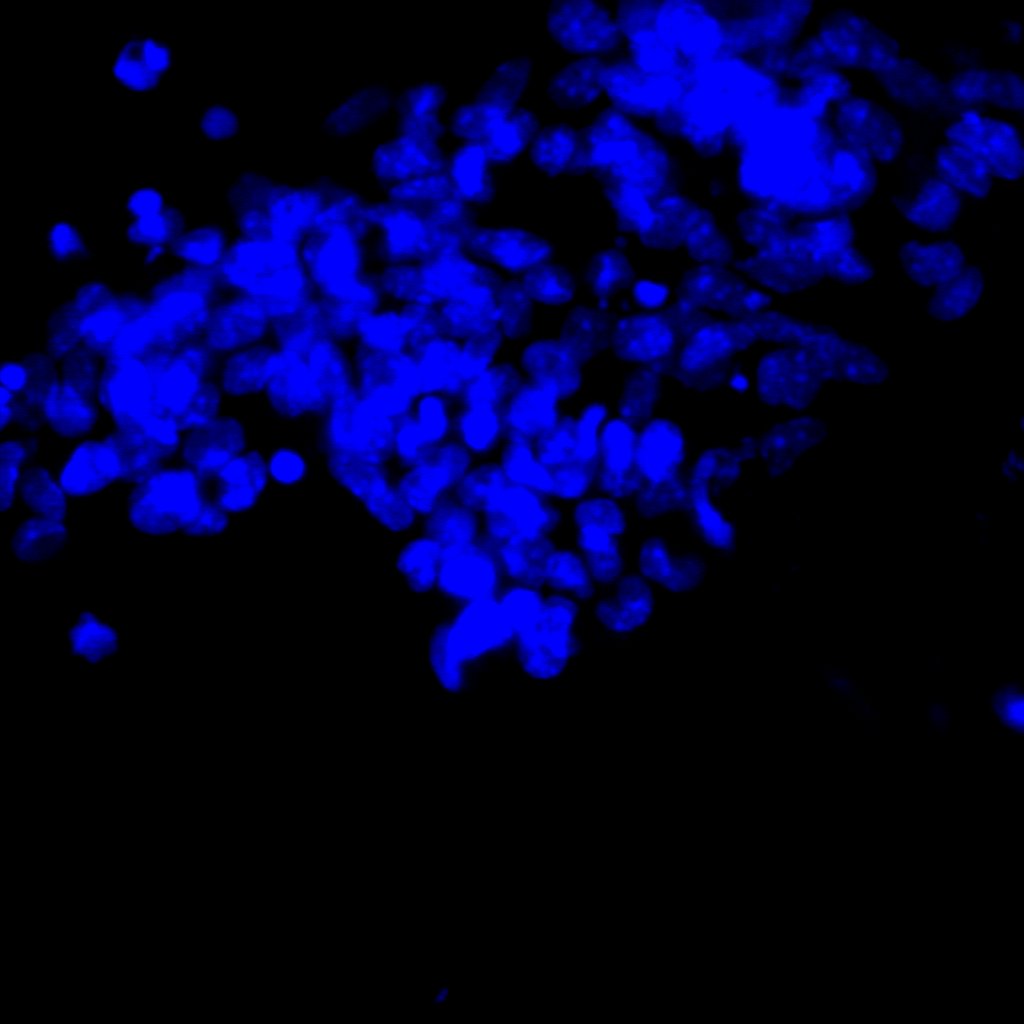

Supplement: S6 File — Representative images in the revised S2C Fig were generated from raw files woGF_4_ch1/2 (W/O GF); EF10cyc_4_ch1/2 (E+F10+Cyc); EF1shh_7_ch1/2 (E+F1+Shh). (ZIP) [file pone.0239995.s007.zip › S6_File/EF1shh_1_ch2.jpg]

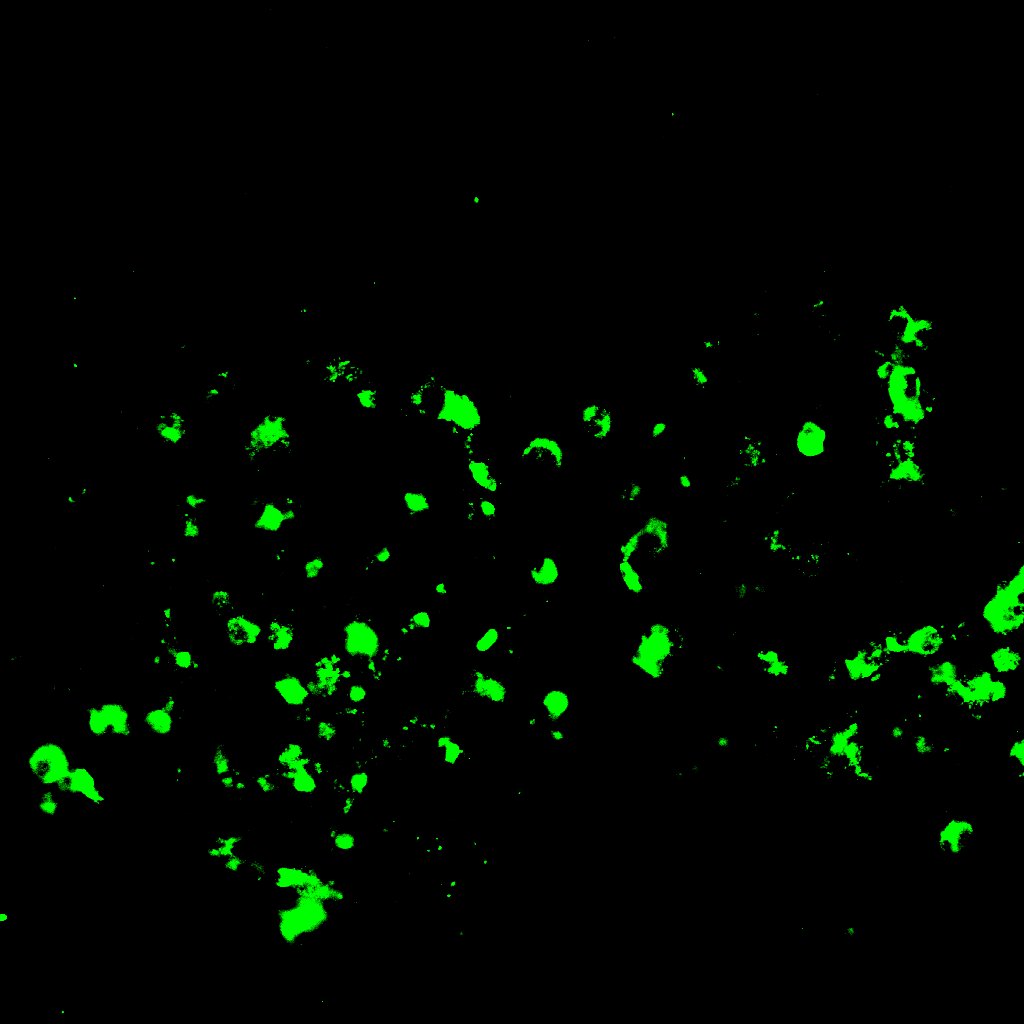

Supplement: S6 File — Representative images in the revised S2C Fig were generated from raw files woGF_4_ch1/2 (W/O GF); EF10cyc_4_ch1/2 (E+F10+Cyc); EF1shh_7_ch1/2 (E+F1+Shh). (ZIP) [file pone.0239995.s007.zip › S6_File/EF1shh_10_ch1.jpg]

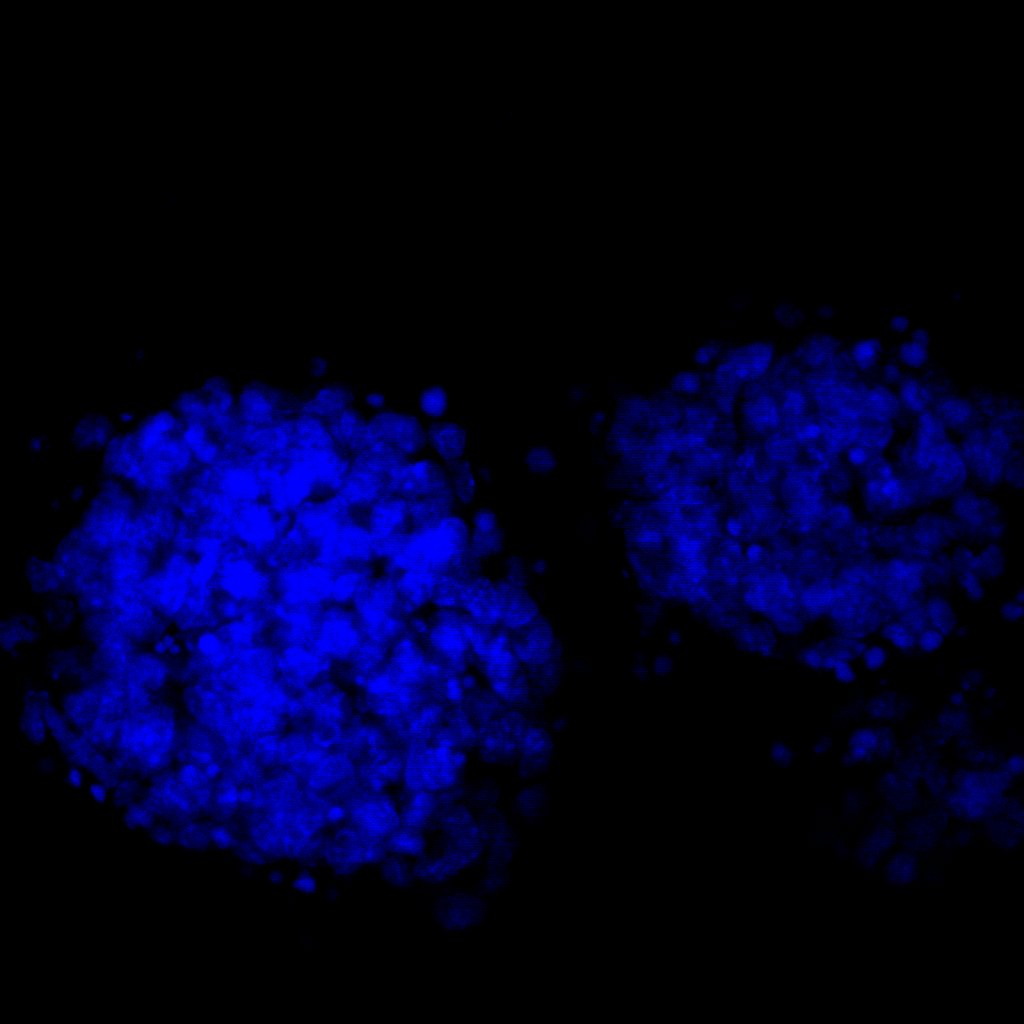

Supplement: S6 File — Representative images in the revised S2C Fig were generated from raw files woGF_4_ch1/2 (W/O GF); EF10cyc_4_ch1/2 (E+F10+Cyc); EF1shh_7_ch1/2 (E+F1+Shh). (ZIP) [file pone.0239995.s007.zip › S6_File/EF1shh_10_ch2.jpg]

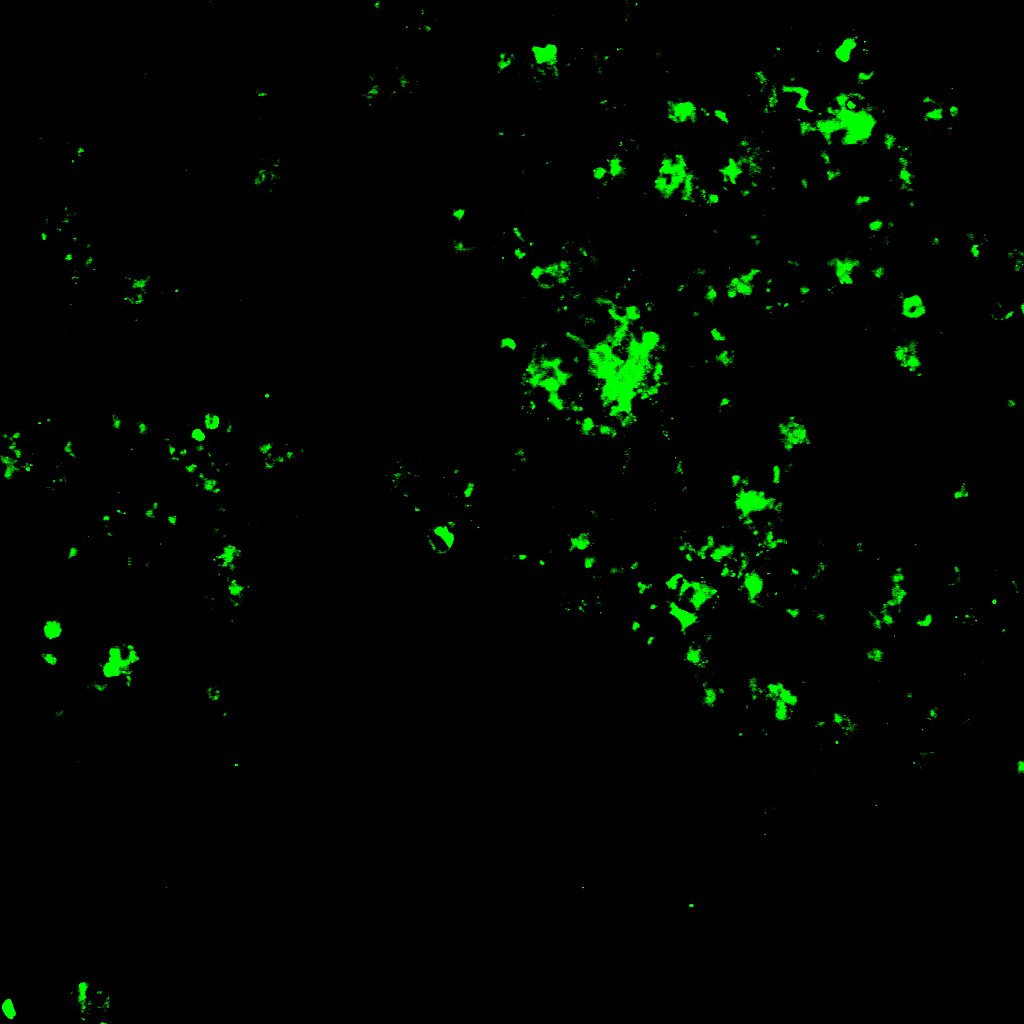

Supplement: S6 File — Representative images in the revised S2C Fig were generated from raw files woGF_4_ch1/2 (W/O GF); EF10cyc_4_ch1/2 (E+F10+Cyc); EF1shh_7_ch1/2 (E+F1+Shh). (ZIP) [file pone.0239995.s007.zip › S6_File/EF1shh_11_ch1.jpg]

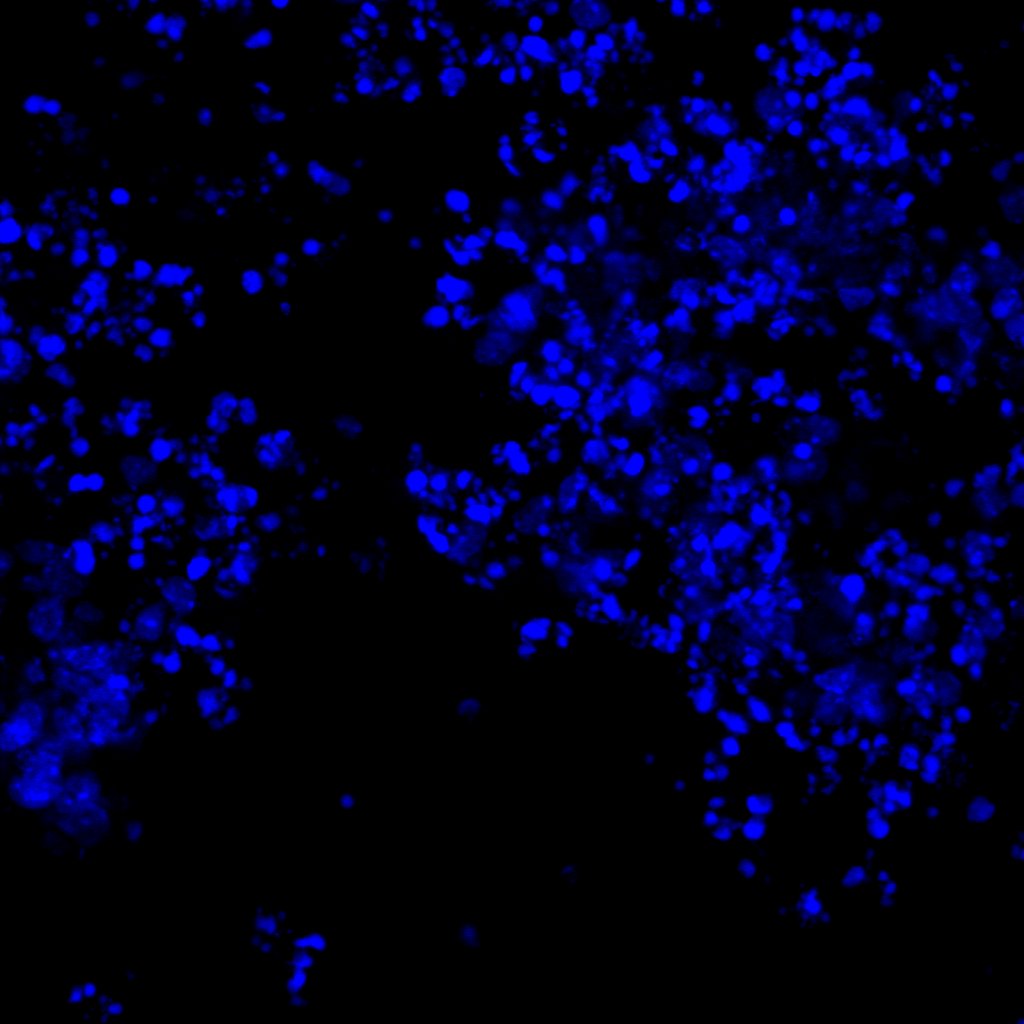

Supplement: S6 File — Representative images in the revised S2C Fig were generated from raw files woGF_4_ch1/2 (W/O GF); EF10cyc_4_ch1/2 (E+F10+Cyc); EF1shh_7_ch1/2 (E+F1+Shh). (ZIP) [file pone.0239995.s007.zip › S6_File/EF1shh_11_ch2.jpg]

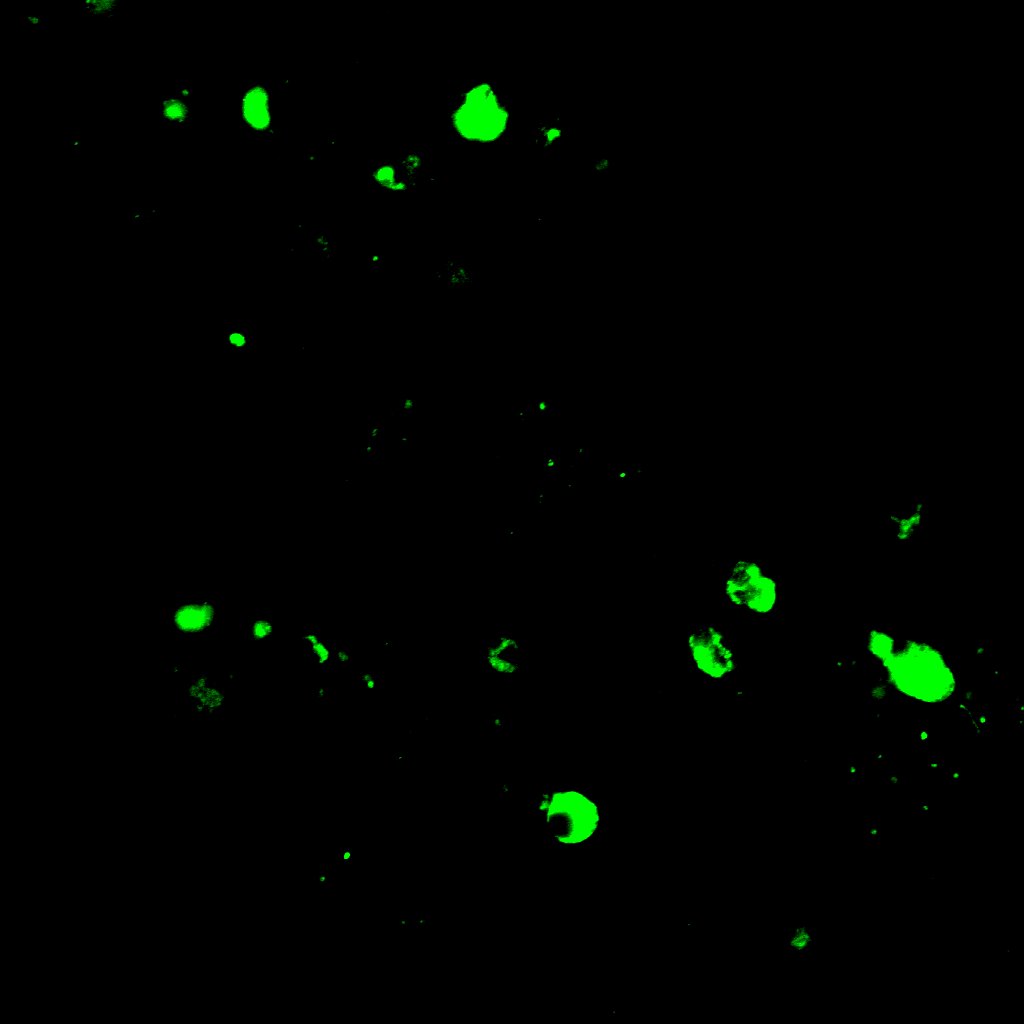

Supplement: S6 File — Representative images in the revised S2C Fig were generated from raw files woGF_4_ch1/2 (W/O GF); EF10cyc_4_ch1/2 (E+F10+Cyc); EF1shh_7_ch1/2 (E+F1+Shh). (ZIP) [file pone.0239995.s007.zip › S6_File/EF1shh_2_ch1.jpg]

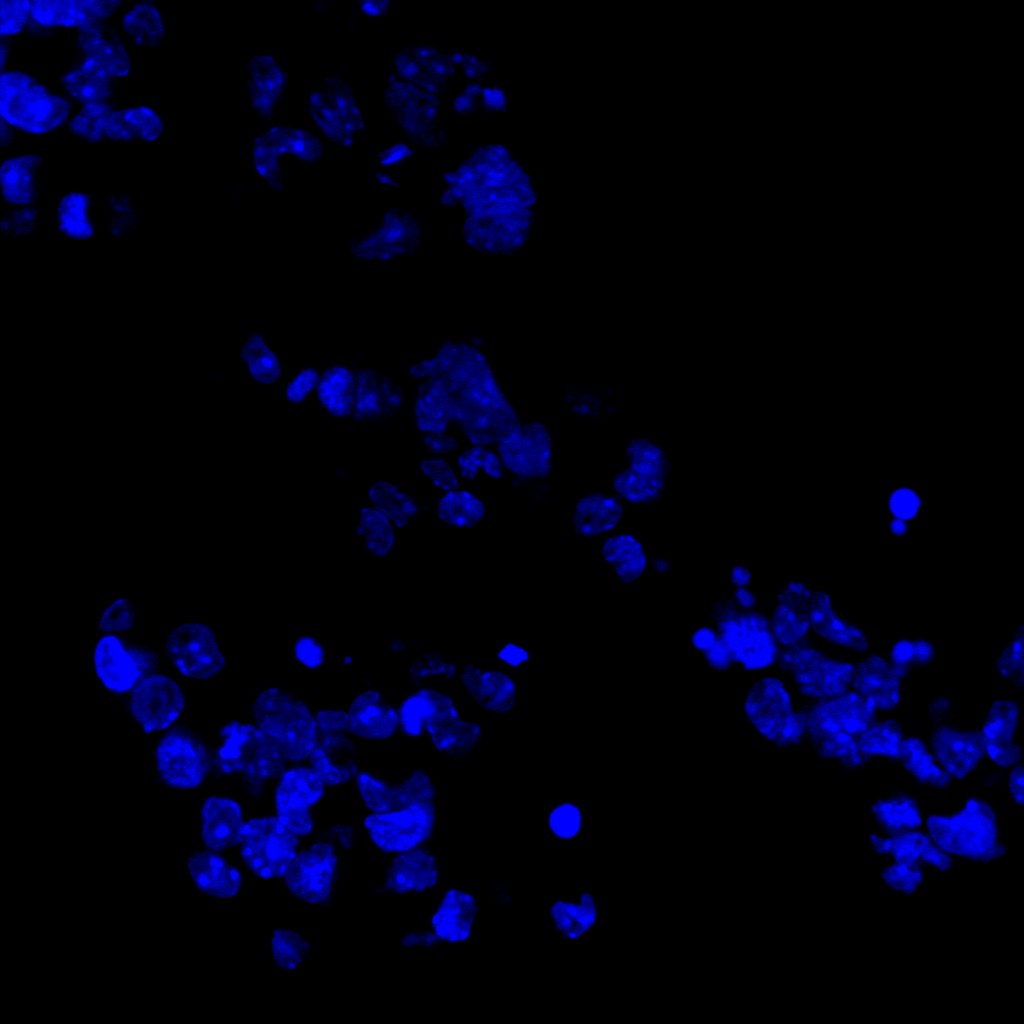

Supplement: S6 File — Representative images in the revised S2C Fig were generated from raw files woGF_4_ch1/2 (W/O GF); EF10cyc_4_ch1/2 (E+F10+Cyc); EF1shh_7_ch1/2 (E+F1+Shh). (ZIP) [file pone.0239995.s007.zip › S6_File/EF1shh_2_ch2.jpg]

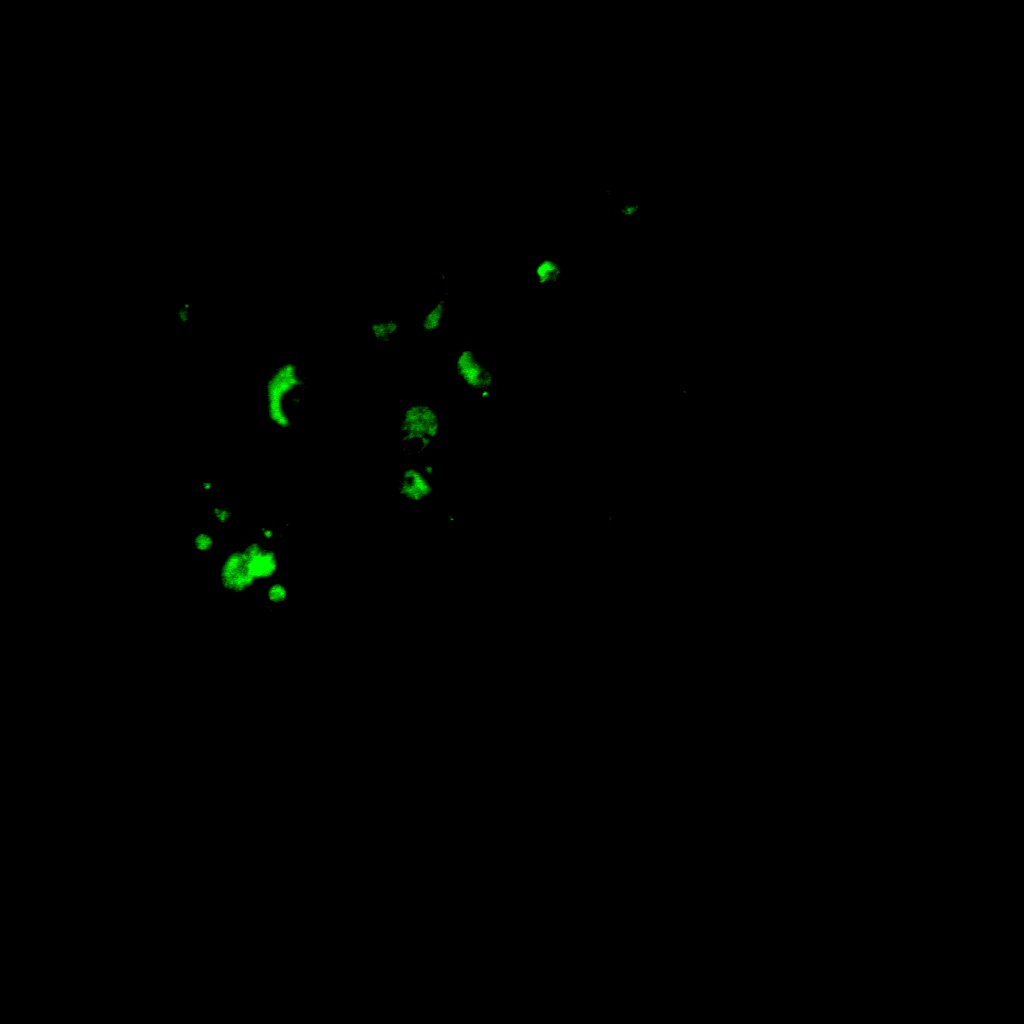

Supplement: S6 File — Representative images in the revised S2C Fig were generated from raw files woGF_4_ch1/2 (W/O GF); EF10cyc_4_ch1/2 (E+F10+Cyc); EF1shh_7_ch1/2 (E+F1+Shh). (ZIP) [file pone.0239995.s007.zip › S6_File/EF1shh_3_ch1.jpg]

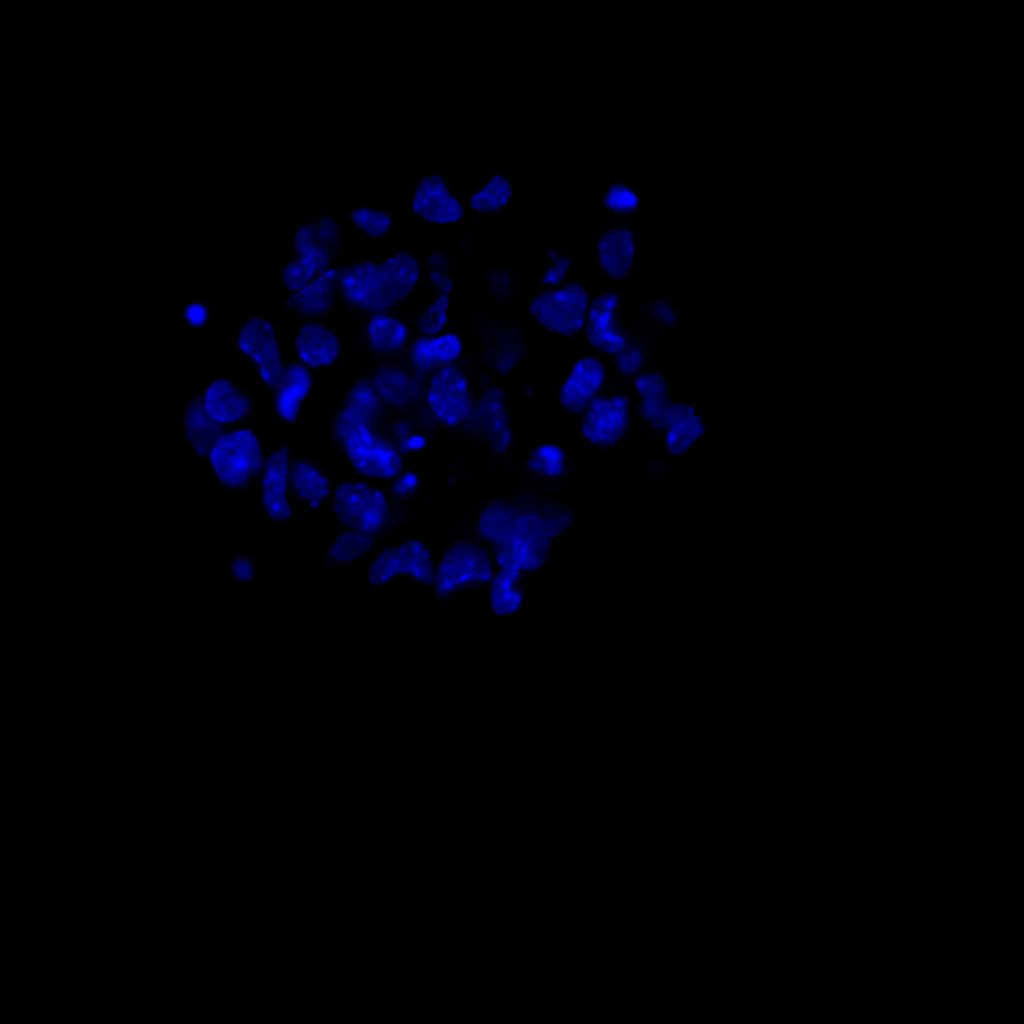

Supplement: S6 File — Representative images in the revised S2C Fig were generated from raw files woGF_4_ch1/2 (W/O GF); EF10cyc_4_ch1/2 (E+F10+Cyc); EF1shh_7_ch1/2 (E+F1+Shh). (ZIP) [file pone.0239995.s007.zip › S6_File/EF1shh_3_ch2.jpg]

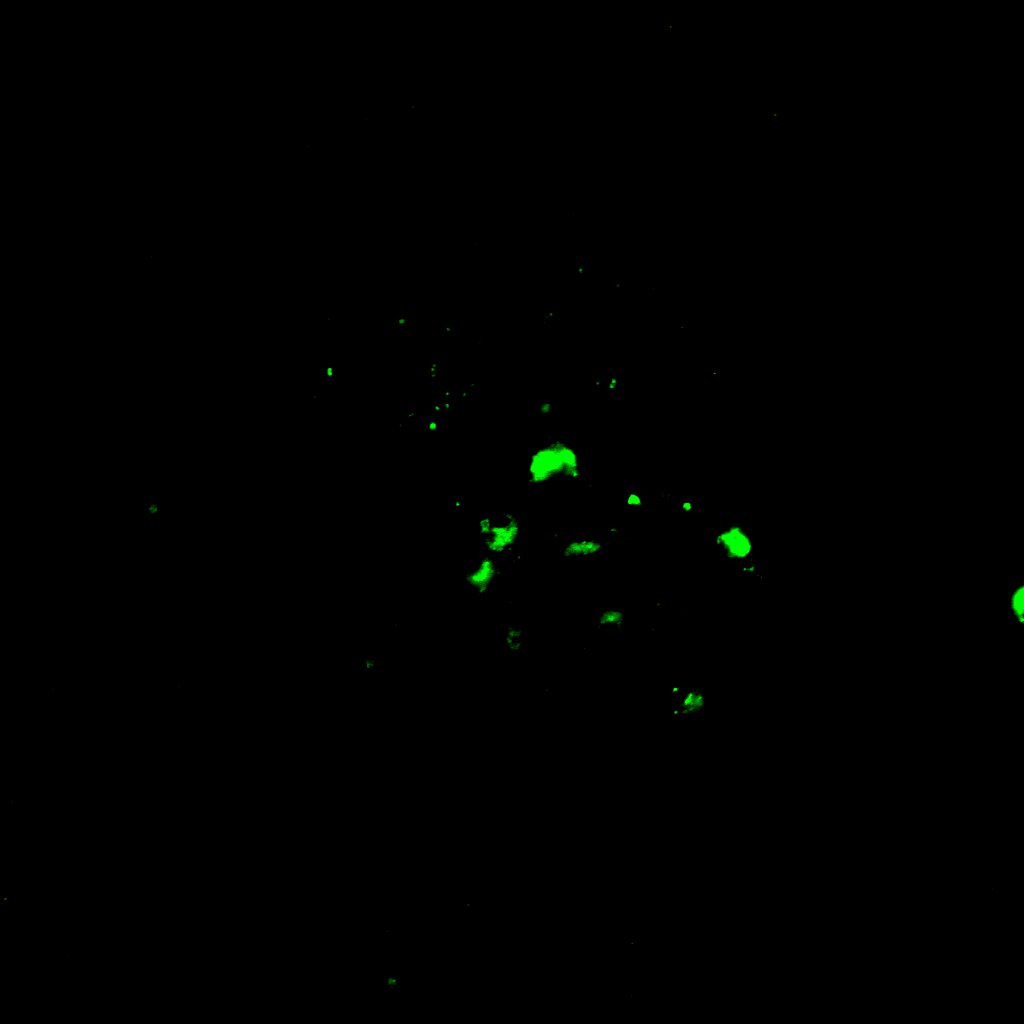

Supplement: S6 File — Representative images in the revised S2C Fig were generated from raw files woGF_4_ch1/2 (W/O GF); EF10cyc_4_ch1/2 (E+F10+Cyc); EF1shh_7_ch1/2 (E+F1+Shh). (ZIP) [file pone.0239995.s007.zip › S6_File/EF1shh_4_ch1.jpg]

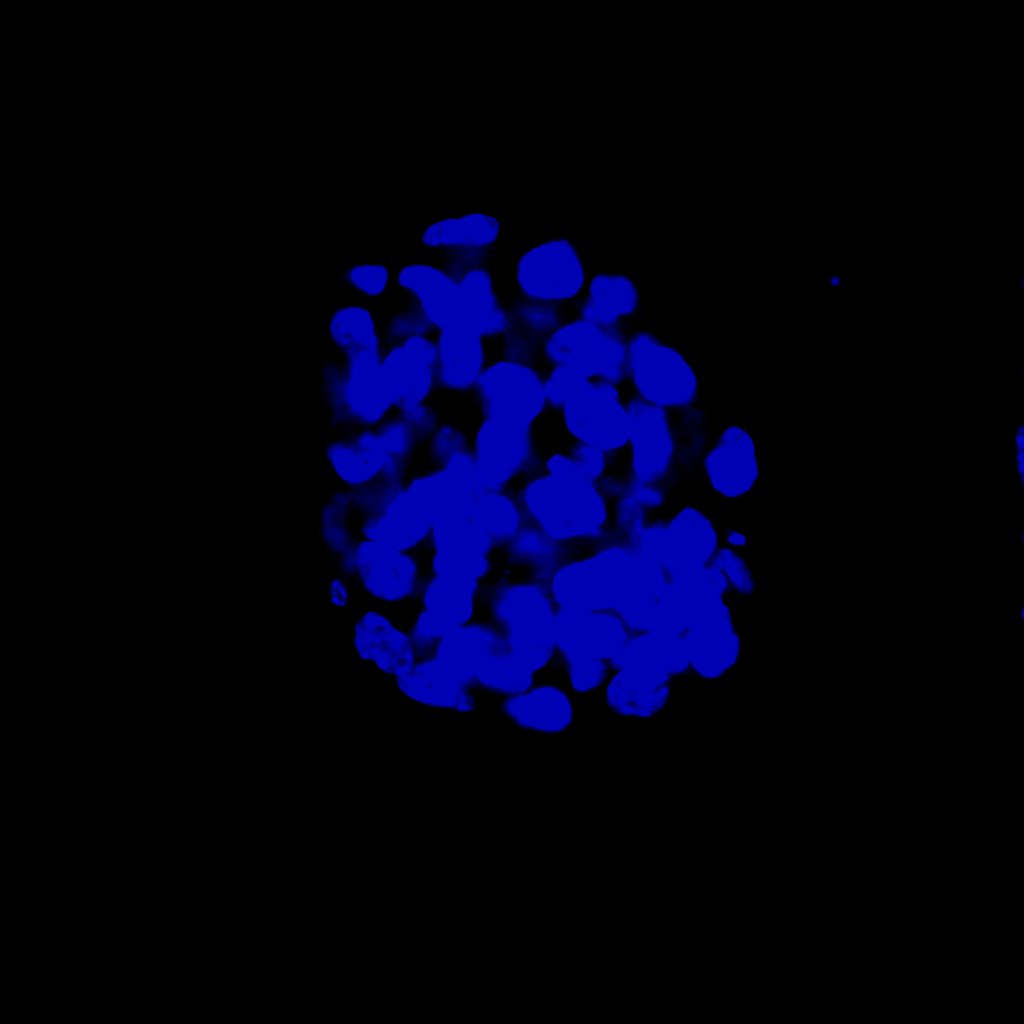

Supplement: S6 File — Representative images in the revised S2C Fig were generated from raw files woGF_4_ch1/2 (W/O GF); EF10cyc_4_ch1/2 (E+F10+Cyc); EF1shh_7_ch1/2 (E+F1+Shh). (ZIP) [file pone.0239995.s007.zip › S6_File/EF1shh_4_ch2.jpg]

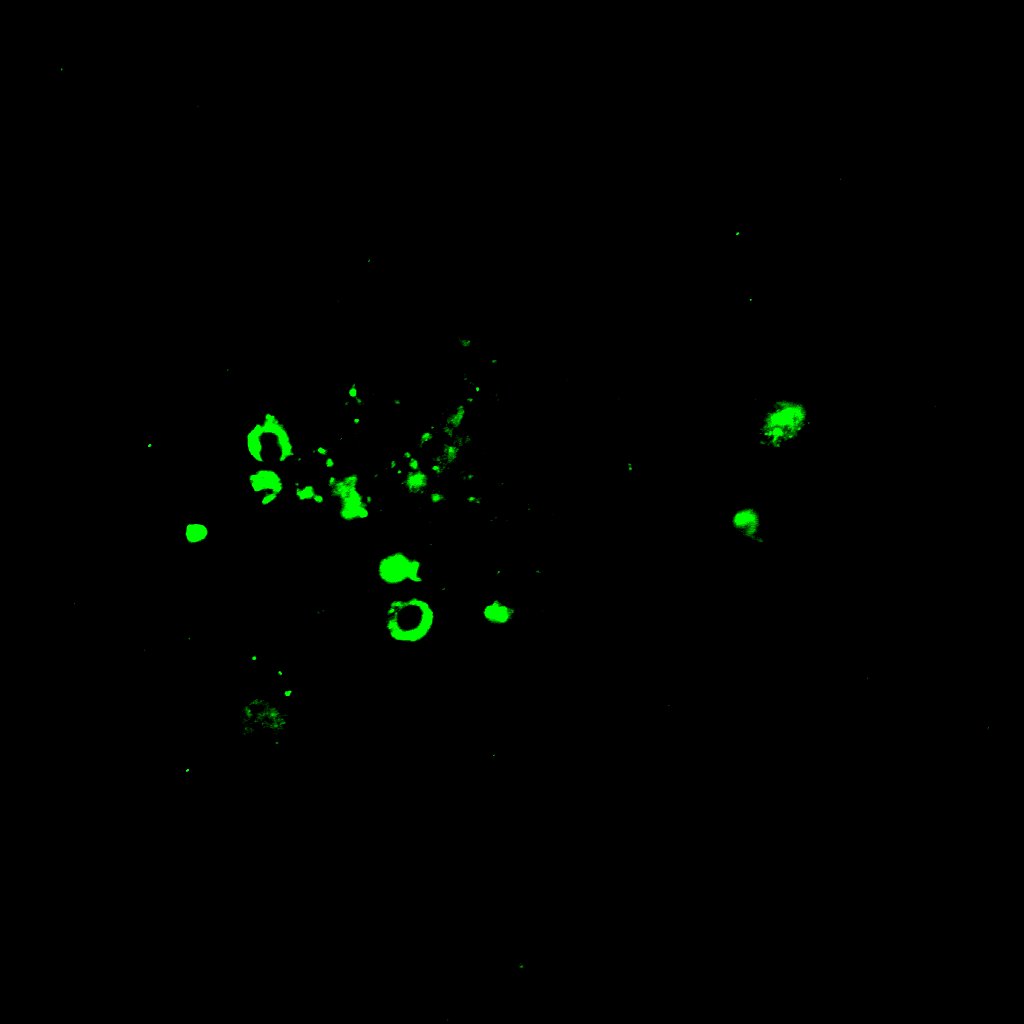

Supplement: S6 File — Representative images in the revised S2C Fig were generated from raw files woGF_4_ch1/2 (W/O GF); EF10cyc_4_ch1/2 (E+F10+Cyc); EF1shh_7_ch1/2 (E+F1+Shh). (ZIP) [file pone.0239995.s007.zip › S6_File/EF1shh_5_ch1.jpg]

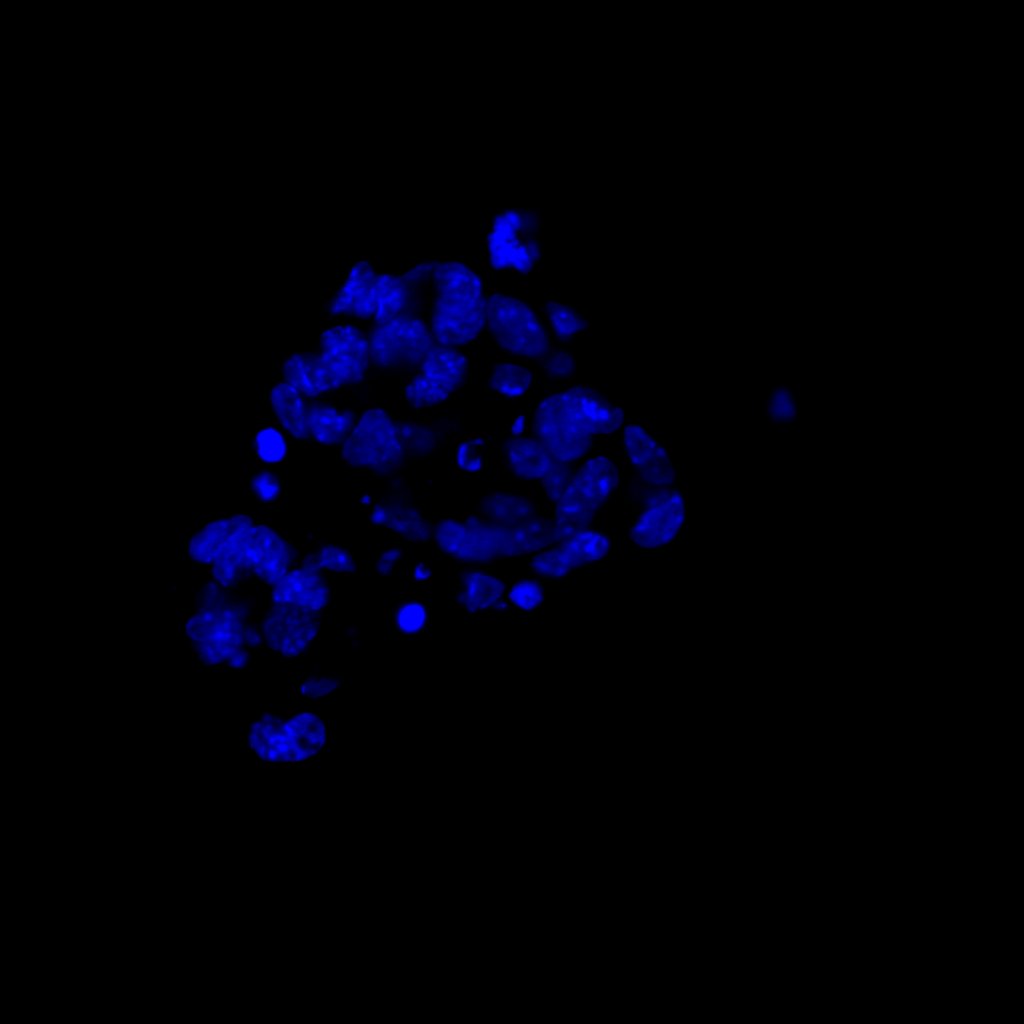

Supplement: S6 File — Representative images in the revised S2C Fig were generated from raw files woGF_4_ch1/2 (W/O GF); EF10cyc_4_ch1/2 (E+F10+Cyc); EF1shh_7_ch1/2 (E+F1+Shh). (ZIP) [file pone.0239995.s007.zip › S6_File/EF1shh_5_ch2.jpg]

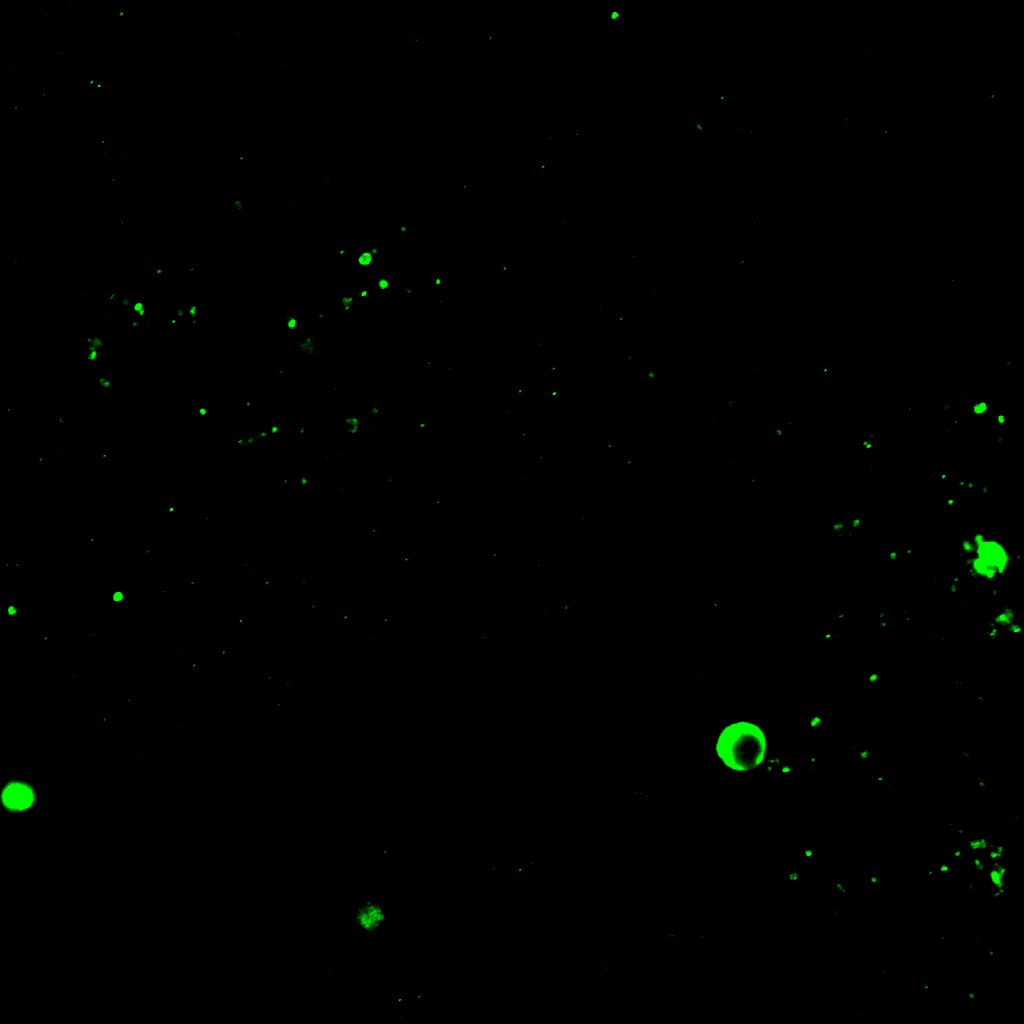

Supplement: S6 File — Representative images in the revised S2C Fig were generated from raw files woGF_4_ch1/2 (W/O GF); EF10cyc_4_ch1/2 (E+F10+Cyc); EF1shh_7_ch1/2 (E+F1+Shh). (ZIP) [file pone.0239995.s007.zip › S6_File/EF1shh_6_ch1.jpg]

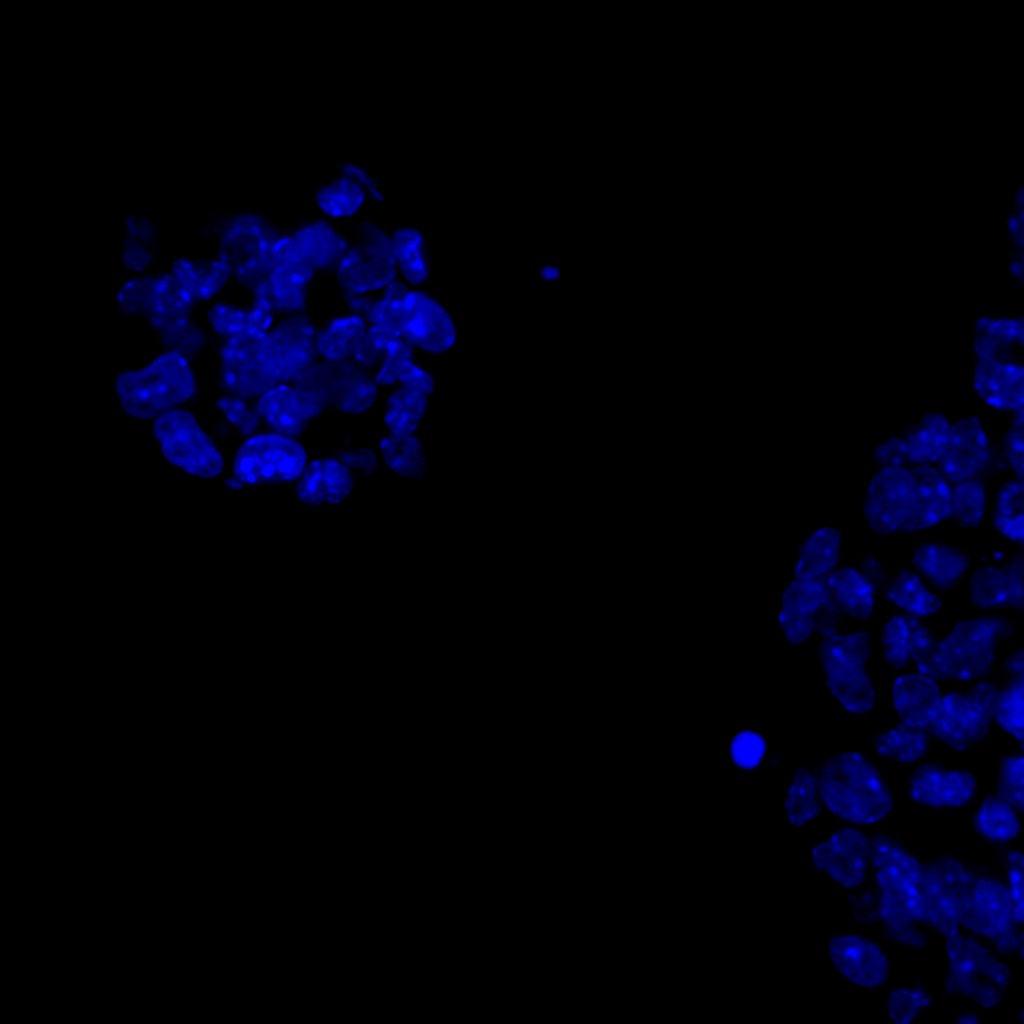

Supplement: S6 File — Representative images in the revised S2C Fig were generated from raw files woGF_4_ch1/2 (W/O GF); EF10cyc_4_ch1/2 (E+F10+Cyc); EF1shh_7_ch1/2 (E+F1+Shh). (ZIP) [file pone.0239995.s007.zip › S6_File/EF1shh_6_ch2.jpg]

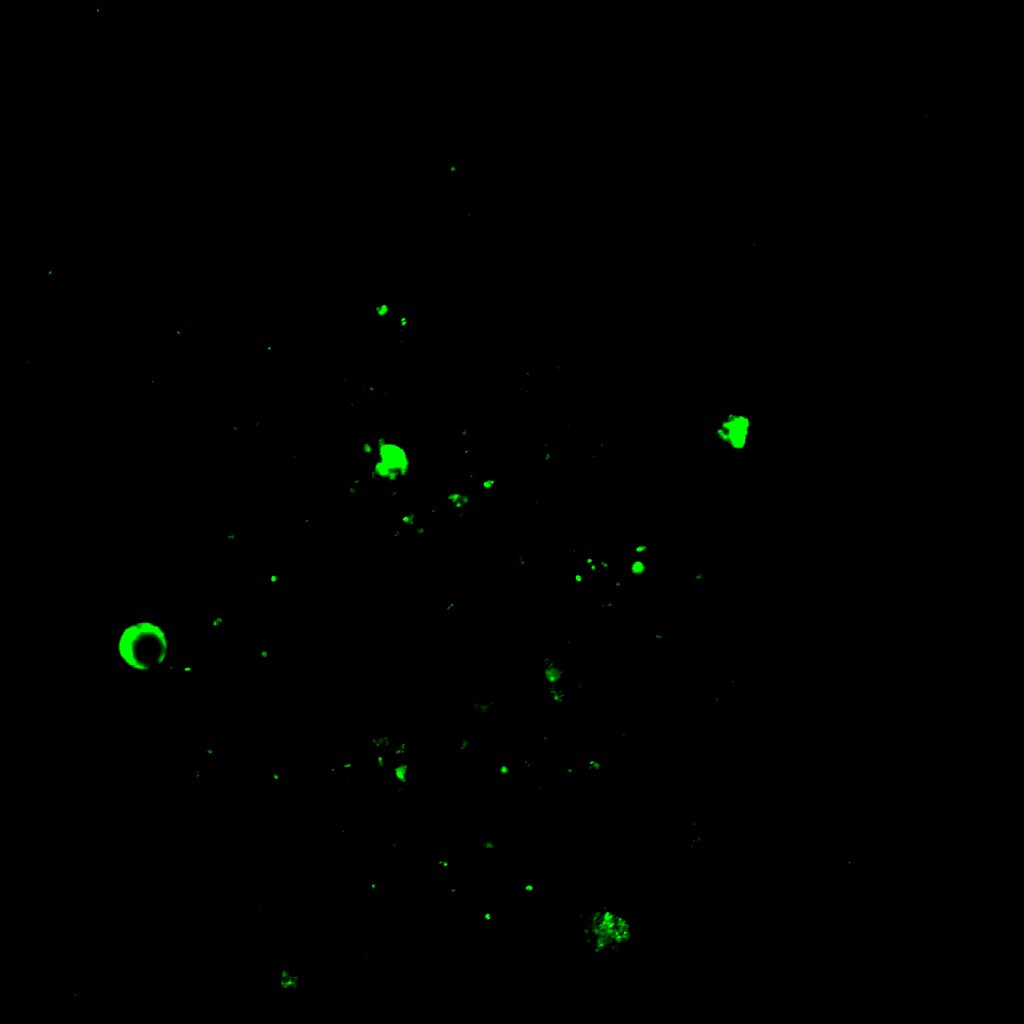

Supplement: S6 File — Representative images in the revised S2C Fig were generated from raw files woGF_4_ch1/2 (W/O GF); EF10cyc_4_ch1/2 (E+F10+Cyc); EF1shh_7_ch1/2 (E+F1+Shh). (ZIP) [file pone.0239995.s007.zip › S6_File/EF1shh_7_ch1.jpg]
