# Supplementary material for: Correction: Proliferation of Murine Midbrain Neural Stem Cells Depends upon an Endogenous Sonic Hedgehog (Shh) Source
Source: PLoS One. 2020 Sep 24;15(9):e0239995. doi: 10.1371/journal.pone.0239995 (PMC7514037; doi:10.1371/journal.pone.0239995)

EXPERIMENTAL SERIES 1 SDS PAGE 10%

Lane 1: control 7 days of treatment in differentiation conditions (without growth factors)
Lane 2: control 7 days of treatment in differentiation conditions
Lane 3: Shh 7 days of treatment in differentiation conditions
Lane 4: Shh 7 days of treatment in differentiation conditions
Lane 5: control 14 days of treatment in differentiation conditions
Lane 6: control 14 days of treatment in differentiation conditions
Lane 7: Shh 14 days of treatment in differentiation conditions
Lane 8: Shh 14 days of treatment in differentiation conditions
Lane 9: Neurospheres control

NESTIN

1 2 3 4 5 6 7 8 9

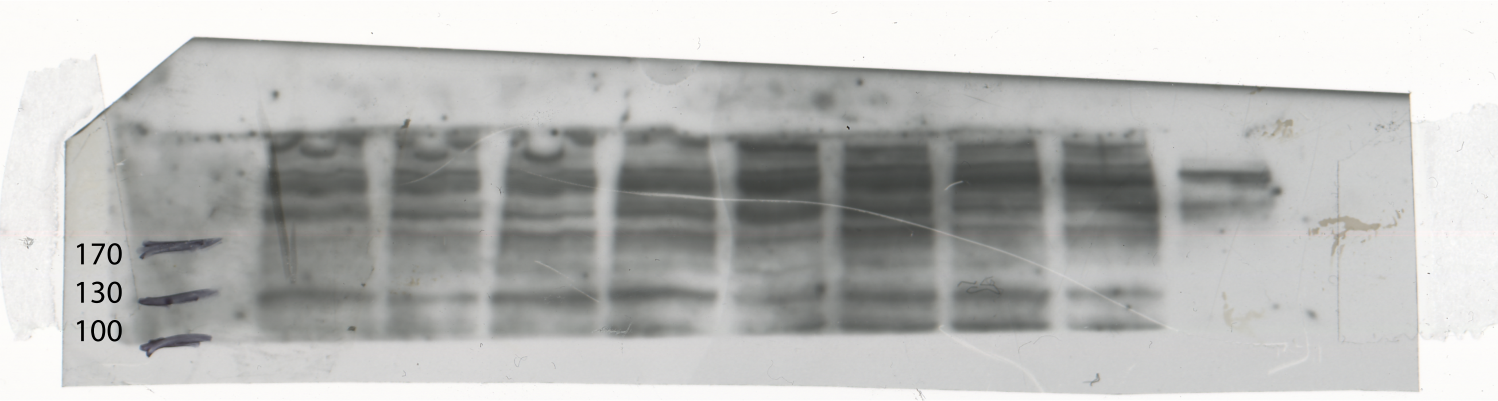

TUBULIN

55 KDa

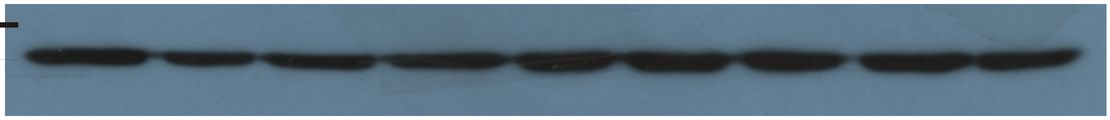

CYCLIN D1

40 KDa

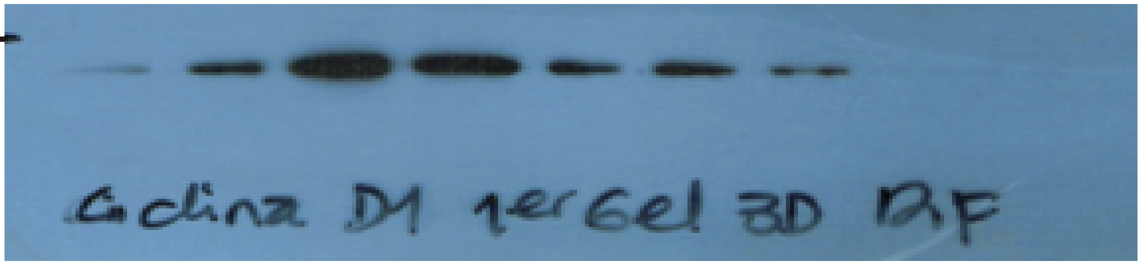

BLBP

25 KDa

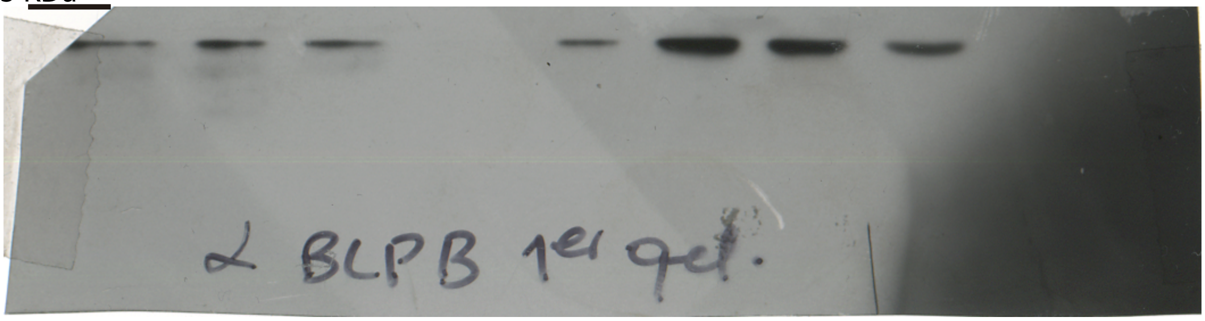

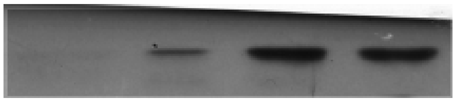

Supplement: S1 File — The membrane was cut at 100 kDa and 25 kDa to facilitate detection of different proteins. Lanes 6, 7 were included in panel B; lanes 2, 3 of the indicated blots were included in panels D and E. The tubulin, Cyclin D1, BLBP, and Nestin data were generated by cutting the same blot into three sections and re-probing with sequential antibodies. I.e., these data represent results for the same neurosphere collagen culture extracts run on the same gel/blot. Abcam ab11306-25 was used for the anti-Nestin western blot. (PDF) [file pone.0239995.s002.pdf]
