# Supplementary material for: Correction: Proliferation of Murine Midbrain Neural Stem Cells Depends upon an Endogenous Sonic Hedgehog (Shh) Source
Source: PLoS One. 2020 Sep 24;15(9):e0239995. doi: 10.1371/journal.pone.0239995 (PMC7514037; doi:10.1371/journal.pone.0239995)

EXPERIMENTAL SERIES2 SDS PAGE 8 %

1. Control 3 days of treatment in differentiation conditions (without growth factors)
2. 3 days of treatment with cyclopamine in differentiation conditions
3. Control 7 days of treatment in differentiation conditions
4. Control 7 days of treatment in differentiation conditions
5. 7 days of treatment with cyclopamine in differentiation conditions
6. 7 days of treatment with cyclopamine in differentiation conditions
7. 7 days of treatment with Shh in differentiation conditions
8. 7 days of treatment with Shh in differentiation conditions
9. 7 days of treatment with Shh in differentiation conditions

TUBULIN

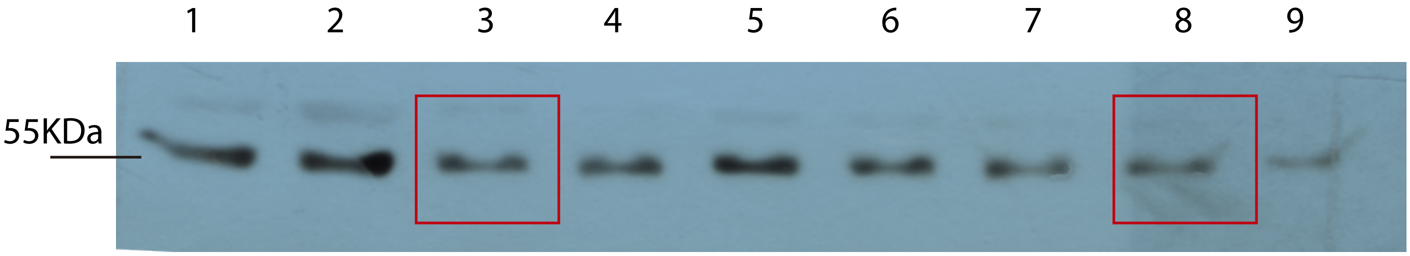

SOX 2

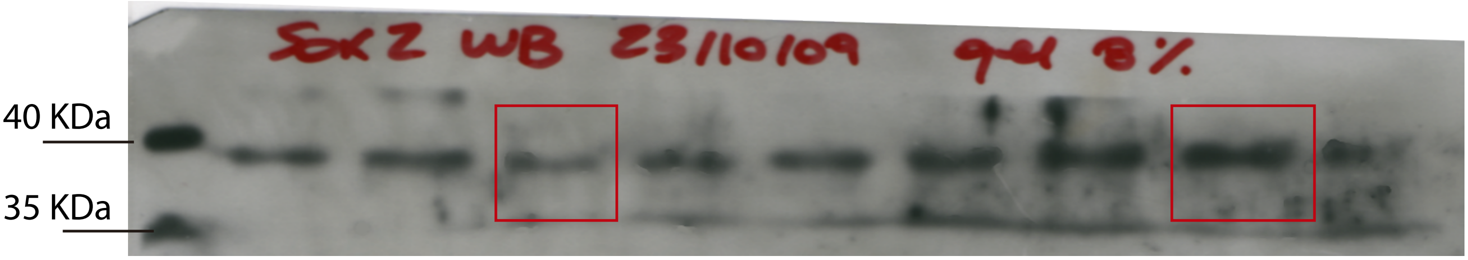

Supplement: S2 File — The membrane was cut, the 40–70 kDa fragment was used to detect a-tubulin and the 35–40 kDa fragment was used to detect Sox-2. Lanes 3 and 8 are shown in the figure. (PDF) [file pone.0239995.s003.pdf]
