# Supplementary figures and images for: Correction: Proliferation of Murine Midbrain Neural Stem Cells Depends upon an Endogenous Sonic Hedgehog (Shh) Source (part 2 of 2)
Source: PLoS One. 2020 Sep 24;15(9):e0239995. doi: 10.1371/journal.pone.0239995 (PMC7514037; doi:10.1371/journal.pone.0239995)

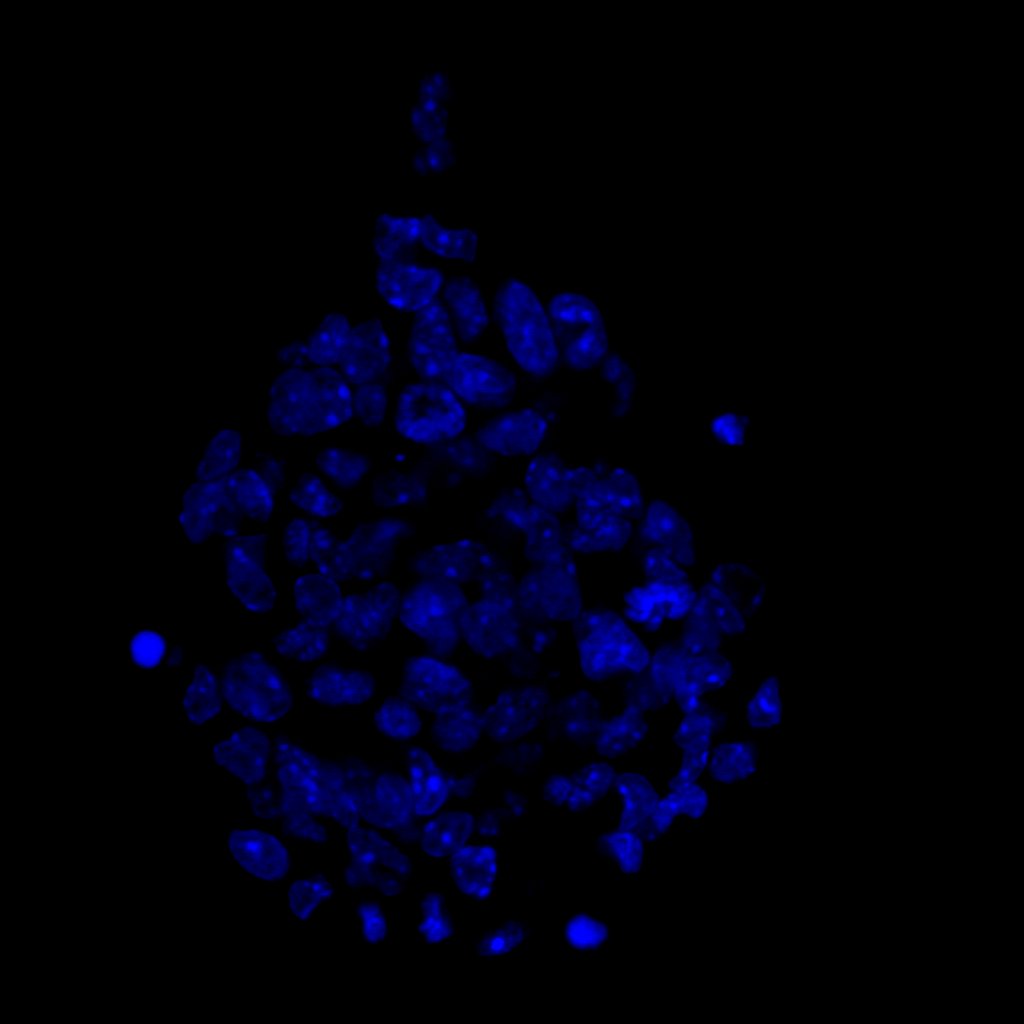

Supplement: S6 File — Representative images in the revised S2C Fig were generated from raw files woGF_4_ch1/2 (W/O GF); EF10cyc_4_ch1/2 (E+F10+Cyc); EF1shh_7_ch1/2 (E+F1+Shh). (ZIP) [file pone.0239995.s007.zip › S6_File/EF1shh_7_ch2.jpg]

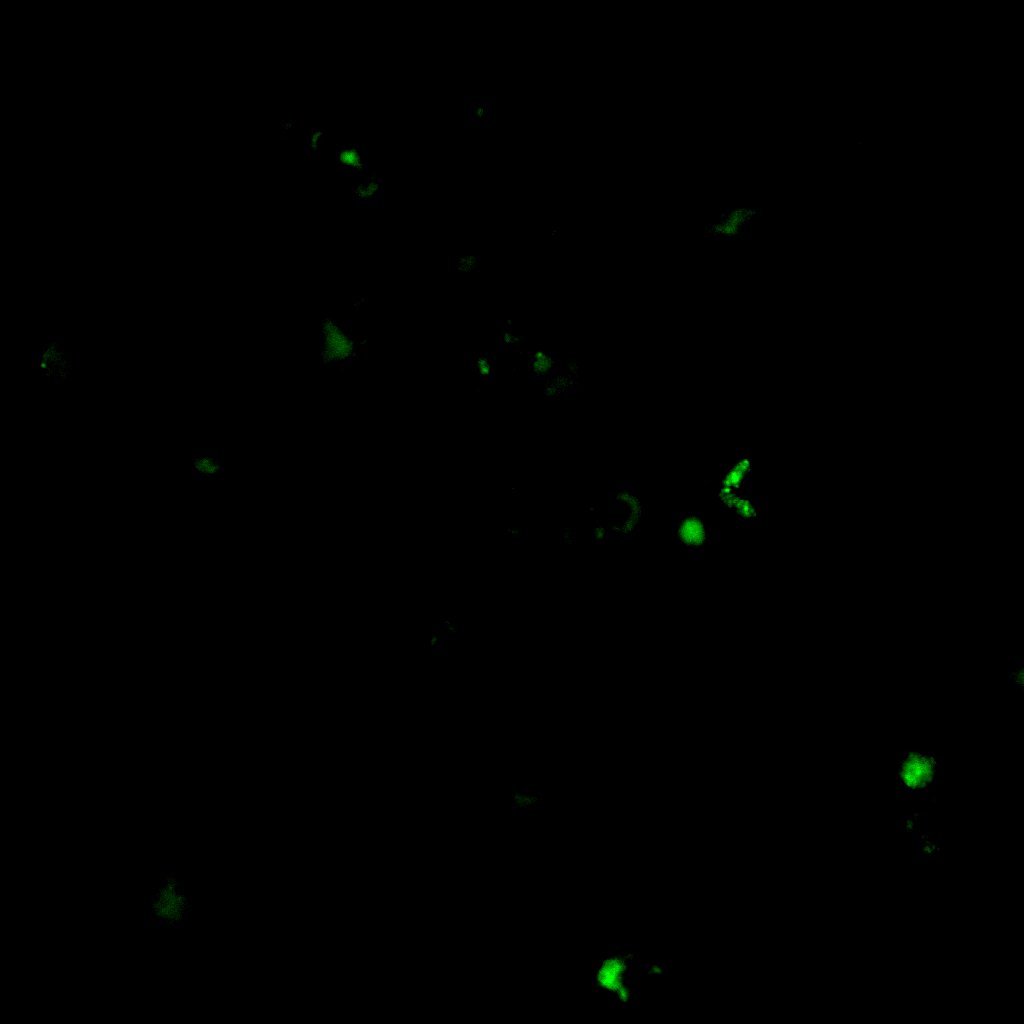

Supplement: S6 File — Representative images in the revised S2C Fig were generated from raw files woGF_4_ch1/2 (W/O GF); EF10cyc_4_ch1/2 (E+F10+Cyc); EF1shh_7_ch1/2 (E+F1+Shh). (ZIP) [file pone.0239995.s007.zip › S6_File/EF1shh_8_ch1.jpg]

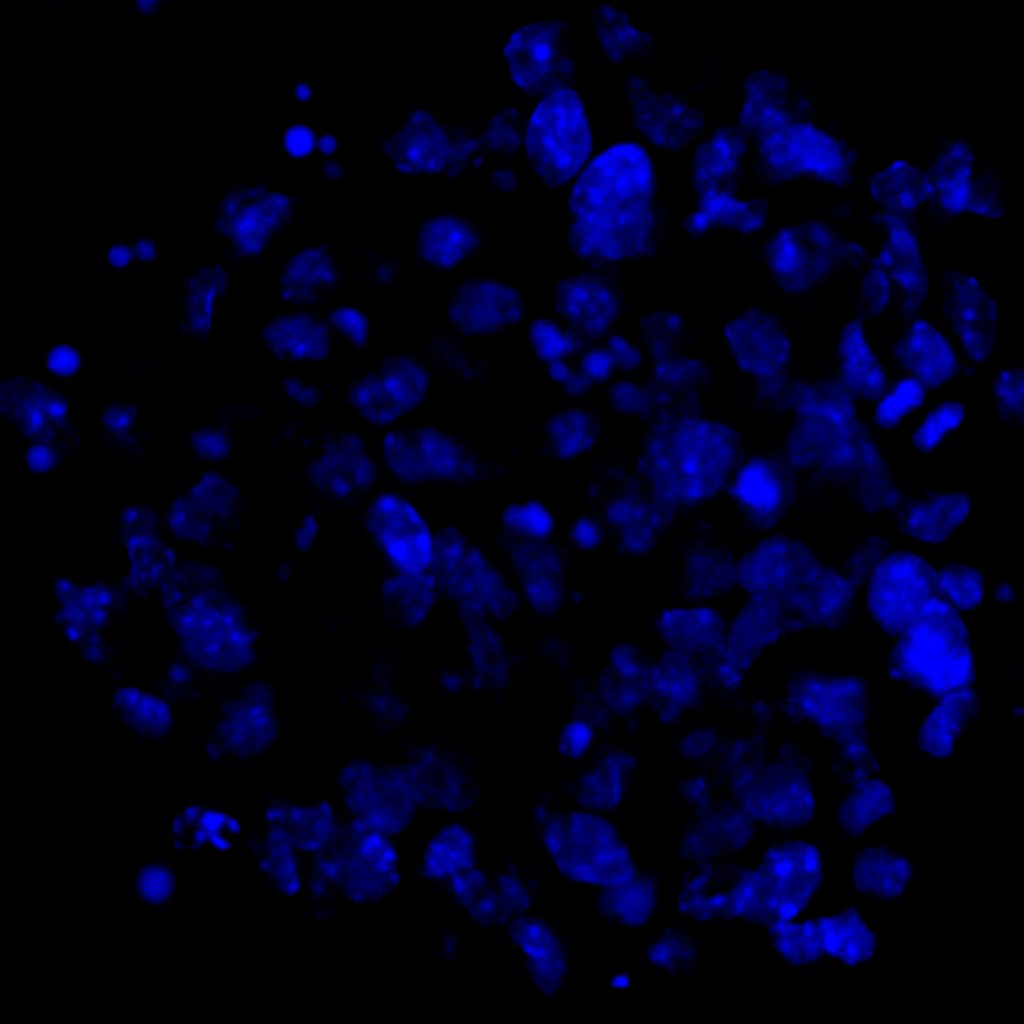

Supplement: S6 File — Representative images in the revised S2C Fig were generated from raw files woGF_4_ch1/2 (W/O GF); EF10cyc_4_ch1/2 (E+F10+Cyc); EF1shh_7_ch1/2 (E+F1+Shh). (ZIP) [file pone.0239995.s007.zip › S6_File/EF1shh_8_ch2.jpg]

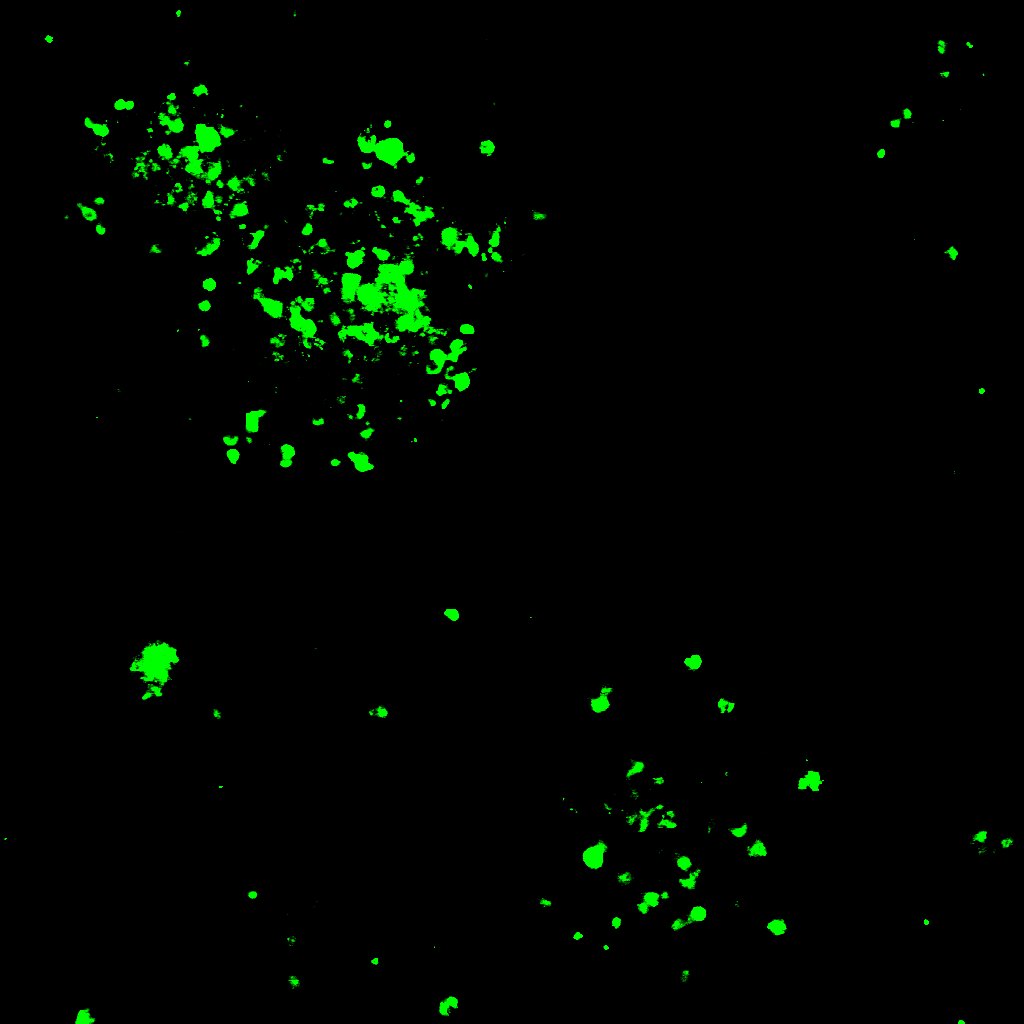

Supplement: S6 File — Representative images in the revised S2C Fig were generated from raw files woGF_4_ch1/2 (W/O GF); EF10cyc_4_ch1/2 (E+F10+Cyc); EF1shh_7_ch1/2 (E+F1+Shh). (ZIP) [file pone.0239995.s007.zip › S6_File/EF1shh_9_ch1.jpg]

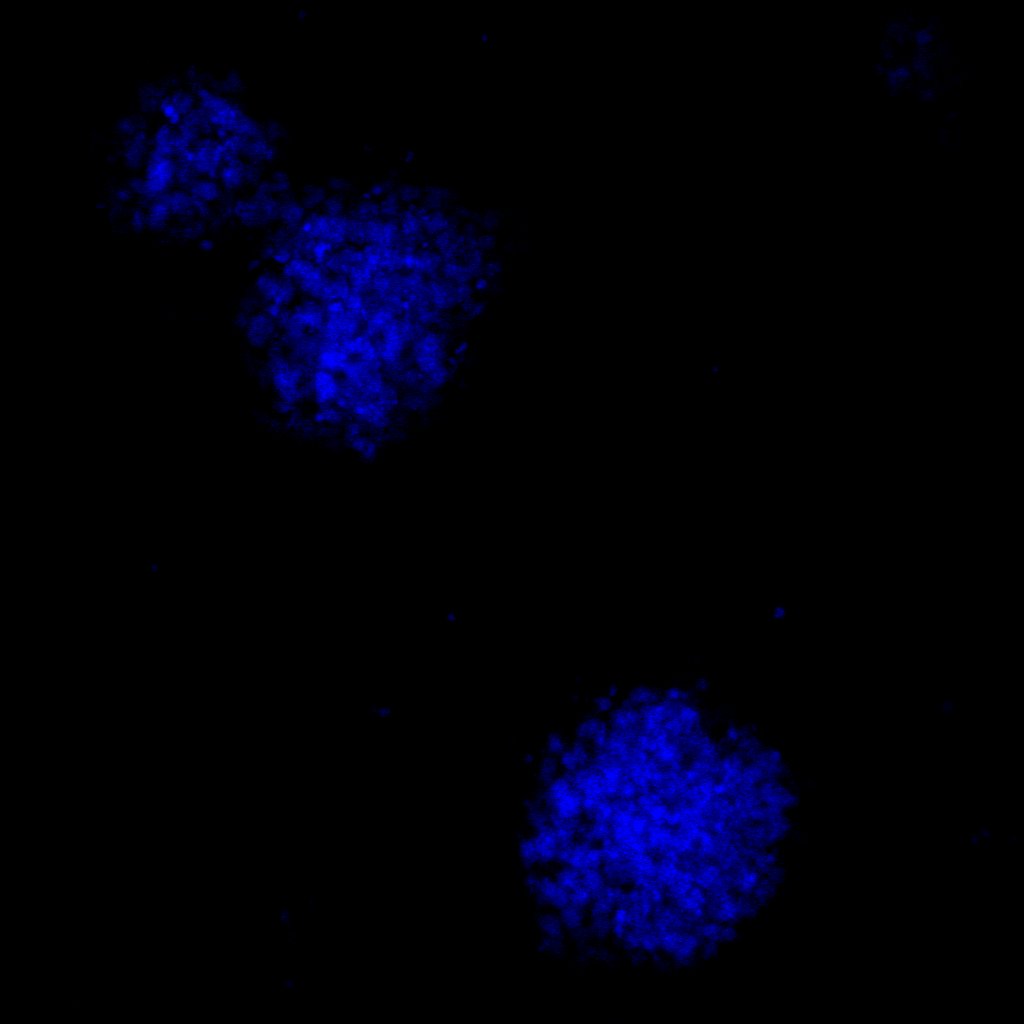

Supplement: S6 File — Representative images in the revised S2C Fig were generated from raw files woGF_4_ch1/2 (W/O GF); EF10cyc_4_ch1/2 (E+F10+Cyc); EF1shh_7_ch1/2 (E+F1+Shh). (ZIP) [file pone.0239995.s007.zip › S6_File/EF1shh_9_ch2.jpg]

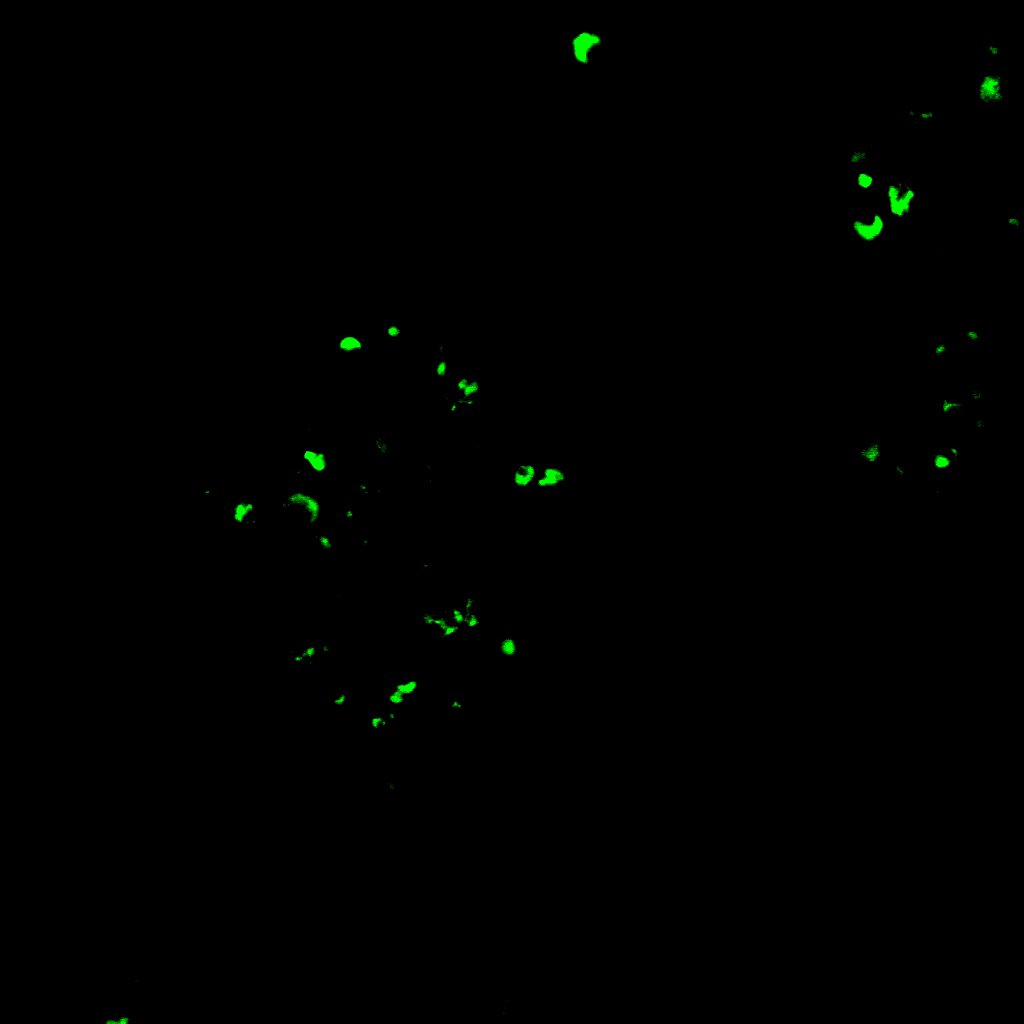

Supplement: S6 File — Representative images in the revised S2C Fig were generated from raw files woGF_4_ch1/2 (W/O GF); EF10cyc_4_ch1/2 (E+F10+Cyc); EF1shh_7_ch1/2 (E+F1+Shh). (ZIP) [file pone.0239995.s007.zip › S6_File/shh_1_ch1.jpg]

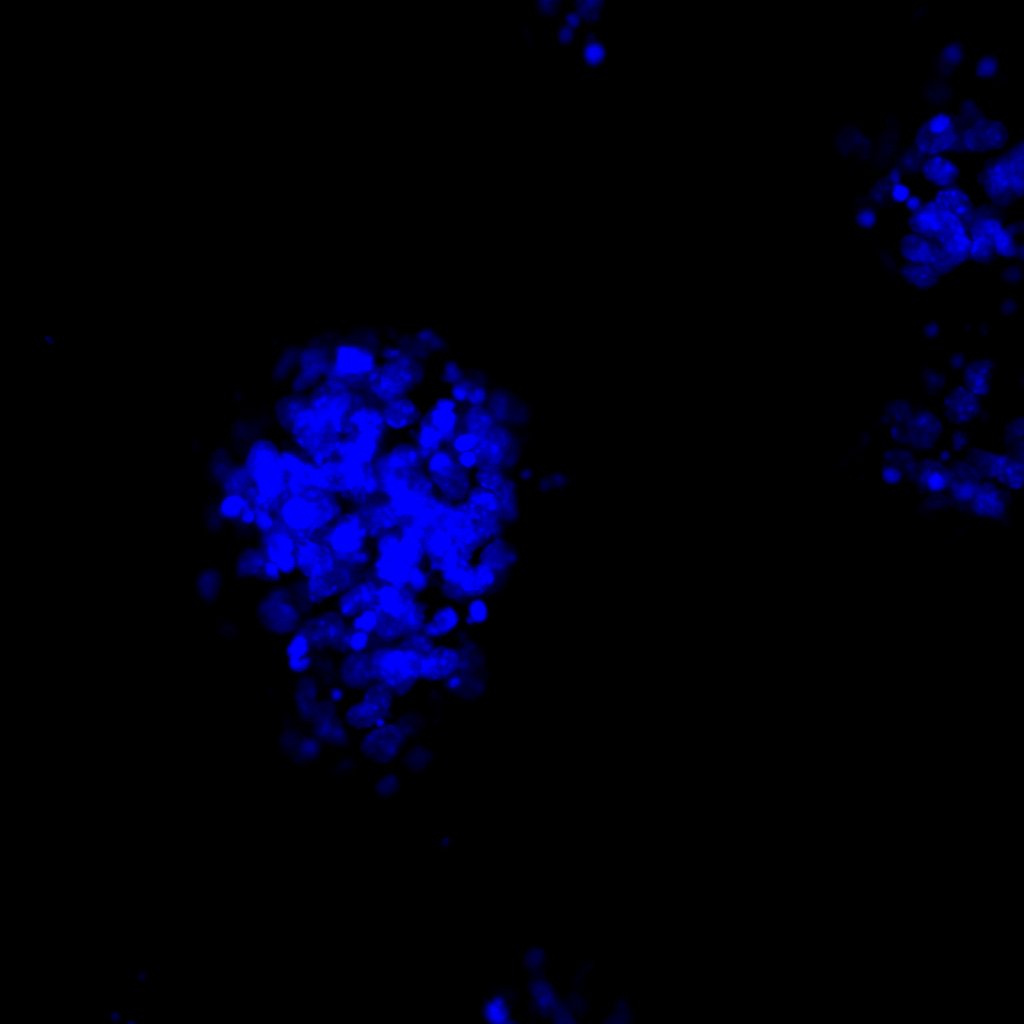

Supplement: S6 File — Representative images in the revised S2C Fig were generated from raw files woGF_4_ch1/2 (W/O GF); EF10cyc_4_ch1/2 (E+F10+Cyc); EF1shh_7_ch1/2 (E+F1+Shh). (ZIP) [file pone.0239995.s007.zip › S6_File/shh_1_ch2.jpg]

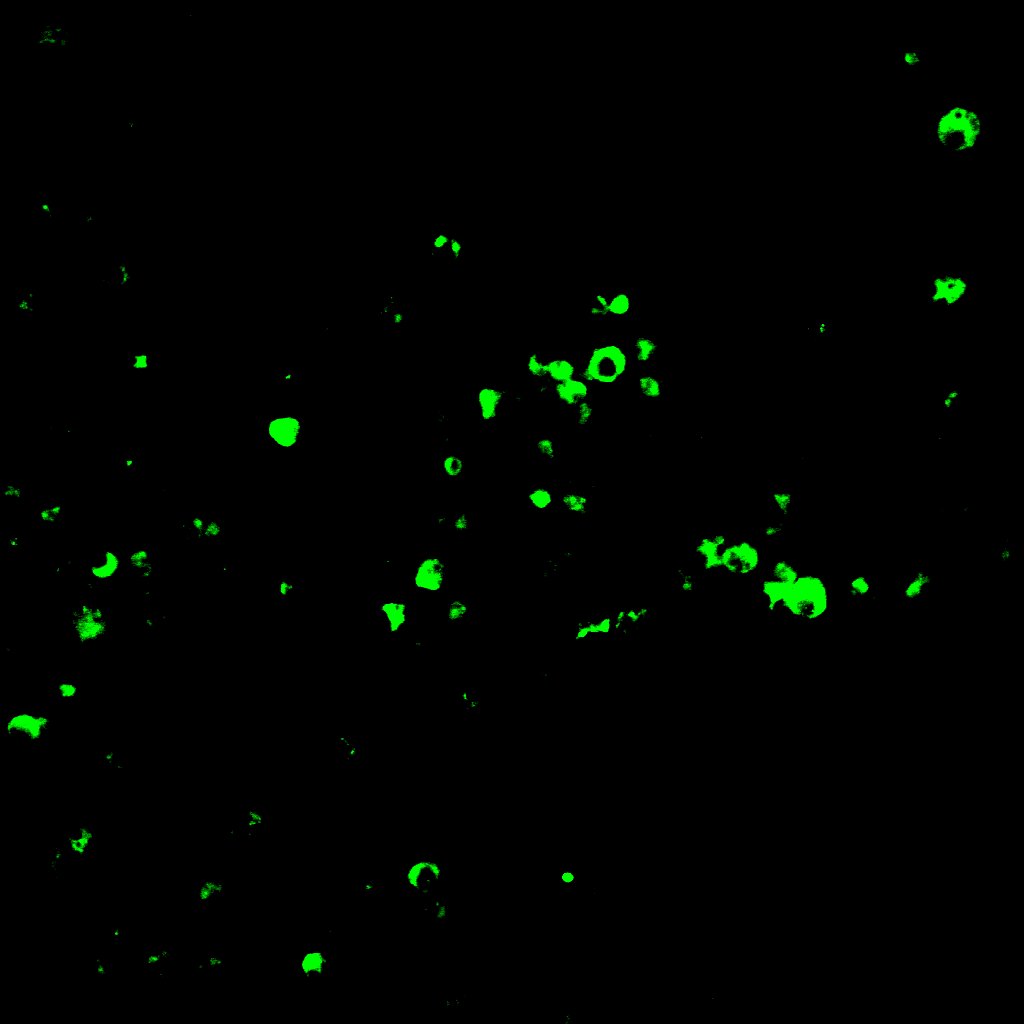

Supplement: S6 File — Representative images in the revised S2C Fig were generated from raw files woGF_4_ch1/2 (W/O GF); EF10cyc_4_ch1/2 (E+F10+Cyc); EF1shh_7_ch1/2 (E+F1+Shh). (ZIP) [file pone.0239995.s007.zip › S6_File/shh_2_ch1.jpg]

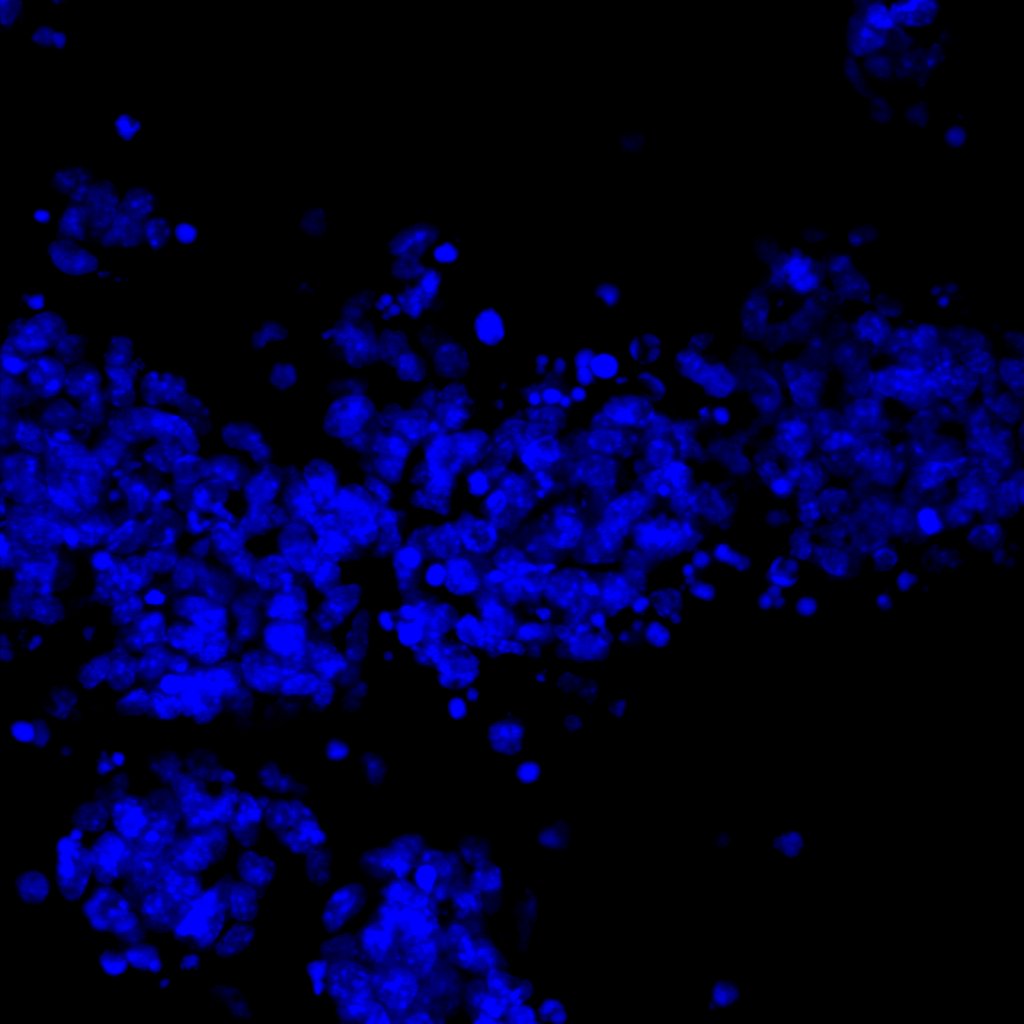

Supplement: S6 File — Representative images in the revised S2C Fig were generated from raw files woGF_4_ch1/2 (W/O GF); EF10cyc_4_ch1/2 (E+F10+Cyc); EF1shh_7_ch1/2 (E+F1+Shh). (ZIP) [file pone.0239995.s007.zip › S6_File/shh_2_ch2.jpg]

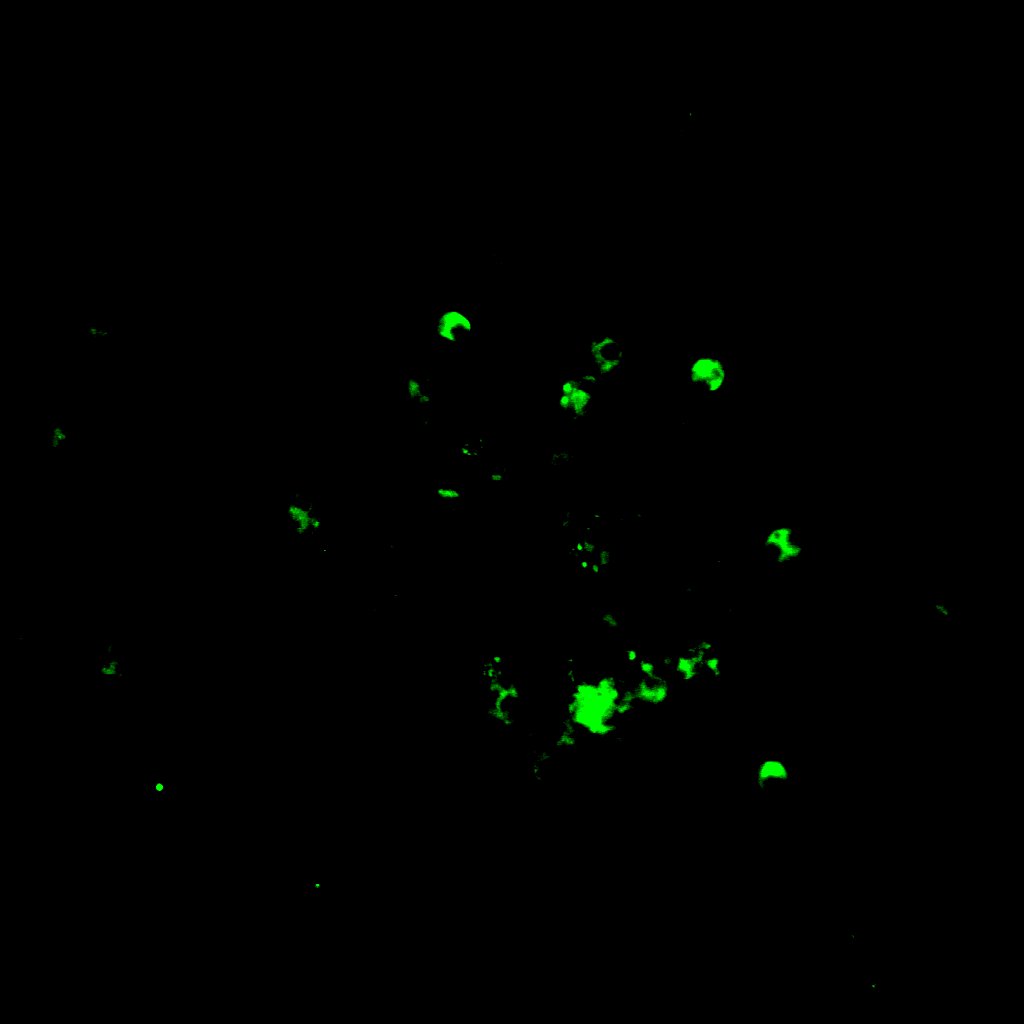

Supplement: S6 File — Representative images in the revised S2C Fig were generated from raw files woGF_4_ch1/2 (W/O GF); EF10cyc_4_ch1/2 (E+F10+Cyc); EF1shh_7_ch1/2 (E+F1+Shh). (ZIP) [file pone.0239995.s007.zip › S6_File/shh_3_ch1.jpg]

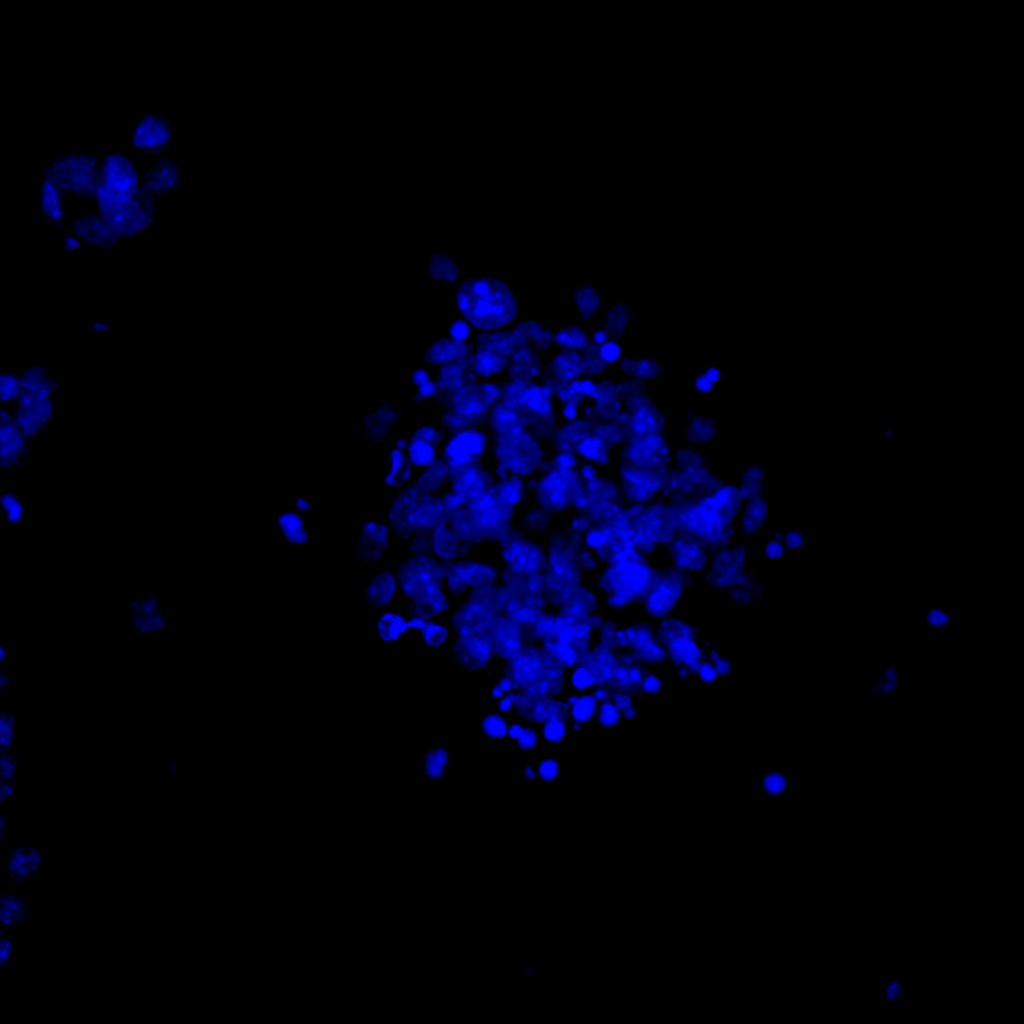

Supplement: S6 File — Representative images in the revised S2C Fig were generated from raw files woGF_4_ch1/2 (W/O GF); EF10cyc_4_ch1/2 (E+F10+Cyc); EF1shh_7_ch1/2 (E+F1+Shh). (ZIP) [file pone.0239995.s007.zip › S6_File/shh_3_ch2.jpg]

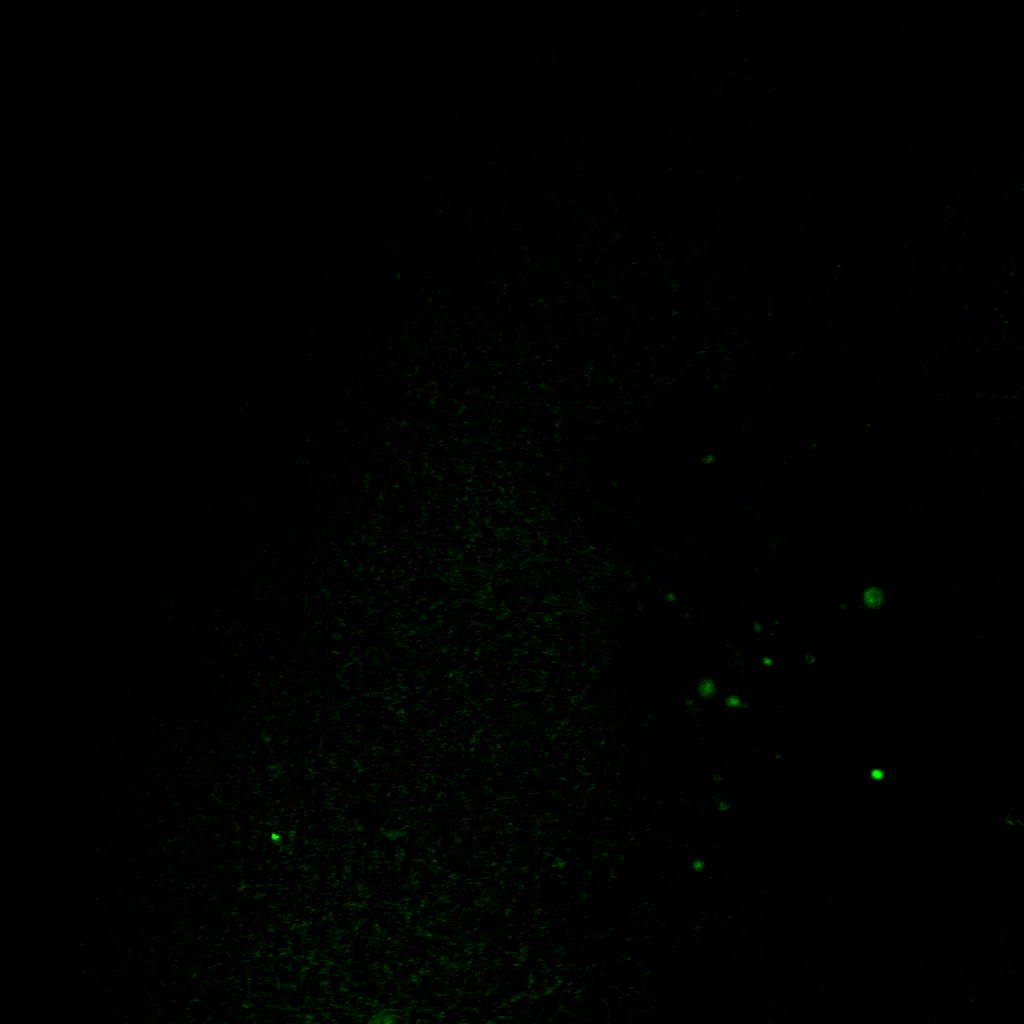

Supplement: S6 File — Representative images in the revised S2C Fig were generated from raw files woGF_4_ch1/2 (W/O GF); EF10cyc_4_ch1/2 (E+F10+Cyc); EF1shh_7_ch1/2 (E+F1+Shh). (ZIP) [file pone.0239995.s007.zip › S6_File/shh_4_ch1.jpg]

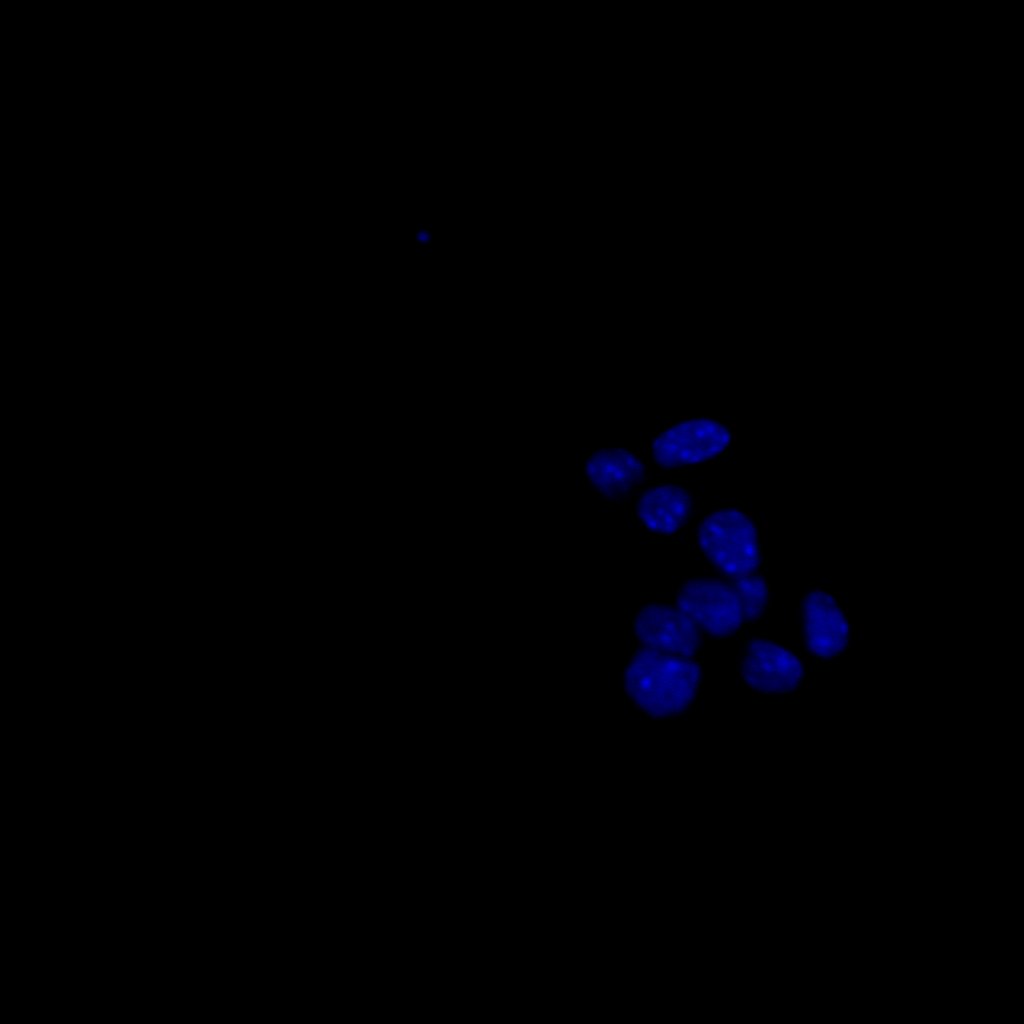

Supplement: S6 File — Representative images in the revised S2C Fig were generated from raw files woGF_4_ch1/2 (W/O GF); EF10cyc_4_ch1/2 (E+F10+Cyc); EF1shh_7_ch1/2 (E+F1+Shh). (ZIP) [file pone.0239995.s007.zip › S6_File/shh_4_ch2.jpg]

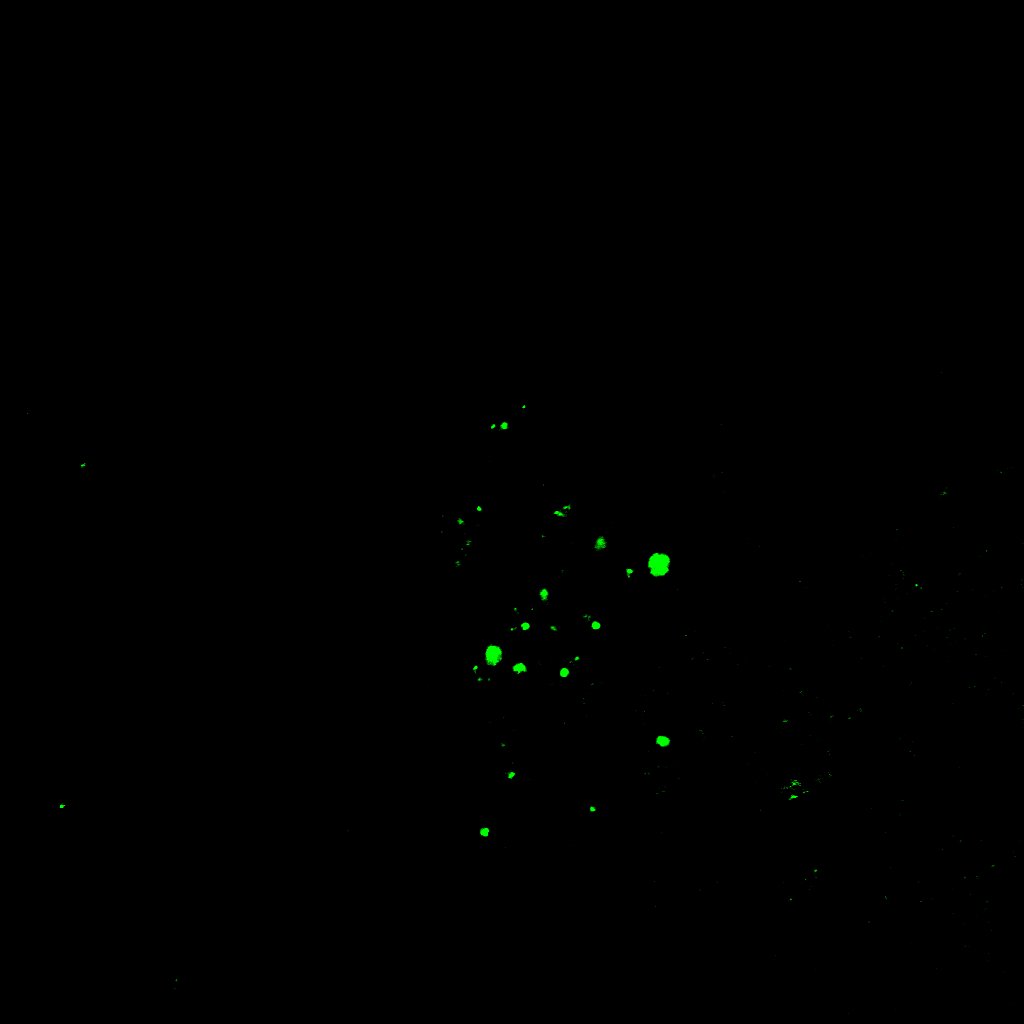

Supplement: S6 File — Representative images in the revised S2C Fig were generated from raw files woGF_4_ch1/2 (W/O GF); EF10cyc_4_ch1/2 (E+F10+Cyc); EF1shh_7_ch1/2 (E+F1+Shh). (ZIP) [file pone.0239995.s007.zip › S6_File/shh_5_ch1.jpg]

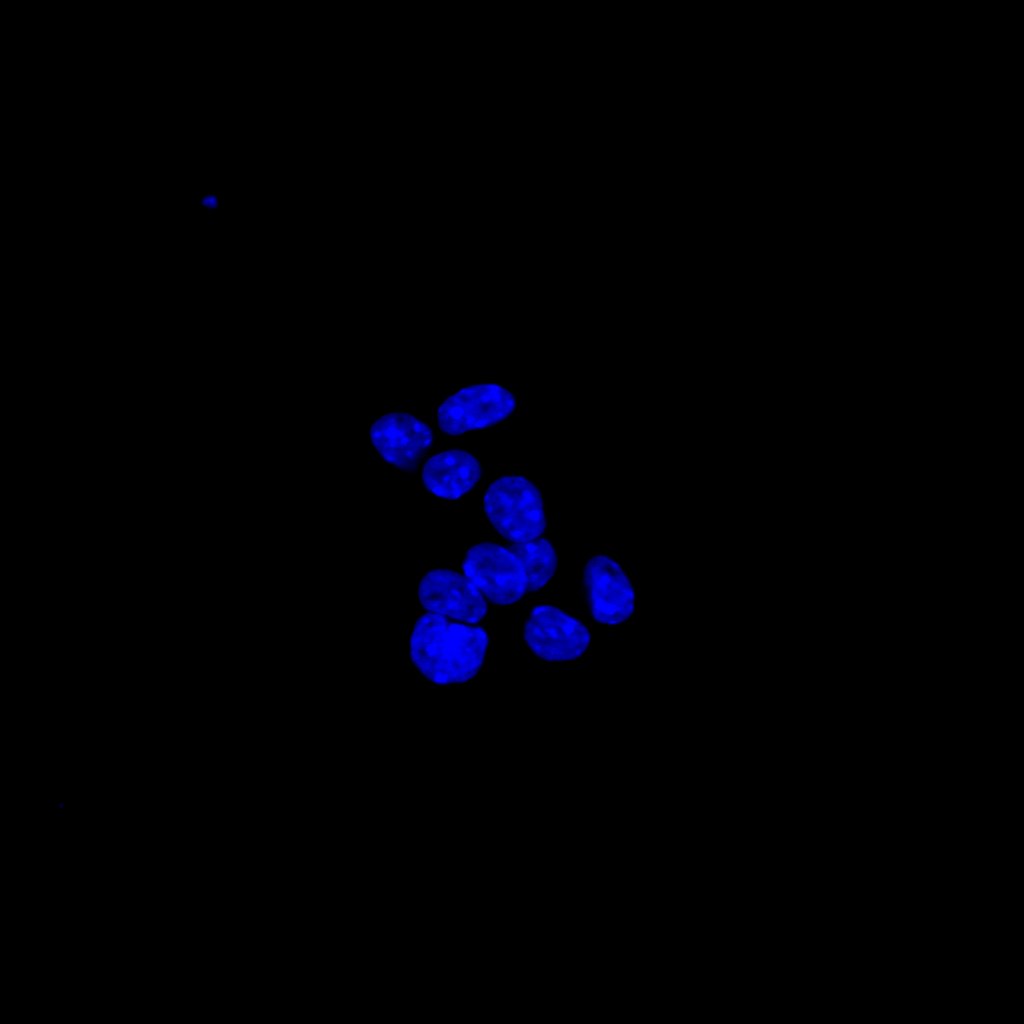

Supplement: S6 File — Representative images in the revised S2C Fig were generated from raw files woGF_4_ch1/2 (W/O GF); EF10cyc_4_ch1/2 (E+F10+Cyc); EF1shh_7_ch1/2 (E+F1+Shh). (ZIP) [file pone.0239995.s007.zip › S6_File/shh_5_ch2.jpg]

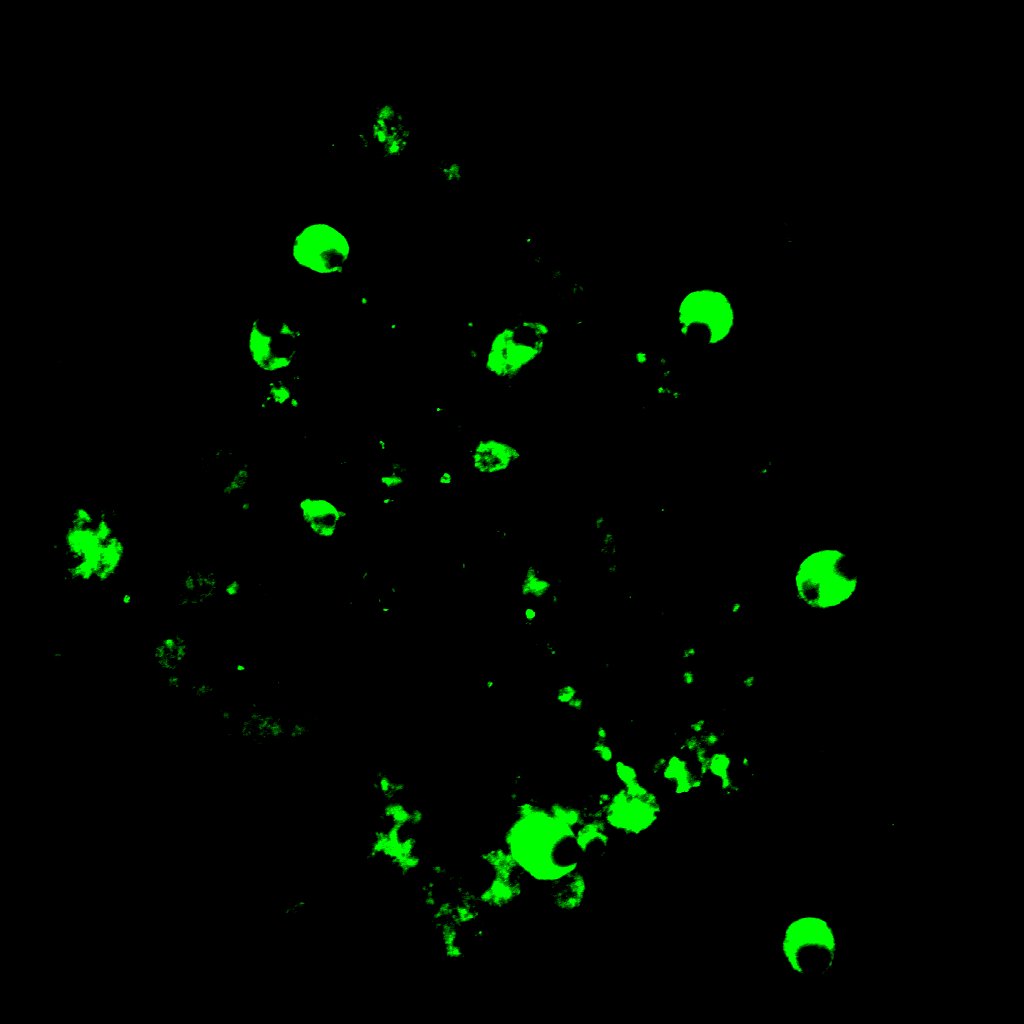

Supplement: S6 File — Representative images in the revised S2C Fig were generated from raw files woGF_4_ch1/2 (W/O GF); EF10cyc_4_ch1/2 (E+F10+Cyc); EF1shh_7_ch1/2 (E+F1+Shh). (ZIP) [file pone.0239995.s007.zip › S6_File/shh_6_ch1.jpg]

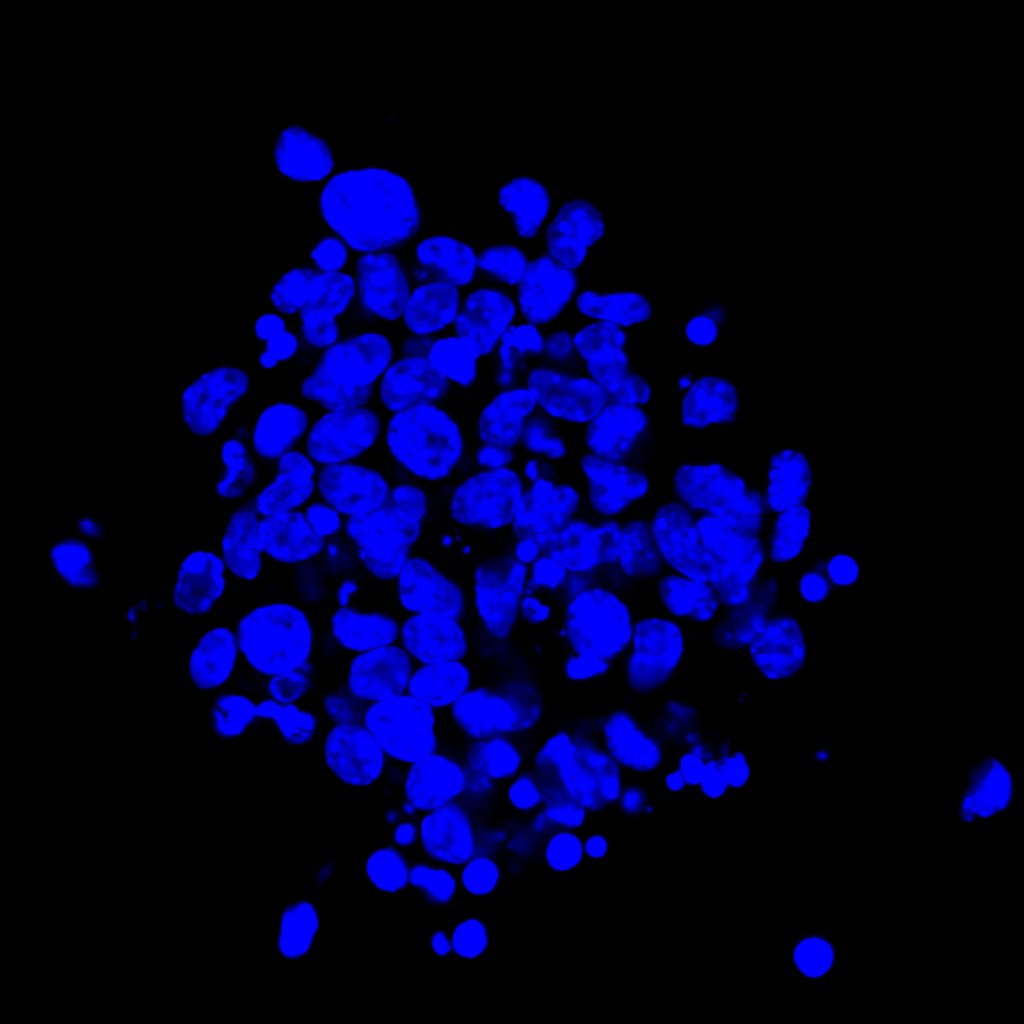

Supplement: S6 File — Representative images in the revised S2C Fig were generated from raw files woGF_4_ch1/2 (W/O GF); EF10cyc_4_ch1/2 (E+F10+Cyc); EF1shh_7_ch1/2 (E+F1+Shh). (ZIP) [file pone.0239995.s007.zip › S6_File/shh_6_ch2.jpg]

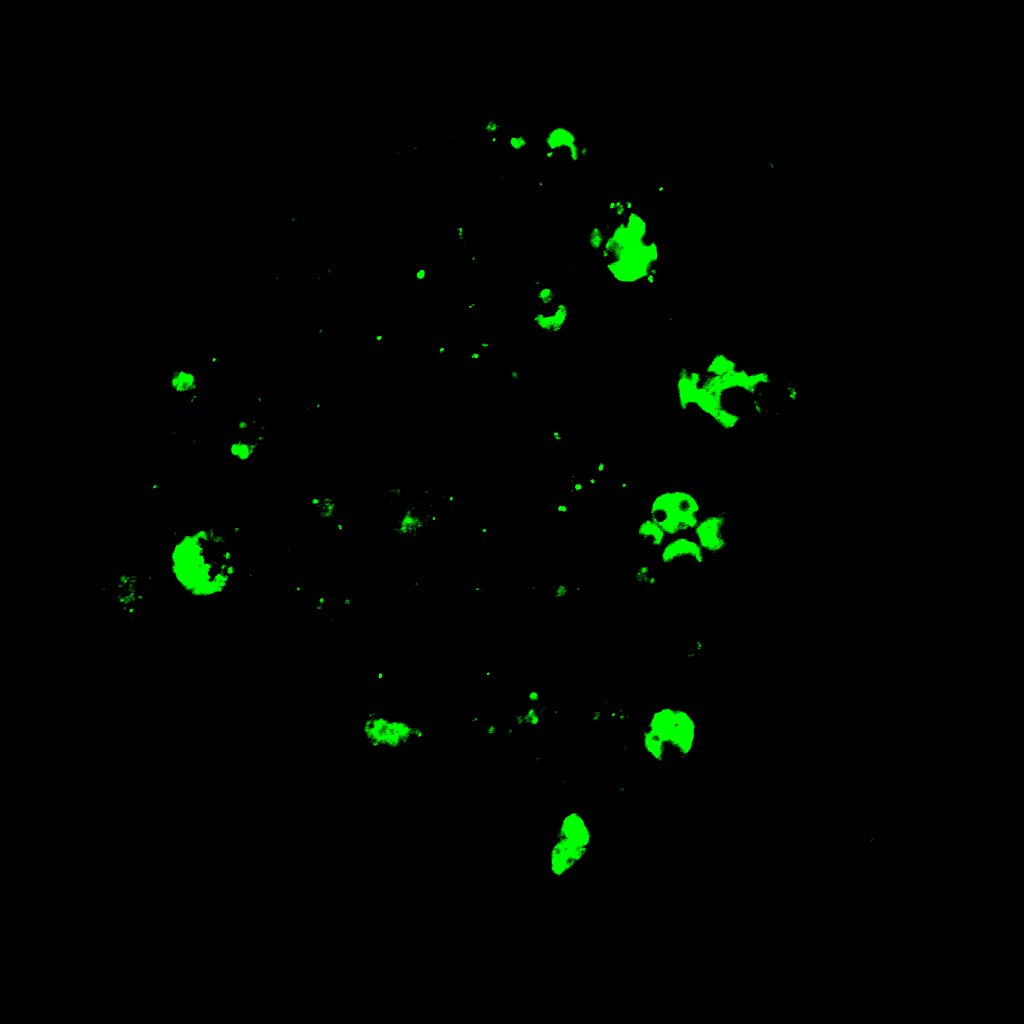

Supplement: S6 File — Representative images in the revised S2C Fig were generated from raw files woGF_4_ch1/2 (W/O GF); EF10cyc_4_ch1/2 (E+F10+Cyc); EF1shh_7_ch1/2 (E+F1+Shh). (ZIP) [file pone.0239995.s007.zip › S6_File/shh_7_ch1.jpg]

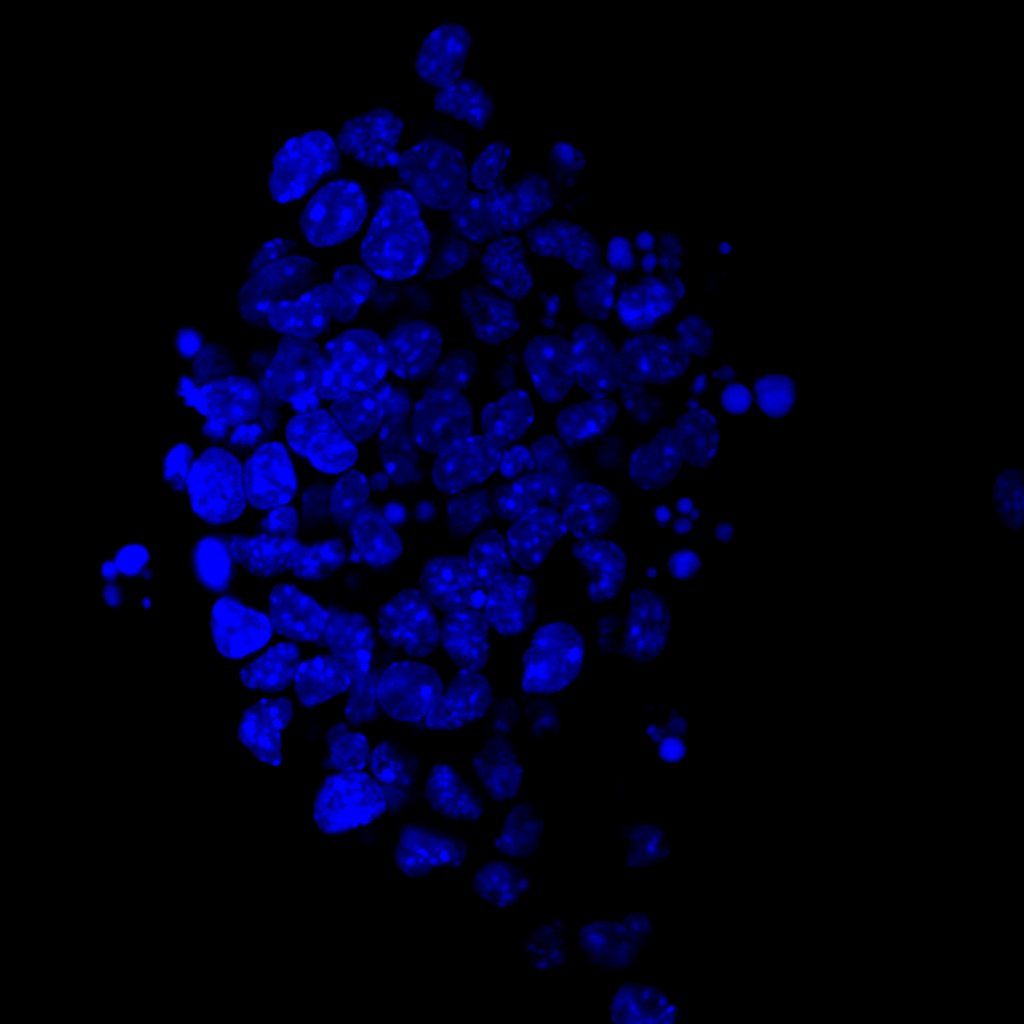

Supplement: S6 File — Representative images in the revised S2C Fig were generated from raw files woGF_4_ch1/2 (W/O GF); EF10cyc_4_ch1/2 (E+F10+Cyc); EF1shh_7_ch1/2 (E+F1+Shh). (ZIP) [file pone.0239995.s007.zip › S6_File/shh_7_ch2.jpg]

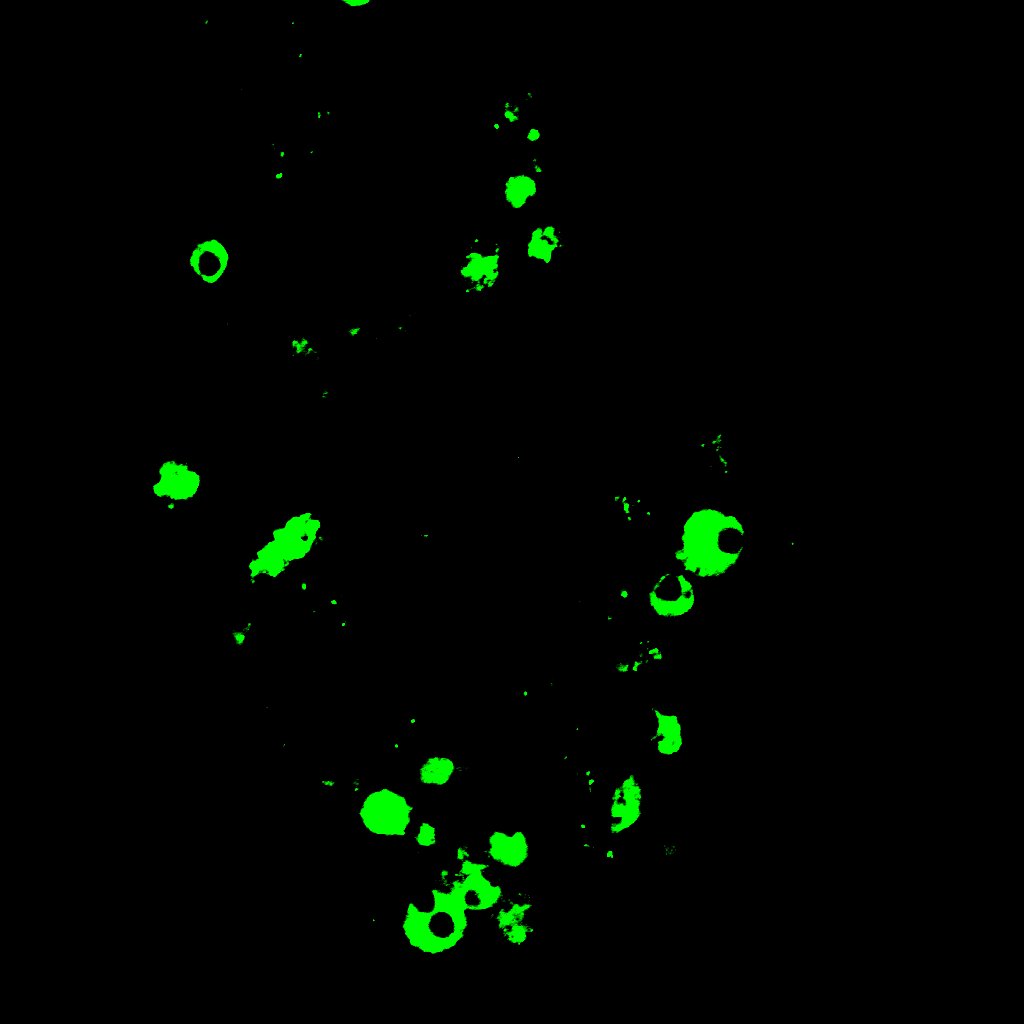

Supplement: S6 File — Representative images in the revised S2C Fig were generated from raw files woGF_4_ch1/2 (W/O GF); EF10cyc_4_ch1/2 (E+F10+Cyc); EF1shh_7_ch1/2 (E+F1+Shh). (ZIP) [file pone.0239995.s007.zip › S6_File/shh_8_ch1.jpg]

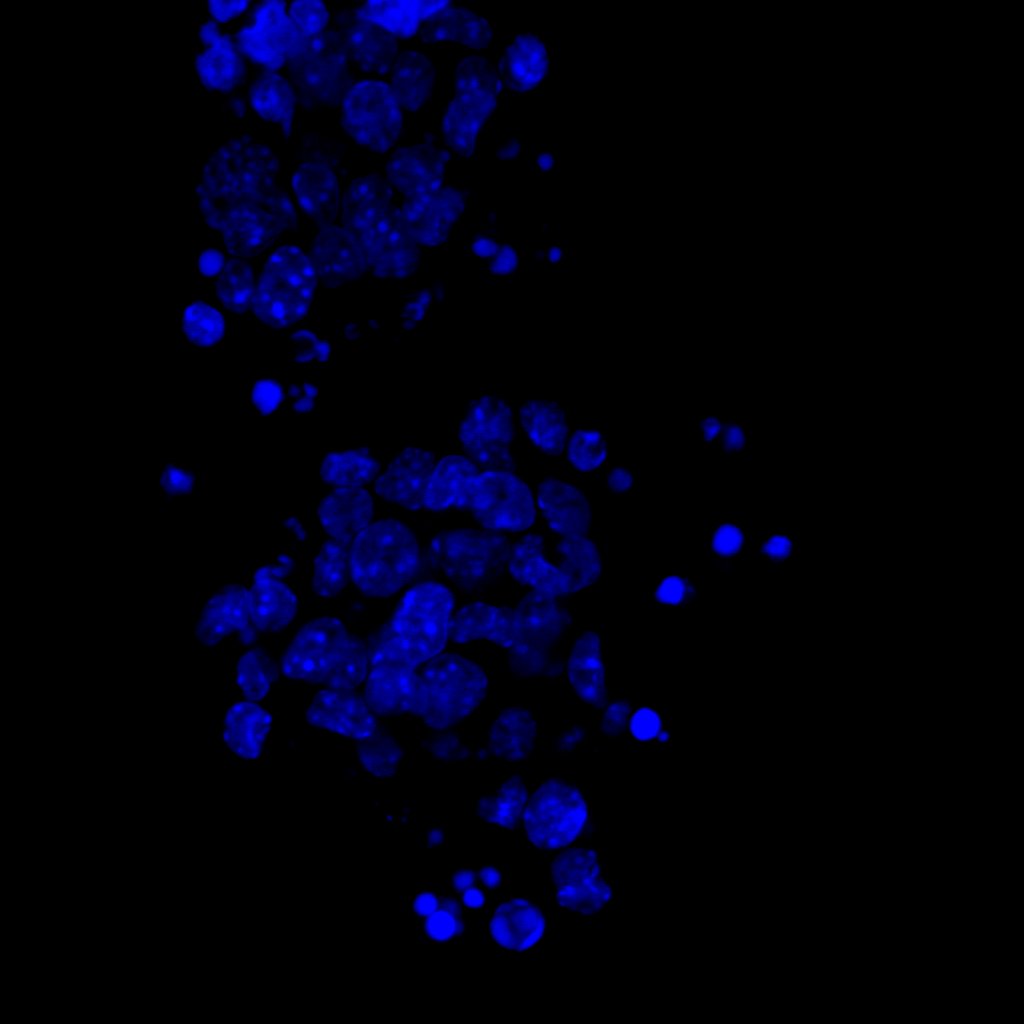

Supplement: S6 File — Representative images in the revised S2C Fig were generated from raw files woGF_4_ch1/2 (W/O GF); EF10cyc_4_ch1/2 (E+F10+Cyc); EF1shh_7_ch1/2 (E+F1+Shh). (ZIP) [file pone.0239995.s007.zip › S6_File/shh_8_ch2.jpg]

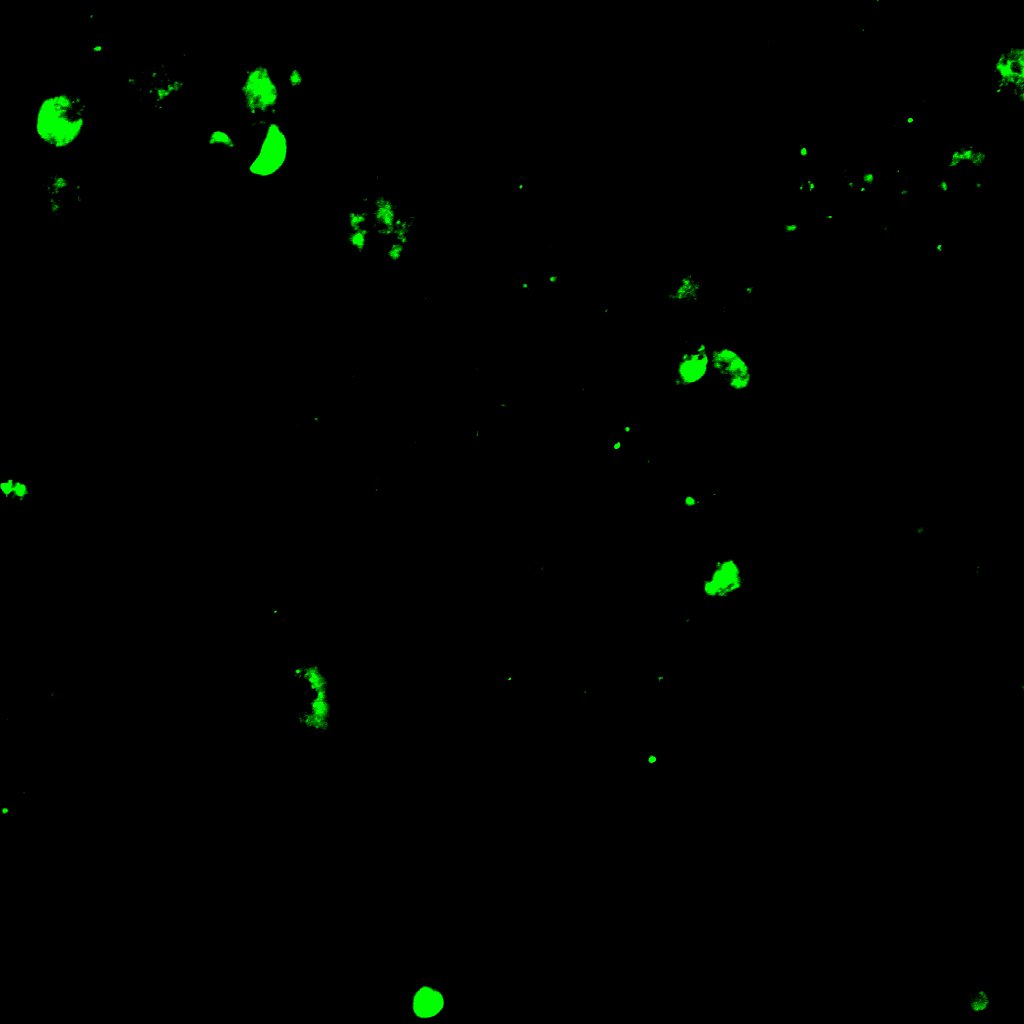

Supplement: S6 File — Representative images in the revised S2C Fig were generated from raw files woGF_4_ch1/2 (W/O GF); EF10cyc_4_ch1/2 (E+F10+Cyc); EF1shh_7_ch1/2 (E+F1+Shh). (ZIP) [file pone.0239995.s007.zip › S6_File/shh_9_ch1.jpg]

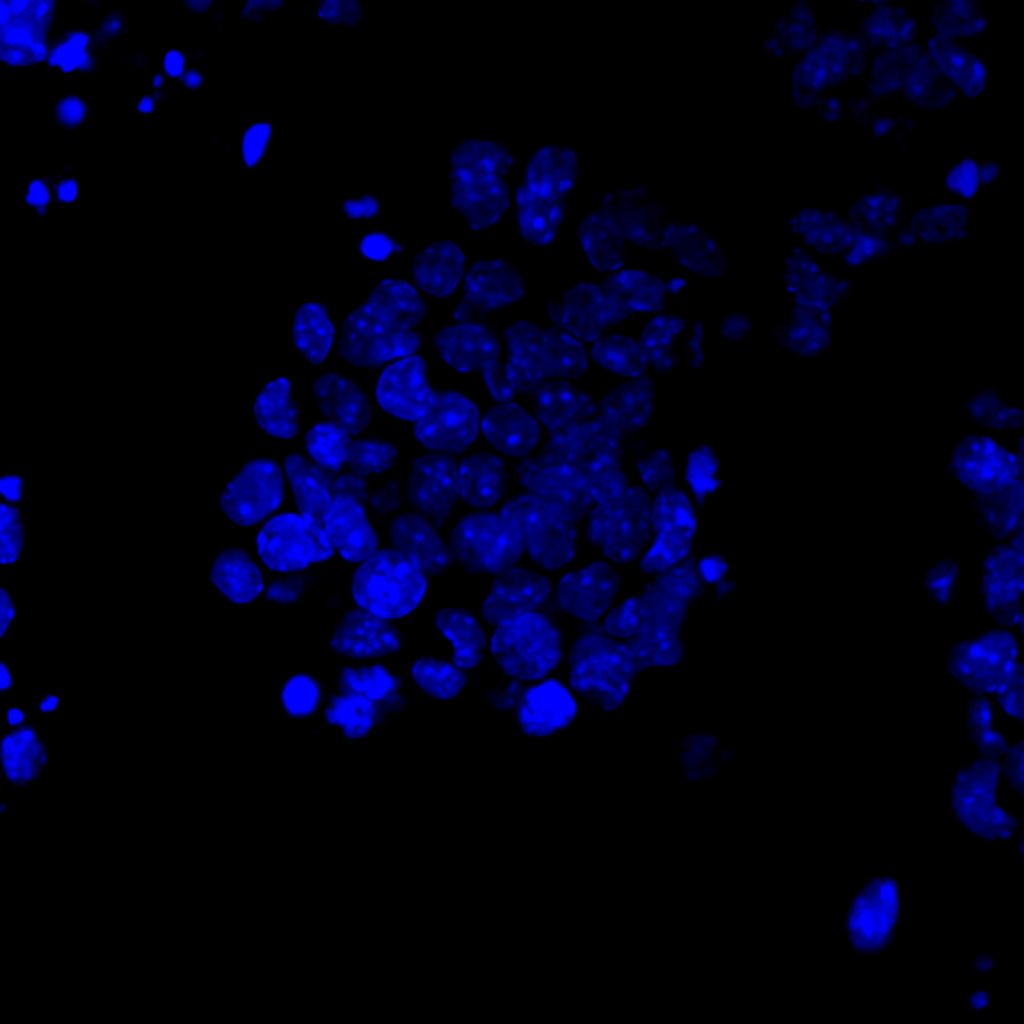

Supplement: S6 File — Representative images in the revised S2C Fig were generated from raw files woGF_4_ch1/2 (W/O GF); EF10cyc_4_ch1/2 (E+F10+Cyc); EF1shh_7_ch1/2 (E+F1+Shh). (ZIP) [file pone.0239995.s007.zip › S6_File/shh_9_ch2.jpg]

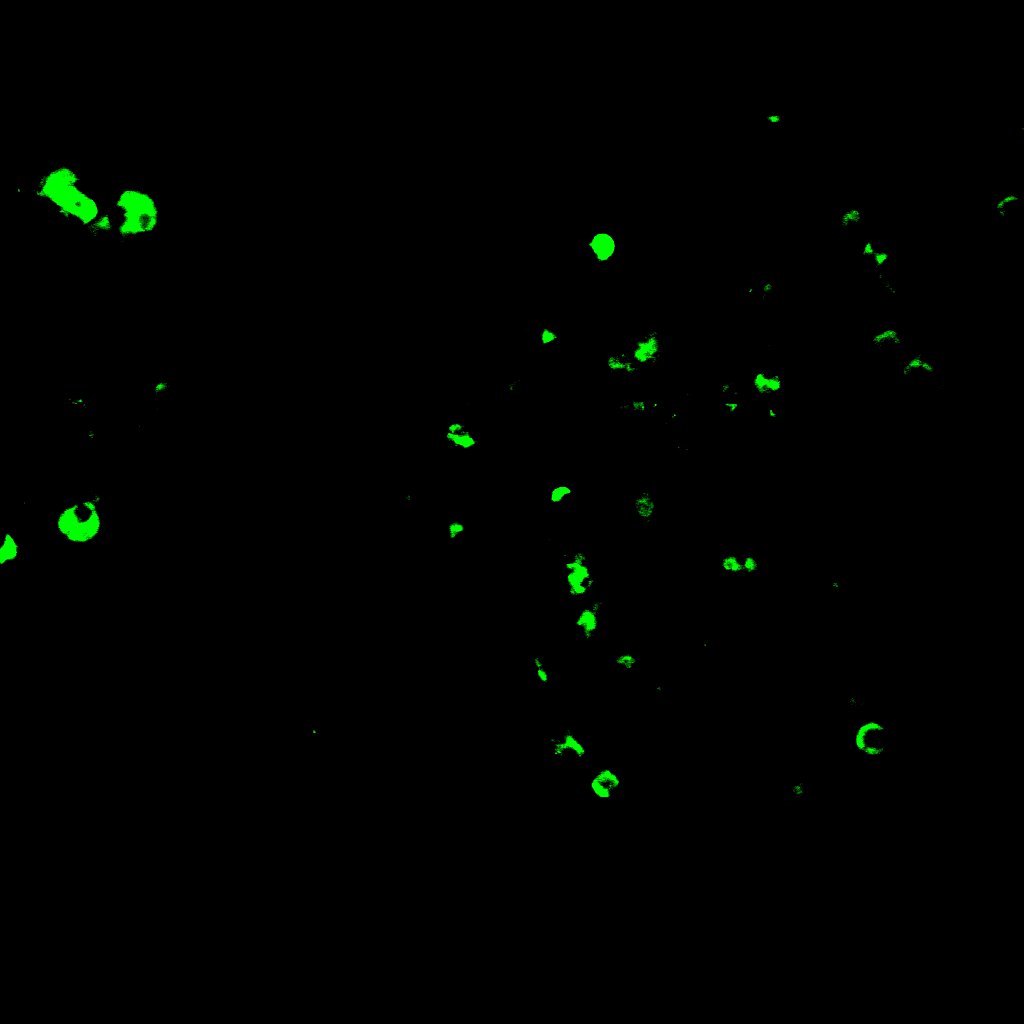

Supplement: S6 File — Representative images in the revised S2C Fig were generated from raw files woGF_4_ch1/2 (W/O GF); EF10cyc_4_ch1/2 (E+F10+Cyc); EF1shh_7_ch1/2 (E+F1+Shh). (ZIP) [file pone.0239995.s007.zip › S6_File/woGF_1_ch1.jpg]

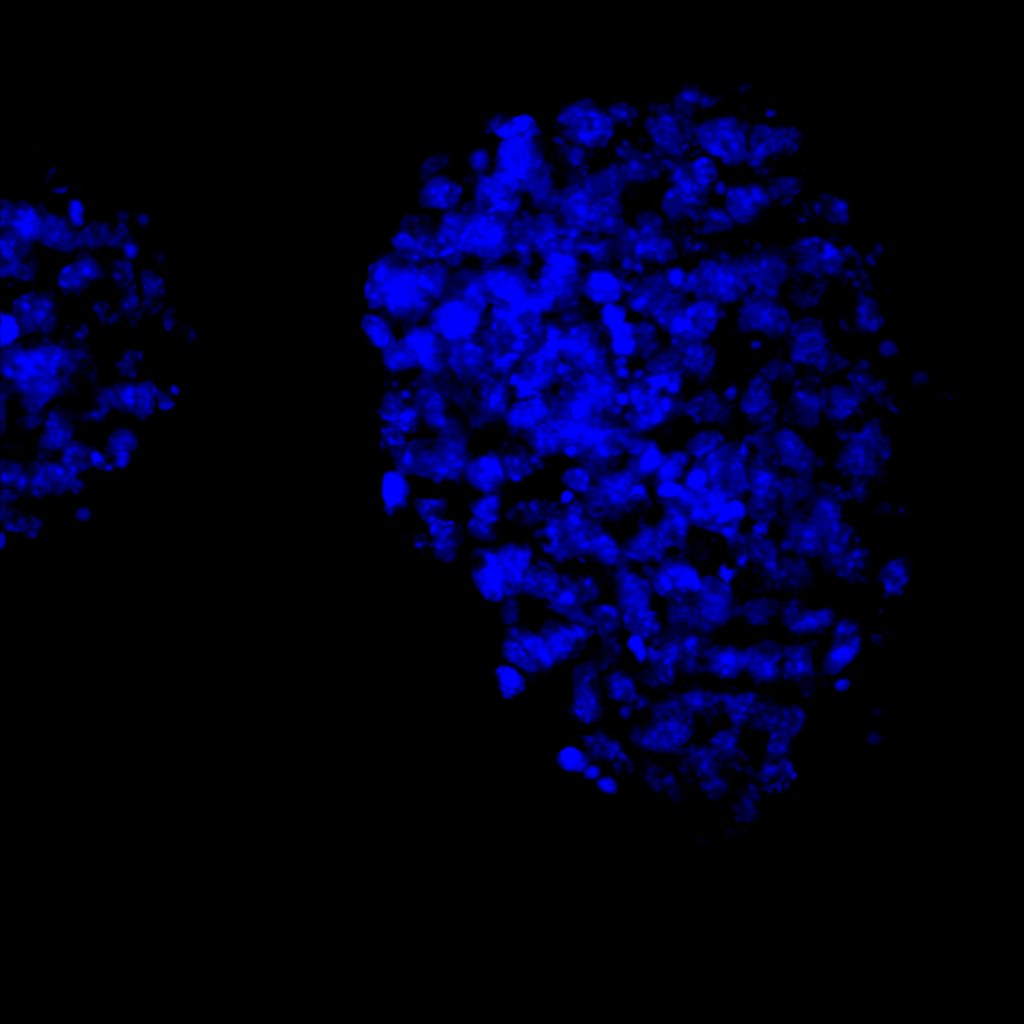

Supplement: S6 File — Representative images in the revised S2C Fig were generated from raw files woGF_4_ch1/2 (W/O GF); EF10cyc_4_ch1/2 (E+F10+Cyc); EF1shh_7_ch1/2 (E+F1+Shh). (ZIP) [file pone.0239995.s007.zip › S6_File/woGF_1_ch2.jpg]

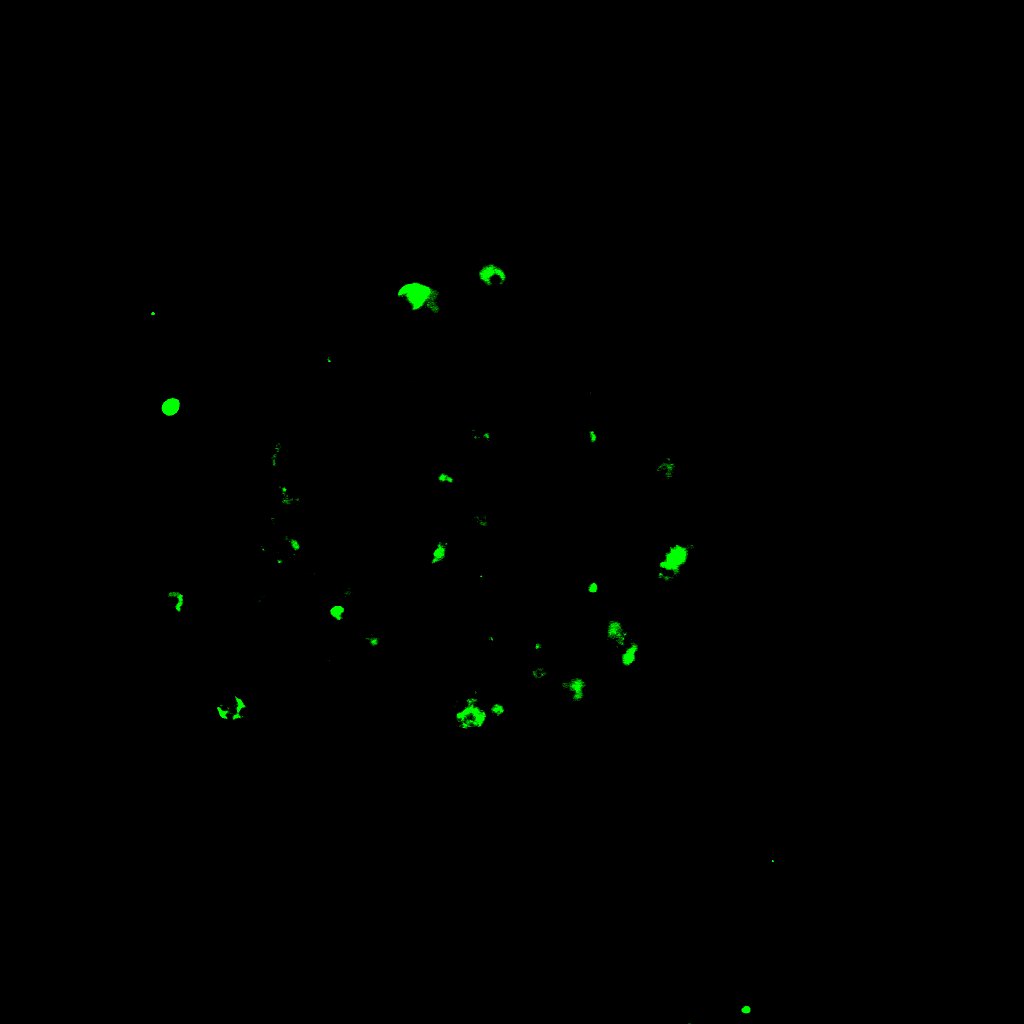

Supplement: S6 File — Representative images in the revised S2C Fig were generated from raw files woGF_4_ch1/2 (W/O GF); EF10cyc_4_ch1/2 (E+F10+Cyc); EF1shh_7_ch1/2 (E+F1+Shh). (ZIP) [file pone.0239995.s007.zip › S6_File/woGF_2_ch1.jpg]

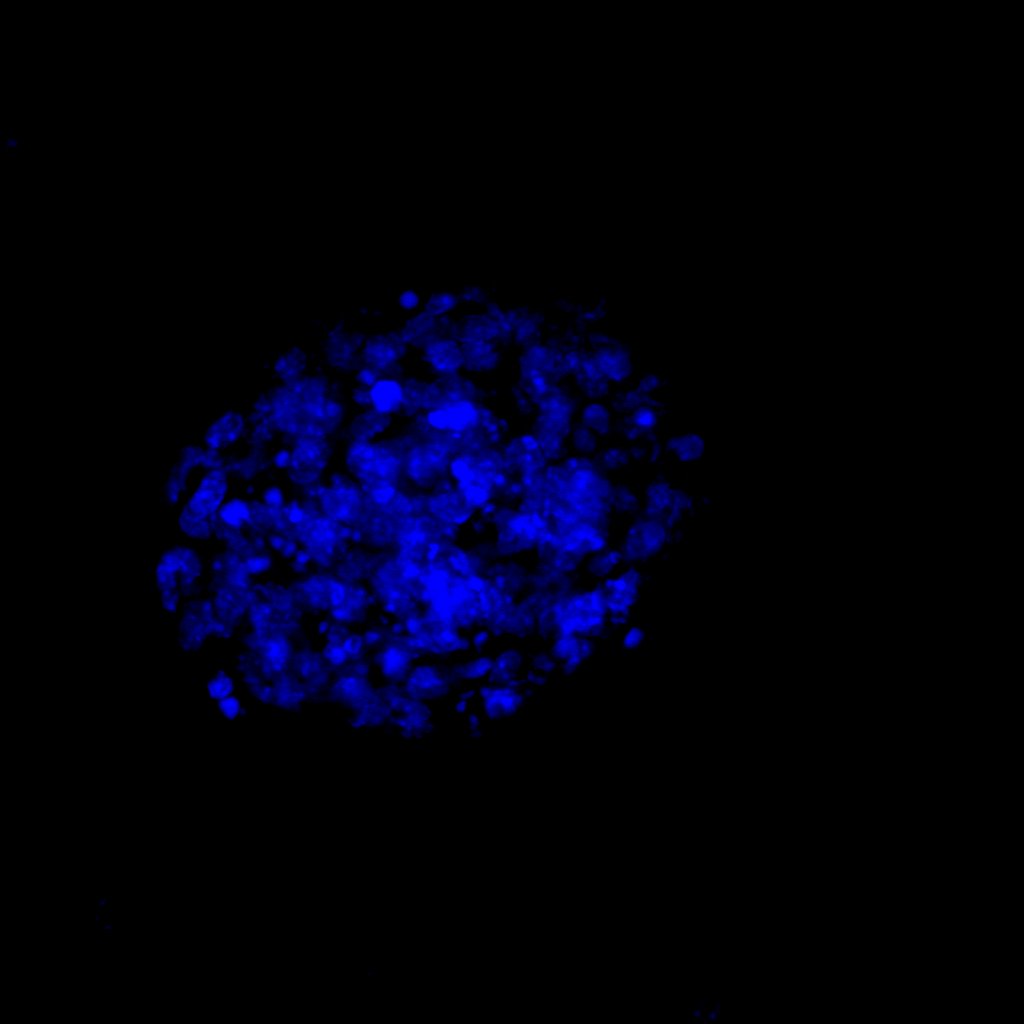

Supplement: S6 File — Representative images in the revised S2C Fig were generated from raw files woGF_4_ch1/2 (W/O GF); EF10cyc_4_ch1/2 (E+F10+Cyc); EF1shh_7_ch1/2 (E+F1+Shh). (ZIP) [file pone.0239995.s007.zip › S6_File/woGF_2_ch2.jpg]

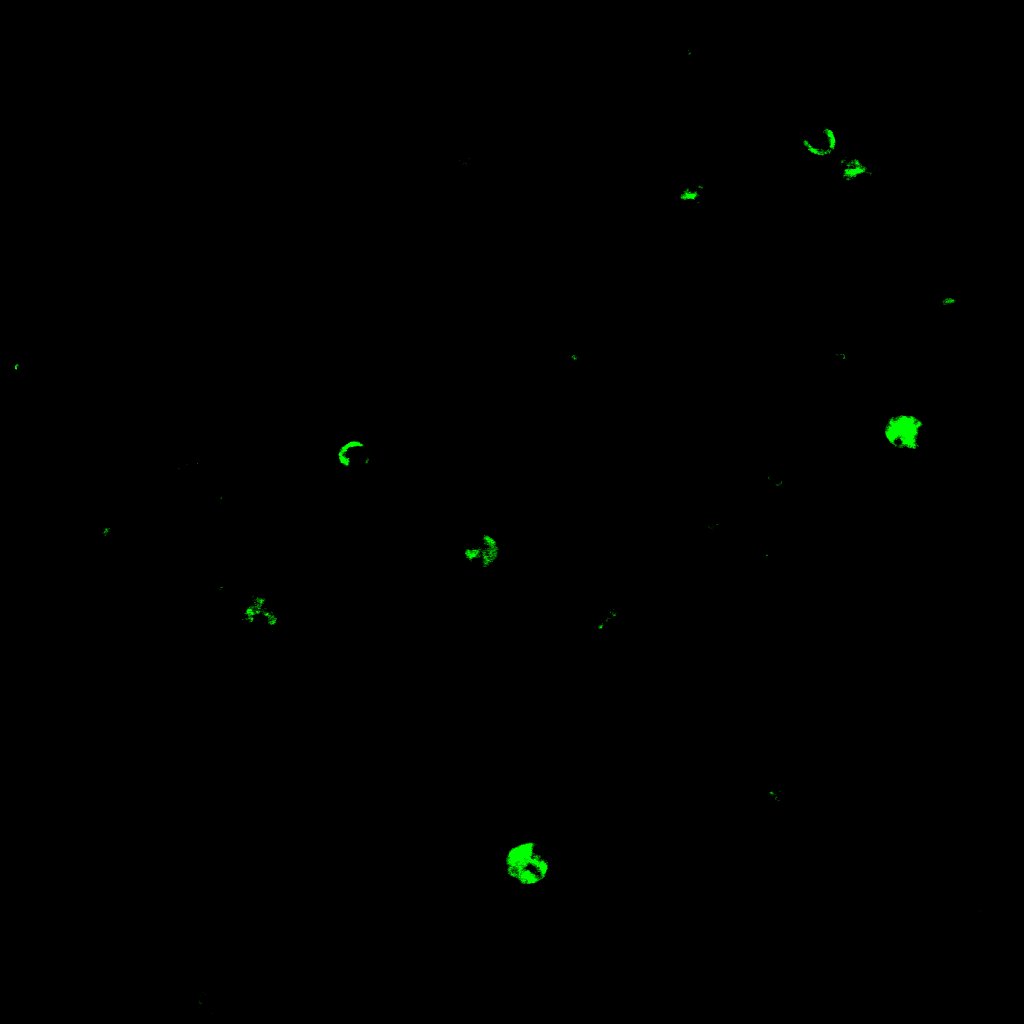

Supplement: S6 File — Representative images in the revised S2C Fig were generated from raw files woGF_4_ch1/2 (W/O GF); EF10cyc_4_ch1/2 (E+F10+Cyc); EF1shh_7_ch1/2 (E+F1+Shh). (ZIP) [file pone.0239995.s007.zip › S6_File/woGF_3_ch1.jpg]

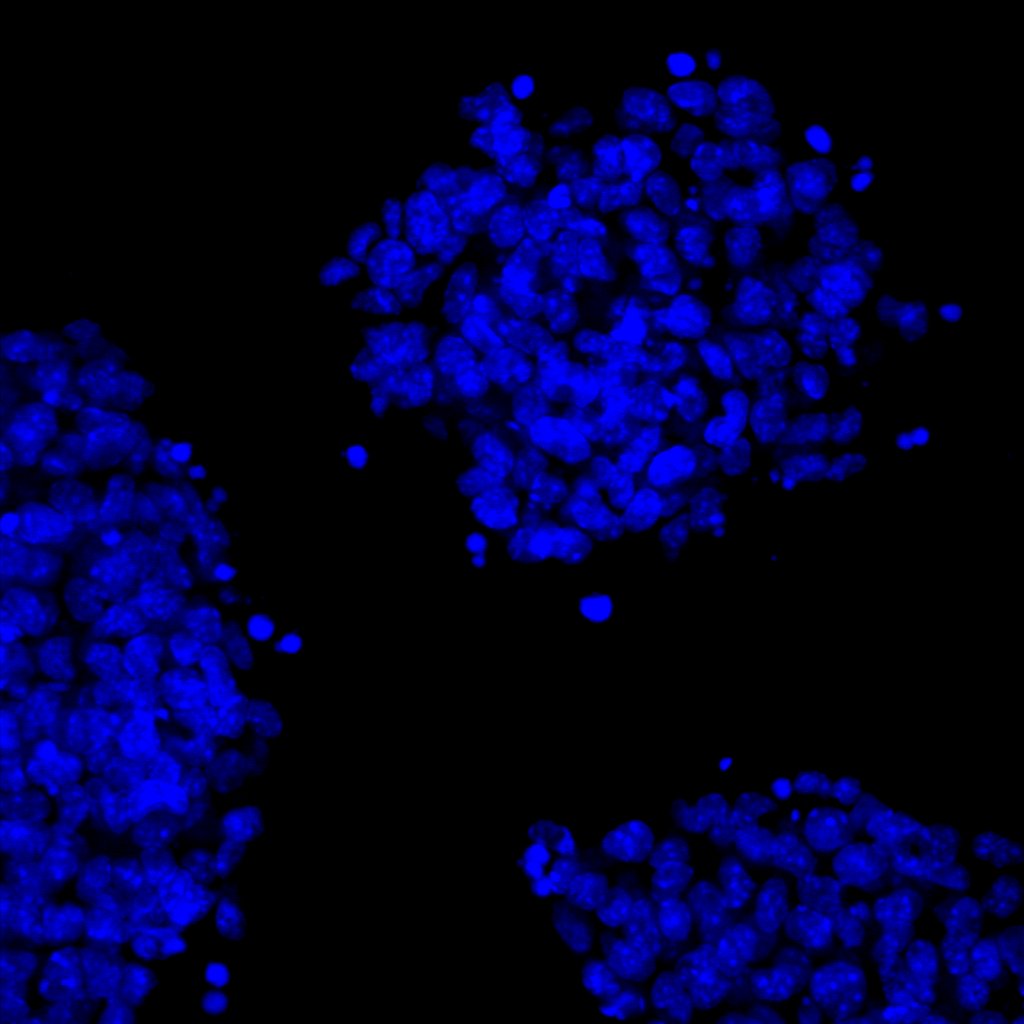

Supplement: S6 File — Representative images in the revised S2C Fig were generated from raw files woGF_4_ch1/2 (W/O GF); EF10cyc_4_ch1/2 (E+F10+Cyc); EF1shh_7_ch1/2 (E+F1+Shh). (ZIP) [file pone.0239995.s007.zip › S6_File/woGF_3_ch2.jpg]

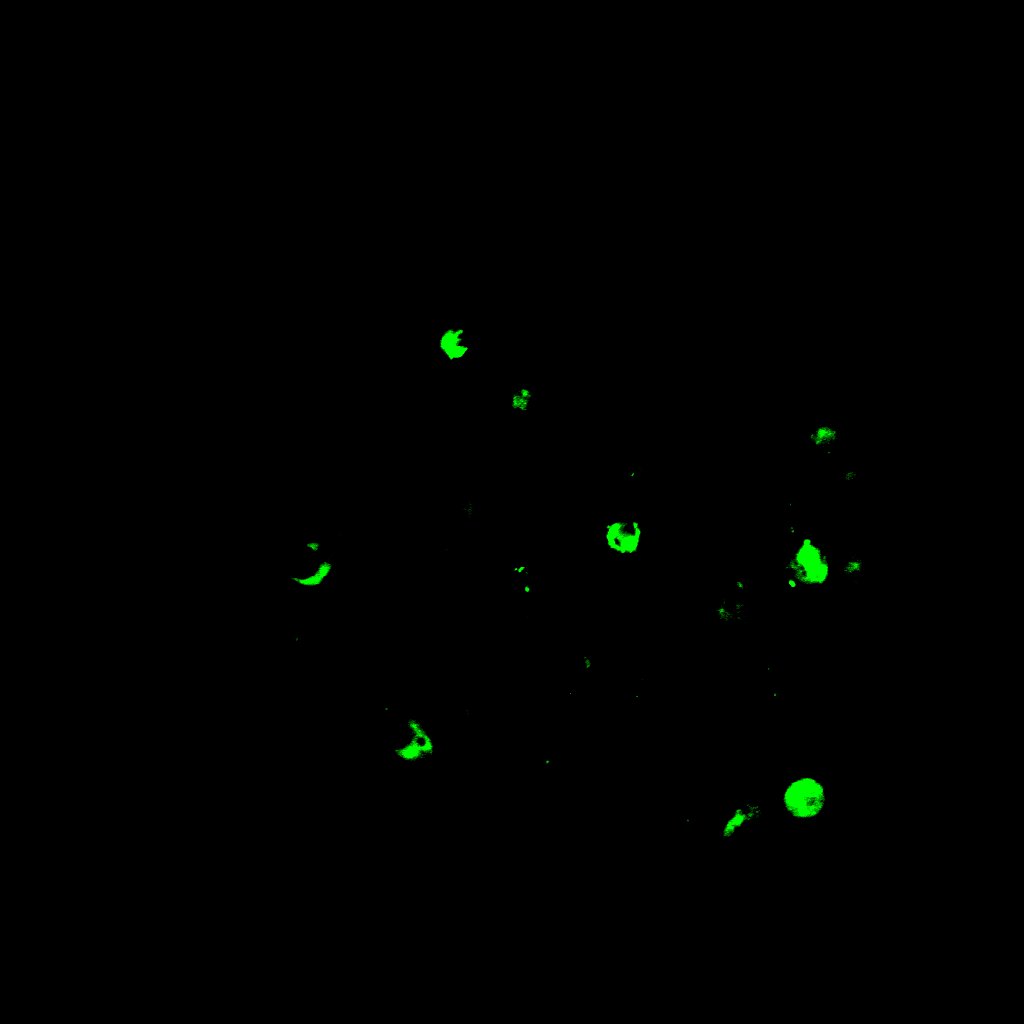

Supplement: S6 File — Representative images in the revised S2C Fig were generated from raw files woGF_4_ch1/2 (W/O GF); EF10cyc_4_ch1/2 (E+F10+Cyc); EF1shh_7_ch1/2 (E+F1+Shh). (ZIP) [file pone.0239995.s007.zip › S6_File/woGF_4_ch1.jpg]

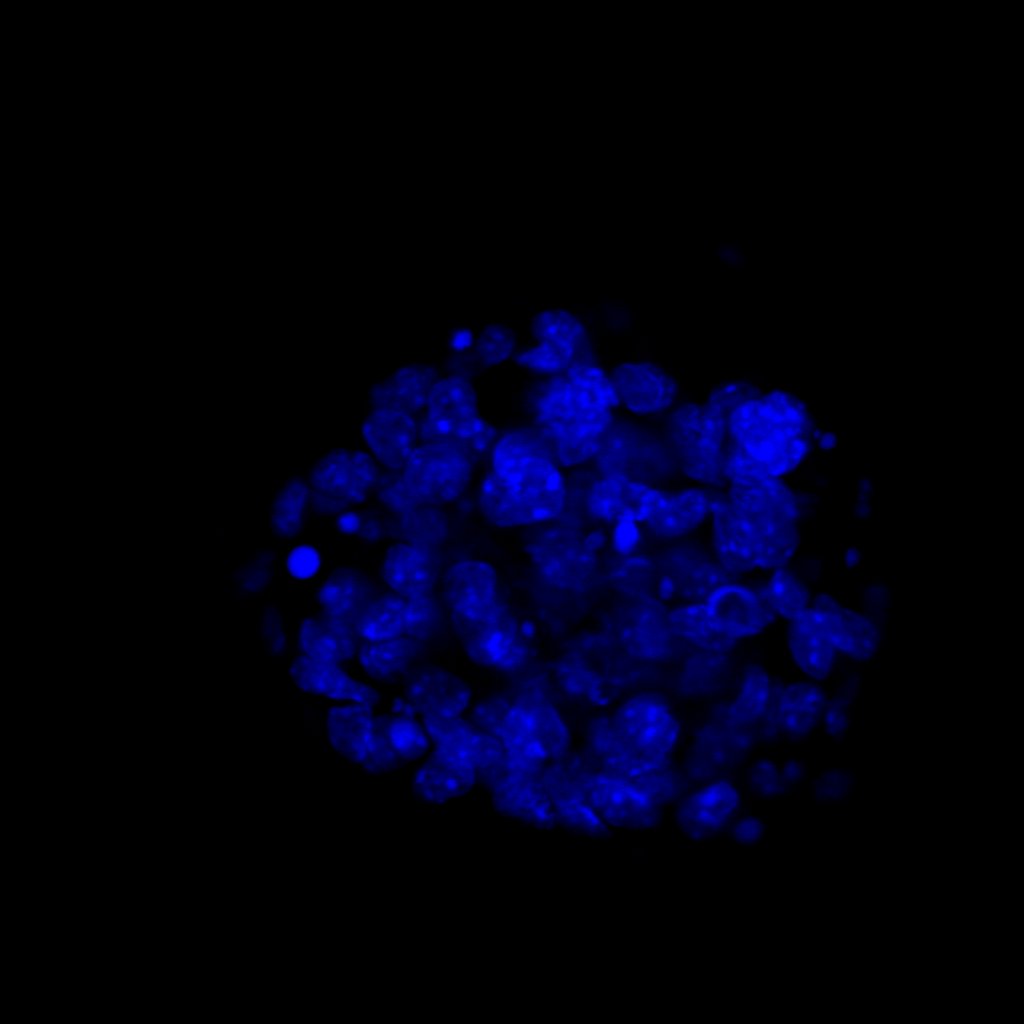

Supplement: S6 File — Representative images in the revised S2C Fig were generated from raw files woGF_4_ch1/2 (W/O GF); EF10cyc_4_ch1/2 (E+F10+Cyc); EF1shh_7_ch1/2 (E+F1+Shh). (ZIP) [file pone.0239995.s007.zip › S6_File/woGF_4_ch2.jpg]

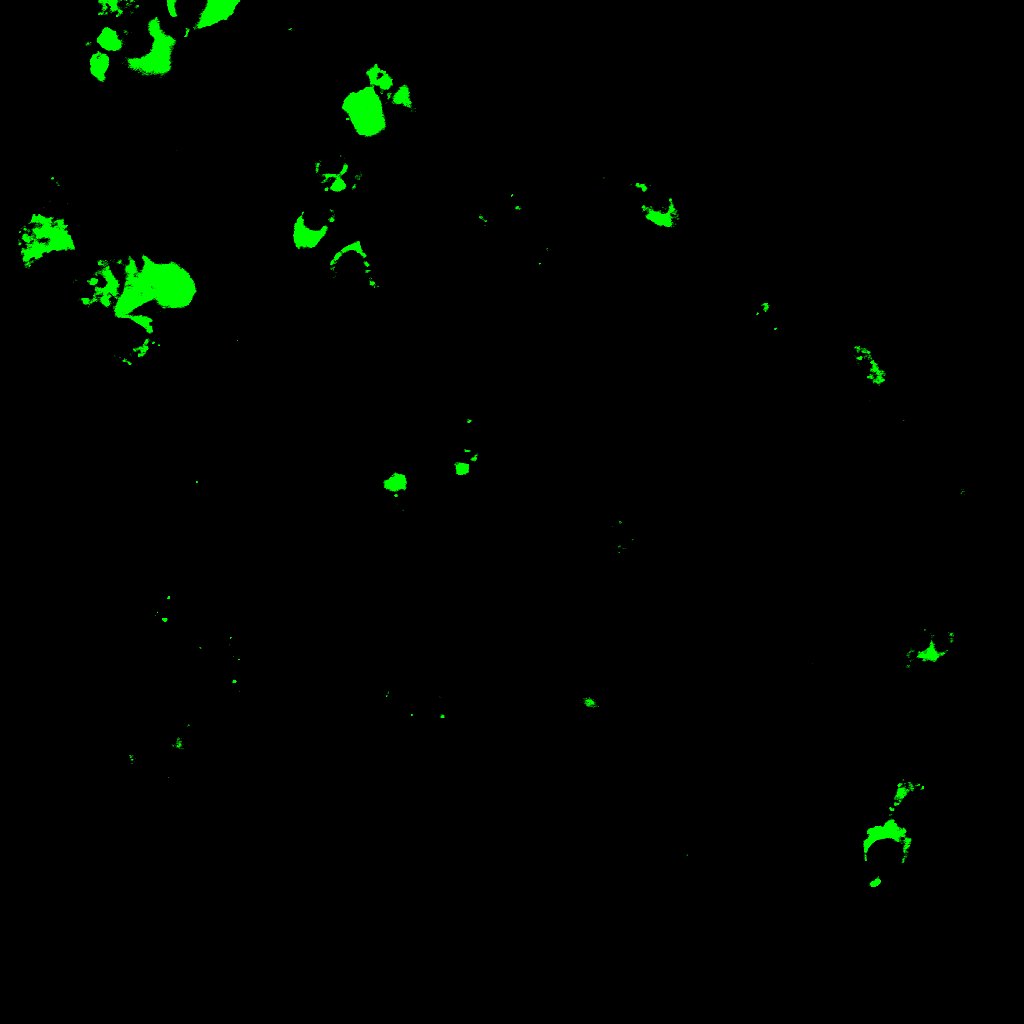

Supplement: S6 File — Representative images in the revised S2C Fig were generated from raw files woGF_4_ch1/2 (W/O GF); EF10cyc_4_ch1/2 (E+F10+Cyc); EF1shh_7_ch1/2 (E+F1+Shh). (ZIP) [file pone.0239995.s007.zip › S6_File/woGF_5_ch1.jpg]

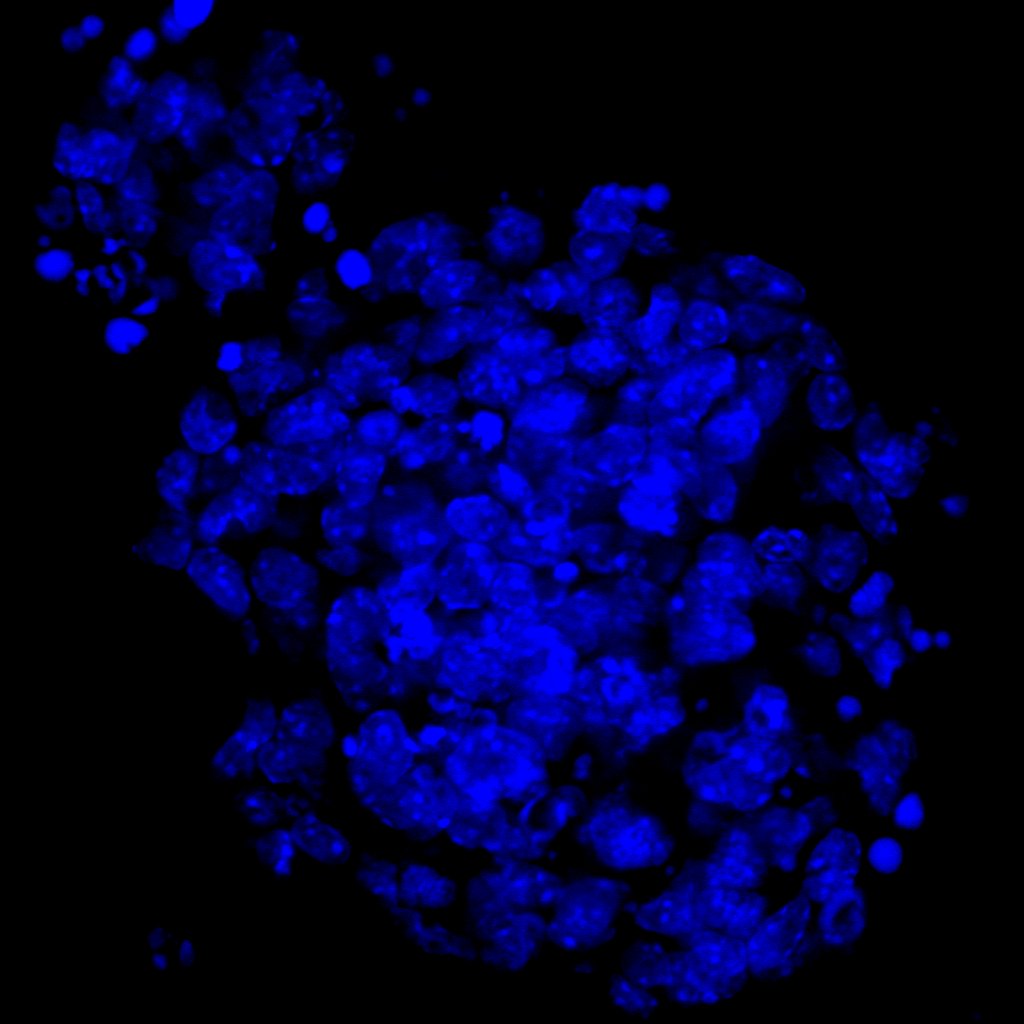

Supplement: S6 File — Representative images in the revised S2C Fig were generated from raw files woGF_4_ch1/2 (W/O GF); EF10cyc_4_ch1/2 (E+F10+Cyc); EF1shh_7_ch1/2 (E+F1+Shh). (ZIP) [file pone.0239995.s007.zip › S6_File/woGF_5_ch2.jpg]

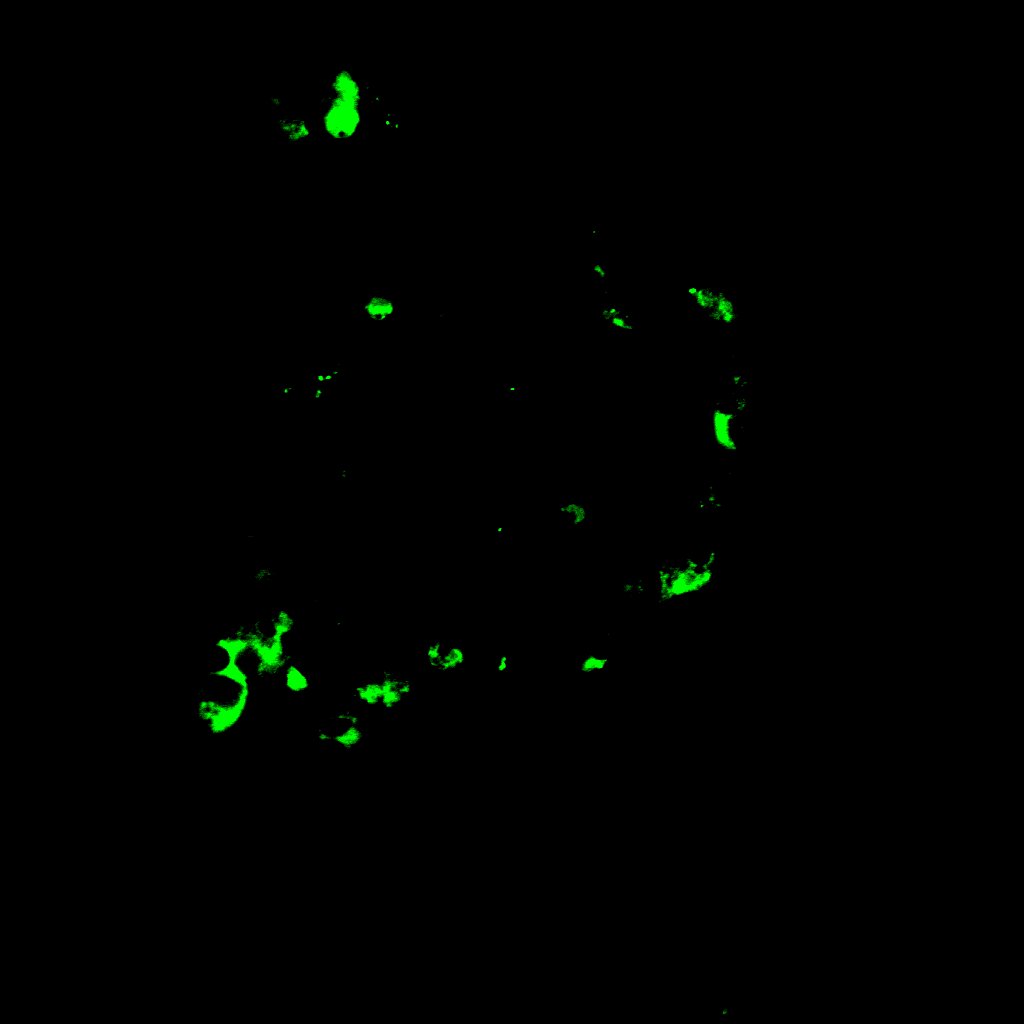

Supplement: S6 File — Representative images in the revised S2C Fig were generated from raw files woGF_4_ch1/2 (W/O GF); EF10cyc_4_ch1/2 (E+F10+Cyc); EF1shh_7_ch1/2 (E+F1+Shh). (ZIP) [file pone.0239995.s007.zip › S6_File/woGF_6_ch1.jpg]

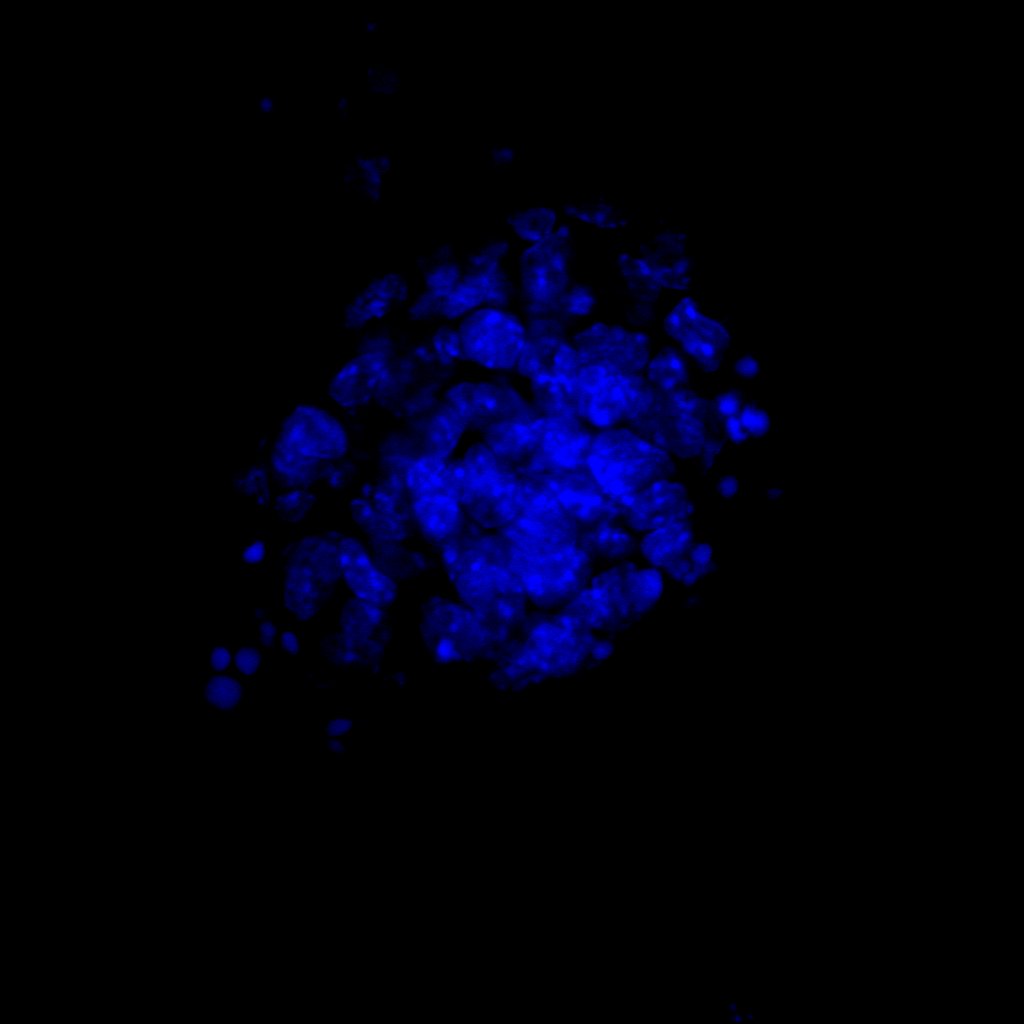

Supplement: S6 File — Representative images in the revised S2C Fig were generated from raw files woGF_4_ch1/2 (W/O GF); EF10cyc_4_ch1/2 (E+F10+Cyc); EF1shh_7_ch1/2 (E+F1+Shh). (ZIP) [file pone.0239995.s007.zip › S6_File/woGF_6_ch2.jpg]

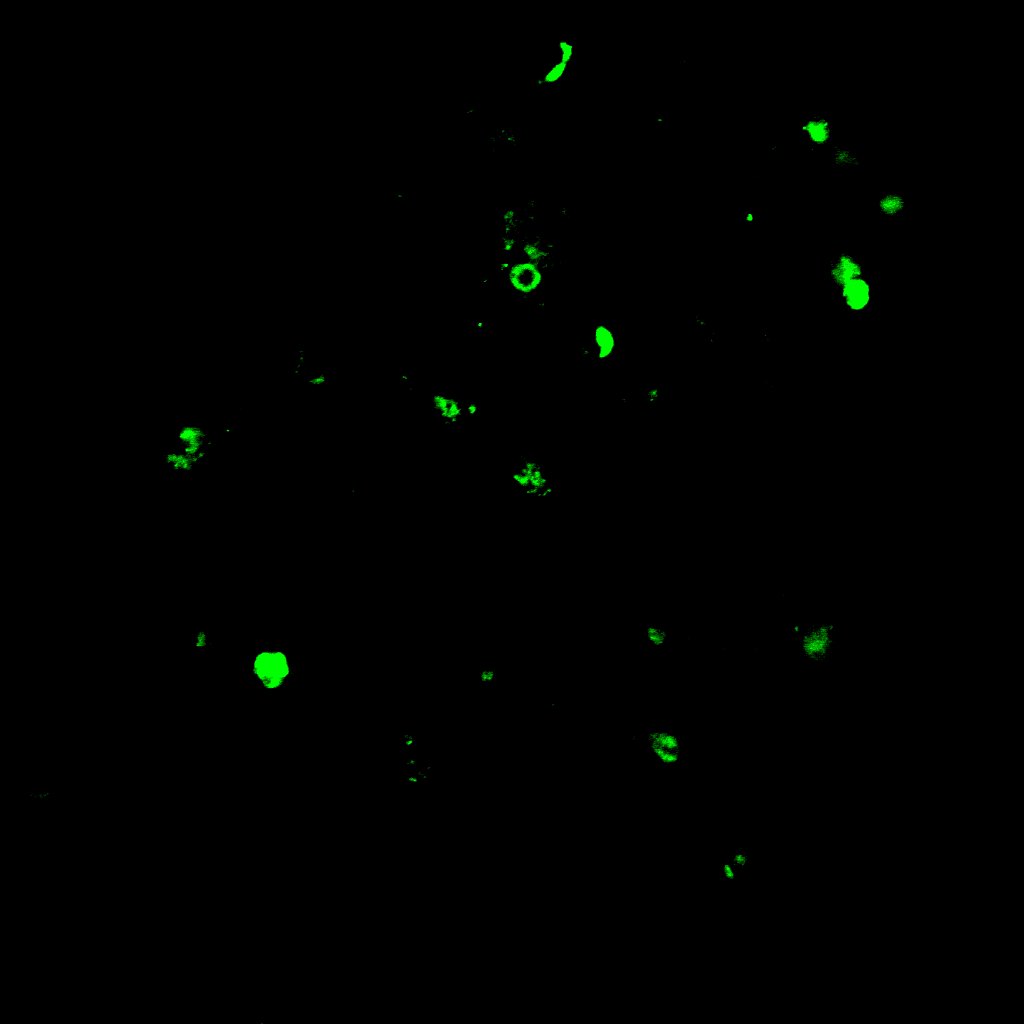

Supplement: S6 File — Representative images in the revised S2C Fig were generated from raw files woGF_4_ch1/2 (W/O GF); EF10cyc_4_ch1/2 (E+F10+Cyc); EF1shh_7_ch1/2 (E+F1+Shh). (ZIP) [file pone.0239995.s007.zip › S6_File/woGF_7_ch1.jpg]

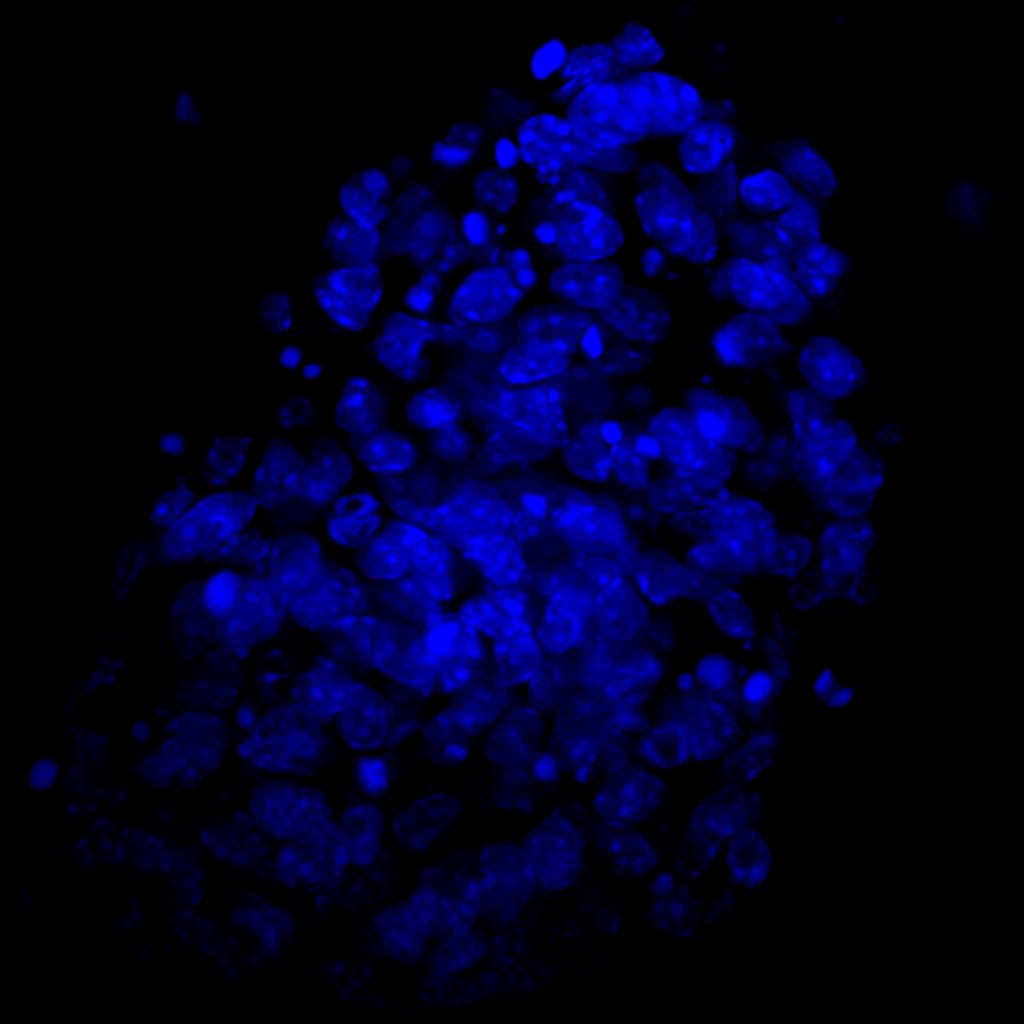

Supplement: S6 File — Representative images in the revised S2C Fig were generated from raw files woGF_4_ch1/2 (W/O GF); EF10cyc_4_ch1/2 (E+F10+Cyc); EF1shh_7_ch1/2 (E+F1+Shh). (ZIP) [file pone.0239995.s007.zip › S6_File/woGF_7_ch2.jpg]
